# Stereoselective Ferrier‐Type *O*‐Glycosylation Enabled by Difluoromethylated Glycal Donors

**DOI:** 10.1002/advs.202519766

**Published:** 2025-11-19

**Authors:** You Zou, Hengfu Xu, Cang‐Xin Zheng, Weiwei Zhang, Xin‐Shan Ye, De‐Cai Xiong

**Affiliations:** ^1^ State Key Laboratory of Natural and Biomimetic Drugs School of Pharmaceutical Sciences Peking University Xue Yuan Road No. 38 Beijing 100191 China; ^2^ School of Pharmacy North China University of Science and Technology Tangshan 063210 China; ^3^ Ningbo Institute of Marine Medicine Peking University Ningbo 315010 China

**Keywords:** difluoroalkylation, ferrier‐type reaction, stereoselective glycosylation

## Abstract

The effective construction of structurally homogeneous glycosides is requisite in oligosaccharide assembly and the development of carbohydrate‐based drugs. A fluorine‐mediated stereoselective Ferrier‐type glycosylation is reported herein. Glycals are pre‐decorated with photo‐2‐difluoroalkylation and reacted with diverse acceptors to obtain exclusively α‐selective Ferrier rearrangement products. This procedure can tolerate extensive substrates and achieve good yields. Subsequent gram‐scale and oligosaccharide syntheses further demonstrate the applicability and practicality of this strategy. Detailed density functional theory studies demonstrate the predominant stereoselectivity of the glycosylation process.

## Graphical abstract

A fluorine‐mediated stereoselective Ferrier‐type glycosylation is reported. Glycals are pre‐decorated with photo‐2‐difluoroalkylation and reacted with diverse acceptors to obtain exclusively α‐selective Ferrier rearrangement products.

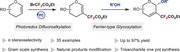

## 1 Introduction

Carbohydrates are ubiquitous natural biomolecules, ranging from cellular energy sources to molecular recognition, and are leading compounds for drug and vaccine discovery.^[^ ^1^, ^2^, ^3^, ^4^, ^5^, ^6^, ^7^, ^8^ ^]^ Increasing our understanding of these functional molecules demands significant amounts of these materials for biological, medicinal, and pharmacological studies.^[^ ^9^, ^10^, ^11^, ^12^ ^]^ Over the past decades, considerable research has been attributed to developing new glycosylation methods, especially those with high stereoselectivity (neighboring participation,^[^ ^13^, ^14^, ^15^, ^16^, ^17^ ^]^ remote group participation,^[^ ^18^, ^19^, ^20^, ^21^, ^22^, ^23^, ^24^ ^]^ conformation control,^[^ ^25^, ^26^, ^27^, ^28^ ^]^ solvent effect,^[^ ^29^, ^30^, ^31^, ^32^ ^]^ etc.^[^ ^33^, ^34^, ^35^, ^36^, ^37^ ^]^).

**Figure 1:**
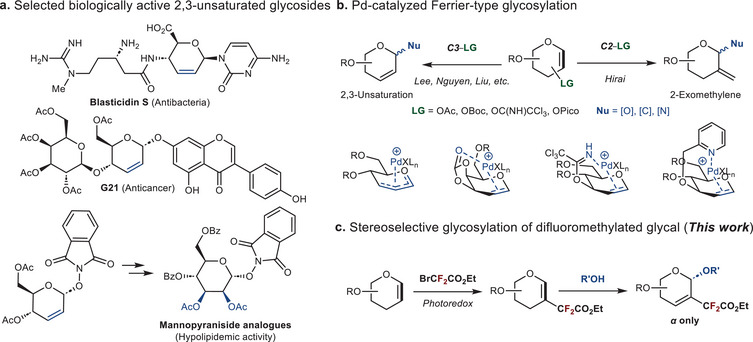
Research background of this work. a) Selected biologically active 2,3‐unsaturated glycosides. b) Pd‐catalyzed Ferrier‐type glycosylation. c) Stereoselective glycosylation of difluoromethylated glycal.

2,3‐Unsaturated glycosides and 2,3‐dideoxyglycosides are widely found in natural products and exhibit excellent antitumor and antibacterial activities.^[^ ^38^, ^39^, ^40^, ^41^, ^42^, ^43^, ^44^, ^45^ ^]^ First discovered in the 1960s, Ferrier‐type glycosylation reactions have become useful processes for direct access to 2,3‐unsaturated glycosides and subsequent convenient transformation to 2,3‐dideoxyglycosides or their analogs,^[^ ^46^, ^47^, ^48^, ^49^, ^50^, ^51^ ^]^ a widely used chiral intermediate in the preparation of biologically active compounds (**Figure** **1**a).^[^ ^41^, ^42^, ^52^, ^53^, ^54^ ^]^ However, most reported methods only offer modest stereoselectivity caused by the intrinsic effect. In recent years, carbohydrate researchers have focused on well‐designed glycosyl donors, leaving groups, and catalysts to improve the poor stereoselectivity in Ferrier rearrangement reactions. Most of the reported literatures often involve the Palladium catalysis for stereo‐control of Ferrier glycosylation products via “inner” or “outer” processes (Figure 1b).^[^ ^55^, ^56^, ^57^, ^58^, ^59^, ^60^, ^61^, ^62^, ^63^ ^]^ Even so, designing a facile and efficient stereoselective Ferrier‐type glycosylation remains a long‐standing challenge in glycochemistry.

The introduction of fluorine into a molecule at a selective site has been exploited extensively in drug design and catalyst development.^[^ ^64^, ^65^, ^66^, ^67^, ^68^, ^69^, ^70^ ^]^ Fluorination is widely recognized to modulate metabolic stability, bioavailability, and protein‐binding affinity in carbohydrate‐based molecules, as demonstrated in numerous studies.^[^ ^71^, ^72^, ^73^, ^74^, ^75^ ^]^ Fluorinated carbohydrates have notably served as chemical probes for molecular recognition.^[^ ^76^, ^77^, ^78^, ^79^ ^]^ In asymmetric synthesis, fluorinated molecules show great potential in orienting the reactivity of substrates and the development of highly stereo‐discriminating catalysts.^[^ ^80^, ^81^, ^82^, ^83^ ^]^ In addition, Gilmour et al. found that 2‐fluorohexose and 3‐fluorosialic acid donors showed remarkable stereoselectivity during glycosylation.^[^ ^84^, ^85^, ^86^ ^]^ Karban et al. also achieved similar stereoselective effects using 3‐ and 4‐fluoro analogs as donors.^[^ ^87^, ^88^, ^89^, ^90^ ^]^ These studies shed light on the use of fluorine in configuration; however, the potential of polyfluoro‐substitution via glycosylation remains unclear. Here, we envisaged a mild and practical method for stereoselective Ferrier‐Type glycosylation based on 2‐difluoromethylated glycals, as well as the efficient construction of fluorine‐bearing glucosides (Figure 1c).

## 2 Results and Discussion

### 2.1 The Reaction Condition Screening

We began our investigation with the proof‐of‐concept of stereoselective Ferrier‐type glycosylation based on 2‐difluoromethylated glycals (**Table** **1**). The model reaction was conducted using glycal **1a** as the glycosyl donor and the alcohol *^i^* PrOH as the glycosyl acceptor. Upon extensive condition screening, BF_3_·Et_2_O was deemed the optimal promoter, generating the desired Ferrier‐Type product **2a** with exclusive α‐selectivity in CH_2_Cl_2_ (Table 1, entry 1). Both the promoter and solvent can affect the reaction results (Table 1, entry 2; Tables S1 and S2, Supporting Information). Moreover, the promoter proved to be essential to our reaction outcome, which impaired the yield at high and low dosages (Table 1, entries 5,6; Table S3, Supporting Information). The temperature and time also had some influence on the yield, with 0 °C and 30 min being selected as the ideal parameters (Table 1, entries 3,4; Tables S4 and S5, Supporting Information). Decreasing the amount of donor reduced the yield, indicating the potential side reaction of the vulnerable donor catalyzed by BF_3_·Et_2_O (Table 1, entries 7,8; Table S6, Supporting Information). Fluorine substituents proved to be essential to our reaction outcome. According to the control experiments, fluorine‐containing donors **1a**, **1b,** and **1c** afforded α stereoselective products (Table 1, entries 1, 9, and 10), while the fluorine‐free donors **1d** and **1e** provided products with poor stereoselectivity (Table 1, entries 11,12). The initial comparative experiments suggested that stereoselective Ferrier‐type glycosylation could be achieved with 2‐fluoroalkylated glycals.

**Table 1:** Reaction optimization and proof‐of‐concept.

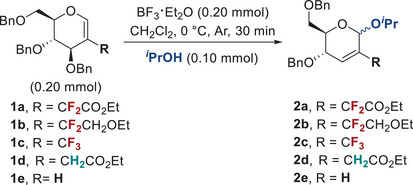			
Entry^a)^	Deviation from above	2 (Yield)^b)^	α/β ratio^c)^
1	none	**2a** (90%)	>20:1
2	THF	**2a** (15%)	>20:1
3	at ‐5 °C	**2a** (68%)	>20:1
4	at 5 °C	**2a** (81%)	>20:1
5	BF_3_·Et_2_O (0.15 mmol)	**2a** (65%)	>20:1
6	BF_3_·Et_2_O (0.25 mmol)	**2a** (71%)	>20:1
7	**1a** (0.05 mmol)	**2a** (49%)	>20:1
8	**1a** (0.10 mmol)	**2a** (73%)	>20:1
9	**1b** instead of **1a**	**2b** (65%)	>20:1
10	**1c** instead of **1a**	**2c** (82%)	>20:1
11	**1d** instead of **1a**	**2d** (79%)	4:1
12	**1e** instead of **1a**	**2e** (72%)	3:1

^a)^ Reaction conditions: glycal **1** (0.20 mmol), *^i^* PrOH (0.10 mmol), BF_3_·Et_2_O (0.20 mmol) at 0 °C under an argon atmosphere and reacted for 30 min;

^b)^ Isolated yield;

^c)^ The α/β ratio was determined by the ^1^H NMR analysis of the crude reaction mixture.

### 2.2 The Scope of Substrate

#### 2.2.1 Preparation of 2‐Fluoroalkylated Glycals

**Figure 2:**
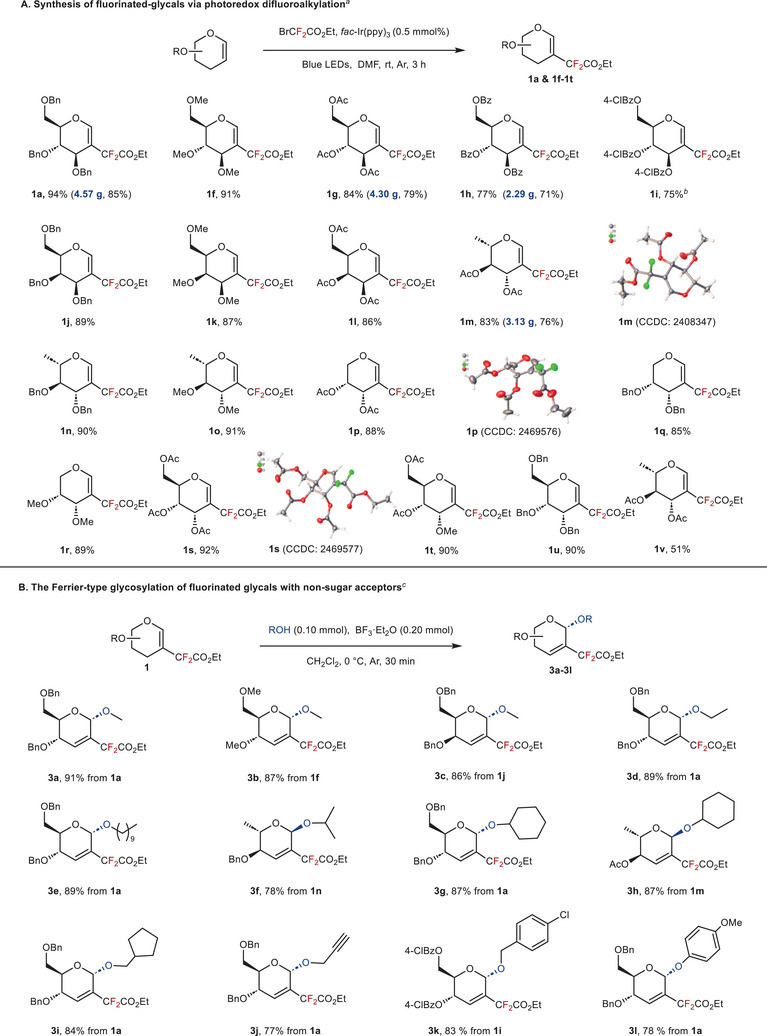
Substrate scope with yields of the isolated products. ^[a]^ Standard conditions: glycal (0.10 mmol), BrCF_2_CO_2_Et (0.30 mmol), *fac*‐Ir(ppy)_3_ (0.5 mol%), dry DMF (2 mL), Ar, rt. ^[b]^4‐ClBz refers to 4‐chlorobenzoyl. ^[c]^Standard conditions: difluoroalkylated glycal donor (0.20 mmol), acceptor (0.10 mmol), BF_3_·Et_2_O (0.20 mmol), dry CH_2_Cl_2_ (2 mL), Ar, 0 °C.

To test the universality of stereoselective Ferrier‐glycosylation in the fluorine‐bearing glycal, we first constructed a series of 2‐difluoroalkylated glycals by photoredox reaction (**Figure** **2**a). Different types of fluorinated glycals (e.g., D‐glucal, d‐galactal, L‐rhamnal, D‐arabinal and d‐allose) provided the corresponding products in moderate to high yields (51% to 94% yields, 18 examples), and gram scale synthesis of this 2‐difluoroalkylation can also be achieved (**1a**, **1g**, **1h,** and **1m**).

#### 2.2.2 Scope of Ferrier‐Glycosylation

With the optimal conditions in hand, we next investigated the scope of Ferrier glycosylation. Under standard reaction conditions, both primary and secondary alcohols successfully deliver the glycosides **3a**–**3j** in good yields with excellent stereoselectivity (Figure 2b). For highly active methanol and ethanol compounds, which usually offer poor glycosylation stereoselectivity, the desired Ferrier‐type products were obtained (**3a**–**3d**). Acceptors with aryl rings (benzyl alcohol and phenol) also gave α‐only products (**3k**,**3l**). Additionally, methyl‐, acetyl‐, and 4‐chlorobenzoyl‐protected donors were well‐tolerated to give glycosides in good yields (**3b**, **3h**, **3k**).

**Figure 3:**
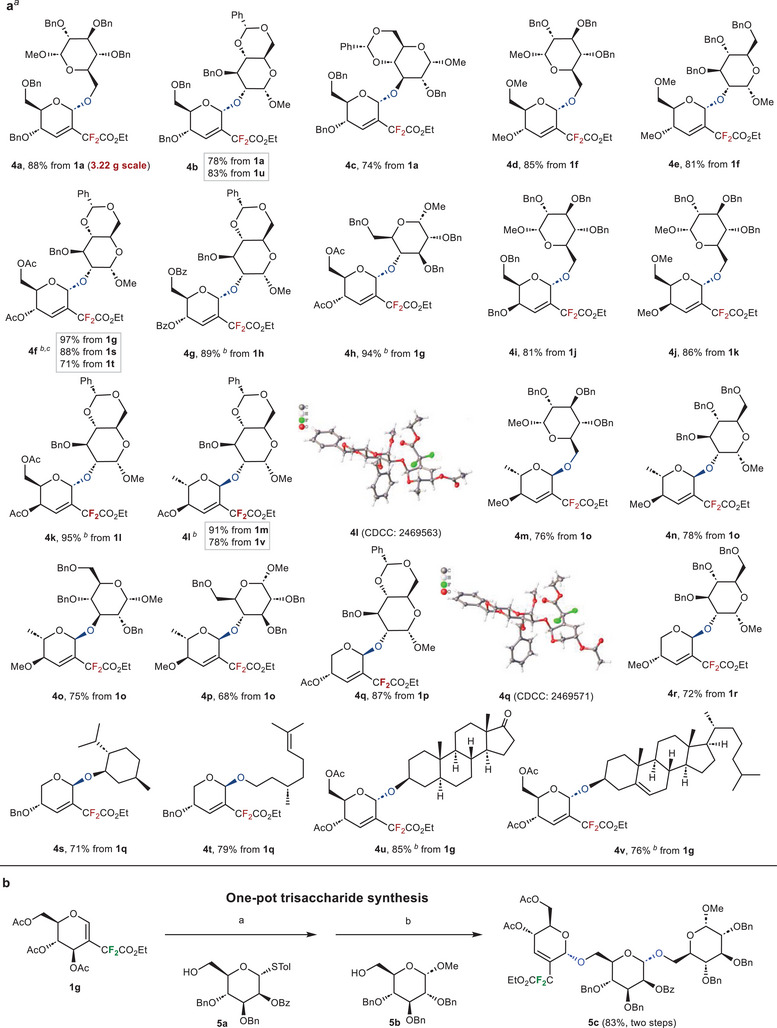
a) Substrate scope with yields of isolated products. ^[a]^Standard conditions: difluoroalkylated glycal donor (0.20 mmol), acceptor (0.10 mmol), BF_3_·Et_2_O (0.20 mmol), dry CH_2_Cl_2_ (2 mL), Ar, 0 °C. ^[b]^0.40 mmol BF_3_·Et_2_O was used. ^[c]^CDCC: 2474200. The single crystal belongs to **4f‐OH,** which undergoes the deacetylation of **4f** (see Figure S4, Supporting Information). b) One‐pot trisaccharide synthesis. Reaction conditions: a. donor **2l** (0.30 mmol), acceptor **5a** (0.15 mmol), BF_3_·Et_2_O (0.60 mmol), dry CH_2_Cl_2_ (2 mL), Ar, 0 °C; b. acceptor **5b** (0.15 mmol), AgOTf (0.30 mmol), *p*‐TolSCl (0.20 mmol), TTBP (0.18 mmol), dry CH_2_Cl_2_ (3 mL), Ar, −78 °C.

Next, we applied our established glycosylation protocol to sugar acceptors and natural products. As shown in **Figure** **3**a, the reaction occurred smoothly under standard conditions, affording the desired α‐stereoselective products (**4a‐**‐**4v**). The reaction occurred in 2‐difluoroalkylated glucals and galactals with various protecting groups, giving high yields, while rhamnal and arabinal were identified as suitable substrates, delivering modest yields. This impaired performance may be due to their high reactivity, making them vulnerable to promoters. Glycals containing electron‐withdrawing groups (e.g., Ac─ and Bz─) always produced high yields compared to those containing electron‐donating groups (e.g., Bn─ and Me─). In addition, reactions with different kinds of sugar acceptors were also permissible and delivered the desired glycosides in satisfactory yields. Lastly, we focused on our glycosylation study in the late‐stage modification of natural products and drugs. Menthol and citronellol have specific aromas and are used as refreshing agents and antibacterial drugs to achieve the expected glycosylation products (**4s** and **4t**). Steroids with complex structures were also well tolerated during the reaction (**4u** and **4v**), which further supported the practicability of our fluorine‐mediated stereoselective Ferrier‐type glycosylation. In addition, gram scale synthesis (**4a**) also demonstrated the method's reliability of this reaction. Moreover, it should be noted that the product configuration of d‐glucals, d‐galactals and l‐rhamnals was analyzed as the α isomer while the d‐arabinals gave the β isomers. Such a situation of d‐arabinals may be responsible to its more stable 1,4‐*trans* conformation. These results have been further confirmed by X‐ray structure (**4f**, **4l,** and **4k**) and NMR NOESY experiments using several representative structures (Figures S1–S4, Supporting Information).

### 2.3 Synthetic Presentation

To further expand the synthetic potential of this method, the one‐pot synthesis of an oligosaccharide was presented in our work (Figure 3b). The 2‐difluoroalkylated donor **1g** was first reacted with acceptor **5a** to furnish the disaccharide within 3 h under Ferrier‐type glycosylation. The subsequent in situ “pre‐activation” of the Ferrier‐type disaccharide at −78 °C followed by the addition of acceptor **5b** effectively delivered the trisaccharide **5c** in moderate yield (83%, two steps). These results further demonstrated the application of our method in oligosaccharide synthesis.

### 2.4 Mechanism Studies

**Figure 4:**
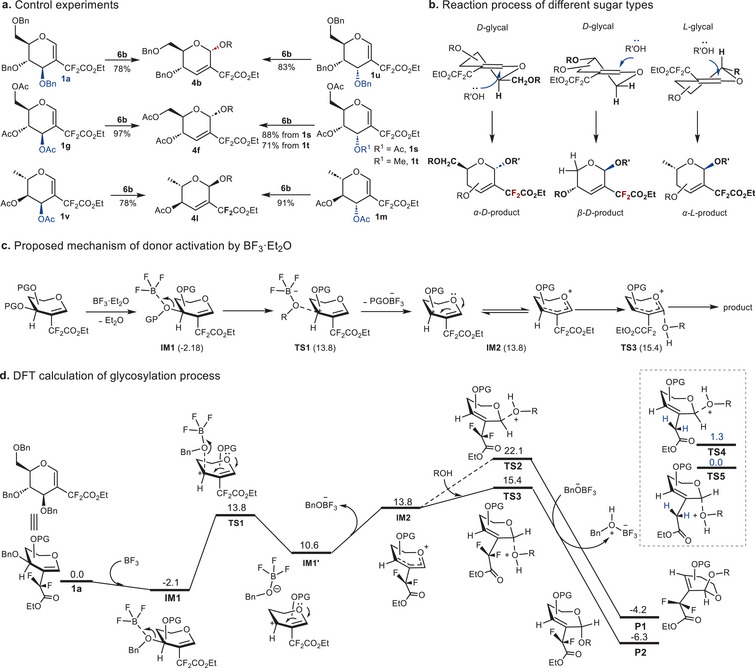
a) Control experiments. b) Reaction process of different sugar types. c) Proposed mechanism of donor activation by BF_3_·Et_2_O. d) DFT calculation of the glycosylation process. Glycal **1a** and acceptor *^i^* PrOH were used in computational experiments. Energy measurements were expressed in kcal mol^−1^.

Control experiments were conducted to investigate the influence of substituents at the C3, C4, and C5 positions on product configuration (**Figures** **4**a,b; S5, Supporting Information). Comparison of glycosylation outcomes among D‐glucals (**1a**, **1f**, **1g**), D‐alloses (**1s**, **1t**, **1u**), and D‐galactals (**1j**, **1k**, **1l**) revealed that the bond orientation at the C3 and C4 positions of the donor does not significantly affect stereoselectivity. In contrast, the substituent at the C5 position plays a critical role, leading to the predominant formation of 1,5‐trans glycoside products. Notably, D‐arabinals featuring a free C5 position afforded 1,4‐trans glycoside products, which can be rationalized by the favorable conformation of the pyranose ring. Based on these control experiments, a possible S_N_2’‐type mechanism involving the C3‐leaving group was ruled out.^[^ ^91^ ^]^

To gain further insight into the stereoselective Ferrier‐type glycosylation process, density functional theory (DFT) calculations were performed using D‐glucal **1a**. Similar to the classical Ferrier type I glycosylation, the reaction proceeds in two steps. Initially, the 2‐difluoroalkylated donor is activated. As illustrated in Figure 4c, the donor interacts with the promoter BF_3_·Et_2_O (**IM1**) via the lone pair of the C3 oxygen atom, progressing through transition state **TS1**. Subsequent B─O bond formation weakens the C─O bond of the donor, ultimately leading to the formation of a delocalized sugar cation (**IM2**) upon departure of the PGOBF_3−_ group. Glycosylation constitutes the rate‐determining step governing stereoselectivity (Figure 4d). According to the S_N_1‐type carbocation mechanism, the ROH acceptor may attack the anomeric C1 center from either the α‐ or β‐face. However, the presence of fluorine substitution enhances the reactivity of the delocalized carbocation, facilitating rapid nucleophilic attack. As shown in the energy profile, a notable energy difference is observed between **TS3** (15.4 kcal mol^−1^) and **TS2** (22.1 kcal mol^−1^). In the absence of fluorine substitution, the energy gap between corresponding transition states (**TS4** and **TS5**) is only 1.3 kcal mol^−1^. Additionally, the free energies of the products (**P2**: −6.3 kcal mol^−1^; **P1**: −4.2 kcal mol^−1^) further support the preference for the α‐product in the Ferrier‐type glycosylation of difluoromethylated glyals.

## 3 Conclusion

To conclude, a practical Ferrier‐type glycosylation with predominant stereoselectivity was established using difluoromethylated glycal donors (18 examples), which were well‐prepared via photoredox functionalization. The proof‐of‐concept experiment emphasized the necessity of C‐2 fluoroalkylation in glycals for their subsequent stereoselective glycosylation, and a series of 35 glycosylation products with high yields and exclusive stereoselectivity, including diverse donors and acceptors, were presented. Gram‐scale synthesis and one‐pot trisaccharide assembly suggested the practicability of our method in homogeneous oligosaccharide acquisition. Subsequent control experiments and DFT investigations further demonstrated the stereoselectivity of glycosylation with regard to the solvated free energy. Given the large abundance of 2,3‐unsaturated glycosides and 2,3‐deoxyglycosides in natural products and the increasing advantages of drug fluorination, this method will have wide applicability in carbohydrate chemistry and carbohydrate‐based drugs.

## Conflict of Interest

The authors declare no conflict of interest.

## Author Contributions

Y.Z., H.X., and C.‐X.Z. contributed equally to this work. D.‐C.X. conceived and designed the research. Y.Z. performed experiments with the help of H.X., C.‐X.Z., and W.Z. The data were analyzed and organized by Y.Z. and C.‐X.Z. D.‐C.X., Y.Z., and C.‐X.Z. wrote the manuscript with input from all authors. D.‐C.X. and X.‐S.Y. directed the project.

## Supporting information



Supporting Information

## Data Availability

The data that support the findings of this study are available in the supplementary material of this article.
