# Supplementary material for: Stereoselective Ferrier‐Type O‐Glycosylation Enabled by Difluoromethylated Glycal Donors
Source: Adv Sci (Weinh). 2025 Nov 19;13(7):e19766. doi: 10.1002/advs.202519766 (PMC12866755; doi:10.1002/advs.202519766)
Supplement: Supplementary file 1 — Supporting Information [file ADVS-13-e19766-s001.pdf]

Supporting Information

Stereoselective Ferrier-Type *O*-Glycosylation Enabled by Difluoromethylated-Glycal Donors

You Zou+[a], Hengfu Xu+[a], Cang-Xin Zheng+[a], Weiwei Zhang [a][b], Xin-Shan Ye *[a], and De-Cai Xiong *[a][c]

[a] State Key Laboratory of Natural and Biomimetic Drugs, School of Pharmaceutical Sciences
Peking University, Xue Yuan Road No. 38, Beijing 100191, China

[b] School of Pharmacy, North China University of Science and Technology, Tangshan 063210, China.

[c] Ningbo Institute of Marine Medicine, Peking University, Ningbo 315010, China.
E-mail: decai@bjmu.edu.cn, xinshan@bjmu.edu.cn

[+] These authors contributed equally to this work.

Table of content

1. General experimental information and reaction procedure 2
1.1 General experimental information 2
1.2 General photoredox difluoroalkylation procedure 2
1.3 General glycosylation procedure 2
2. Optimization of reactions 3
Table S1. Preliminary experiments screening 3
Table S2. The catalysts screening 3
Table S3. The dosage of catalyst screening 4
Table S4. The reaction time screening 4
Table S5. The reaction temperature screening 4
Table S6. The ratio of donor/acceptor screening 5
3. The configuration confirmation of 2,3-unsaturated glycosides 5
4. Reaction scope 7
4.1 Photoredox difluoroalkylation scope 7
4.2 Glycosylation scope 15
5. X-Ray Crystallographic Data 40
Table S7 . X-ray crystallographic data of compound 1m 40
Table S8 . X-ray crystallographic data of compound 1p 41
Table S9 . X-ray crystallographic data of compound 1s 42
Table S10 . X-ray crystallographic data of compound 4l 43
Table S11 . X-ray crystallographic data of compound 4q 44
6. Computational Methods 45
7. References 66
8. NMR Spectra 68

1. General experimental information and reaction procedure

1.1 General experimental information

All commercially-available reagents were used without further purification. CH_2Cl_2 was distilled over CaH_2. Reactions were monitored by thin-layer chromatography (TLC) on silica gel-coated aluminium plates (60 F_{254}, E. Merck). Reaction spots were detected under UV light (254 nm) and chromogenic agent ($(NH_4)_6Mo_7O_{24}\cdot 4H_2O$ (24.00 g, 19.4 mmol), $Ce(NH_4)_2(NO_3)_6$ (0.50 g, 0.90 mmol) in sulfuric acid (5%, 500 mL)). Column chromatography was performed on silica gel (200-300 mesh from Qingdao Ocean Chemical Co. Ltd.). ^1H NMR and ^{19}F NMR spectra were recorded on Bruker AV 400 (400 MHz) or Bruker AV 600 MHz (600 MHz) at room temperature in $CDCl_3$ with TMS (δ = 0 ppm) as internal standard. ^{13}C NMR spectra were obtained using the coincident NMR spectrometer and calibrated with $CDCl_3$ (δ = 77.16 ppm). The following standard abbreviations involving in NMR data are used to indicate multiplicity: s = singlet, d = doublet, t = triplet, q = quartet, m = multiplet, dd = doublet of doublets, td = triplet of doublets, br = broad. High-resolution mass spectra (HRMS) were performed on a Waters XE VO-G2QTOF instrument. Photoreaction experiments were carried out using four 12W Blue LED lamps (450-470 nm).

1.2 General photoredox difluoroalkylation procedure

To an oven-dried 10 mL Schlenk flask with a Teflon-coated magnetic stir bar were added glycal (0.10 mmol), *fac*-Ir(ppy)$_3$ (0.5 mol%) and $BrCF_2CO_2Et$ (0.30 mmol). The flask was evacuated and sealed, and refilled with Ar for three times. Then dried DMF (2 mL) was injected to the flask dissolving the above reactants. The reaction mixture was stirred under irradiation with blue LEDs (12 W × 4, 450-470 nm) at ambient temperature. After reaction completion monitored by TLC, the reaction mixture was concentrated under reduced pressure followed by column chromatography purification on silica gel to give the target product.

1.3 General glycosylation procedure

Procedure A:

An oven-dried 10 mL flask with a Teflon-coated magnetic stir bar was evacuated and dried. Then difluoroalkylated glycal donor (0.20 mmol) and acceptor (0.10 mmol) were added to the flask, and the system was refilled with Ar for three times, followed by the addition of CH_2Cl_2 (2 mL) to dissolve the above materials. The mixture was stirred at ice-water bath for 10 min, and then $BF_3\cdot Et_2O$ (0.20 mmol) was added dropwise to the system. After reaction completion about 30 min monitored by TLC, Et_3N (0.2 mL) was added to quench the reaction. The reaction mixture was concentrated under reduced pressure followed by column chromatography purification on silica gel to give the target product.

Procedure B:

An oven-dried 10 mL flask with a Teflon-coated magnetic stir bar was evacuated and dried. Then difluoroalkylated glycal donor (0.20 mmol) and acceptor (0.10 mmol) were added to the flask, and the system was refilled with Ar for three times, followed by the addition of CH_2Cl_2 (2 mL) to dissolve the above materials. The mixture was stirred at ice-water bath

for 10 min, and then $BF_3 \cdot Et_2O$ (0.40 mmol) was added dropwise to the system. After reaction completion about 60 min monitored by TLC, Et_3N (0.2 mL) was added to quench the reaction. The reaction mixture was concentrated under reduced pressure followed by column chromatography purification on silica gel to give the target product.

2. Optimization of reactions

Table S1. Preliminary experiments screening

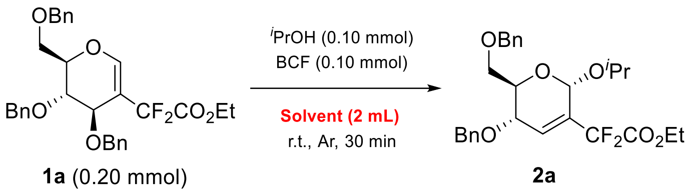

Entry	Solvent	Yield (%)[a]
1	CH_2Cl_2	34
2	CH_3CN	< 5
3	THF	0
4	Toluene	0
5	DME	0
6	Acetone	0
7	DMF	0
8	Toluene	0
9	DMA	0

[a] Isolated yield

Table S2. The catalysts screening

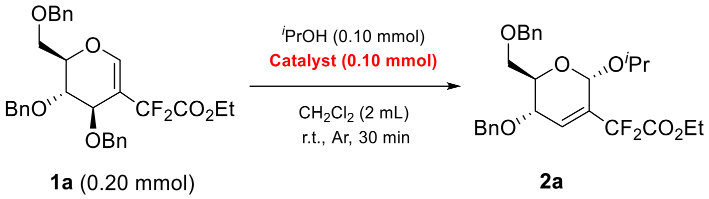

Entry	Catalyst	Yield (%)[a]
1	no catalyst	0
2	BCF	34
3	$Cu(OTf)_2$	0
4	TMSOTf	0
5	$BF_3 \cdot Et_2O$	53
6	BCl_3	0
7	BBr_3	0

[a] Isolated yield

Table S3. The dosage of catalyst screening

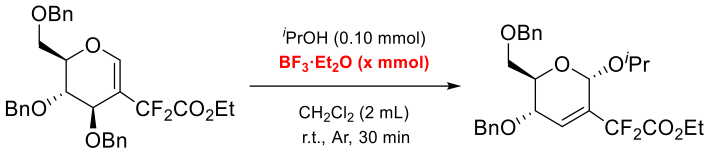

1a (0.20 mmol) **2a**

Entry	$BF_3 \cdot Et_2O$ (x mmol)	Yield (%)[a]
1	0.05 mmol	43
2	0.10 mmol	53
3	0.15 mmol	57
4	0.20 mmol	77
5	0.25 mmol	63

[a] Isolated yield

Table S4. The reaction time screening

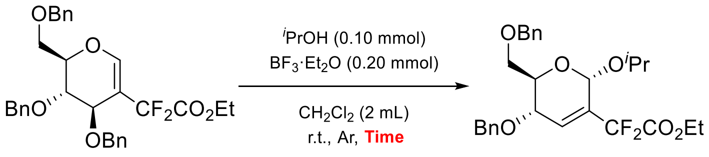

1a (0.20 mmol) **2a**

Entry	Time	Yield (%)[a]
1	10 min	23
2	20 min	52
3	30 min	77
4	40 min	75
5	50 min	66
6	60 min	58
7	90 min	49

[a] Isolated yield

Table S5. The reaction temperature screening

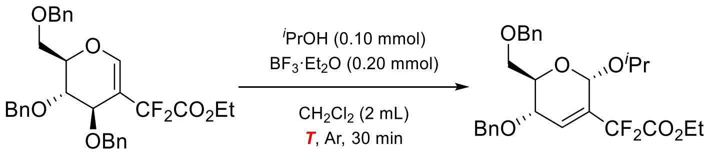

1a (0.20 mmol) **2a**

Entry	Temperature (*T*)	Yield (%)[a]
1	-20 ℃	< 5
2	-10 ℃	53
3	-5 ℃	68
4	0 ℃	89

5	5 ℃	81
6	10 ℃	79
7	20 ℃	76

[a] Isolated yield

Table S6. The ratio of donor/acceptor screening

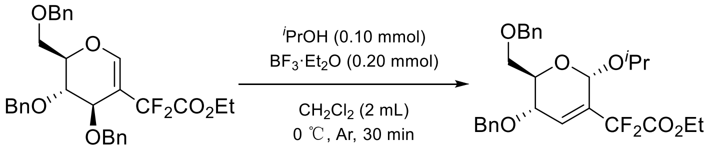

1a (0.20 mmol) **2a**

Entry	Ratio (Donor : Acceptor)	Yield (%)[a]
1	1 : 2	49
2	1 : 1	73
3	1.5 : 1	82
4	2 : 1	**90**
5	2.5 : 1	89

[a] Isolated yield

3. The configuration confirmation of 2,3-unsaturated glycosides

The anomeric configuration was confirmed based on 1 D NOESY. The correlation of H-1 and H-4 will be presented. (Taking **3k** for example)

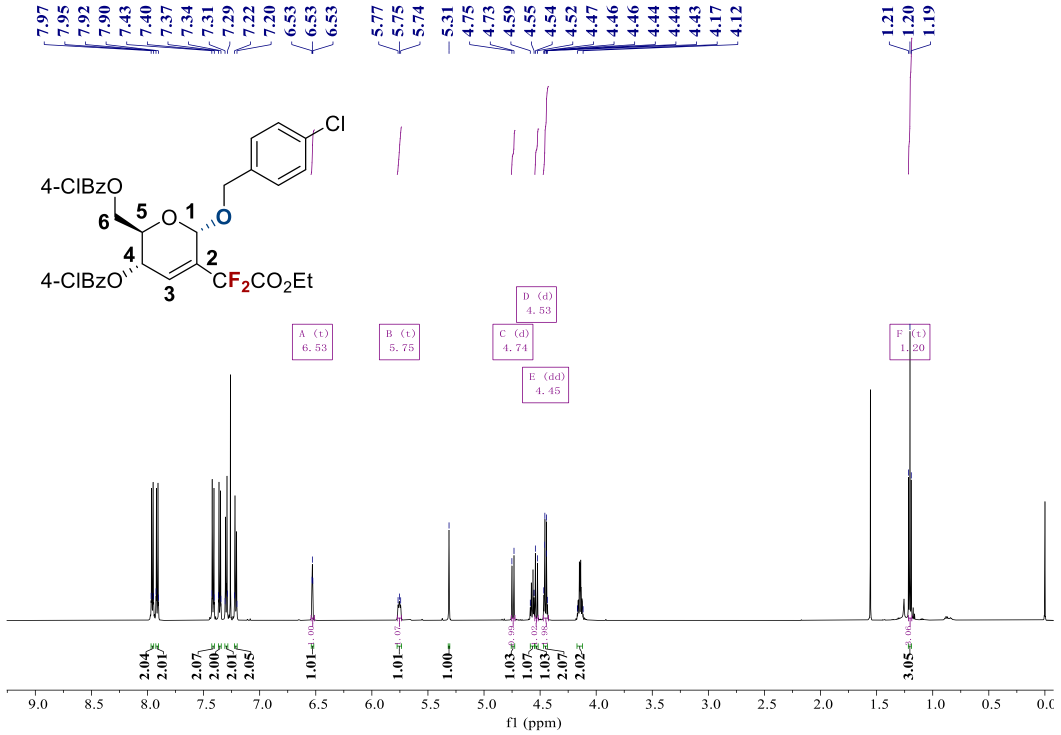

Fig S1. ^{1}H NMR of Compound **3k** (600 MHz, $CDCl_3$)

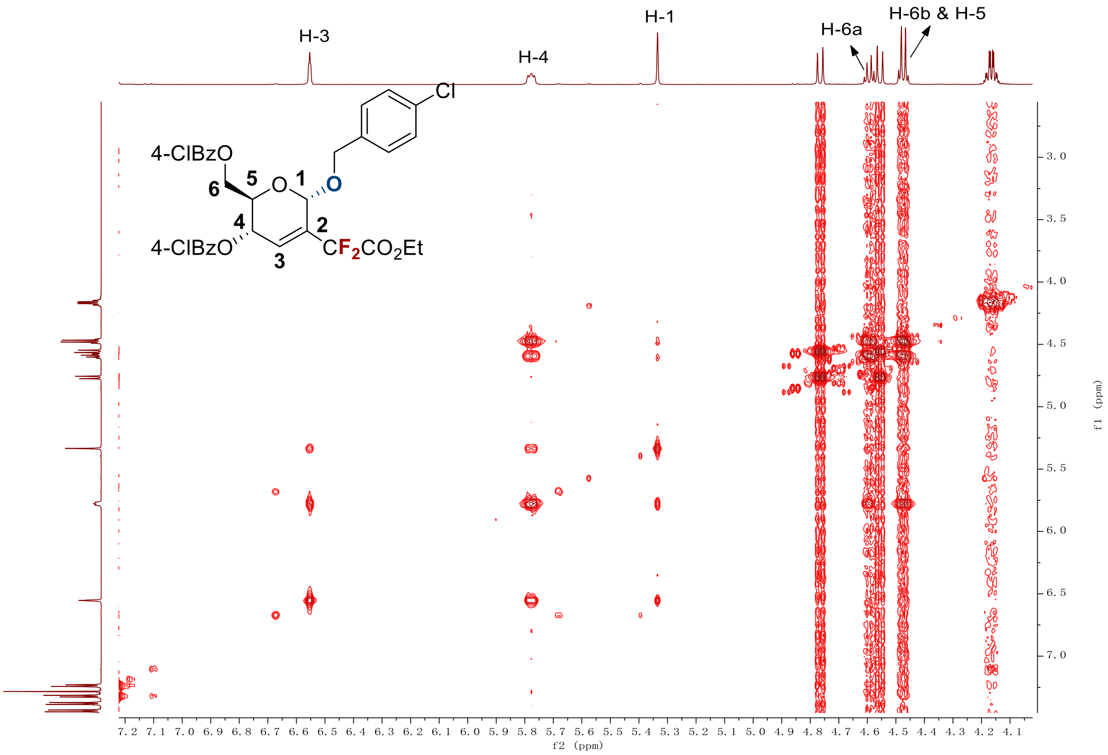

Fig S2. ^{1}H-^{1}H COSY of Compound **3k** (600 MHz, $CDCl_3$)

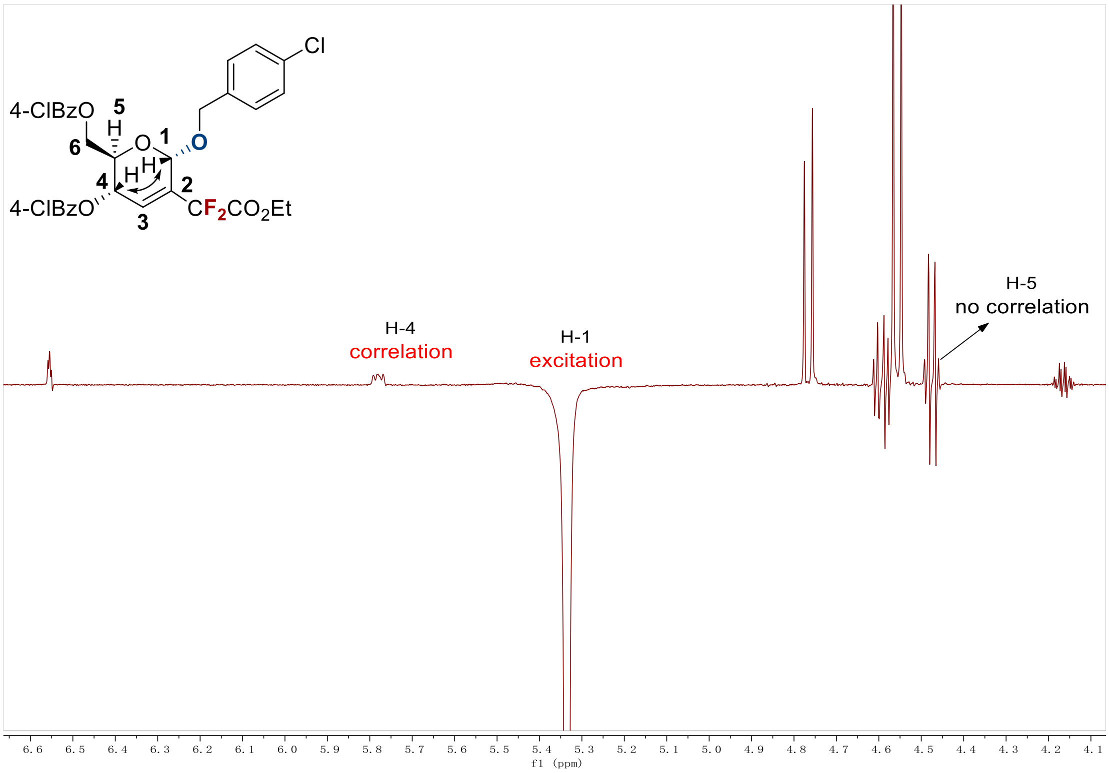

Fig S3. 1 D NOESY of Compound **3k** (H-1 was excited)

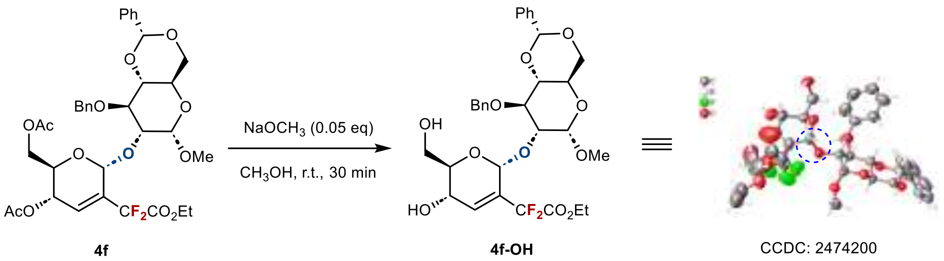

Fig S4. The anomeric configuration conformation of disaccharide **4f**. The single crystal of **4f** is hardly to obtain, so we conducted the deacetylation step affording compound **4f-OH**, followed by obtaining its X-ray structure.

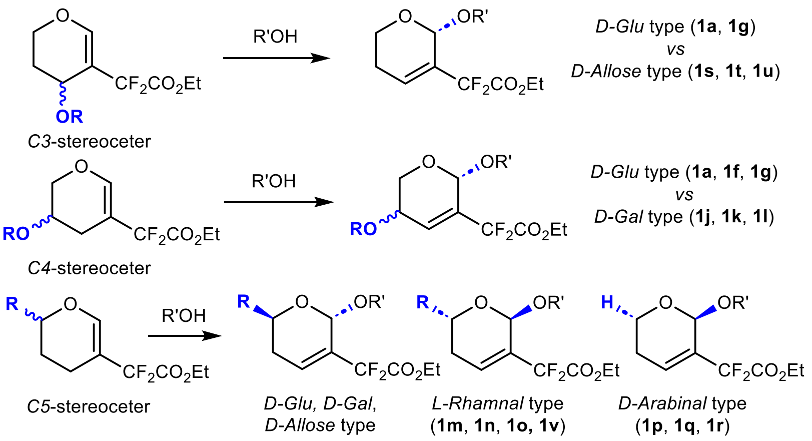

Fig S5. Control experiments.

4. Reaction scope

4.1 Photoredox difluoroalkylation scope

The synthesis of compound 1a

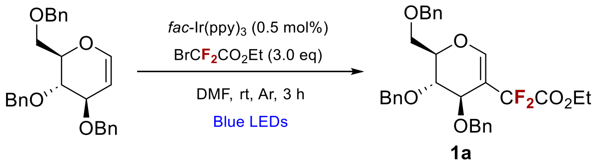

According to the *General photoredox difluoroalkylation procedure*, reaction mixture was purified by column chromatography on silica gel (petroleum ether : ethyl acetate = 20 :1) to give **1a** as a colorless oil (50.6 mg, 94%). ^{1}H NMR (400 MHz, $CDCl_3$) δ 7.35-7.24 (m, 13H), 7.22-7.19 (m, 2H), 6.97 (d, J = 2.6 Hz, 1H), 4.62-4.55 (m, 2H), 4.52-4.40 (m, 5H), 4.15-4.01 (m, 3H), 3.87 (t, J = 3.9 Hz, 1H), 3.76 (dd, J = 10.6, 6.5 Hz, 1H), 3.65 (dd, J = 10.6, 4.7 Hz, 1H), 1.14 (t, J = 7.1 Hz, 3H); ^{19}F NMR (376 MHz, $CDCl_3$) δ -102.73 (d, J = 256.0 Hz, 1F), -108.76 (d, J = 256.1 Hz, 1F). The ^{1}H NMR and ^{19}F NMR data are in accordance with those reported previously.[1]

The synthesis of compound 1b

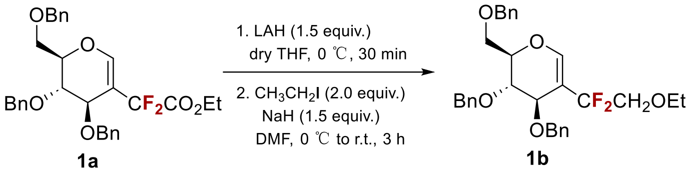

To an oven-dried 25 mL round-bottom flask with a Teflon-coated magnetic stir bar was added glycal **1a** (0.64 g, 1.19 mmol). Then dry THF (5 mL) was added to the flask dissolving the above reactant, followed by the addition of $LiAlH_4$ (0.71 mL, 2.5 N in THF) at 0 ℃. The reaction mixture was stirred for 30 min. After reaction completion monitored by TLC, the reaction mixture was slowly quenched by ice water at 0 ℃. The mixture was filtered and the filtrate was concentrated under reduced pressure followed by column chromatography purification on silica gel (petroleum ether : ethyl acetate = 10 :1) to give an oil. The oil was dissolved in 5 mL DMF and stirred at 0 ℃ for 5 min. Then the CH_3CH_2I (2.0 equiv.) and NaH (1.5 equiv.) were added to the reaction mixture. After reaction completion monitored by TLC, the reaction mixture was slowly quenched by ice water at 0 ℃. The mixture was extracted by CH_3COOEt three times and the organic phase was concentrated under reduced pressure followed by column chromatography purification on silica gel (petroleum ether : ethyl acetate = 10 :1) to give a colorless oil **1b** (0.50 g, 81%, two steps). ^{1}H NMR (600 MHz, $CDCl_3$) δ 7.36-7.26 (m, 13H), 7.22-7.20 (m, 2H), 6.87 (d, J = 2.2 Hz, 1H), 4.61 (d, J = 2.1 Hz, 2H), 4.62 (d, J = 11.9 Hz, 1H), 4.60 (d, J = 11.9 Hz, 1H), 4.52-4.43 (m, 5H), 4.12 (dd, J = 3.5, 1.6 Hz, 1H), 3.89 (t, J = 3.6 Hz, 1H), 3.79-3.71 (m, 2H), 3.66-3.58 (m, 2H), 3.56-3.52 (m, 2H), 1.16 (t, J = 7.0 Hz, 3H); ^{19}F NMR (376 MHz, $CDCl_3$) δ -102.11 (d, J = 253 Hz, 1F), -103.76 (d, J = 253 Hz, 1F). ^{13}C NMR (151 MHz, $CDCl_3$) δ 146.34 (t, J_{CF} = 146.3 Hz), 137.87, 137.77, 137.63, 128.55, 128.41, 128.00, 127.99, 127.87, 127.84, 127.73, 127.72, 120.77 (dd, J_{CF} = 245.1, 241.7 Hz), 106.67 (dd, J_{CF} = 26.4, 23.2 Hz), 75.94, 73.35, 72.37 (dd, J_{CF} = 32.9, 29.8 Hz), 72.11, 72.08, 71.72, 70.07, 67.76, 67.69, 15.03. HRMS (ESI) m/z $[M + Na]^+$ calculated for $C_{31}H_{34}NaO_5F_2$ 547.2266, found 547.2253.

The synthesis of compound 1c

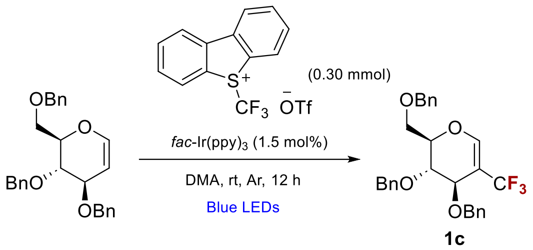

To an oven-dried 10 mL round-bottom flask with a Teflon-coated magnetic stir bar were added glucal (41.6 mg, 0.10 mmol), *fac*-Ir(ppy)$_3$ (1.5 mol%) and Umemoto agent (131.0 mg, 0.30 mmol). The flask was evacuated and sealed, and refilled with Ar for three times. Then dried DMA (2 mL) was injected to the flask dissolving the above reactants. The reaction mixture was stirred under irradiation with blue LEDs (12 W×4, 450-470 nm) at ambient temperature. After reaction completion monitored by TLC about 12 h, the reaction mixture was concentrated under reduced pressure followed by column chromatography purification on silica gel (petroleum ether : ethyl acetate = 20 :1) to give **1c** (40.7 mg, 84%)

in a colorless oil. ^{1}H NMR (400 MHz, $CDCl_3$) δ 7.29-7.11 (m, 15H), 6.99 (d, *J* = 1.3 Hz, 1H), 4.49 (d, *J* = 5.9 Hz, 2H), 4.44-4.31 (m, 5H), 4.01 (m, 1H), 3.80 (t, *J* = 3.2 Hz, 1H), 3.71 (dd, *J* = 10.3, 6.8 Hz, 1H), 3.59 (dd, *J* = 10.5, 5.1 Hz, 1H); ^{19}F NMR (376 MHz, $CDCl_3$) δ -62.53. The ^{1}H NMR and ^{9}F NMR data are in accordance with those reported previously.[2]

The synthesis of compound 1d

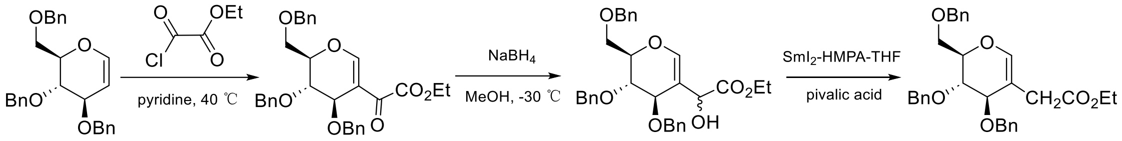

To an oven-dried 50 mL round-bottom flask with a Teflon-coated magnetic stir bar was added glucal (4.2 g, 0.01 mol). The flask was evacuated and sealed, and refilled with Ar for three times. Then dry pyridine (20 mL) was injected to the flask dissolving the above reactant, followed by the addition of oxalyl chloride (4.2 mL, 0.05 mol). The reaction mixture was stirred at 40 ℃. After reaction completion monitored by TLC about one week, the reaction mixture was concentrated under reduced pressure followed by column chromatography purification on silica gel (petroleum ether : ethyl acetate = 10 :1) to give **1d'** (5.11 g, 99%) in a colorless oil.

1d': ^{1}H NMR (400 MHz, $CDCl_3$) δ 8.08 (s, 1H), 7.49-7.17 (m, 15H), 4.72 (td, *J* = 5.1, 2.6 Hz, 2H), 4.67 (d, *J* = 11.3 Hz, 1H), 4.61-4.44 (m, 7H), 4.35 (q, *J* = 7.1 Hz, 2H), 3.87 (t, *J* = 2.1 Hz, 2H), 3.82 (dd, *J* = 10.7, 7.7 Hz, 1H), 3.67 (dd, *J* = 10.7, 4.9 Hz, 1H), 1.40 (t, *J* = 7.1 Hz, 3H); ^{13}C NMR (101 MHz, $CDCl_3$) δ 184.14, 162.99, 162.53, 138.06, 137.64, 137.21, 128.55, 128.44, 128.35, 128.05, 128.03, 127.82, 127.78, 127.76, 127.74, 113.08, 78.55, 73.39, 72.69, 71.64, 71.20, 68.38, 65.98, 62.15, 14.05. HRMS (ESI) m/z $[M + Na]^+$ calculated for $C_{31}H_{32}NaO_7$ 539.2148, found 539.2137.

To an oven-dried 50 mL round-bottom flask with a Teflon-coated magnetic stir bar was added **1d'** (1.3 g, 2.0 mmol). The flask was evacuated and refilled with Ar for three times. Then dry MeOH (10 mL) was added to the flask dissolving the above reactant. The reaction mixture was stirred at -30 ℃, followed by the addition of $NaBH_4$ (75.6 mg, 2.0 mol). After reaction completion monitored by TLC about 3 h, the reaction mixture was concentrated under reduced pressure followed by column chromatography purification on silica gel (petroleum ether : ethyl acetate = 4 :1) to give **1d"** (1.0 g, 93%) in a colorless oil. **1d"**: ^{1}H NMR (400 MHz, $CDCl_3$) δ 7.53-7.20 (m, 15H), 6.58 (s, 1H), 4.85-4.42 (m, 7H), 4.25 (qd, *J* = 7.1, 1.6 Hz, 4H), 4.01 (dd, *J* = 6.9, 5.2 Hz, 1H), 3.83 (dd, *J* = 10.7, 5.3 Hz, 1H), 3.74 (dd, *J* = 10.7, 3.8 Hz, 1H), 3.65 (d, *J* = 9.4 Hz, 1H), 1.30 (t, *J* = 7.1 Hz, 3H); ^{13}C NMR (101 MHz, $CDCl_3$) δ 172.94, 145.44, 137.79, 137.62, 137.57, 128.55, 128.42, 128.10, 127.99, 127.98, 127.79, 127.75, 127.73, 109.97, 76.54, 75.63, 74.18, 73.50, 73.12, 72.09, 67.77, 61.43, 14.27. HRMS (ESI) m/z $[M + Na]^+$ calculated for $C_{27}H_{28}NaO_4$ 439.5170, found 439.5164.

To an oven-dried 50 mL round-bottom flask with a Teflon-coated magnetic stir bar was added **1d"** (0.52 g, 1.0 mmol). The flask was evacuated and refilled with Ar for three times. Then dry THF (10 mL) was added to the flask dissolving the above reactant, followed by the addition of HMPA (0.9 mL, 5.0 mmol) and pivalic acid (10.1 mg, 1.0 mmol). The

reaction mixture was stirred at room temperature for 10 min, and then SmI_2 (5.0 mL, 0.1 mol/L in THF) was added. After reaction completion monitored by TLC about 5 h, the reaction mixture was concentrated under reduced pressure followed by column chromatography purification on silica gel (petroleum ether : ethyl acetate = 20 :1) to give **1d** (0.44 g, 88%) in a colorless oil. **1d**: ^1H NMR (400 MHz, $CDCl_3$) δ 7.55-7.07 (m, 15H), 6.37 (s, 1H), 4.77 (d, *J* = 11.4 Hz, 1H), 4.71-4.64 (m, 2H), 4.62-4.53 (m, 3H), 4.30 (d, *J* = 5.6 Hz, 1H), 4.19 (td, *J* = 4.8, 2.5 Hz, 1H), 4.09 (q, *J* = 7.0 Hz, 2H), 3.96 (dd, *J* = 7.7, 5.8 Hz, 1H), 3.83 (dd, *J* = 10.7, 5.2 Hz, 1H), 3.75 (dd, *J* = 10.7, 3.3 Hz, 1H), 3.17 (d, *J* = 15.9 Hz, 1H), 2.84 (d, *J* = 16.0 Hz, 1H), 1.22 (t, *J* = 7.1 Hz, 3H). HRMS (ESI) m/z $[M + Na]^+$ calculated for $C_{31}H_{34}NaO_6$ 525.6070, found 525.6069.

The synthesis of compound 1f

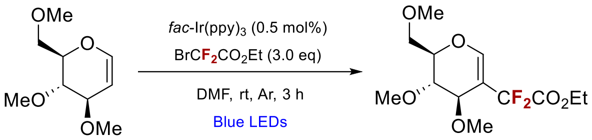

1f

According to the *General photoredox difluoroalkylation procedure*, reaction mixture was purified by column chromatography on silica gel (petroleum ether : ethyl acetate = 15 :1) to give **1f** as a colorless oil (28.2 mg, 91%). ^1H NMR (400 MHz, $CDCl_3$) δ 6.90 (d, *J* = 2.9 Hz, 1H), 4.37-4.23 (m, 3H), 3.83 (d, *J* = 4.2 Hz, 1H), 3.68 (dd, *J* = 10.6, 6.4 Hz, 1H), 3.59-3.54 (m, 2H), 3.49 (s, 3H), 3.40 (s, 3H), 3.40 (s, 3H), 1.33 (t, *J* = 7.1 Hz, 3H); ^{19}F NMR (376 MHz, $CDCl_3$) δ -102.46 (d, *J* = 256.1 Hz, 1F), -109.64 (d, *J* = 256.2 Hz, 1F). The ^1H NMR and ^{19}F NMR data are in accordance with those reported previously.[1]

The synthesis of compound 1g

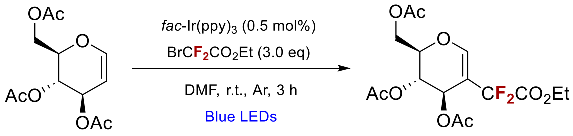

1g

According to the *General photoredox difluoroalkylation procedure*, reaction mixture was purified by column chromatography on silica gel (petroleum ether : ethyl acetate = 8 :1) to give **1g** as a colorless oil (33.1 mg, 84%). ^1H NMR (400 MHz, $CDCl_3$) δ 8.13 (s, 1H), 6.63 (d, *J* = 3.1 Hz, 1H), 5.71 (dd, *J* = 11.7, 9.3 Hz, 1H), 5.09 (t, *J* = 9.8 Hz, 1H), 4.44-4.27 (m, 3H), 4.17-4.10 (m, 1H), 4.08 (dd, *J* = 12.4, 2.1 Hz, 1H), 3.22 (ddt, *J* = 19.2, 11.7, 3.7 Hz, 1H), 2.11 (s, 3H), 2.05 (s, 3H), 1.98 (s, 3H), 1.41 (t, *J* = 7.1 Hz, 3H); ^{19}F NMR (376 MHz, $CDCl^3$) δ -109.09 (d, *J* = 271 Hz, 1F), -112.49 (d, *J* = 271 Hz, 1F). The ^1H NMR and ^{19}F NMR data are in accordance with those reported previously.[1]

The synthesis of compound 1h

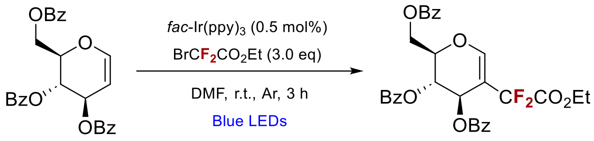

1h

According to the *General photoredox difluoroalkylation procedure*, reaction mixture was purified by column chromatography on silica gel (petroleum ether : ethyl acetate = 5 :1) to give **1h** as a colorless oil (44.7 mg, 77%). ^{1}H NMR (400 MHz, $CDCl_3$) δ 8.14 (dd, *J* = 8.4, 1.3 Hz, 1H), 8.05 (dd, *J* = 8.3, 1.2 Hz, 2H), 7.99 (dd, *J* = 8.3, 1.2 Hz, 2H), 7.94 (dd, *J* = 8.4, 1.3 Hz, 2H), 7.67-7.36 (m, 8H), 7.33 (s, 1H), 6.01-5.94 (m, 1H), 5.73 (t, *J* = 3.0 Hz, 1H), 5.01-4.91 (m, 1H), 4.87 (dd, *J* = 11.7, 7.9 Hz, 1H), 4.56 (dd, *J* = 11.7, 5.2 Hz, 1H), 4.27-4.08 (m, 1H), 1.21 (t, *J* = 7.2 Hz, 3H); ^{19}F NMR (376 MHz, $CDCl_3$) δ -103.45 (d, *J* = 263 Hz, 1F), -104.89 (d, *J* = 263 Hz, 1F). The ^{1}H NMR and ^{19}F NMR data are in accordance with those reported previously.[1]

The synthesis of compound 1i

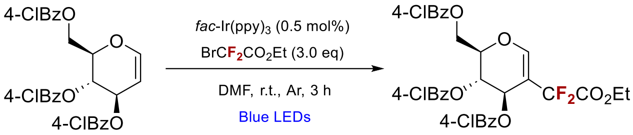

1i

According to the *General photoredox difluoroalkylation procedure*, reaction mixture was purified by column chromatography on silica gel (petroleum ether : ethyl acetate = 15 :1) to give **1i** as a colorless oil (51.2 mg, 75%). ^{1}H NMR (400 MHz, $CDCl_3$) δ 7.96-7.93 (m, 2H), 7.87-7.81 (m, 4H), 7.45-7.42 (m, 2H), 7.38-7.34 (m, 4H), 7.29 (d, *J* = 1.7 Hz, 1H), 5.93 (d, *J* = 3.3 Hz, 1H), 5.67 (t, *J* = 3.3 Hz, 1H), 4.92-4.88 (m, 1H), 4.76 (dd, *J* = 11.8, 7.6 Hz, 1H), 4.51 (dd, *J* = 11.8, 5.6 Hz, 1H), 4.26-4.18 (m, 1H), 4.17-4.09 (m, 1H), 1.22 (t, *J* = 7.1 Hz, 3H); ^{19}F NMR (376 MHz, $CDCl_3$) δ -103.43 (d, *J* = 263 Hz, 1F), -104.32 (d, *J* = 263 Hz, 1F); ^{13}C NMR (101 MHz, $CDCl_3$) δ 164.94, 164.02, 163.96, 163.39 (t, J_{CF} = 35.3 Hz), 149.21 (t, J_{CF} = 10.3 Hz), 140.44, 140.30, 139.96, 131.28, 131.05, 130.96, 128.99, 128.95, 128.79, 127.43, 127.17, 127.11, 112.70 (t, J_{CF} = 252.2 Hz), 103.48 (t, J_{CF} = 25.0 Hz), 73.66, 66.26, 63.38, 62.44, 61.54, 26.92, 13.78. HRMS (ESI) *m/z* [M + Na]$^+$ calculated for $C_{31}H_{23}Cl_3F_2O_9Na$ 705.0376, found 705.0375.

The synthesis of compound 1j

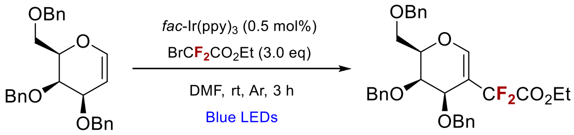

1j

According to the *General photoredox difluoroalkylation procedure*, reaction mixture was purified by column chromatography on silica gel (petroleum ether : ethyl acetate = 20 :1) to give **1j** as a colorless oil (48.0 mg, 89%). ^{1}H NMR (400 MHz, $CDCl_3$) δ 7.40-7.28 (m,

15H), 6.89 (d, J = 3.0 Hz, 1H), 4.80-4.76 (m, 2H), 4.65-4.46 (m, 4H), 4.42 (dt, J = 7.7, 3.7 Hz, 1H), 4.34 (d, J = 3.6 Hz, 1H), 4.04 (q, J = 7.1 Hz, 2H), 3.99 (t, J = 3.6 Hz, 1H), 3.90 (dd, J = 10.9, 7.8, 1H), 3.80 (dd, J = 10.9, 3.8, 1H), 1.10 (t, J = 7.1 Hz, 3H); ^{19}F NMR (376 MHz, $CDCl_3$) δ -101.44 (d, J = 258.2 Hz, 1F), -109.09 (d, J = 258.7 Hz, 1F). The ^{1}H NMR and ^{19}F NMR data are in accordance with those reported previously.[1]

The synthesis of compound 1k

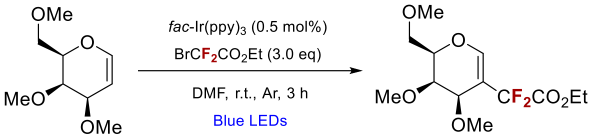

1k

According to the *General photoredox difluoroalkylation procedure*, reaction mixture was purified by column chromatography on silica gel (petroleum ether : ethyl acetate = 16 :1) to give **1k** as a colorless oil (27.0 mg, 87%). ^{1}H NMR (400 MHz, $CDCl_3$) δ 6.85 (d, J = 2.3 Hz, 1H), 4.42-4.20 (m, 3H), 4.06 (s, 1H), 3.79-3.73 (m, 2H), 3.65 (dd, J = 10.8, 3.9 Hz, 1H), 3.56 (s, 3H), 3.43 (s, 3H), 3.42 (s, 3H), 1.35 (t, J = 7.1 Hz, 3H); ^{19}F NMR (376 MHz, $CDCl_3$) δ -101.75 (d, J = 256 Hz, 1F), -108.93 (d, J = 259 Hz, 1F). The ^{1}H NMR and ^{19}F NMR data are in accordance with those reported previously.[1]

The synthesis of compound 1l

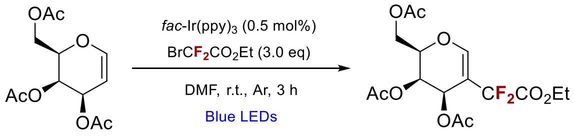

1l

According to the *General photoredox difluoroalkylation procedure*, reaction mixture was purified by column chromatography on silica gel (petroleum ether : ethyl acetate = 8 :1) to give **1l** as a colorless oil (33.9 mg, 86%). ^{1}H NMR (400 MHz, $CDCl_3$) δ 7.01 (s, 1H), 5.79 (d, J = 4.4 Hz, 1H), 5.44 (dd, J = 4.4, 2.6 Hz, 1H), 4.44-4.40 (m, 1H), 4.37-4.23 (m, 4H), 2.12-2.10 (m, 6H), 2.00 (s, 3H), 1.35 (t, J = 7.1 Hz, 3H); ^{19}F NMR (376 MHz, $CDCl^3$) δ -103.8 (d, J = 263 Hz, 1F), -104.7 (d, J =263 Hz, 1F). The ^{1}H NMR and ^{19}F NMR data are in accordance with those reported previously.[1]

The synthesis of compound 1m

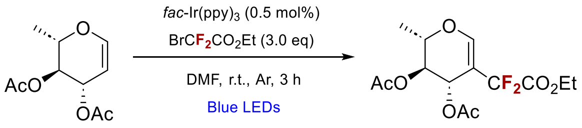

1m

According to the *General photoredox difluoroalkylation procedure*, reaction mixture was purified by column chromatography on silica gel (petroleum ether : ethyl acetate = 12 :1) to give **1m** as a colorless oil (27.9 mg, 83%). ^{1}H NMR (400 MHz, $CDCl_3$) δ 7.04 (s, 1H), 5.59 (d, J = 4.0 Hz, 1H), 4.97 (t, J = 4.6 Hz, 1H), 4.37-4.34 (m, 1H), 4.34-4.29 (m, 2H), ,2.08 (s, 3H), 2.02 (s, 3H), 1.39-1.37 (m, 3H), 1.35 (d, J = 7.2 Hz, 3H); ^{19}F NMR (376 MHz, $CDCl_3$) δ -103.35 (d, J = 261 Hz, 1F), -104.85 (d, J = 261 Hz, 1F). The ^{1}H NMR and ^{19}F NMR data are in accordance with those reported previously.[1]

The synthesis of compound 1n

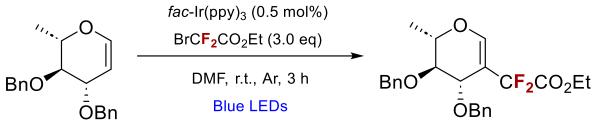

1n

According to the *General photoredox difluoroalkylation procedure*, reaction mixture was purified by column chromatography on silica gel (petroleum ether : ethyl acetate = 24 :1) to give **1n** as a colorless oil (38.9 mg, 90%). ^{1}H NMR (400 MHz, $CDCl_3$) δ 7.52-7.18 (m, 10H), 6.95 (d, *J* = 2.6 Hz, 2H), 4.68-4.56 (m, 4H), 4.41-4.28 (m, 1H), 4.18 (d, *J* = 3.6 Hz, 1H), 4.16-3.99 (m, 2H), 3.59 (s, 1H), 1.41 (d, *J* = 6.9 Hz, 3H), 1.17 (t, *J* = 7.1 Hz, 3H); ^{19}F NMR (376 MHz, $CDCl_3$) δ -102.58 (d, *J* = 256 Hz, 1F), -108.89 (d, *J* = 256 Hz, 1F). The ^{1}H NMR and ^{19}F NMR data are in accordance with those reported previously.[1]

The synthesis of compound 1o

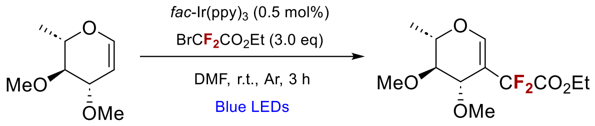

1o

According to the *General photoredox difluoroalkylation procedure*, reaction mixture was purified by column chromatography on silica gel (petroleum ether : ethyl acetate = 18 :1) to give **1o** as a colorless oil (25.6 mg, 91%). ^{1}H NMR (400 MHz, $CDCl_3$) δ 6.87 (d, *J* = 1.8 Hz, 1H), 4.44-4.16 (m, 3H), 3.85 (d, *J* = 3.4 Hz, 1H), 3.53 (s, 3H), 3.42 (s, 3H), 3.30 (t, *J* = 5.0 Hz, 1H), 1.45-1.29 (m, 6H); ^{19}F NMR (376 MHz, $CDCl_3$) δ -102.37 (d, *J* = 259 Hz, 1F), -109.96 (d, *J* = 256 Hz, 1F). The ^{1}H NMR and ^{19}F NMR data are in accordance with those reported previously.[1]

The synthesis of compound 1p

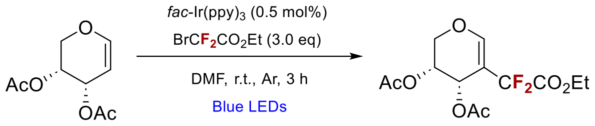

1p

According to the *General photoredox difluoroalkylation procedure*, reaction mixture was purified by column chromatography on silica gel (petroleum ether : ethyl acetate = 15 :1) to give **1p** as a colorless oil (30.0 mg, 93%). ^{1}H NMR (400 MHz, $CDCl_3$) δ 7.07 (s, 1H), 5.82 (d, *J* = 3.8 Hz, 1H), 5.20-5.15 (m, 1H), 4.40-4.25 (m, 2H), 4.13-4.09 (m, 1H), 4.01 (t, *J* = 10.8 Hz, 1H), 2.07 (s, 3H), 2.04 (s, 3H), 1.36 (t, *J* = 7.1 Hz, 3H); ^{19}F NMR (376 MHz, $CDCl_3$) δ -102.8 (d, *J* = 260 Hz, 1F), -104.4 (d, *J* = 260 Hz, 1F). HRMS (ESI) m/z $[M + NH_4]^+$ calculated for $C_{13}H_{20}NF_2O_7$ 340.1202, found 340.1198. The ^{1}H NMR and ^{19}F NMR data are in accordance with those reported previously.[1]

The synthesis of compound 1q

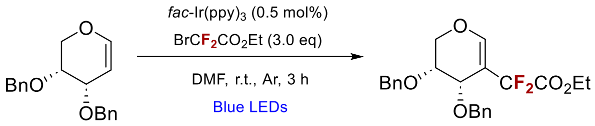

1q

According to the *General photoredox difluoroalkylation procedure*, reaction mixture was

purified by column chromatography on silica gel (petroleum ether : ethyl acetate = 15 :1) to give **1q** as a colorless oil (35.9 mg, 86%). ^{1}H NMR (400 MHz, $CDCl_3$) δ 7.44-7.26 (m, 10H), 6.91 (d, *J* = 2.4 Hz, 1H), 4.98 (d, *J* = 10.5 Hz, 1H), 4.77-4.58 (m, 3H), 4.38-4.32 (m, 1H), 4.19-4.08 (m, 4H), 3.86-3.77 (m, 1H), 1.22 (t, *J* = 7.1 Hz, 3H); ^{19}F NMR (376 MHz, $CDCl_3$) δ -100.50 (d, *J* = 252 Hz, 1F), -107.58 (d, *J* = 252 Hz, 1F). The ^{1}H NMR and ^{19}F NMR data are in accordance with those reported previously.[1]

The synthesis of compound 1r

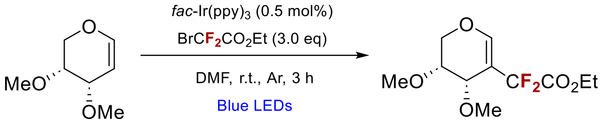

1r

According to the *General photoredox difluoroalkylation procedure*, reaction mixture was purified by column chromatography on silica gel (petroleum ether : ethyl acetate = 18 :1) to give **1r** as a colorless oil (25.6 mg, 91%). ^{1}H NMR (400 MHz, $CDCl_3$) δ 6.88 (d, *J* = 2.7 Hz, 1H), 4.38-4.27 (m, 2H), 4.07 (ddd, *J* = 10.2, 4.4, 1.6 Hz, 1H), 4.03 (dd, *J* = 3.3, 1.5 Hz, 1H), 3.95 (t, *J* = 10.7 Hz, 1H), 3.55 (ddd, *J* = 11.3, 4.3, 3.2 Hz, 1H), 3.49 (s, 3H), 3.49 (s, 3H), 1.35 (t, *J* = 7.1 Hz, 3H); ^{19}F NMR (376 MHz, $CDCl_3$) δ -100.83 (d, *J* = 252 Hz, 1F), -107.58 (d, *J* = 248 Hz, 1F). The ^{1}H NMR and ^{19}F NMR data are in accordance with those reported previously.[1]

The synthesis of compound 1s

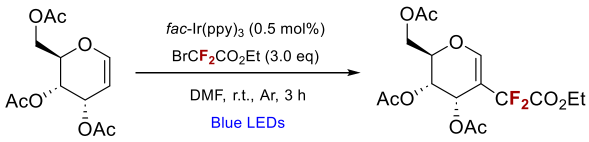

1s

According to the *General photoredox difluoroalkylation procedure*, reaction mixture was purified by column chromatography on silica gel (petroleum ether : ethyl acetate = 5 :1) to give **1s** as a colorless oil (31.9 mg, 81%). ^{1}H NMR (400 MHz, $CDCl_3$) δ 7.09 (s, 1H), 5.86 (d, *J* = 3.5 Hz, 1H), 5.16-5.11 (m, 1H), 4.38-4.28 (m, 5H), 2.09 (s, 3H), 2.08 (s, 3H), 2.02 (s, 3H), 1.37 (t, *J* = 7.1 Hz, 3H); ^{19}F NMR (376 MHz, $CDCl_3$) δ -102.8 (d, *J* = 259 Hz, 1F), -104.6 (d, *J* = 259 Hz, 1F). ^{13}C NMR (101 MHz, $CDCl_3$) δ 170.5, 169.7, 169.1, 163.3 (t, J_{CF} = 34.3 Hz), 150.5 (t, J_{CF} = 10.1 Hz), 104.3 (t, J_{CF} = 25.5 Hz), 71.0, 65.2, 63.3, 61.2, 60.2, 60.2, 20.7, 20.6, 20.5, 13.9. HRMS (ESI) m/z $[M + NH_4]^+$ calculated for $C_{16}H_{24}NF_2O_9$ 412.1413, found 412.1403.

The synthesis of compound 1t

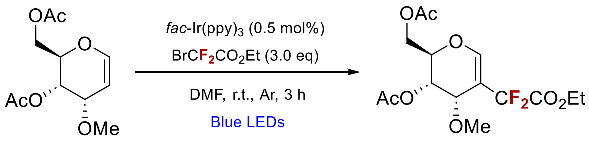

1t

According to the *General photoredox difluoroalkylation procedure*, reaction mixture was purified by column chromatography on silica gel (petroleum ether : ethyl acetate = 5 :1) to give **1t** as a colorless oil (32.2 mg, 88%). ^{1}H NMR (400 MHz, $CDCl_3$) δ 6.92 (dd, *J* = 2.5,

1.3 Hz, 1H), 5,03 (dd, J = 10.3, 3.4 Hz, 1H), 4.41-4.28 (m, 5H), 4.08 (d, J = 3.3 Hz, 1H), 3.42 (s, 1H), 2.13 (s, 3H), 2.07 (s, 3H), 1.35 (t, J = 7.2 Hz, 3H); ^{19}F NMR (376 MHz, $CDCl_3$) δ -102.1 (d, J = 253 Hz, 1F), -107.4 (d, J = 254 Hz, 1F). ^{13}C NMR (101 MHz, $CDCl_3$) δ 170.5, 169.6, 163.8 (dd, J_{CF} = 37.3, 34.0 Hz), 147.9 (t, J_{CF} = 11.7, 9.2 Hz), 112.7 (t, J_{CF} = 251.5 Hz), 107.0 (dd, J_{CF} = 26.8, 24.0 Hz), 70.9, 69.0, 68.9, 68.2, 63.1, 61.5, 60.3, 20.8, 20.7, 13.9. HRMS (ESI) m/z [M + H]$^+$ calculated for $C_{15}H_{21}F_2O_8$ 367.1199, found 367.1193.

The synthesis of compound 1u

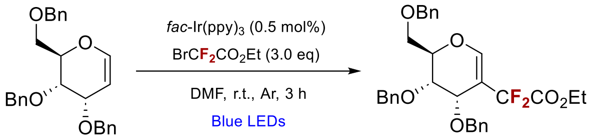

1u

According to the *General photoredox difluoroalkylation procedure*, reaction mixture was purified by column chromatography on silica gel (petroleum ether : ethyl acetate = 5 :1) to give **1u** as a colorless oil (47.4 mg, 88%). ^{1}H NMR (400 MHz, $CDCl_3$) δ 7.35-7.23 (m, 15H), 6.93 (d, J = 2.2 Hz, 1H), 4.88 (d, J = 10.5 Hz, 1H), 4.74 (d, J = 11.4 Hz, 1H), 4.64-4.53 (m, 4H), 4.39 (dt, J = 10.7, 2.8 Hz, 1H), 4.32 (d, J = 3.0 Hz, 1H), 4.18-4.04 (m, 2H), 3.90 (dd, J = 10.8, 3.0 Hz, 1H), 3.85-3.82 (m, 2H), 1.20 (t, J = 7.1 Hz, 3H); ^{19}F NMR (376 MHz, $CDCl_3$) δ -100.5 (d, J = 251 Hz, 1F), -106.9 (d, J = 251 Hz, 1F). ^{13}C NMR (101 MHz, $CDCl_3$) δ 164.0 (t, J_{CF} = 38.4 Hz), 148.7 (t, J_{CF} = 9.1 Hz), 138.5, 137.8, 137.5, 128.5, 128.4, 128.1, 128.0, 127.8, 127.7, 127.5, 113.2 (t, J_{CF} = 251.5 Hz), 106.2 (dd, J_{CF} = 26.9, 23.6 Hz), 74.6, 74.1, 73.6, 73.5, 72.4, 68.2, 66.8, 66.7, 62.9, 13.8. HRMS (ESI) m/z [M + NH_4]$^+$ calculated for $C_{31}H_{36}NF_2O_6$ 556.2505, found 556.2491.

The synthesis of compound 1v

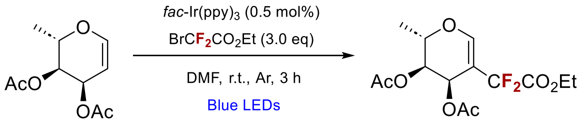

1v

According to the *General photoredox difluoroalkylation procedure*, reaction mixture was purified by column chromatography on silica gel (petroleum ether : ethyl acetate = 6 :1) to give **1v** as a colorless oil (51.0 mg, 51%). ^{1}H NMR (400 MHz, $CDCl_3$) δ 7.05 (s, 1H), 5.80 (d, J = 3.6 Hz, 1H), 4.86 (dd, J = 10.8, 3.6 Hz, 1H), 4.37-4.31 (m, 1H), 4.31-4.21 (m, 2H), 2.07 (s, 3H), 2.04 (s, 3H), 1.37 (d, J = 7.1 Hz, 3H), 1.34-1.32 (m, 3H); ^{13}C NMR (101 MHz, $CDCl_3$) δ 169.85, 169.48, 150.88, 77.34, 77.02, 76.70, 70.12, 69.57, 63.22, 60.62, 29.71, 20.69 (d, J = 16.8 Hz), 16.77, 13.89;^{19}F NMR (376 MHz, $CDCl_3$) δ -102.62 (d, J = 258.8 Hz, 1F), -104.18 (d, J = 258.8 Hz, 1F). HRMS (ESI) Calculated for $C_{14}H_{22}O_7NF_2$ [M+NH_4]$^+$: 354.1358 Found: 354.1351.

4.2 Glycosylation scope

The synthesis of compound 2a

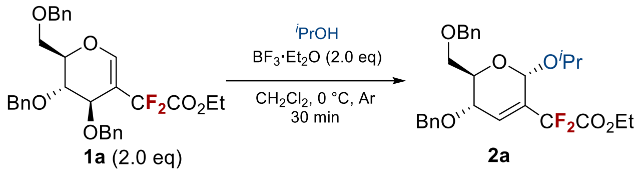

According to the *General glycosylation procedure A*, reaction mixture was purified by column chromatography on silica gel (petroleum ether : ethyl acetate = 12 : 1) to give **2a** as a colorless oil (42.6 mg, 87%). ^{1}H NMR (400 MHz, $CDCl_3$) δ 7.38-7.27 (m, 10H), 6.56 (t, *J* = 2.3 Hz, 1H), 5.27 (s, 1H), 4.69-4.65 (m, 2H), 4.54-4.47 (m, 2H), 4.34-4.27 (m, 3H), 4.04-3.94 (m, 2H), 3.79-3.65 (m, 2H), 1.35 (t, *J* = 7.1 Hz, 3H), 1.20 (d, *J* = 6.2 Hz, 3H), 1.14 (d, *J* = 6.1 Hz, 3H); ^{19}F NMR (376 MHz, $CDCl_3$) δ -107.19 (d, *J* = 258.9 Hz, 1F), -109.43 (d, *J* = 259.1 Hz, 1F); ^{13}C NMR (101 MHz, $CDCl_3$) δ163.0 (dd, J_{CF} = 36.5, 32.4 Hz), 138.0, 137.6, 131.7 (t, J_{CF} = 8.1 Hz), 131.33 (dd, J_{CF} = 24.9, 22.5 Hz), 128.5, 128.4, 128.0, 127.8, 127.7, 111.9 (t, J_{CF} = 251.49 Hz), 91.5 (d, J_{CF} = 5.2 Hz), 73.4, 71.4, 71.2, 69.9, 68.5, 68.4, 62.8, 23.6, 21.3, 13.9. HRMS (ESI) m/z [M + Na]$^+$ calculated for $C_{27}H_{32}NaF_2O_6$ 513.2065, found 513.2070.

The synthesis of compound 2b

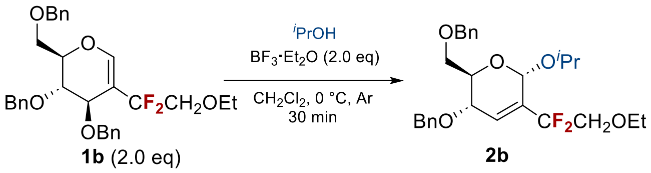

According to the *General glycosylation procedure A*, reaction mixture was purified by column chromatography on silica gel (petroleum ether : ethyl acetate = 15 : 1) to give **2b** as a colorless oil (31.0 mg, 65%). ^{1}H NMR (600 MHz, $CDCl_3$) δ 7.33-7.26 (m, 8H), 7.24-7.23 (m, 2H), 6.36 (t, *J* = 2.2 Hz, 1H), 5.26 (s, 1H), 4.65-4.62 (m, 4H), 4.49 (d, *J* = 12.1 Hz, 1H), 4.43 (d, *J* = 11.3 Hz, 1H), 4.24-4.20 (m, 1H), 4.03-3.96 (m, 2H), 3.84 (ddd, *J* = 20.4, 12.1, 6.1 Hz, 1H), 3.73 (dd, *J* = 10.7, 4.1 Hz, 1H), 3.69 (dd, *J* = 10.7, 2.2 Hz, 1H), 3.66-3.59 (m, 3H), 1.21-1.19 (m, 6H), 1.16 (t, *J* = 6.1 Hz, 3H); ^{19}F NMR (376 MHz, $CDCl_3$) δ -104.96 (d, *J* = 250 Hz, 1F), -110.43 (d, *J* = 252 Hz, 1F); ^{13}C NMR (151 MHz, $CDCl_3$) δ 138.11, 137.69, 133.26 (dd, J_{CF} = 26.0, 22.6 Hz), 131.00 (t, J_{CF} = 7.5 Hz), 128.45, 128.36, 127.95, 127.93, 127.81, 127.65, 119.42 (dd, J_{CF} = 248.3, 240.9 Hz), 91.94 (t, J_{CF} = 4.6 Hz), 73.40, 71.77 (dd, J_{CF} = 31.5, 26.4 Hz), 71.39, 70.91, 70.01, 68.60, 68.46, 67.98, 23.63, 21.77, 15.14. HRMS (ESI) m/z [M + Na]$^+$ calculated for $C_{27}H_{34}NaF_2O_5$ 499.2266, found 499.2264.

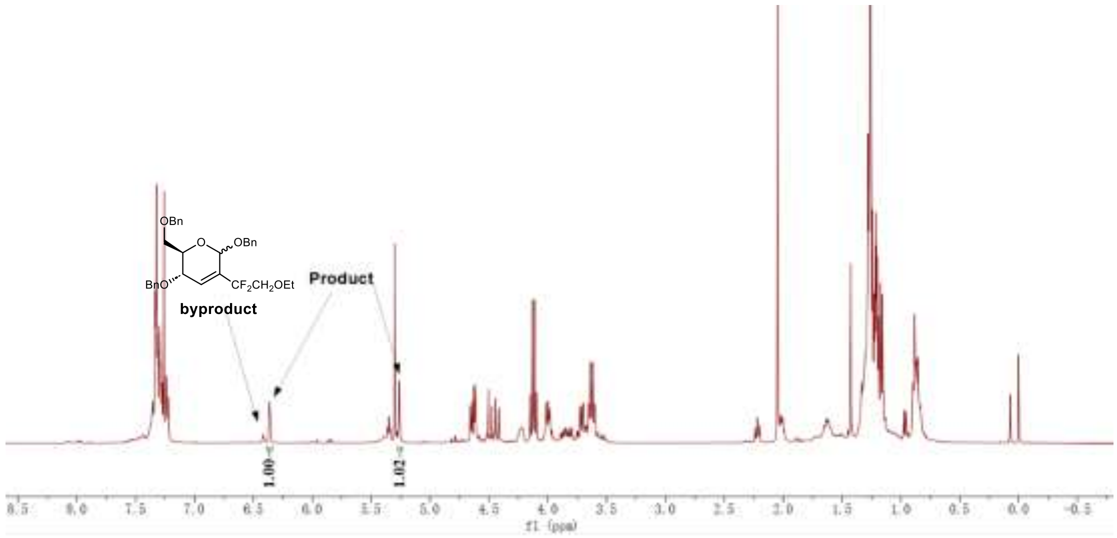

Crude product analysis of 2b

The synthesis of compound 2c

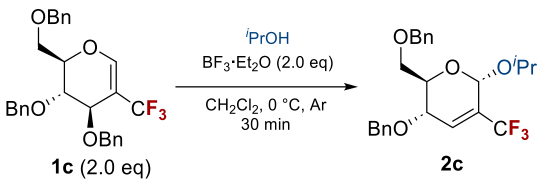

According to the *General glycosylation procedure A*, reaction mixture was purified by column chromatography on silica gel (petroleum ether : ethyl acetate = 8 : 1) to give **2c** as a colorless oil (35.8 mg, 82%). ^{1}H NMR (400 MHz, $CDCl_3$) δ 7.39-7.24 (m, 10H), 6.58 (s, 1H), 5.29 (s, 1H), 4.66 (dd, J = 11.7, 9.7 Hz, 2H), 4.49 (dd, J = 17.0, 11.8 Hz, 2H), 4.29 (d, J = 9.4 Hz, 1H), 4.10 (dt, J = 9.6, 2.7 Hz, 1H), 4.02 (p, J = 6.2 Hz, 1H), 3.78 (dd, J = 10.8, 3.6 Hz, 1H), 3.71 (dd, J = 10.7, 1.9 Hz, 1H), 1.25 (d, J = 6.2 Hz, 3H), 1.19 (d, J = 6.1 Hz, 3H); ^{19}F NMR (400 MHz, $CDCl_3$) δ -65.89; ^{13}C NMR (400 MHz, $CDCl_3$) δ 137.96, 137.44, 133.29 (d, J_{CF} = 5.1 Hz), 129.74 (d, J_{CF} = 30.8 Hz), 128.47, 128.37, 128.01, 127.90, 127.85, 127.72, 91.33 (d, J_{CF} = 1.7 Hz), 73.44, 71.67, 71.55, 69.47, 68.50, 68.32, 23.48, 21.65. HRMS (ESI) m/z [M + Na]$^+$ calculated for $C_{24}H_{27}NaF_3O_4$ 459.1861, found 459.1862.

The synthesis of compound 2d

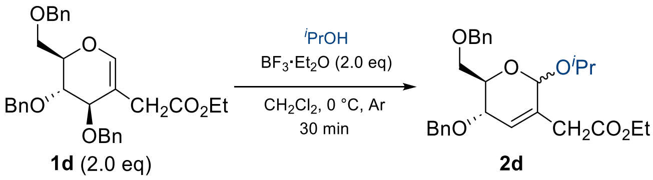

According to the *General glycosylation procedure A*, reaction mixture was purified by column chromatography on silica gel (petroleum ether : ethyl acetate = 7 : 1) to give **2d** as a colorless oil (40.0 mg, 79%, α/β = 2:1). ^{1}H NMR (400 MHz, $CDCl_3$) δ 7.48-6.97 (m, 10H), 5.96 (s, 0.66H, α-H3), 5.95 (s, 0.28 H, β-H3), 5.18 (s, 1H), 4.83 (d, J = 11.8 Hz, 0.7H), 4.70-4.50 (m, 4.3H), 4.45 (dd, J = 11.5, 4.0 Hz, 1H), 4.31-3.90 (m, 5.2H), 3.82-3.56 (m, 2.4H), 3.15 (ddt, J = 13.2, 11.6, 1.5 Hz, 1H), 3.09-2.99 (m, 1H), 1.33-1.08 (m, 6H). HRMS

(ESI) m/z [M + Na]+ calculated for $C_{27}H_{34}NaO_6$ 477.2355, found 477.2354.

The synthesis of compound 2e

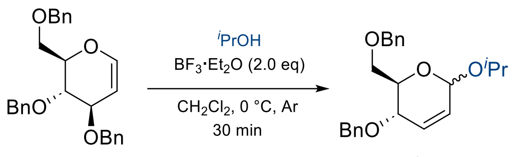

According to the *General glycosylation procedure A*, reaction mixture was purified by column chromatography on silica gel (petroleum ether : ethyl acetate = 8 : 1) to give **2e** as a colorless oil (27.6 mg, 72%, α/β = 3:1). ^{1}H NMR (400 MHz, $CDCl_3$) δ 7.38-7.25 (m, 18H), 6.09 (d, *J* = 10.3 Hz, 2.92H, α-H3), 6.05 (dt, *J* = 10.4, 2.1 Hz, 1H, β-H3), 5.82 (d, *J* = 10.3 Hz, 1H, β-H2), 5.77 (dt, *J* = 10.2, 2.2 Hz, 2.92H, α-H2), 5.22 (s, 1H, β-H1), 5.15 (s, 2.92H, α-H1), 4.72-4.44 (m, 16H), 4.23-3.96 (m, 12H), 3.82-3.68 (m, 8H), 1.26 (t, *J* = 5.8 Hz, 12H), 1.20 (t, *J* = 5.4 Hz, 12H); ^{13}C NMR (400 MHz, $CDCl_3$) δ 138.39, 138.27, 138.19, 138.05, 130.33, 129.79, 128.41, 128.36, 128.35, 128.31, 127.97, 127.81, 127.80, 127.78, 127.70, 127.66, 127.57, 127.23, 94.23, 92.84, 75.36, 73.37, 73.34, 71.05, 70.96, 70.45, 70.03, 69.94, 69.80, 68.98, 29.72, 23.67, 23.64, 21.91, 21.89. The NMR data are in accordance with those reported previously.[3]

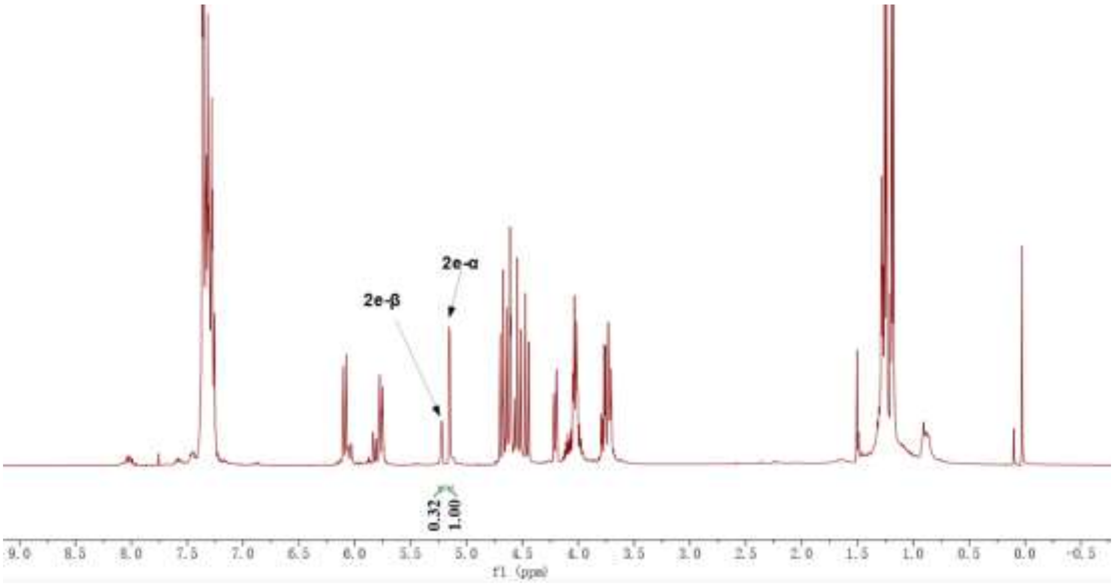

Crude product analysis of 2e

The synthesis of compound 3a

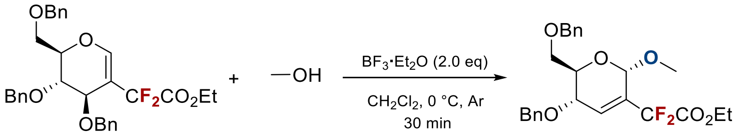

According to the *General glycosylation procedure A*, reaction mixture was purified by column chromatography on silica gel (petroleum ether : ethyl acetate = 10 : 1) to give **3a** as a colorless oil (42.0 mg, 91%). ^{1}H NMR (400 MHz, CDCl3) δ 7.36-7.23 (m, 10H), 6.55 (dd, *J* = 3.3, 1.8 Hz, 1H), 5.01 (s, 1H), 4.64 (dd, *J* = 11.8, 2.8 Hz, 1H), 4.52-4.44 (m, 2H), 4.33-4.23 (m, 3H), 3.94 (dt, *J* = 9.6, 3.1 Hz, 1H), 3.76-3.69 (m, 2H), 3.39 (s, 3H), 1.32 (t, *J*

= 7.1 Hz, 3H); ^{19}F NMR (376 MHz, CDCl3) δ -105.41 (d, *J* = 257.3 Hz, 1F), -110.78 (d, *J* = 257.4 Hz, 1F); ^{13}C NMR (101 MHz, $CDCl_3$) δ 163.1 (dd, J_{CF} = 35.9, 32.2 Hz), 138.0, 137.5, 132.1 (dd, J_{CF} = 8.5, 6.9 Hz), 131.0 (dd, J_{CF} = 24.8, 22.8 Hz), 128.5, 128.4, 128.0, 128.0, 127.9, 127.7, 111.8 (dd, J_{CF} = 252.7, 249.1 Hz), 92.1 (dd, J_{CF} = 5.4, 1.9 Hz), 73.5, 71.5, 69.8, 68.7, 68.3, 63.0, 56.2, 13.9. HRMS (ESI) *m/z* $[M + Na]^+$ calculated for $C_{25}H_{28}NaF_2O_6$ 485.1752, found 485.1758.

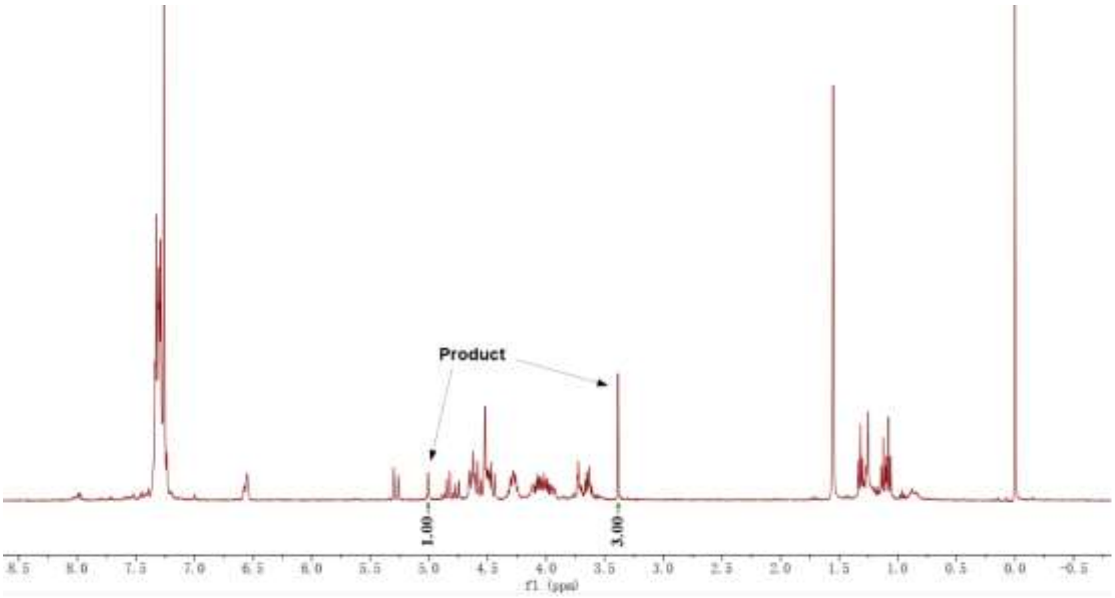

Crude product analysis of 3a

The synthesis of compound 3b

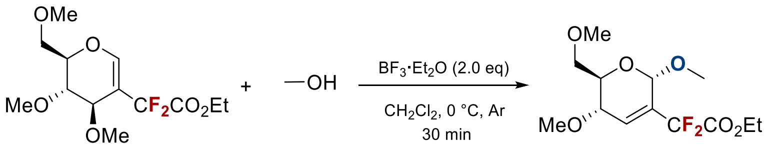

1b (2.0 eq) **3b**

According to the *General glycosylation procedure A*, reaction mixture was purified by column chromatography on silica gel (petroleum ether : ethyl acetate = 8 : 1) to give **3b** as a colorless oil (29.0 mg, 87%). ^{1}H NMR (400 MHz, $CDCl_3$) δ 6.57 (dd, *J* = 3.4, 1.8 Hz, 1H), 5.00 (s, 1H), 4.37-4.25 (m, 2H), 4.02-3.97 (m, 1H), 3.85 (dt, *J* = 9.5, 3.2 Hz, 1H), 3.69-3.63 (m, 2H), 3.46 (s, 3H), 3.45 (s, 3H), 3.40 (s, 3H), 1.33 (t, *J* = 7.1 Hz, 3H); ^{19}F NMR (376 MHz, $CDCl_3$) δ -105.73 (d, *J* = 257.5 Hz, 1F), -110.73 (d, *J* = 257.5 Hz, 1F); ^{13}C NMR (101 MHz, $CDCl_3$) δ 163.1 (t, J_{CF} = 33.3 Hz), 131.6 (t, J_{CF} = 6.1 Hz), 131.1 (t, J_{CF} = 22.2 Hz), 111.8 (t, J_{CF} = 250.5 Hz), 94.2 (dd, J_{CF} = 5.3, 2.0 Hz), 71.5, 71.1, 68.3, 63.0, 59.4, 56.9, 56.2, 13.9. HRMS (ESI) *m/z* $[M + Na]^+$ calculated for $C_{13}H_{20}NaF_2O_6$ 333.1126, found 333.1122.

The synthesis of compound 3c

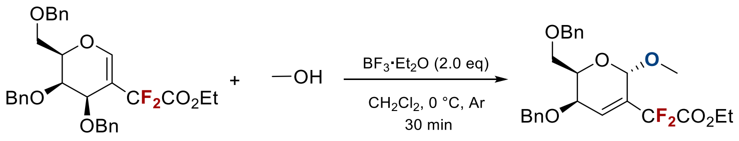

1c (2.0 eq) **3c**

According to the *General glycosylation procedure A*, reaction mixture was purified by column chromatography on silica gel (petroleum ether : ethyl acetate = 10 : 1) to give **3c** as a colorless oil (39.7 mg, 86%). ^{1}H NMR (400 MHz, $CDCl_3$) δ 7.39-7.30 (m, 10H), 6.58 (dd, J = 5.3, 3.0 Hz, 1H), 5.10 (s, 1H), 4.71-4.55 (m, 4H), 4.35-4.28 (m, 2H), 4.24 (td, J = 6.3, 2.6 Hz, 1H), 3.91-3.89 (m, 1H), 3.87-3.76 (m, 2H), 3.42 (s, 3H), 1.34 (t, J = 7.1 Hz, 3H); ^{19}F NMR (376 MHz, $CDCl_3$) δ -106.46 (d, J = 256 Hz, 1F), -111.76 (d, J = 256 Hz, 1F); ^{13}C NMR (101 MHz, $CDCl_3$) δ 163.05 (t, J_{CF} = 33.8 Hz), 138.16, 137.88, 133.64 (t, J_{CF} = 23.7 Hz), 128.47, 128.40, 128.14, 128.07, 127.95, 127.93, 127.67, 127.61, 111.97 (t, J_{CF} = 252.5Hz), 93.71, 73.52, 71.62, 68.98, 66.47, 63.03, 55.89, 13.87. HRMS (ESI) *m/z* [M + Na]$^+$ calculated for $C_{25}H_{28}NaF_2O_6$ 485.1752, found 485.1758.

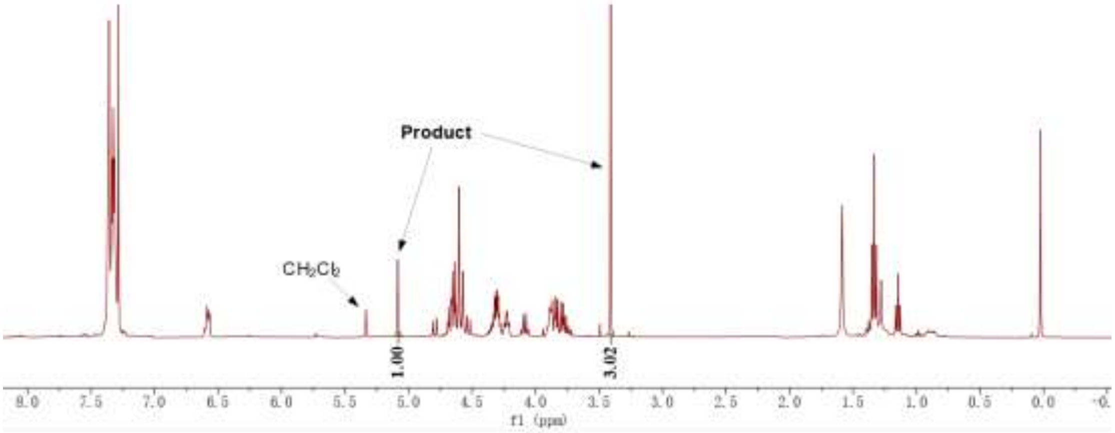

Crude product analysis of 3c

The synthesis of compound 3d

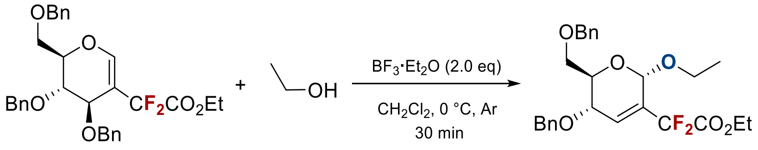

1a (2.0 eq) **3d**

According to the *General glycosylation procedure A*, reaction mixture was purified by column chromatography on silica gel (petroleum ether : ethyl acetate = 10 :1) to give **3d** as a colorless oil (42.4 mg, 89%). ^{1}H NMR (400 MHz, $CDCl_3$) δ 7.36-7.27 (m, 10H), 6.58 (s, 1H), 5.16 (s, 1H), 4.69-4.65 (m, 2H), 4.54-4.47 (m, 2H), 4.34-4.27 (m, 3H), 4.00 (dt, J = 9.6, 2.9 Hz, 1H), 3.85-3.71 (m, 3H), 3.59-3.51 (m, 1H), 1.35 (t, J = 7.1 Hz, 3H), 1.18 (t, J = 7.1 Hz, 3H); ^{19}F NMR (376 MHz, $CDCl_3$) δ -105.90 (d, J = 257.6 Hz, 1F), -110.69 (d, J = 257.6 Hz, 1F); ^{13}C NMR (101 MHz, $CDCl_3$) δ 163.0 (dd, J_{CF} = 36.4, 32.0 Hz), 138.0, 137.6, 131.7 (dd, J_{CF} = 8.5, 7.0 Hz), 131.1 (dd, J_{CF} = 24.8, 22.6 Hz), 128.5, 128.4, 128.3, 128.0, 128.0, 127.8, 127.7, 111.8 (dd, J_{CF} = 252.7, 248.6 Hz), 93.0 (dd, J_{CF} = 5.3, 2.0 Hz), 73.5, 71.5, 69.9, 68.7, 68.4, 64.6, 62.9, 15.0, 13.9. HRMS (ESI) *m/z* [M + Na]$^+$ calculated for $C_{26}H_{30}NaF_2O_6$ 499.1908, found 499.1907.

The synthesis of compound 3e

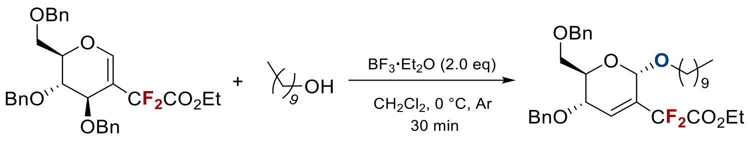

1a (2.0 eq) **3e**

According to the *General glycosylation procedure A*, reaction mixture was purified by column chromatography on silica gel (petroleum ether : ethyl acetate = 15 :1) to give **3e** as a colorless oil (53.1 mg, 89%). ^{1}H NMR (400 MHz, $CDCl_3$) δ 7.37-7.26 (m, 10H), 6.57 (s, 1H), 5.13 (s, 1H), 4.69-4.65 (m, 2H), 4.53-4.46 (m, 2H), 4.29 (q, J = 7.1 Hz, 3H), 4.00-3.97 (m, 1H), 3.79-3.70 (m, 3H), 3.50-3.45 (m, 1H), 1.55-1.50 (m, 2H), 1.34 (t, J = 7.2 Hz, 3H), 1.30-1.27 (m, 16H), 0.90 (t, J = 6.8 Hz, 3H); ^{19}F NMR (376 MHz, $CDCl_3$) δ -105.94 (d, J = 258.1 Hz, 1F), -110.34 (d, J = 258.1 Hz, 1F); ^{13}C NMR (101 MHz, $CDCl_3$) δ 138.0, 137.5, 131.7 (t, J_{CF} = 8.1 Hz), 131.2 (t, J_{CF} = 23.7 Hz), 128.5, 128.4, 128.0, 127.8, 127.7, 93.1, 93.1 (d, J_{CF} = 5.3 Hz), 73.4, 71.5, 69.9, 69.3, 68.7, 68.3, 62.9, 31.9, 29.7, 29.6, 29.6, 29.4, 29.3, 26.0, 22.7, 14.1, 13.9. HRMS (ESI) *m/z* $[M + Na]^+$ calculated for $C_{34}H_{46}NaF_2O_6$ 606.3606, found 606.3602.

The synthesis of compound 3f

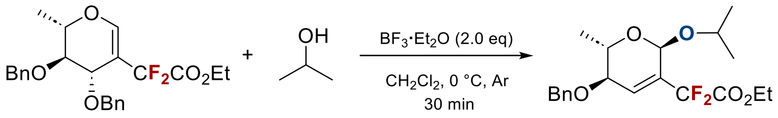

1d (2.0 eq) **3f**

According to the *General glycosylation procedure A*, reaction mixture was purified by column chromatography on silica gel (petroleum ether : ethyl acetate = 8 :1) to give **3f** as a colorless oil (30.0 mg, 78%). ^{1}H NMR (400 MHz, $CDCl_3$) δ 7.40-7.31 (m, 5H), 6.55 (s, 1H), 5.15 (s, 1H), 4.75 (d, J = 11.5 Hz, 1H), 4.58 (d, J = 11.5 Hz, 1H), 4.37-4.20 (m, 2H), 4.01-3.88 (m, 2H), 3.82-3.73 (m, 1H), 1.35 (t, J = 7.2 Hz, 3H), 1.30 (d, J = 6.2 Hz, 3H), 1.19 (d, J = 6.2 Hz, 3H), 1.13 (d, J = 6.2 Hz, 3H); ^{19}F NMR (376 MHz, $CDCl_3$) δ -107.39 (d, J = 256 Hz, 1F), -109.41 (d, J = 259 Hz, 1F); ^{13}C NMR (101 MHz, $CDCl_3$) δ 163.28, 162.96, 162.92, 162.60 (dd, J_{CF} = 32.3, 36.4 Hz), 137.63, 131.65 (t, J_{CF} = 20.2 Hz), 131.50 (t, J_{CF} = 22.2 Hz), 128.51, 128.00, 127.96, 111.97 (dd, J_{CF} = 249.5, 252.5 Hz), 91.42, 91.37, 75.86, 71.19, 71.17, 64.94, 62.77, 23.65, 21.40, 17.84, 13.93. HRMS (ESI) *m/z* $[M + Na]^+$ calculated for $C_{20}H_{26}NaF_2O_5$ 407.1646, found 407.1642.

The synthesis of compound 3g

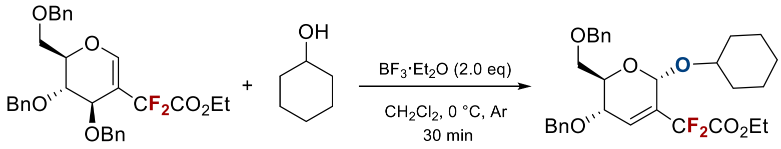

1a (2.0 eq) **3g**

According to the *General glycosylation procedure A*, reaction mixture was purified by column chromatography on silica gel (petroleum ether : ethyl acetate = 12 :1) to give **3g** as a colorless oil (46.1 mg, 87%). ^{1}H NMR (400 MHz, $CDCl_3$) δ 7.36-7.27 (m, 10H), 6.55 (s, 1H), 5.30 (s, 1H), 4.67 (dd, J = 11.8, 5.7 Hz, 2H), 4.49 (t, J = 12.3 Hz, 2H), 4.32-4.27 (m, 3H), 4.04 (d, J = 9.0 Hz, 1H), 3.79-3.64 (m, 3H), 1.88 (t, J = 11.2 Hz, 1H), 1.77-1.64 (m,

1H), 1.52 (dd, J = 10.3, 2.8 Hz, 2H), 1.34 (t, J = 7.1 Hz, 3H), 1.28-1.22 (mii, 6H); ^{19}F NMR (376 MHz, $CDCl_3$) δ -107.32 (d, J = 259.4 Hz, 1F), -109.13 (d, J = 259.4 Hz, 1F); ^{13}C NMR (101 MHz, $CDCl_3$) δ 162.9 (dd, J_{CF} = 36.7, 32.4 Hz), 138.1, 137.6, 131.7 (dd, J_{CF} = 8.9, 6.5 Hz), 131.33 (d, J_{CF} = 23.0 Hz), 128.5, 128.4, 128.0, 127.8, 127.7, 111.9 (t, J_{CF} = 251.5 Hz), 91.6 (t, J_{CF} = 3.7 Hz), 73.4, 71.4, 70.0, 68.5, 62.8, 33.7, 31.5, 25.6, 24.2, 24.0, 14.0. HRMS (ESI) *m/z* [M + Na]$^+$ calculated for $C_{30}H_{36}NaF_2O_6$ 553.2378, found 553.2377.

The synthesis of compound 3h

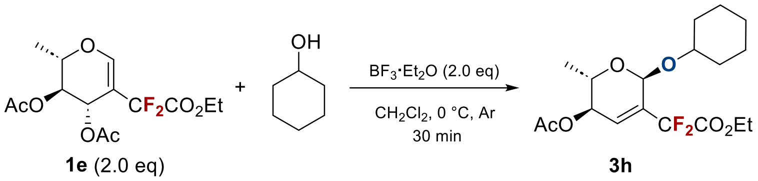

According to the *General glycosylation procedure A*, reaction mixture was purified by column chromatography on silica gel (petroleum ether : ethyl acetate = 15 :1) to give **3h** as a colorless oil (65.4 mg, 87%). ^{1}H NMR (400 MHz, $CDCl_3$) δ 6.31 (s, 1H), 5.23 (s, 1H), 5.13 (dd, J = 8.6, 4.9 Hz, 1H), 4.58-4.17 (m, 2H), 4.09-3.96 (m, 1H), 3.61 (s, 1H), 2.13 (s, 3H), 1.93-1.83 (m, 2H), 1.78-1.63 (m, 2H), 1.60-1.48 (m, 1H), 1.39-1.19 (m, 11H); ^{19}F NMR (376 MHz, $CDCl_3$) δ -107.70 (d, J = 259 Hz, 1F), -109.07 (d, J = 259 Hz, 1F); ^{13}C NMR (101 MHz, $CDCl_3$) δ 170.27, 162.79 (t, J_{CF} = 34.3 Hz), 132.67 (t, J_{CF} = 24.2 Hz), 130.89 (t, J_{CF} = 8.1 Hz), 111.76 (t, J_{CF} = 252.0 Hz), 91.48, 91.45, 91.41, 77.53, 70.14, 64.14, 62.89, 33.76, 31.53, 25.56, 24.15, 23.94, 20.95, 17.56, 13.90. HRMS (ESI) *m/z* [M + Na]$^+$ calculated for $C_{18}H_{26}NaF_2O_6$ 399.1697, found 399.1697.

The synthesis of compound 3i

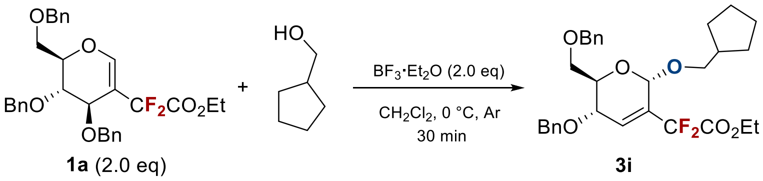

According to the *General glycosylation procedure A*, reaction mixture was purified by column chromatography on silica gel (petroleum ether : ethyl acetate = 12 :1) to give **3i** as a colorless oil (44.6 mg, 84%). ^{1}H NMR (400 MHz, $CDCl_3$) δ 7.37-7.22 (m, 10H), 6.55 (t, J = 2.4 Hz, 1H), 5.12 (s, 1H), 4.65 (dd, J = 11.7, 5.6 Hz, 2H), 4.48 (dd, J = 16.1, 11.7 Hz, 2H), 4.31-4.23 (m, 3H), 4.01-3.96 (m, 1H), 3.77-3.68 (m, 2H), 3.58 (dd, J = 9.2, 7.7 Hz, 1H), 3.38 (dd, J = 9.2, 6.6 Hz, 1H), 2.15-2.02 (m, 1H), 1.74-1.62 (m, 2H), 1.60-1.44 (m, 4H), 1.32 (t, J = 7.1 Hz, 3H), 1.24-1.13 (m, 2H); ^{19}F NMR (376 MHz, $CDCl_3$) δ -106.22 (d, J = 243 Hz), -109.75 (d, J = 243 Hz); ^{13}C NMR (101 MHz, $CDCl_3$) δ 14.05, 25.50, 25.51, 29.42, 29.84, 39.35, 63.02, 68.48, 68.79, 70.00, 71.64, 73.55, 73.72, 93.21 (dd, J_{CF} = 5.4, 2.2 Hz), 109.45, 111.92, 114.43, 127.81, 127.96, 128.15, 128.48, 128.61, 131.26 (dd, J_{CF} = 24.7, 22.7 Hz), 131.86 (t, J_{CF} = 7.7 Hz), 137.57, 138.10. 163.05 (dd, J_{CF} = 36.6, 32.4 Hz). HRMS (ESI) *m/z* [M + Na]$^+$ calculated for $C_{30}H_{36}NaF_2O_6$ 553.2378, found 553.2382.

The synthesis of compound 3j

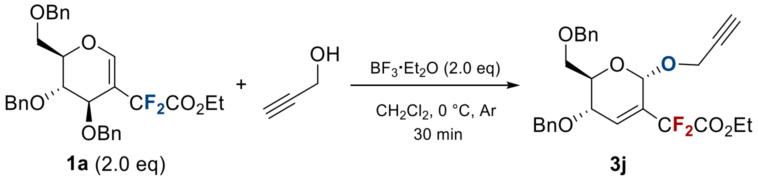

According to the *General glycosylation procedure A*, reaction mixture was purified by column chromatography on silica gel (petroleum ether : ethyl acetate = 10 :1) to give **3j** as a colorless oil (37.5 mg, 77%). ^{1}H NMR (400 MHz, $CDCl_3$) δ 7.37-7.23 (m, 10H), 6.61 (t, *J* = 2.4 Hz, 1H), 5.31 (s, 1H), 4.64 (dd, *J* = 11.8, 9.2 Hz, 2H), 4.55-4.43 (m, 2H), 4.39-4.23 (m, 5H), 3.98 (dt, *J* = 9.5, 3.0 Hz, 1H), 3.78-3.68 (m, 2H), 2.42 (t, *J* = 2.4 Hz, 1H), 1.34 (t, *J* = 7.1 Hz, 3H); ^{19}F NMR (376 MHz, $CDCl^3$) δ -105.64 (d, *J* = 243 Hz), -109.92 (d, *J* = 243 Hz); ^{13}C NMR (101 MHz, $CDCl_3$) δ 14.03, 55.58, 63.25, 68.15, 69.12, 69.73, 71.64, 73.57, 74.93, 78.69, 91.82 (dd, J_{CF} = 5.2, 2.0 Hz).109.19, 111.68, 114.19, 127.88, 127.99, 128.09, 128.15, 128.53, 128.60, 130.61 (dd, J_{CF} = 24.9, 23.1 Hz)., 132.82 (dd, J_{CF} = 8.3, 8.1 Hz), 137.50, 137.98, 163.04 (dd, J_{CF} = 35.4, 32.5 Hz). HRMS (ESI) *m/z* $[M + Na]^+$ calculated for $C_{27}H_{28}NaF_2O_6$ 509.1752, found 509.1753.

The synthesis of compound 3k

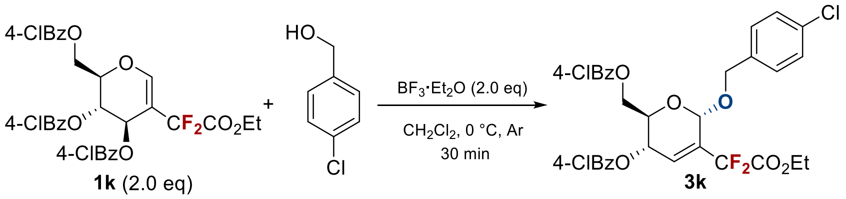

According to the *General glycosylation procedure A*, reaction mixture was purified by column chromatography on silica gel (petroleum ether : ethyl acetate = 10 :1) to give **3k** as a colorless oil (55.6 mg, 83%). ^{1}H NMR (400 MHz, $CDCl_3$) δ 7.97-7.95 (m, 2H), 7.92-7-90 (m, 2H), 7.43-7.40 (m, 2H), 7.37-7.34 (m, 2H), 7.31-7.29 (m, 2H), 7.22-7.20 (m, 2H), 6.53 (t, *J* = 2.4 Hz, 1H), 5.75 (t, *J* = 7.0 Hz, 1H), 5.31 (s, 1H), 4.74 (d, *J* = 11.4 Hz, 1H), 4.59-4.55 (m, 1H), 4.53 (d, *J* = 11.4 Hz, 1H), 4.45 (dd, *J* = 8.7, 1.0 Hz, 2H), 4.17-4.12 (m, 2H), 1.20 (t, *J* = 7.2 Hz, 3H); ^{19}F NMR (376 MHz, $CDCl_3$) δ -106.35 (d, *J* = 259 Hz, 1F), -109.36 (d, *J* = 259 Hz, 1F); ^{13}C NMR (101 MHz, $CDCl_3$) δ 165.25, 164.74, 162.65 (dd, J_{CF} = 35.5, 32.0 Hz), 140.36, 139.85, 135.04, 133.95, 132.54 (m), 131.24, 130.99, 129.42, 128.94, 128.79, 128.59, 127.92, 127.32, 111.37 (dd, J_{CF} = 253.7, 250.5 Hz), 92.32 (dd, J_{CF} = 4.8, 1.7 Hz), 70.23, 66.83, 65.75, 63.43, 63.24, 29.71, 13.78. HRMS (ESI) *m/z* $[M + NH_4]^+$ calculated for $C_{31}H_{29}NF_2O_8Cl_3$ 686.0927, found 686.0931.

The synthesis of compound 3l

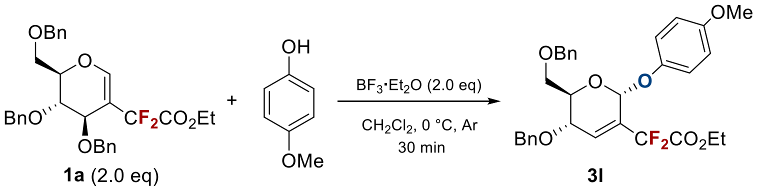

According to the *General glycosylation procedure A*, reaction mixture was purified by column chromatography on silica gel (petroleum ether : ethyl acetate = 7 :1) to give **3l** as a colorless oil (43.3 mg, 78%). ^{1}H NMR (400 MHz, $CDCl_3$) δ 7.40-7.29 (m, 10H), 7.04 (d, *J* =

9.0 Hz, 2H), 6.79 (d, J = 9.0 Hz, 2H), 6.71 (s, 1H), 5.61 (s, 1H), 4.72 (d, J = 11.4 Hz, 1H), 4.63 (d, J = 12.0 Hz, 1H), 4.52 (dd, J = 11.7, 9.5 Hz, 2H), 4.37-4.26 (m, 3H), 4.25-4.17 (m, 1H), 3.77 (s,3H), 1.29 (t, J = 6.0 Hz, 3H); ^{19}F NMR (376 MHz, $CDCl_3$) δ -106.39 (d, J = 259 Hz, 1F), -109.27 (d, J = 259 Hz, 1F); ^{13}C NMR (101 MHz, $CDCl_3$) δ 155.65, 150.93, 137.95, 137.38, 128.53, 128.37, 128.10, 128.01, 127.85, 127.71, 119.66, 114.51, 93.60 (t, J_{CF} = 4.0 Hz), 73.44, 71.60, 69.74, 69.39, 68.39, 63.19, 55.62, 13.89. HRMS (ESI) m/z [M + Na]$^+$ calculated for $C_{31}H_{32}NaF_2O_7$ 577.2116, found 577.2115.

The synthesis of compound 4a

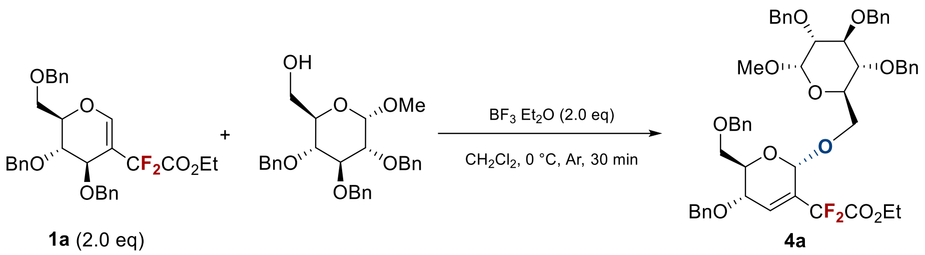

According to the *General glycosylation procedure A*, reaction mixture was purified by column chromatography on silica gel (petroleum ether : ethyl acetate = 8 : 1) to give **4a** as a colorless oil (78.8 mg, 88%). ^{1}H NMR (400 MHz, $CDCl_3$) δ 7.36-7.21 (m, 25H), 6.52 (t, J = 2.2 Hz, 1H), 5.23 (s, 1H), 4.97 (d, J = 11.0 Hz, 1H), 4.85 (d, J = 11.0 Hz, 1H), 4.80-4.75 (m, 2H), 4.67-4.55 (m, 4H), 4.52 (d, J = 3.5 Hz, 1H), 4.44-4.37 (m, 2H), 4.29-4.25 (m, 1H), 4.19 (q, J = 7.1 Hz, 2H), 3.98-3.91 (m, 2H), 3.88 (dd, J = 11.2, 4.6 Hz, 1H), 3.75 (dd, J = 11.2, 1.9 Hz, 1H), 3.70-3.65 (m, 2H), 3.56 (dd, J = 10.8, 2.1 Hz, 2H), 3.52 (dd, J = 9.7, 3.5 Hz, 1H), 3.45 (dd, J = 10.0, 8.9 Hz, 1H), 3.33 (s, 3H), 1.22 (t, J = 7.2 Hz, 3H); ^{19}F NMR 376 MHz, $CDCl_3$) δ -105.65 (d, J = 261.9 Hz, 1F), -107.03 (d, J = 261.8 Hz, 1F); ^{13}C NMR (101 MHz, $CDCl_3$) δ 162.8 (t, J_{CF} = 35.6 Hz), 138.9, 138.4, 138.2, 137.9, 137.5, 132.7 (t, J_{CF} = 7.9 Hz), 131.0 (t, J_{CF} = 24.5 Hz), 128.5, 128.4, 128.4, 128.4, 128.3, 128.2, 128.0, 128.0, 127.9, 127.8, 127.8, 127.7, 127.6, 127.6, 127.5, 111.8 (t, J_{CF} = 252.3 Hz), 97.9, 93.3, 82.0, 79.8, 77.8, 75.5, 74.8, 73.4, 73.3, 71.6, 70.1, 69.7, 68.7, 68.1, 67.2, 63.0, 55.1, 13.9. HRMS (ESI) m/z [M + Na]$^+$ calculated for $C_{52}H_{56}NaF_2O_{11}$ 912.4135, found 912.4147.

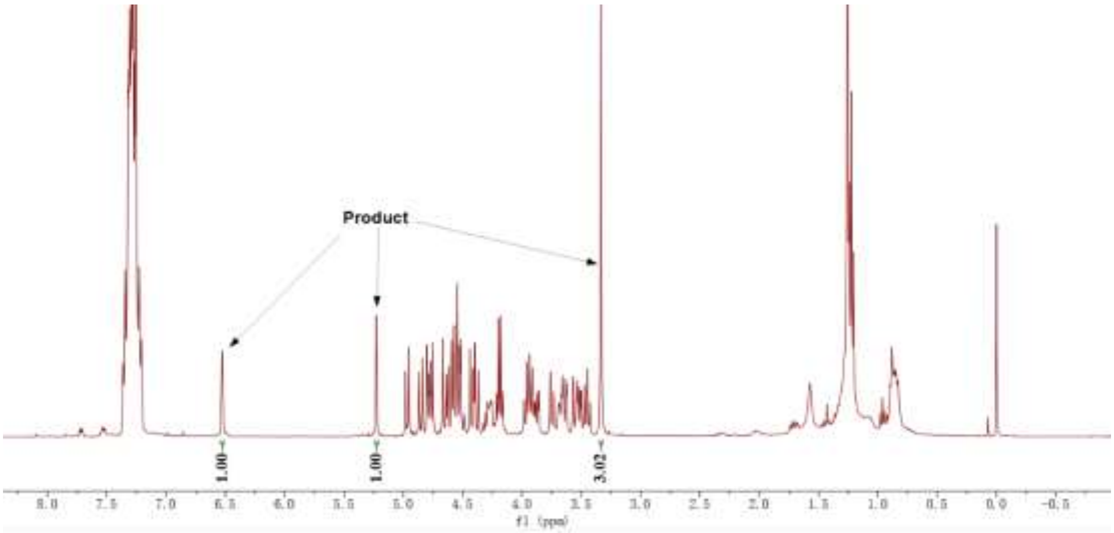

Crude product analysis of 4a

The synthesis of compound 4b

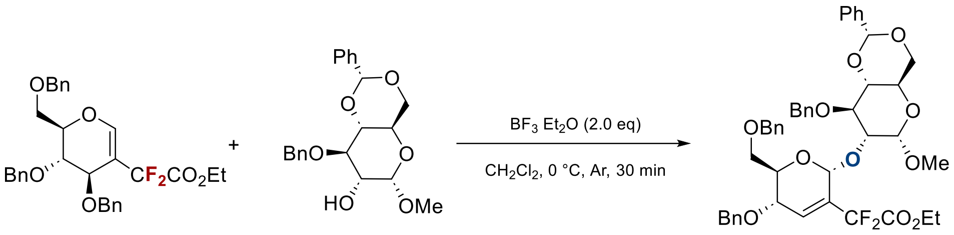

1a (2.0 eq) **4b**

According to the *General glycosylation procedure A*, reaction mixture was purified by column chromatography on silica gel (petroleum ether : ethyl acetate = 9 :1) to give **4b** as a colorless oil (62.3 mg, 78%). ^{1}H NMR (400 MHz, $CDCl_3$) δ 7.48-7.46 (m, 2H), 7.39-7.27 (m, 11H), 7.23-7.11 (m, 7H), 6.57 (s, 1H), 5.57 (s, 1H), 5.22 (s, 1H), 4.87-4.81 (m, 2H), 4.60-4.50 (m, 3H), 4.38-4.26 (m, 5H), 4.21 (d, *J* = 12.1 Hz, 1H), 4.06 (d, *J* = 9.5 Hz, 1H), 3.91-3.82 (m, 3H), 3.74 (t, *J* = 10.2 Hz, 1H), 3.65-3.60 (m,1H), 3.43 (s, 3H), 3.26-3.20 (m, 2H), 1.33 (t, *J* = 7.1 Hz, 3H); ^{19}F NMR (376 MHz, $CDCl_3$) δ -106.77 (d, *J* = 263.2 Hz, 1F), -107.55 (d, *J* = 263.2 Hz, 1F); ^{13}C NMR (101 MHz, $CDCl_3$) δ 162.8 (t, J_{CF} = 35.4 Hz), 138.9, 138.4, 138.2, 137.9, 137.5, 132.7 (t, J_{CF} = 7.1 Hz), 131.0 (t, J_{CF} = 24.2 Hz), 128.5, 128.4, 128.4, 128.4, 128.3, 128.2, 128.0, 128.0, 127.9, 127.8, 127.8, 127.7, 127.6, 127.6, 127.5, 111.8 (t, J_{CF} = 249.5 Hz), 97.9, 93.3, 82.0, 79.8, 77.8, 75.5, 74.9, 73.4, 73.3, 71.6, 70.1, 69.7, 68.7, 68.1, 67.2, 63.0, 55.1, 13.9. HRMS (ESI) *m/z* $[M + Na]^+$ calculated for $C_{45}H_{48}NaF_2O_{11}$ 820.3509, found 820.3519.

The synthesis of compound 4c

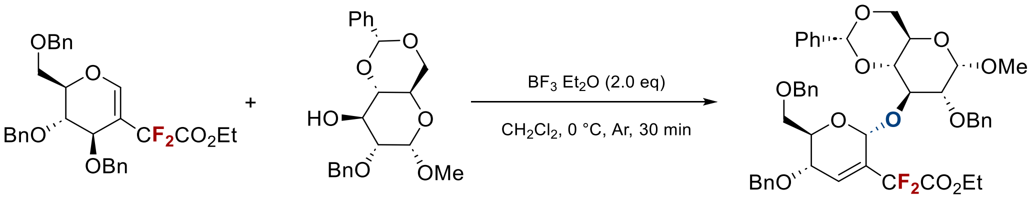

1a (2.0 eq) **4c**

According to the *General glycosylation procedure A*, reaction mixture was purified by column chromatography on silica gel (petroleum ether : ethyl acetate = 8 :1) to give **4c** as a colorless oil (59.4 mg, 74%). ^{1}H NMR (400 MHz, $CDCl_3$) δ 7.50-7.48 (m, 2H), 7.35-7.19 (m, 18H), 6.53 (s, 1H), 5.75 (s, 1H), 5.60 (s, 1H), 4.65-4.51 (m, 5H), 4.43-4.33 (m, 4H), 4.26-4.15 (m, 3H), 3.98 (d, *J* = 9.5 Hz, 1H), 3.82-3.76 (m, 1H), 3.72 (t, *J* = 10.0 Hz, 1H), 3.62-3.55 (m, 2H), 3.51 (dd, *J* = 9.6, 2.5 Hz, 1H), 3.39 (s, 3H), 3.33 (dd, *J* = 9.3, 3.8 Hz, 1H), 1.30 (t, *J* = 7.1 Hz, 3H); ^{19}F NMR (376 MHz, $CDCl_3$) δ -105.90 (d, *J* = 258.4 Hz, 1F), -109.31 (d, *J* = 258.7 Hz, 1F); ^{13}C NMR (101 MHz, $CDCl_3$) δ 138.1, 137.9, 137.8, 137.3, 132.9 (t, J_{CF} = 7.1 Hz), 128.8, 128.4, 128.4, 128.3, 128.2, 128.1, 128.0, 127.9, 127.9, 127.6, 126.2, 126.2, 101.4, 98.8, 92.4, 82.2, 78.2, 73.7, 73.4, 71.8, 69.6, 69.0, 68.6, 67.7, 62.7, 61.8, 55.3, 29.7, 14.1. HRMS (ESI) m/z $[M + Na]^+$ calculated for $C_{45}H_{48}NaF_2O_{11}$ 820.3509, found 820.3510.

The synthesis of compound 4d

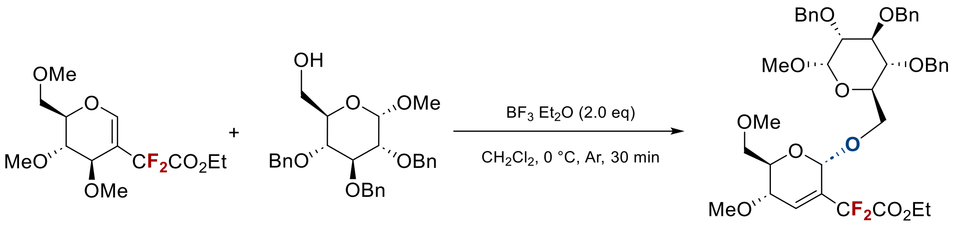

1f (2.0 eq) **4d**

According to the *General glycosylation procedure A*, reaction mixture was purified by column chromatography on silica gel (petroleum ether : ethyl acetate = 7 :1) to give **4d** as a colorless oil (63.2 mg, 85%). ^{1}H NMR (400 MHz, $CDCl_3$) δ 7.41-7.28 (m, 15H), 6.58 (t, J = 2.1 Hz, 1H), 5.25 (s, 1H), 5.01 (d, J = 11.0 Hz, 1H), 4.90 (d, J = 10.9 Hz, 1H), 4.84-4.80 (m, 2H), 4.70 (d, J = 12.1 Hz, 1H), 4.61 (d, J = 10.9 Hz, 1H), 4.57 (d, J = 3.5 Hz, 1H), 4.26-4.18 (m, 2H), 4.04-3.98 (m, 2H), 3.92 (dd, J = 11.3, 4.6 Hz, 1H), 3.86 (ddd, J = 9.6, 3.7, 2.1 Hz, 1H), 3.79 (dd, J = 11.2, 1.9 Hz, 1H), 3.73-3.69 (m, 1H), 3.62-3.47 (m, 4H), 3.45 (s, 3H), 3.37 (s, 6H), 1.26 (t, J = 7.1 Hz, 3H); ^{19}F NMR (376 MHz, $CDCl_3$) δ -105.96 (d, J = 261.7 Hz, 1F), -107.13 (d, J = 260.9 Hz, 1F); ^{13}C NMR (101 MHz, $CDCl_3$) δ 162.8 (dd, J_{CF} = 35.0, 33.6 Hz), 138.9, 138.4, 138.3, 132.2 (t, J_{CF} = 7.7 Hz), 131.1 (t, J_{CF} = 23.9 Hz), 128.4, 128.4, 128.3, 128.2, 127.9, 127.8, 127.7, 127.7, 127.5, 111.8 (t, J_{CF} = 251.1 Hz), 97.9, 93.3 (t, J_{CF} = 3.3 Hz), 82.0, 79.8, 75.6, 74.9, 73.4, 71.3, 70.8, 70.1, 68.4, 67.3, 63.0, 59.4, 56.9, 55.2, 13.9. HRMS (ESI) *m/z* $[M + NH_4]^+$ calculated for $C_{40}H_{52}NF_2O_{10}$ 760.3508, found 760.3504.

The synthesis of compound 4e

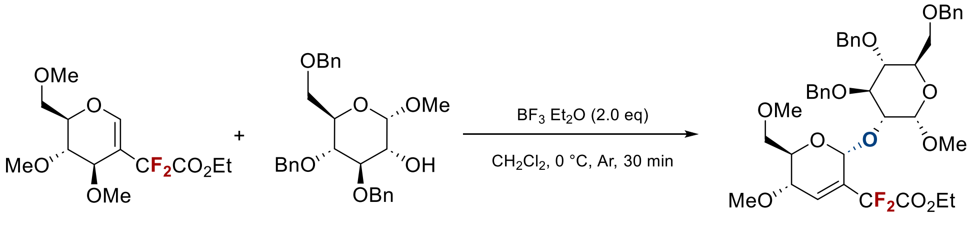

1f (2.0 eq) **4e**

According to the *General glycosylation procedure A*, reaction mixture was purified by column chromatography on silica gel (petroleum ether : ethyl acetate = 10 :1) to give **4e** as a colorless oil (60.2 mg, 81%). ^{1}H NMR (400 MHz, $CDCl_3$) δ 7.40-7.26 (m, 13H), 7.17-7.14 (m, 2H), 6.60 (d, J = 2.0 Hz, 1H), 5.26 (s, 1H), 4.89-4.77 (m, 4H), 4.68 (d, J = 12.1 Hz, 1H), 4.56-4.53 (m, 2H), 4.39-4.28 (m, 2H), 4.13-4.09 (m, 1H), 3.95-3.85 (m, 3H), 3.82-3.77 (m, 2H), 3.73-3.68 (m, 2H), 3.44 (s, 3H), 3.39 (s, 3H), 3.35-3.32 (m, 4H), 3.25 (dd, J = 11.0, 2.5 Hz, 1H), 1.37 (t, J = 7.1 Hz, 3H); ^{19}F NMR (376 MHz, $CDCl_3$) δ -106.80 (d, J = 267.0 Hz, 1F), -107.63 (d, J = 263.2 Hz, 1F); ^{13}C NMR (101 MHz, $CDCl_3$) δ 162.7 (t, J_{CF} = 34.3 Hz), 138.7, 138.2, 138.1, 133.3 (t, J_{CF} = 7.7 Hz), 130.6 (t, J_{CF} = 23.9 Hz), 128.4, 128.4, 128.3, 127.9, 127.8, 127.8, 127.7, 127.4, 111.8 (t, J_{CF} = 252.5 Hz), 96.8, 90.35 (d, J_{CF} = 3.5 Hz), 80.5, 78.1, 75.9, 75.5, 75.1, 73.5, 70.8, 70.2, 70.1, 68.6, 68.3, 63.0, 59.3, 56.9, 55.2, 14.0. HRMS (ESI) *m/z* $[M + NH_4]^+$ calculated for $C_{40}H_{52}NF_2O_{10}$ 760.3508, found 760.3504.

The synthesis of compound 4f

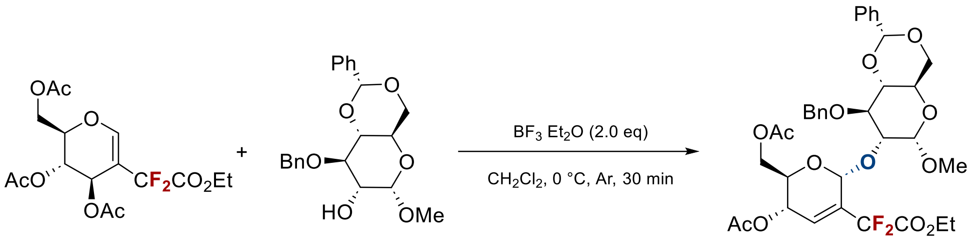

According to the *General glycosylation procedure B*, reaction mixture was purified by column chromatography on silica gel (petroleum ether : ethyl acetate = 10 :1) to give **4f** as a colorless oil (68.6 mg, 97%). ^{1}H NMR (400 MHz, $CDCl_3$) δ 7.50-7.29 (m, 10H), 6.41 (s, 1H), 5.62 (s, 1H), 5.41 (d, *J* = 10.0 Hz, 1H), 5.26 (s, 1H), 4.95 (d, *J* = 10.9 Hz, 1H), 4.83 (d, *J* = 3.3 Hz, 1H), 4.64 (d, *J* = 10.9 Hz, 1H), 4.41-4.30 (m, 3H), 4.18 (dt, *J* = 10.1, 2.5 Hz, 1H), 4.02-3.63 (m, 7H), 3.46 (s, 3H), 2.12 (s, 3H), 2.04 (s, 3H), 1.38 (t, *J* = 7.1 Hz, 3H); ^{19}F NMR (376 MHz, $CDCl_3$) δ -107.03 (d, *J* = 263 Hz, 1F), -107.89 (d, *J* = 267 Hz, 1F); ^{13}C NMR (101 MHz, $CDCl_3$) δ 170.53, 169.95, 138.41, 137.35, 131.86 (t, J_{CF} = 8.1 Hz), 128.95, 128.42, 128.31, 128.25, 127.72, 125.98, 111.46 (t, J_{CF} = 250.0 Hz), 101.32, 97.71, 90.62, 83.04, 75.72, 75.38, 69.16, 66.21, 63.96, 63.17, 62.34, 61.43, 55.42, 30.92, 20.86, 20.71, 13.96. HRMS (ESI) m/z $[M + NH_4]^+$ calculated for $C_{35}H_{44}NF_2O_{13}$ 724.4782, found 724.4785.

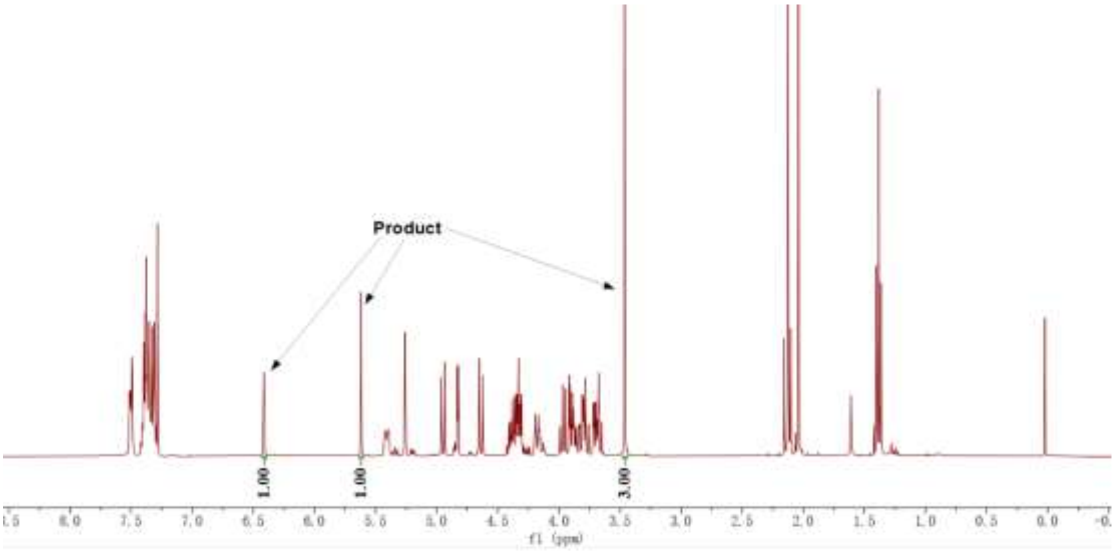

Crude product analysis of 4f

The synthesis of compound 4f-OH

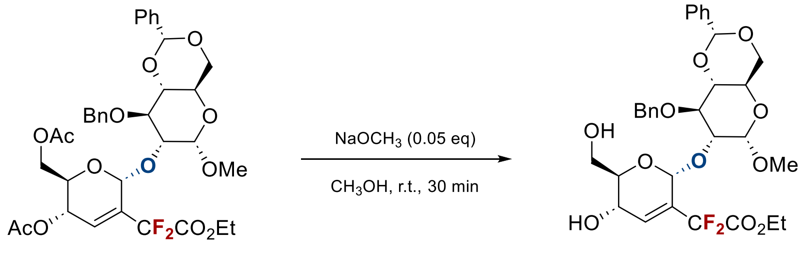

To a solution of **4f** (70.7 mg, 0.10 mmol) in CH_3OH (2 mL) was added $NaOCH_3$ (0.05 eq), followed by stir at room temperature for 30 min. The reaction mixture was purified by column chromatography on silica gel (petroleum ether : ethyl acetate = 3 :1) to give **4f-OH** as a white solid (56.7 mg, 91%). ^{1}H NMR (400 MHz, $CDCl_3$) δ 7.49-7.46 (m, 2H), 7.40-7.29 (m, 2H), 6.42 (s, 1H), 5.59 (s, 1H), 5.14 (s, 1H), 4.95 (d, J = 11.0 Hz, 1H), 4.79 (d, J = 3.5 Hz, 1H), 4.64 (d, J = 11.1 Hz, 1H), 4.40-4.29 (m, 3H), 4.15 (dd, J = 10.0, 4.6 Hz, 1H), 3.98 (t, J = 9.4 Hz, 1H), 3.89-3.85 (m, 2H), 3.77-3.71 (m, 2H), 3.67 (t, J = 9.3 Hz, 1H), 3.45-3.42 (m, 4H), 3.38 (dd, J = 11.6, 5.4 Hz, 1H), 31.37 (t, J = 7.1 Hz, 3H); ^{13}C NMR (101 MHz, $CDCl_3$) δ 162.6 (t, J_{CF} = 36.2 Hz), 138.5, 137.3, 135.7 (t, J_{CF} = 7.8 Hz), 130.3 (t, J_{CF} = 24.1 Hz), 129.0, 128.8, 128.5, 128.3, 128.0, 126.0, 111.7 (t, J_{CF} = 251.2 Hz), 101.4, 97.9, 90.4 (t, J_{CF} = 3.6 Hz), 83.1, 75.6, 75.2, 70.2, 69.2, 64.7, 63.1, 62.5, 62.4, 55.5, 14.0. HRMS (ESI) m/z $[M + NH_4]^+$ calculated for $C_{31}H_{40}NF_2O_{11}$ 640.2563, found 640.2550.

The synthesis of compound 4g

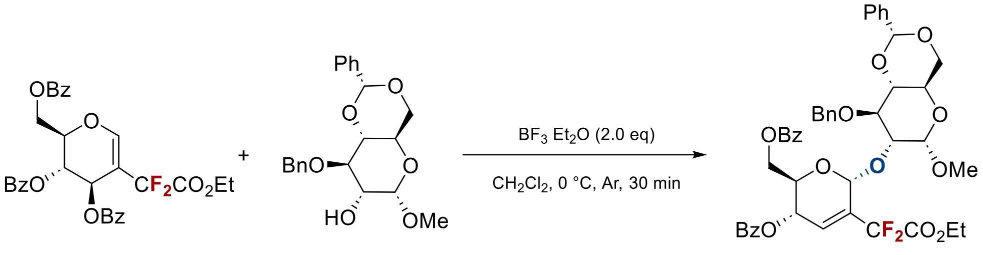

1h (2.0 eq) **4g**

According to the *General glycosylation procedure B*, reaction mixture was purified by column chromatography on silica gel (petroleum ether : ethyl acetate = 10 :1) to give **4g** as a colorless oil (68.6 mg, 97%). ^{1}H NMR (400 MHz, $CDCl_3$) δ 8.04-8.02 (m, 2H), 7.98-7.96 (m, 2H), 7.63-7.60 (m, 1H), 7.54-7.51 (m, 1H), 7.49-7.46 (m, 4H), 7.40-7.36 (m, 7H), 7.23-7.21 (m, 2H), 7.19-7.16 (m, 1H), 6.56 (t, J = 2.0 Hz, 1H), 5.75 (dd, J = 9.9, 3.4 Hz, 1H), 5.51 (s, 1H), 5.31 (s, 1H), 4.95 (d, J = 11.3 Hz, 1H), 4.83 (d, J = 2.6 Hz, 1H), 4.71 (d, J = 11.3 Hz, 1H), 4.53 (ddd, J = 9.9, 4.8, 2.5 Hz, 1H), 4.36-4.27 (m, 4H), 3.98-3.93 (m, 3H), 3.84 (td, J = 10.0, 4.8 Hz, 1H), 3.70 (t, J = 10.3 Hz, 1H), 3.53-3.50 (m, 1H), 3.44 (s, 3H), 1.34 (t, J = 7.2 Hz, 3H); ^{19}F NMR (376 MHz, $CDCl_3$) δ -106.85 (d, J = 263 Hz, 1F), -107.91 (d, J = 263 Hz, 1F); ^{13}C NMR (101 MHz, $CDCl_3$) δ 166.03, 165.56, 162.39 (m), 138.57, 137.36, 133.65, 132.98, 132.30 (m), 131.66 (t, J_{CF} = 8.1 Hz), 129.92, 129.88, 129.88, 129.84, 129.11, 128.96, 128.52, 128.27, 128.25, 128.11, 127.62, 125.97, 112.31 (m), 101.26, 97.63, 90.22 (t, J_{CF} = 3.3 Hz), 82.98, 76.42, 75.46, 75.05, 69.16, 66.48, 65.08, 63.20, 62.54, 62.35, 55.47, 13.97. HRMS (ESI) m/z $[M + NH_4]^+$ calculated for $C_{45}H_{48}NF_2O_{13}$ 848.3094, found 848.3109.

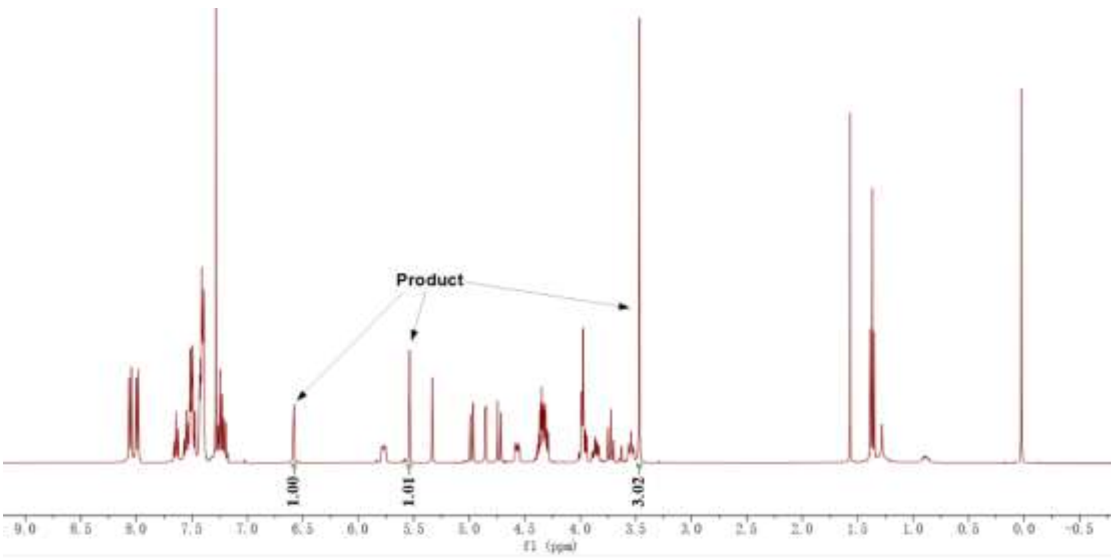

Crude product analysis of 4g

The synthesis of compound 4h

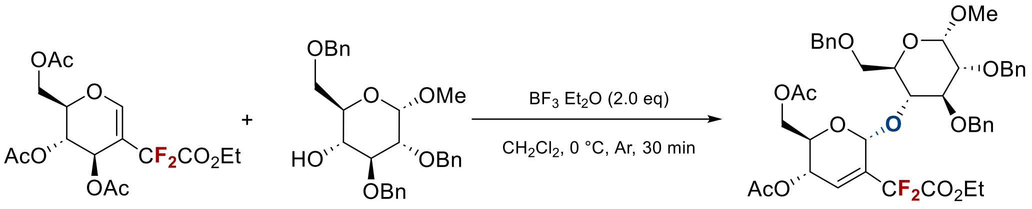

1g (2.0 eq) **4h**

According to the *General glycosylation procedure B*, reaction mixture was purified by column chromatography on silica gel (petroleum ether : ethyl acetate = 10 :1) to give **4h** as a colorless oil (75.1 mg, 94%). ^{1}H NMR (400 MHz, $CDCl_3$) δ 7.41-7.29 (m, 15H), 6.37 (s, 1H), 6.00 (s, 1H), 5.34 (dd, *J* = 9.3, 5.7 Hz, 1H), 5.14 (d, *J* = 11.0 Hz, 1H), 4.94 (d, *J* = 11.1 Hz, 1H), 4.72 (d, *J* = 12.0 Hz, 1H), 4.68-4.51 (m, 4H), 4.38-4.24 (m, 2H), 4.13-4.01 (m, 2H), 3.97 (dd, *J* = 12.2, 1.9 Hz, 1H), 3.85 (t, *J* = 9.2 Hz, 1H), 3.75-3.63 (m, 3H), 3.60-3.52 (m, 2H), 3.41 (s, 3H), 2.15 (s, 3H), 2.04 (s, 3H), 1.39 (t, *J* = 7.1 Hz, 3H); ^{19}F NMR (376 MHz, $CDCl_3$) δ -106.53 (d, *J* = 263 Hz, 1F), -108.97 (d, *J* = 263 Hz, 1F); ^{13}C NMR (101 MHz, $CDCl_3$) δ 170.55, 169.81, 139.02, 138.04, 137.86, 131.36 (t, J_{CF} = 5.1 Hz), 128.50, 128.42, 128.22, 128.18, 128.01, 127.66, 127.53, 127.43, 127.21, 97.60, 91.83, 81.03, 80.52, 74.04, 73.46, 73.22, 72.22, 69.66, 66.54, 64.40, 63.14, 62.06, 55.27, 20.89, 20.74, 13.96. HRMS (ESI) m/z $[M + NH_4]_+$ calculated for $C_{42}H_{52}NF_2O_{13}$ 821.3545, found 821.3548.

The synthesis of compound 4i

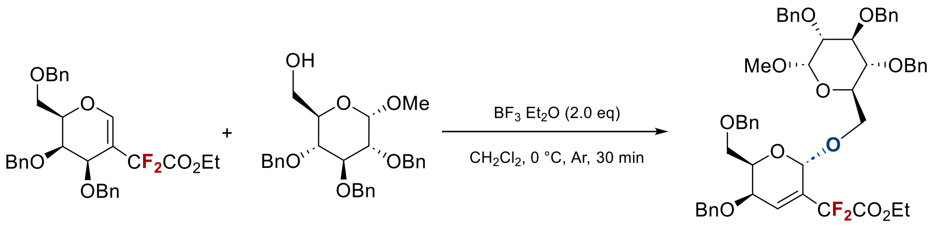

1j (2.0 eq) **4i**

According to the *General glycosylation procedure A*, reaction mixture was purified by column chromatography on silica gel (petroleum ether : ethyl acetate = 8 : 1) to give **4i** as a colorless oil (72.5 mg, 81%). ^{1}H NMR (400 MHz, $CDCl_3$) δ 7.42-7.29 (m, 25H), 6.55 (d, *J* = 3.9 Hz, 1H), 5.33 (s, 1H), 5.00 (d, *J* = 11.0 Hz, 1H), 4.89-4.77 (m, 3H), 4.72-4.49 (m, 7H), 4.31-4.18 (m, 3H), 3.99 (t, *J* = 9.2 Hz, 1H), 3.90 (dd, *J* = 11.3, 4.5 Hz, 2H), 3.85-3.79 (m, 2H), 3.74-3.67 (m, 2H), 3.55 (dd, *J* = 9.6, 3.3 Hz, 1H), 3.46 (t, *J* = 9.5 Hz, 1H), 3.37 (s, 3H), 1.25 (t, *J* = 7.1 Hz, 3H); ^{19}F NMR (376 MHz, $CDCl_3$) δ -106.54 (d, *J* = 259 Hz, 1F), -108.11 (d, *J* = 263 Hz, 1F); ^{13}C NMR (101 MHz, $CDCl_3$) δ 162.79 (t, J_{CF} = 34.3 Hz), 138.94, 138.33, 138.24, 138.09, 137.90, 133.47 (t, J_{CF} = 23.7 Hz), 128.86, 128.79, 128.71, 128.46, 128.43, 128.40, 128.37, 128.33, 128.15, 127.92, 127.86, 127.79, 127.69, 127.65, 127.57, 127.51, 112.06 (t, J_{CF} = 253.0 Hz), 97.90, 92.79, 81.95, 79.83, 77.89, 75.59, 74.95, 73.39, 73.33, 71.68, 70.19, 68.82, 68.66, 66.98, 66.42, 63.05, 55.15, 13.90. HRMS (ESI) *m/z* [M + Na]$^+$ calculated for $C_{52}H_{56}NaF_2O_{11}$ 912.4135, found 912.4128.

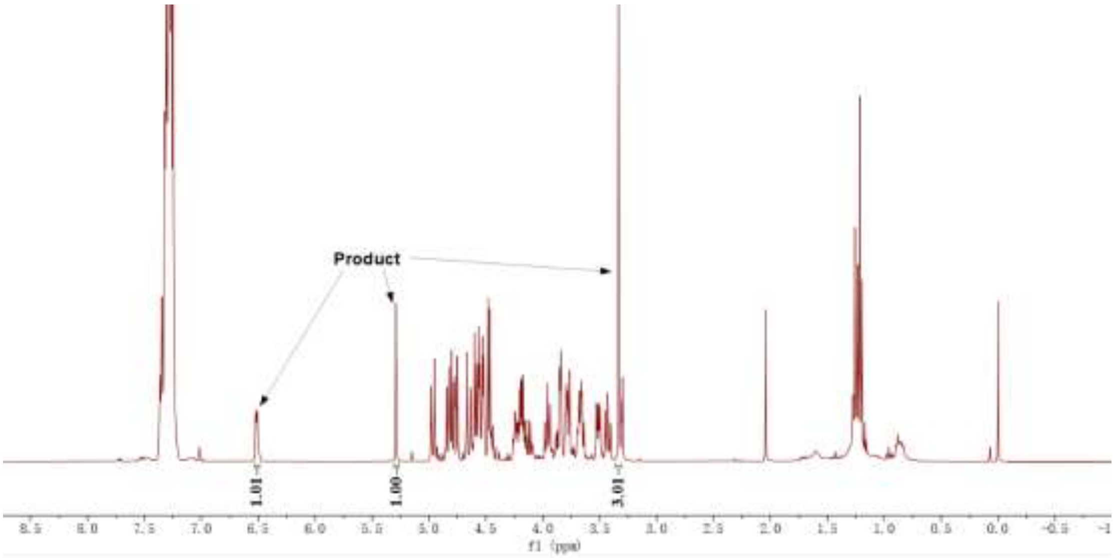

Crude product analysis of 4i

The synthesis of compound 4j

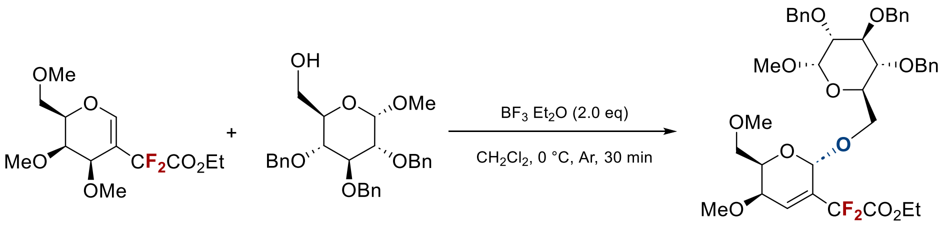

1k (2.0 eq) **4j**

According to the *General glycosylation procedure A*, reaction mixture was purified by column chromatography on silica gel (petroleum ether : ethyl acetate = 8 :1) to give **4j** as a colorless oil (57.2 mg, 86%). ^{1}H NMR (400 MHz, $CDCl_3$) δ 7.41-7.28 (m, 15H), 6.67 (dd, *J* = 5.2, 2.0 Hz, 1H), 5.31 (s, 1H), 5.01 (d, *J* = 11.0 Hz, 1H), 4.92-4.78 (m, 3H), 4.70 (d, *J* = 12.1 Hz, 1H), 4.63 (d, *J* = 10.8 Hz, 1H), 4.58 (d, *J* = 3.4 Hz, 1H), 4.29-4.16 (m, 3H), 4.00 (t, *J* = 9.3 Hz, 1H), 3.9 (dd, *J* = 11.4, 4.5 Hz, 1H), 3.82 (dd, *J* = 11.4, 1.8 Hz, 1H), 3.75-3.69 (m, 1H), 3.69-3.62 (m, 2H), 3.60-3.54 (m, 2H), 3.50 (t, 1H), 3.44 (s, 3H), 3.38 (s, 3H), 3.34 (s, 3H), 1.26 (t, *J* = 7.1 Hz, 3H); ^{19}F NMR (376 MHz, $CDCl_3$) δ -106.53 (d, *J* = 263 Hz, 1F),

-107.96 (d, J = 263 Hz, 1F); ^{13}C NMR (101 MHz, CDCl^3) δ 162.77 (t, J_{CF} = 34.3 Hz), 138.94, 138.36, 138.25, 133.84(t, J_{CF} = 23.7 Hz), 128.43, 128.39, 128.34, 128.18, 127.87, 127.81, 127.68, 127.52, 112.03 (t, J_{CF} = 252.5 Hz), 97.94, 92.85, 81.97, 79.78, 77.79, 75.61, 74.94, 73.36, 71.01, 70.15, 68.68, 68.36, 67.00, 63.08, 59.19, 57.28, 55.16, 13.90. HRMS (ESI) *m/z* [M + NH_4]$^+$ calculated for $C_{40}H_{52}NF_2O_{10}$ 760.3508, found 760.3510.

The synthesis of compound 4k

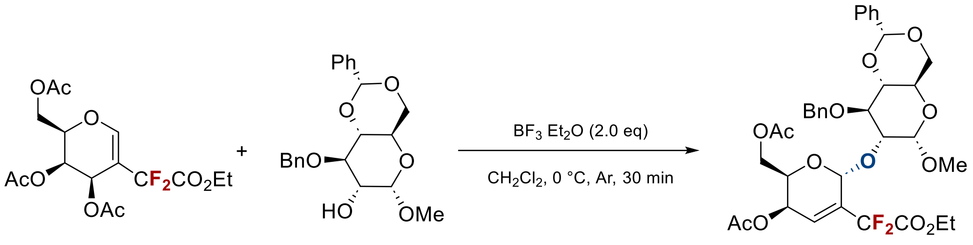

1l (2.0 eq) **4k**

According to the *General glycosylation procedure B*, reaction mixture was purified by column chromatography on silica gel (petroleum ether : ethyl acetate = 10 :1) to give **4k** as a colorless oil (67.1 mg, 95%). ^{1}H NMR (600 MHz, $CDCl_3$) δ 7.46-7.45 (m, 2H), 7.38-7.35 (m, 3H), 7.31-7.25 (m, 5H), 6.56 (dd, J = 5.6, 1.8 Hz, 1H), 5.56 (s, 1H), 5.30 (s, 1H), 5.07 (dd, J = 5.5, 2.8 Hz, 1H), 4.85-4.83 (m, 2H), 4.65 (d, J = 11.5 Hz, 1H), 4.37 (td, J = 6.5, 2.8 Hz, 1H), 4.34-4.28 (m, 3H), 4.13 (dd, J = 11.2, 6.5 Hz, 1H), 3.92-3.88 (m, 3H), 3.84 (td, J = 9.9, 4.7 Hz, 1H), 3.75 (t, J = 10.3 Hz, 1H), 3.63-3.59 (m, 1H), 3.43 (s, 3H), 2.06 (s, 3H), 1.96 (s, 3H), 1.34 (t, J = 7.2 Hz, 3H); ^{19}F NMR (376 MHz, $CDCl_3$) δ -107.9 (d, J = 263 Hz, 1F), -108.7 (d, J = 267 Hz, 1F); ^{13}C NMR (151 MHz, $CDCl_3$) δ 170.2, 170.0, 162.2 (t, J_{CF} = 33.8 Hz), 138.6, 137.3, 134.3 (t, J_{CF} = 23.9 Hz), 129.0, 128.2, 128.2, 128.0, 127.6, 127.2, (t, J_{CF} = 7.9 Hz), 126.0, 111.7 (t, J_{CF} = 251.8 Hz), 101.4, 97.5, 89.6 (t, J_{CF} = 3.8 Hz), 82.8, 75.1, 74.9, 69.1, 66.0, 63.2, 62.3, 61.5, 61.5, 55.4, 20.7, 20.6, 14.0. HRMS (ESI) m/z [M + NH_4]$^+$ calculated for $C_{35}H_{44}NF_2O_{13}$ 724.4782, found 724.4785.

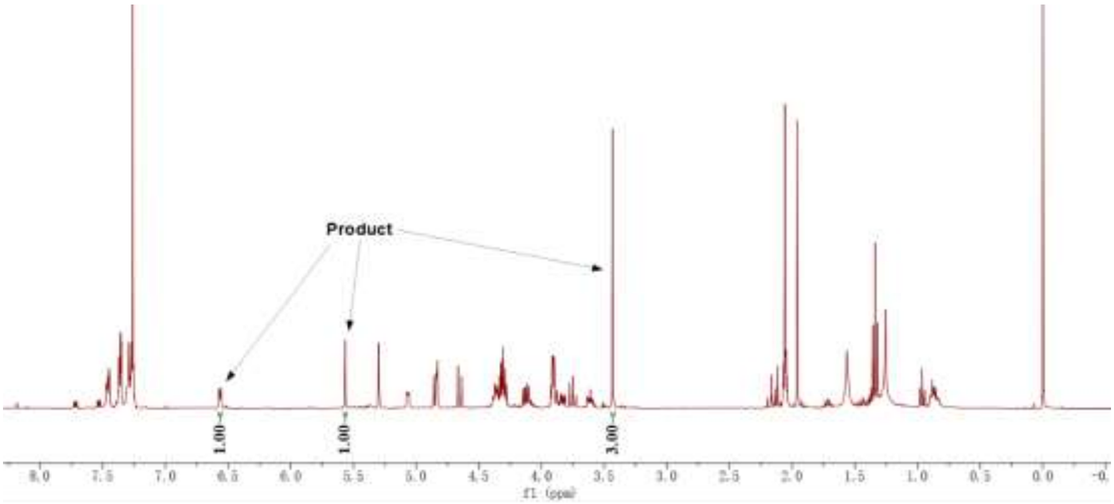

Crude product analysis of 4k

The synthesis of compound 4l

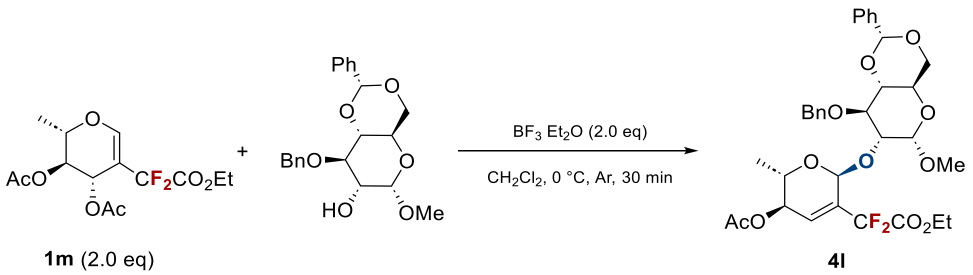

According to the *General glycosylation procedure A*, reaction mixture was purified by column chromatography on silica gel (petroleum ether : ethyl acetate = 10 :1) to give **4l** as a colorless oil (59.0 mg, 91%). ^{1}H NMR (600 MHz, $CDCl_3$) δ 7.48-7.46 (m, 2H), 7.40-7.37 (m, 5H), 7.31-7.28 (m, 2H), 7.27-7.24 (m, 1H), 6.35 (s, 1H), 5.61 (s, 1H), 5.58 (s, 1H), 5.32 (s, 1H), 5.16-5.13 (m, 1H), 4.96 (d, J = 10.5 Hz, 1H), 4.91 (d, J = 10.4 Hz, 1H), 4.69 (d, J = 3.8 Hz, 1H), 4.39-4.29 (m, 3H), 4.02 (t, J = 9.2 Hz, 1H), 3.99-3.94 (m, 1H), 3.91-3.84 (m, 2H), 3.77 (t, J = 10.3 Hz, 1H), 3.68 (t, J = 9.3 Hz, 1H), 3.42 (s, 3H), 2.16 (s, 3H), 1.37 (t, J = 7.2 Hz, 3H), 1.26 (d, J = 6.3 Hz, 3H); ^{19}F NMR (376 MHz, $CDCl_3$) δ -106.4 (d, J = 263 Hz, 1F), -109.1 (d, J = 262 Hz, 1F); ^{13}C NMR (151 MHz, $CDCl_3$) δ 170.2, 162.8 (dd, J_{CF} = 36.4, 32.0 Hz), 138.9, 137.3, 132.0 (t, J_{CF} = 25.7 Hz), 131.7 (t, J_{CF} = 7.6 Hz), 128.9, 128.2, 128.2, 128.1, 127.4, 126.0, 111.7 (t, J_{CF} = 251.6 Hz), 101.4, 100.2, 93.6 (d, J_{CF} = 4.5 Hz), 83.3, 77.7, 76.1, 74.4, 70.1, 69.1, 64.3, 63.1, 62.0, 55.2, 53.4, 29.7, 21.0, 17.6, 13.9. HRMS (ESI) m/z $[M + NH_4]^+$ calculated for $C_{33}H_{42}NF_2O_{11}$ 666.2720, found 666.2707.

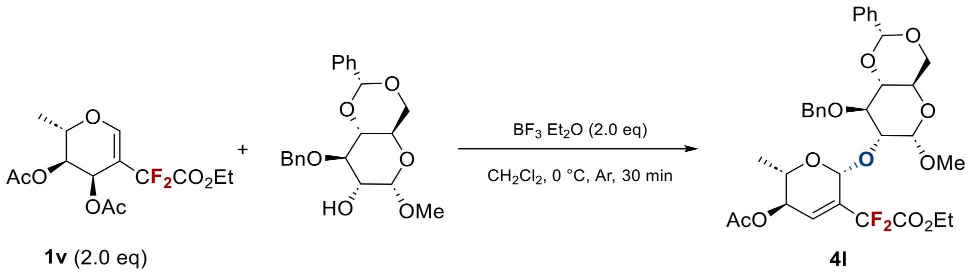

According to the *General glycosylation procedure A*, reaction mixture was purified by column chromatography on silica gel (petroleum ether : ethyl acetate = 4 :1) to give **4l** as a colorless oil (50.7 mg, 78%).

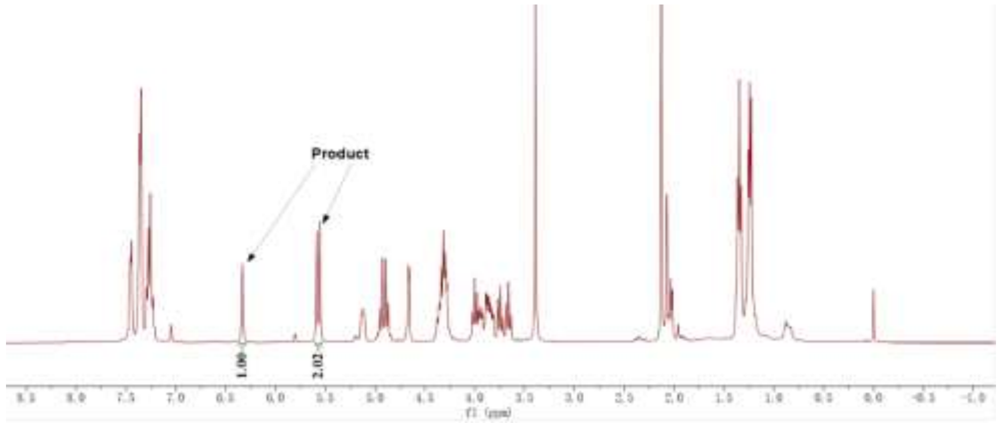

Crude product analysis of 4l

The synthesis of compound 4m

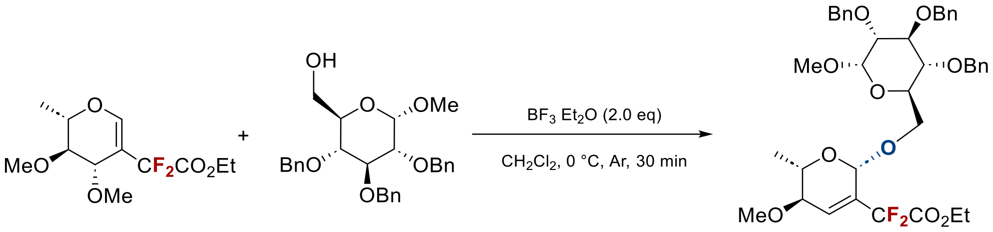

1o (2.0 eq) **4m**

According to the *General glycosylation procedure A*, reaction mixture was purified by column chromatography on silica gel (petroleum ether : ethyl acetate = 10 :1) to give **4m** as a colorless oil (54.2 mg, 76%). ^{1}H NMR (400 MHz, $CDCl_3$) δ 7.40-7.29 (m, 15H), 6.53 (s, 1H), 5.15 (s, 1H), 5.01 (d, J = 11.0 Hz, 1H), 4.92 (d, J = 11.0 Hz, 1H), 4.87-4.79 (m, 2H), 4.69 (d, J = 12.2 Hz, 1H), 4.62 (d, J = 11.0 Hz, 1H), 4.57 (d, J = 3.5 Hz, 1H), 4.24 (q, J = 7.1 Hz, 2H), 4.02 (t, J = 9.3 Hz, 1H), 3.92-3.83 (m, 2H), 3.79-3.69 (m, 2H), 3.60-3.47 (m, 3H), 3.45 (s, 3H), 3.41 (s, 3H), 1.30 (d, J = 6.3 Hz, 3H), 1.26 (t, J = 7.1 Hz, 3H); ^{19}F NMR (376 MHz, $CDCl_3$) δ -106.39 (d, J = 259 Hz, 1F), -107.34 (d, J = 259 Hz, 1F); ^{13}C NMR (101 MHz, $CDCl_3$) δ 163.22 (dd, J_{CF} = 34.8, 33.3 Hz), 139.18, 138.71, 138.52, 132.68 (t, J_{CF} = 7.7 Hz), 131.47 (t, J_{CF} = 23.7 Hz), 128.74, 128.69, 128.66, 128.45, 128.19, 128.06, 127.95, 127.82, 112.27 (t, J_{CF} = 248.9 Hz), 98.36, 93.34 (t, J_{CF} = 3.3 Hz), 82.27, 80.01, 78.19, 78.04, 75.92, 75.18, 73.63, 70.64, 67.51, 65.28, 63.28, 57.13, 55.55, 18.08, 14.18. HRMS (ESI) *m/z* [M + $NH_4]^+$ calculated for $C_{39}H_{50}NF_2O_{10}$ 730.3403, found 730.3395.

The synthesis of compound 4n

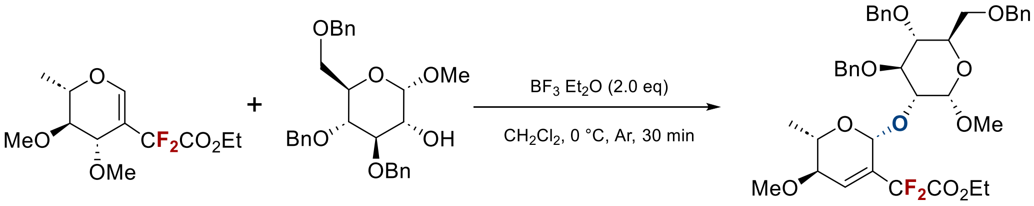

1o (2.0 eq) **4n**

According to the *General glycosylation procedure A*, reaction mixture was purified by column chromatography on silica gel (petroleum ether : ethyl acetate = 12 :1) to give **4n** as a colorless oil (55.6 mg, 78%). ^{1}H NMR (400 MHz, $CDCl_3$) δ 7.42-7.36 (m, 2H), 7.35-7.26 (m, 13H), 6.52 (s, 1H), 5.51 (s, 1H), 5.10 (d, J = 11.0 Hz, 1H), 4.85 (d, J = 11.0 Hz, 1H), 4.80-4.72 (m, 2H), 4.66 (d, J = 12.1 Hz, 1H), 4.50 (dd, J = 18.3, 11.5 Hz, 2H), 4.36-4.26 (m, 2H), 3.98-3.82 (m, 3H), 3.81-3.66 (m, 4H), 3.57-3.51 (m, 1H), 3.47 (s, 3H), 3.37 (s, 3H), 1.36-1.30 (m, 6H); ^{19}F NMR (376 MHz, $CDCl_3$) δ -105.78 (d, J = 259 Hz, 1F), -108.14 (d, J = 263 Hz, 1F); ^{13}C NMR (101 MHz, $CDCl_3$) δ 163.59 (t, J_{CF} = 34.3 Hz), 139.07, 138.58, 138.03, 132.39(t, J_{CF} = 7.6 Hz), 131.37 (t, J_{CF} = 24.2 Hz), 128.36, 128.29, 128.22, 127.87, 127.65, 127.63, 127.48, 112.21(t, J_{CF} = 251.5 Hz), 98.14, 93.22, 78.02, 77.94, 77.47, 74.66, 73.99, 73.44, 73.15, 69.25, 69.14, 65.26, 63.04, 56.90, 55.18, 17.86, 14.00. HRMS (ESI) m/z [M + NH_4] + calculated for $C_{39}H_{50}NF_2O_{10}$ 730.3403, found 730.3401.

The synthesis of compound 4o

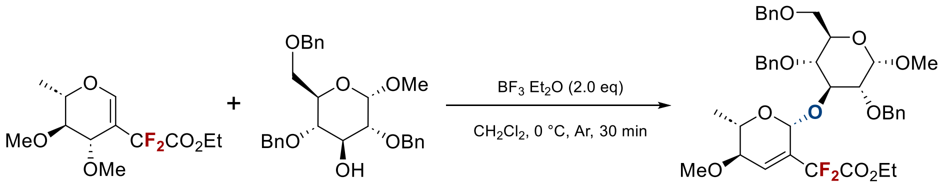

According to the *General glycosylation procedure A*, reaction mixture was purified by column chromatography on silica gel (petroleum ether : ethyl acetate = 12 :1) to give **4o** as a colorless oil (53.5 mg, 75%). ^{1}H NMR (400 MHz, $CDCl_3$) δ7.43-7.25 (m, 15H), 6.57 (s, 1H), 5.64 (s, 1H), 4.93 (dd, J = 11.9, 4.2 Hz, 2H), 4.58-4.47 (m, 4H), 4.40 (d, J = 11.8 Hz, 1H), 4.26 (dd, J = 10.2, 3.1 Hz, 1H), 4.23-4.06 (m, 2H), 3.99-3.87 (m, 3H), 3.72 (d, J = 2.5 Hz, 1H), 3.61-3.56 (m, 1H), 3.56-3.50 (m, 1H), 3.49 (s, 3H), 3.26 (s, 3H), 1.34 (d, J = 6.2 Hz, 3H), 1.23 (t, J = 7.1 Hz, 3H); ^{19}F NMR (376 MHz, $CDCl_3$) δ -105.78 (d, J = 259 Hz, 1F), -108.14 (d, J = 263 Hz, 1F); ^{13}C NMR (101 MHz, $CDCl_3$) δ 163.26 (t, J_{CF} = 34.3 Hz), 139.07, 138.58, 138.03, 132.39 (t, J_{CF} = 7.6 Hz), 131.37 (t, J_{CF} = 24.2 Hz), 128.36, 128.29, 128.22, 127.87, 127.65, 127.63, 127.48, 112.21 (t, J_{CF} = 253.0 Hz), 98.14, 93.22, 78.02, 77.94, 77.47, 74.66, 73.99, 73.44, 73.15, 69.25, 69.14, 65.26, 63.04, 56.90, 55.18, 17.86, 14.00. HRMS (ESI) m/z $[M + NH_4]^+$ calculated for $C_{39}H_{50}NF_2O_{10}$ 730.3403, found 730.3395.

The synthesis of compound 4p

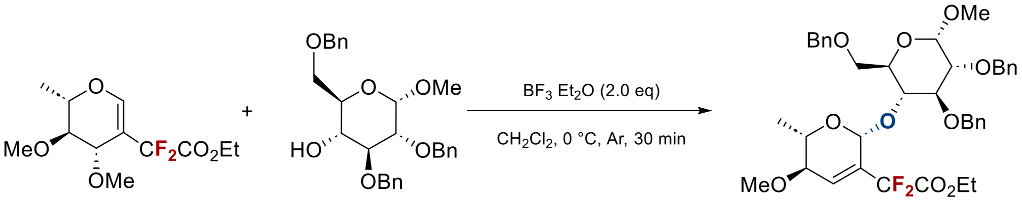

According to the *General glycosylation procedure A*, reaction mixture was purified by column chromatography on silica gel (petroleum ether : ethyl acetate = 10 :1) to give **4p** as a colorless oil (48.5 mg, 68%). ^{1}H NMR (400 MHz, $CDCl_3$) δ 7.40-7.27 (m, 15H), 6.53 (s, 1H), 5.14 (s, 1H), 5.02 (d, J = 10.9 Hz, 1H), 4.76 (d, J = 12.0 Hz, 1H), 4.68 (d, J = 10.9 Hz, 1H), 4.64-4.53 (m, 4H), 4.38-4.26 (m, 2H), 4.10 (t, J = 9.5 Hz, 1H), 3.88-3.78 (m, 2H), 3.72-3.58 (m, 4H), 3.49-3.43 (m, 1H), 3.38 (s, 3H), 3.35 (s, 3H), 1.37 (t, J = 7.1 Hz, 3H), 0.89 (d, J = 6.1 Hz, 3H); ^{19}F NMR (376 MHz, $CDCl_3$) δ -106.22 (d, J = 259 Hz, 1F), -109.67 (d, J = 259 Hz, 1F); ^{13}C NMR (101 MHz, $CDCl_3$) δ 163.17 (dd, J_{CF} = 32.3, 37.4 Hz), 138.87, 138.05, 137.88, 132.79 (t, J_{CF} = 8.1 Hz), 130.60(dd, J_{CF} = 22.2, 25.3 Hz), 128.47, 128.29, 128.26, 128.14, 127.98, 127.85, 127.69, 127.55, 127.27, 112.07 (t, J_{CF} = 251.0 Hz), 97.91, 90.91, 90.86, 80.46, 80.16, 77.56, 77.40, 77.08, 76.77, 75.90, 73.48, 73.28, 72.32, 69.68, 68.30, 65.09, 63.02, 56.57, 55.33, 17.41, 14.10. HRMS (ESI) m/z $[M + NH_4]^+$ calculated for $C_{39}H_{50}NF_2O_{10}$ 730.3403, found 730.3403.

The synthesis of compound 4q

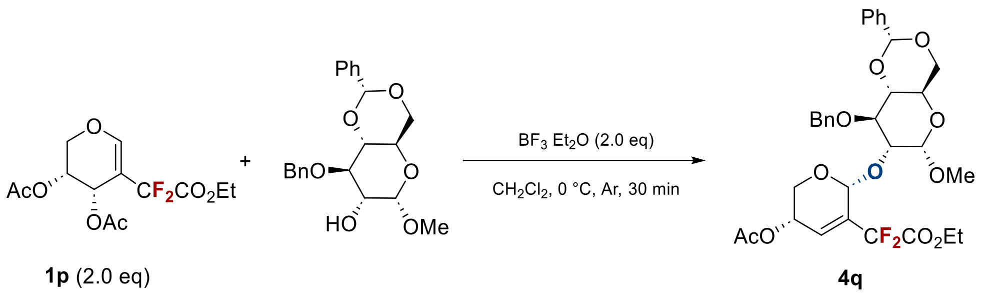

According to the *General glycosylation procedure A*, reaction mixture was purified by column chromatography on silica gel (petroleum ether : ethyl acetate = 10 :1) to give **4q** as a colorless oil (55.2 mg, 87%). ^{1}H NMR (600 MHz, $CDCl_3$) δ 7.49-7.46 (m, 2H), 7.41-7.37 (m, 5H), 7.32-7.29 (m, 2H), 7.27-7.24 (m, 1H), 6.55 (d, J = 5.6 Hz, 1H), 5.69 (s, 1H), 5.58 (s, 1H), 5.14 (ddd, J = 5.7, 3.0, 1.2 Hz, 1H), 4.95 (d, J = 10.6 Hz, 1H), 4.91 (d, J = 10.6 Hz, 1H), 4.75 (d, J = 3.8 Hz, 1H), 4.38-4.31 (m, 3H), 4.15 (dd, J = 13.0, 3.0 Hz, 1H), 4.02 (t, J = 9.2 Hz, 1H), 3.90-3.84 (m, 3H), 3.77 (t, J = 10.3 Hz, 1H), 3.69 (t, J = 9.3 Hz, 1H), 3.40 (s, 3H), 2.13 (s, 3H), 1.36 (t, J = 7.1 Hz, 3H); ^{19}F NMR (376 MHz, $CDCl_3$) δ -106.9 (d, J = 263 Hz, 1F), -109.7 (d, J = 264 Hz, 1F); ^{13}C NMR (101 MHz, $CDCl_3$) δ 170.4, 162.7 (dd, J_{CF} = 35.9, 32.1 Hz), 138.9, 137.3, 135.0 (t, J_{CF} = 24.1 Hz), 128.9, 128.2, 128.2, 128.1, 127.4, 126.5 (dd, J_{CF} = 9.5, 6.8 Hz), 126.0, 111.8 (t, J_{CF} = 252.1 Hz), 101.4, 100.0, 92.3 (d, J_{CF} = 4.2 Hz), 83.2, 77.6, 76.2, 74.4, 69.1, 63.2, 62.3, 62.0, 60.4, 55.1, 20.9, 13.9. HRMS (ESI) m/z $[M + NH_4]^+$ calculated for $C_{32}H_{40}NF_2O_{11}$ 652.2563, found 652.2551.

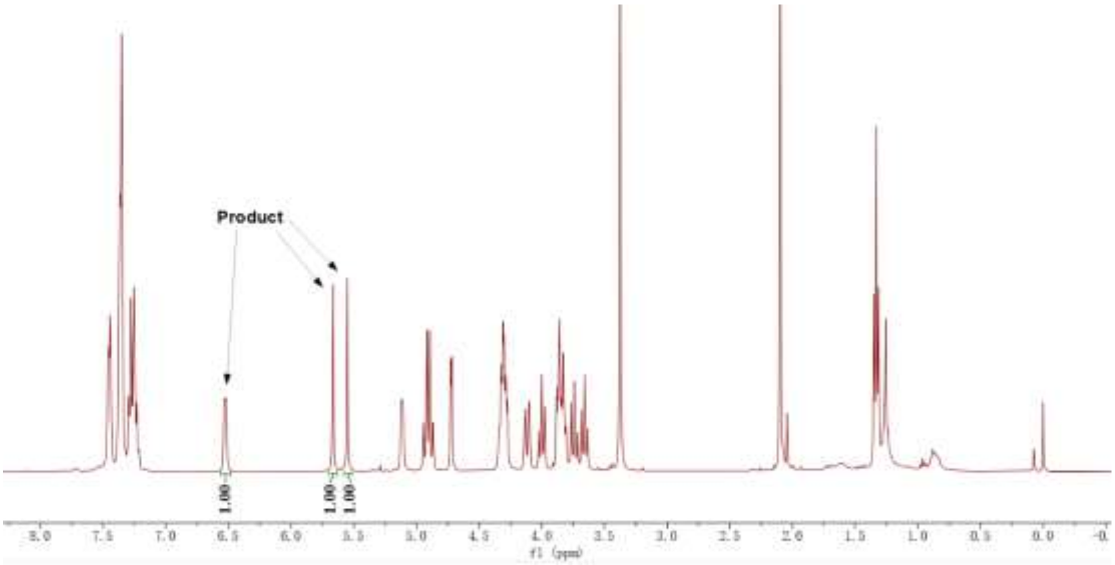

Crude product analysis of 4q

The synthesis of compound 4r

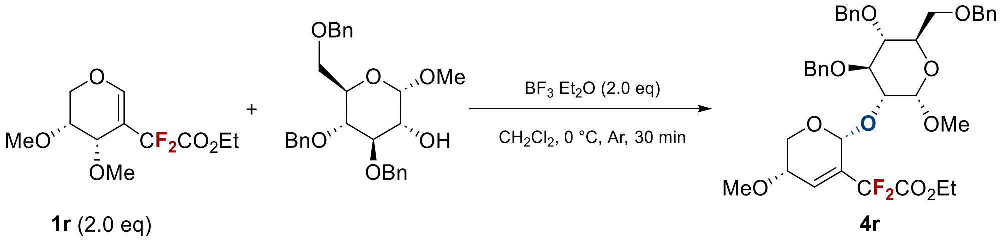

According to the *General glycosylation procedure A*, reaction mixture was purified by column chromatography on silica gel (petroleum ether : ethyl acetate = 10 :1) to give **4r** as a colorless oil (50.1 mg, 72%). ^{1}H NMR (400 MHz, $CDCl_3$) δ 7.42-7.29 (m, 15H), 6.58 (s, 1H), 5.26 (s, 1H), 5.02 (d, J = 11.0 Hz, 1H), 4.90 (d, J = 11.0 Hz, 1H), 4.83 (dd, J = 11.6, 7.3 Hz, 2H), 4.70 (d, J = 12.1 Hz, 1H), 4.62 (d, J = 11.0 Hz, 1H), 4.58 (d, J = 3.5 Hz, 1H), 4.27-4.20 (m, 2H), 4.04-3.98 (m, 2H), 3.92 (dd, J = 9.4, 3.8 Hz, 1H), 3.87 (dt, J = 9.5, 2.7 Hz, 1H), 3.80 (dd, J = 11.2, 1.6 Hz, 1H), 3.74-3.70 (m, 1H), 3.62-3.50 (m, 4H), 3.45 (s, 3H), 3.38 (s, 6H), 1.27 (t, J = 7.1 Hz, 3H); ^{19}F NMR (376 MHz, $CDCl_3$) δ -105.80 (d, J = 263 Hz, 1F), -107.86 (d, J = 263 Hz, 1F); ^{13}C NMR (101 MHz, $CDCl_3$) δ 162.74 (dd, J_{CF} = 32.3, 25.4 Hz), 138.88, 138.33, 138.20, 132.59 (t, J_{CF} = 8.1 Hz), 131.31 (t, J_{CF} = 24.2 Hz), 128.45, 128.43, 128.37, 128.19, 127.89, 127.87, 127.79, 127.72, 127.54, 111.79 (t, J_{CF} = 252.0 Hz), 92.70, 82.06, 79.73, 77.75, 75.61, 74.96, 73.37, 71.24, 70.09, 67.30, 62.95, 59.32, 56.87, 55.15, 13.89. HRMS (ESI) m/z $[M + NH_4]^+$ calculated for $C_{38}H_{48}NF_2O_{10}$ 716.3246, found 716.3244.

The synthesis of compound 4s

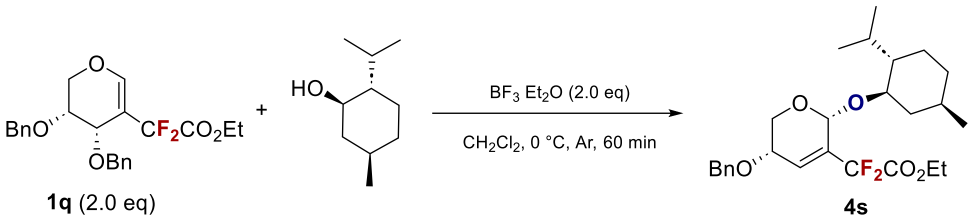

According to the *General glycosylation procedure A*, reaction mixture was purified by column chromatography on silica gel (petroleum ether : ethyl acetate = 10 :1) to give **4s** as a colorless oil (33.1 mg, 71%). ^{1}H NMR (400 MHz, $CDCl_3$) δ 7.37-7.34 (m, 4H), 7.32-7.28 (m, 1H), 6.55 (dd, J = 5.3, 2.6 Hz, 1H), 5.35 (s, 1H), 4.64 (d, J = 12.0 Hz, 1H), 4.61 (d, J = 11.9 Hz, 1H), 4.29-4.20 (m, 2H), 3.95 (d, J = 2.0 Hz, 2H), 3.84 (dd, J = 4.7, 2.3 Hz, 1H), 3.54 (td, J = 10.7, 4.1 Hz, 1H), 2.15-2.10 (m, 1H), 2.10-2.06 (m, 1H), 1.66-1.59 (m, 2H), 1.37-1.29 (m, 4H), 1.13-1.08 (m, 1H), 0.98 (td, J = 12.7, 3.2 Hz, 1H), 0.93 (d, J = 6.6 Hz, 3H), 0.86-0.79 (m, 5H), 0.76 (d, J = 7.0 Hz, 3H); ^{19}F NMR (376 MHz, $CDCl_3$) δ -108.69 (d, J = 259 Hz, 1F), -110.48 (d, J = 263 Hz, 1F); ^{13}C NMR (101 MHz, $CDCl_3$) δ 162.87 (dd, J_{CF} = 36.2, 32.5 Hz), 134.20 (dd, J_{CF} = 24.5, 22.1 Hz), 128.52, 127.90, 127.82, 127.60 (dd, J_{CF} = 9.0, 6.6 Hz), 112.04 (t, J_{CF} = 251.3 Hz), 87.33 (dd, J_{CF} = 5.0, 2.1 Hz), 75.04, 70.58, 66.36, 62.84, 60.81, 48.12, 38.49, 34.43, 31.42, 25.17, 22.87, 22.31, 21.04, 15.18, 13.86. HRMS (ESI) m/z $[M + NH_4]^+$ calculated for $C_{26}H_{40}NF_2O_5$ 484.2875, found 484.2872.

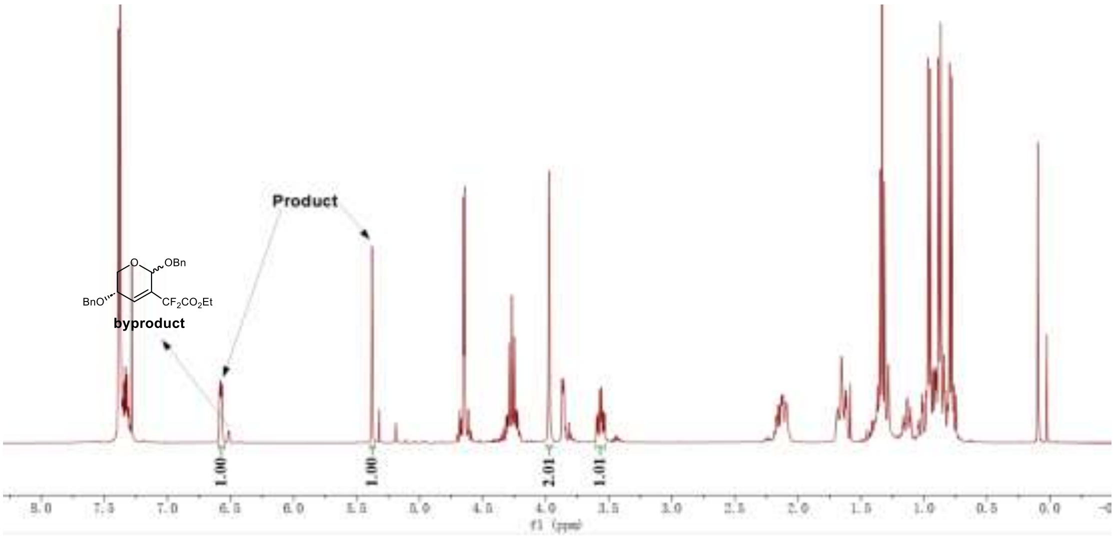

Crude product analysis of 4s

The synthesis of compound 4t

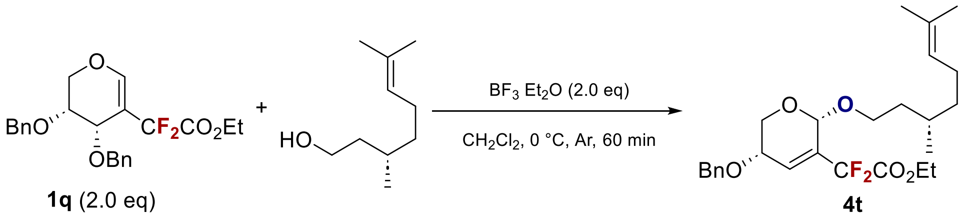

According to the *General glycosylation procedure A*, reaction mixture was purified by column chromatography on silica gel (petroleum ether : ethyl acetate = 10 :1) to give **4t** as a colorless oil (36.9 mg, 79%). ^{1}H NMR (400 MHz, $CDCl_3$) δ 7.36-7.34 (m, 4H), 7.33-7.28 (m, 1H), 6.53 (dd, J = 5.2, 2.9 Hz, 1H), 5.11 (s, 1H), 5.10-5.07 (m, 1H), 4.62 (m, 2H), 4.27 (q, J = 7.2 Hz, 2H), 4.00 (dd, J = 12.7, 3.1 Hz, 1H), 3.94 (d, J = 12.7 Hz, 1H), 3.84-3.81 (m, 1H), 3.76 (dt, J = 9.6, 6.9 Hz, 1H), 3.49-3.43 (m, 1H), 2.04-1.87 (m, 2H), 1.68 (s, 3H), 1.59 (s, 3H), 1.53-1.46 (m, 1H), 1.39-1.28 (m, 6H), 1.18-1.09 (m, 1H), 0.87 (d, J = 6.5 Hz, 3H); ^{19}F NMR (376 MHz, $CDCl_3$) δ -106.87 (d, J = 259 Hz, 1F), -111.10 (d, J = 259 Hz, 1F); ^{13}C NMR (101 MHz, $CDCl_3$) δ 162.99 (dd, J_{CF} = 35.7, 32.4 Hz), 137.78, 133.84 (dd, J_{CF} = 24.3, 22.5 Hz), 131.22, 128.52, 127.92, 127.82, 127.69 (dd, J_{CF} = 9.1, 2.2 Hz), 124.68, 111.91 (t, J_{CF} = 252.5, 250.4 Hz), 91.93 (dd, J_{CF} = 5.0, 2.3 Hz), 70.67, 67.38, 66.39, 62.96, 60.82, 36.94, 36.54, 29.38, 25.72, 25.41, 19.51, 17.62, 13.91. HRMS (ESI) m/z $[M + NH_4]^+$ calculated for $C_{26}H_{40}NF_2O_5$ 484.2875, found 484.2868.

The synthesis of compound 4u

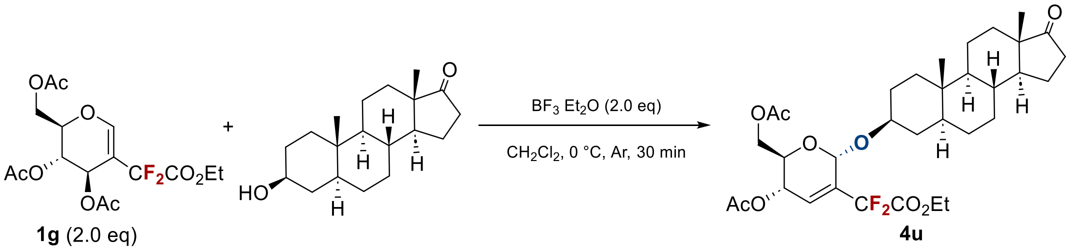

According to the *General glycosylation procedure B*, reaction mixture was purified by column chromatography on silica gel (petroleum ether : ethyl acetate = 6 :1) to give **4u** as

a colorless oil (53.1 mg, 85%). ^1H NMR (400 MHz, $CDCl_3$) δ 6.37 (s, 1H), 5.41 (m, 1H), 5.31 (s, 1H), 4.33 (m, 2H), 4.23 (s, 2H), 4.22-4.17 (m, 1H), 3.65-3.58 (m, 1H)2.52-2.38 (m, 1H), 2.14 (s, 3H), 2.12 (s, 3H), 1.89-1.78 (m, 4H), 1.74-1.62 (m, 4H), 1.60-1.52 (m, 10H), 1.43-1.21 (m, 16H), 1.17-1.09 (m, 16H), 1.01-1.0.94 (m, 2H), 0.88 (s, 3H), 0.83 (s, 3H), 0.73-0.67 (m, 1H); ^{19}F NMR (376 MHz, $CDCl_3$) δ -107.55 (d, J = 263 Hz, 1F), -109.28 (d, J = 259 Hz, 1F); ^{13}C NMR (101 MHz, $CDCl_3$) δ 221.25, 170.70, 170.06, 130.37 (t, J_{CF} = 8.6 Hz), 91.83, 78.98, 66.36, 64.63, 63.05, 62.67, 54.40, 51.38, 47.79, 44.94, 36.76, 36.13, 35.84, 35.67, 35.04, 31.53, 30.88, 29.70, 28.56, 27.39, 21.77, 20.88, 20.77, 20.45, 13.94, 13.82, 12.22. HRMS (ESI) m/z [M + NH_4]$^+$ calculated for $C_{33}H_{50}NF_2O_9$ 642.3454, found 642.3450.

The synthesis of compound 4v

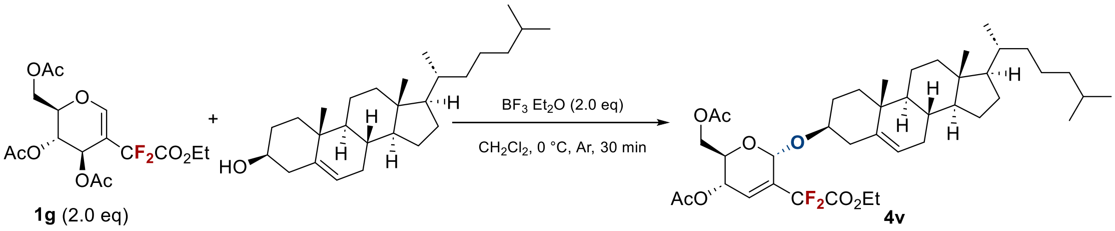

According to the *General glycosylation procedure B*, reaction mixture was purified by column chromatography on silica gel (petroleum ether : ethyl acetate = 9 :1) to give **4v** as a colorless oil (54.8 mg, 76%). ^1H NMR (400 MHz, $CDCl_3$) δ 6.37 (t, J = 2.1 Hz, 1H), 5.49-5.34 (m, 2H), 5.31 (s, 1H), 4.33 (m, 2H), 4.27-4.17 (m, 3H), 3.55 (dt, J = 11.3, 6.7 Hz, 1H), 2.40 (m, 1H), 2.34-2.22 (m, 1H), 2.14 (s, 3H), 2.11 (s, 3H), 2.07-1.92 (m, 2H), 1.92-1.80 (m, 3H), 1.64-1.43 (m, 10H), 1.37 (t, J = 7.1 Hz, 3H), 1.31-1.22 (m, 5H), 1.21-1.12 (m, 5H), 1.05-1.11 (m, 2H), 1.01 (s, 3H), 0.94 (d, J = 6.5 Hz, 3H), 0.90 (d, J = 1.7 Hz, 3H), 0.88 (d, J = 1.7 Hz, 3H), 0.70 (s, 3H); ^{19}F NMR (376 MHz, $CDCl_3$) δ -107.54 (d, J = 259 Hz, 1F), -109.36 (d, J = 259 Hz, 1F); ^{13}C NMR (101 MHz, $CDCl_3$) δ 170.75, 170.08, 140.51, 132.71 (t, J_{CF} = 22.3 Hz), 130.39 (t, J_{CF} = 8.1 Hz), 122.12, 111.58 (t, J_{CF} = 250.5 Hz), 91.62, 79.32, 66.42, 64.69, 63.07, 62.67, 56.72, 56.15, 50.12, 42.33, 40.32, 39.74, 39.52, 36.99, 36.64, 36.19, 35.80, 31.92, 31.88, 29.71, 28.23, 28.02, 27.56, 24.29, 23.83, 22.82, 22.57, 21.04, 20.89, 20.80, 19.35, 18.72, 13.96, 11.87. HRMS (ESI) m/z [M + NH_4]$^+$ calculated for $C_{41}H_{66}NF_2O_8$ 738.4757, found 738.4750.

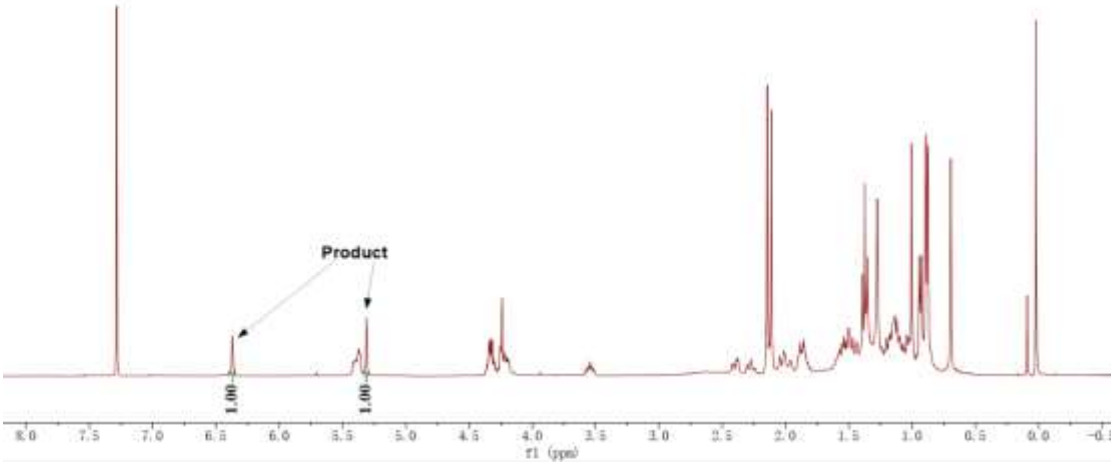

Crude product analysis of 4v

One pot synthesis of trisaccharide 5c

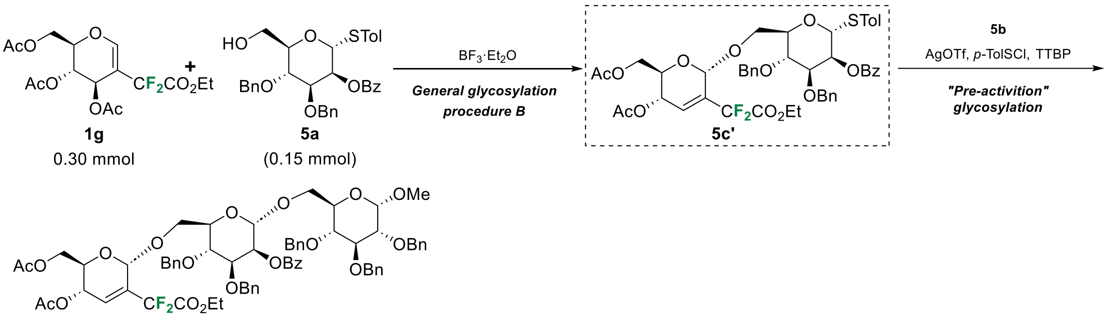

5c, 83%, two steps

Glycal donor **1g** (118 mg, 0.20 mmol) and acceptor **5a** (67.1 mg, 0.15 mmol) were conducted glycosylation according to the *General glycosylation procedure B*. After the reaction completion, the intermediate product **5c'** was obtained and dissolved in dry CH_2Cl_2 (3 mL). Then the reaction system temperature was downed to -78 ℃, followed by the addition of AgOTf (77.1 mg, 0.30 mmol), *p*-TolSCl (32.6 μL, 0.20 mmol) and TTBP (44.8 mg, 0.18 mmol) to "pre-activate" **5c'**. When the donor **5c'** was activated completely monitored by TLC, the acceptor **5b** (77.1 mg, 0.15 mmol) was added to this reaction system, and the reaction was stirred at -78 ℃ for 2 h. After completion, NEt_3 was added to quench reaction, and the reaction mixture was purified by column chromatography on silica gel (petroleum ether : ethyl acetate = 10 :1) to give **5c** as a colorless oil (115.0 mg, 83%, two steps). ^{1}H NMR (400 MHz, $CDCl_3$) δ 8.23-8.02 (m, 2H), 7.60 (t, *J* = 7.0, 1.2 Hz, 1H), 7.48 (t, *J* = 7.7 Hz, 2H), 7.44-7.40 (m, 2H), 7.39-7.15 (m, 25H), 6.37 (s, 1H), 5.70-5.58 (m, 1H), 5.47-5.36 (m, 1H), 5.29 (s, 1H), 5.07-4.94 (m, 3H), 4.91 (s, 1H), 4.86-4.69 (m, 3H), 4.67-4.45 (m, 4H), 4.20 (m, 2H), 4.16-3.99 (m, 5H), 3.96-3.89 (m, 1H), 3.88-3.73 (m, 5H), 3.70 (dd, *J* = 11.3, 1.4 Hz, 1H), 3.60 (dd, *J* = 9.6, 3.5 Hz, 1H), 3.54-3.46 (m, 1H), 3.35 (s, 3H), 2.08 (s, 3H), 1.97 (s, 3H), 1.18 (t, *J* = 7.1 Hz, 3H); ^{13}C NMR (101 MHz, $CDCl_3$) δ 170.58, 170.00, 165.72, 138.77, 138.55, 138.22, 138.17, 137.69, 133.19, 130.09, 129.84, 128.50, 128.43, 128.42, 128.36, 128.33, 128.23, 128.15, 128.06, 127.99, 127.97, 127.93, 127.90, 127.74, 127.71, 127.65, 127.63, 127.58, 127.41, 98.21, 97.84, 81.99, 80.15, 80.01, 77.74, 75.77, 75.06, 74.79, 74.28, 73.45, 73.37, 71.32, 71.03, 70.69, 69.96, 68.72, 67.84, 66.64, 66.46, 66.14, 64.40, 63.26, 62.17, 61.90, 55.22, 20.83, 20.63, 13.79. HRMS (ESI) m/z $[M + NH_4]^+$ calculated for $C_{69}H_{78}NF_2O_{19}$ 1262.5136, found 1262.5160.

5. X-Ray Crystallographic Data

Table S7 . X-ray crystallographic data of compound 1m

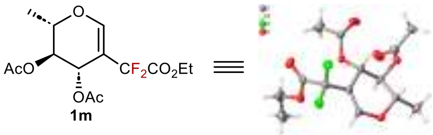

1m

Identification code	CCDC: 2408347
Empirical formula	$C_{14}H_{18}F_2O_7$
Formula weight	336.28
Temperature/K	100.00(10)
Crystal system	orthorhombic
Space group	$P2_12_12_1$
a/Å	8.23370(10)
b/Å	8.68780(10)
c/Å	22.0939(3)
α/°	90
β/°	90
γ/°	90
Volume/Å^3	1580.44(3)
Z	4
ρ_{calc}g/cm^3	1.413
μ/mm^{-1}	1.112
F(000)	704.0
Crystal size/mm^3	0.32 × 0.12 × 0.03
Radiation	Cu Kα (λ = 1.54184)
2Θ range for data collection/°	8.004 to 154.022
Index ranges	-10 ≤ h ≤ 7, -10 ≤ k ≤ 10, -27 ≤ l ≤ 27
Reflections collected	12232
Independent reflections	3218 [R_{int} = 0.0209, R_{sigma} = 0.0166]
Data/restraints/parameters	3218/2/217
Goodness-of-fit on F^2	1.046
Final R indexes [I>=2σ (I)]	R_1 = 0.0250, wR_2 = 0.0666
Final R indexes [all data]	R_1 = 0.0257, wR_2 = 0.0671
Largest diff. peak/hole / e Å^{-3}	0.20/-0.17
Flack parameter	0.00(4)

Table S8 . X-ray crystallographic data of compound 1p

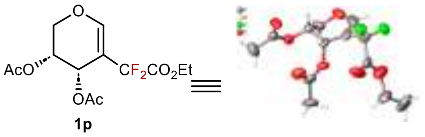

Identification code	CCDC: 2469576
Empirical formula	$C_{13}H_{16}F_2O_7$
Formula weight	322.26
Temperature/K	100.1(3)
Crystal system	monoclinic
Space group	P21
a/Å	10.4950(7)
b/Å	6.5169(4)
c/Å	11.6972(7)
α/°	90
β/°	111.643(7)
γ/°	90
Volume/Å3	743.63(9)
Z	2
ρcalcg/cm3	1.439
μ/mm-1	1.157
F(000)	336.0
Crystal size/mm3	0.08 × 0.03 × 0.02
Radiation	Cu Kα (λ = 1.54184)
2Θ range for data collection/°	8.132 to 154.382
Index ranges	-13 ≤ h ≤ 10, -7 ≤ k ≤ 7, -14 ≤ l ≤ 14
Reflections collected	7809
Independent reflections	2899 [Rint = 0.0625, Rsigma = 0.0493]
Data/restraints/parameters	2899/37/276
Goodness-of-fit on F2	1.097
Final R indexes [I>=2σ (I)]	R1 = 0.0865, wR2 = 0.2081
Final R indexes [all data]	R1 = 0.0975, wR2 = 0.2165
Largest diff. peak/hole / e Å-3	0.51/-0.37
Flack parameter	0.06(15)

Table S9 . X-ray crystallographic data of compound 1s

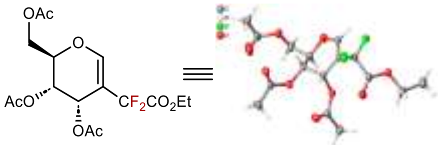

1s

Identification code	CCDC: 2469577
Empirical formula	$C_{16}H_{20}F_2O_9$
Formula weight	394.32
Temperature/K	100.01(13)
Crystal system	monoclinic
Space group	P21
a/Å	9.13272(8)
b/Å	6.63109(5)
c/Å	15.30758(12)
α/°	90
β/°	96.4625(7)
γ/°	90
Volume/Å3	921.135(13)
Z	2
ρcalcg/cm3	1.422
μ/mm-1	1.125
F(000)	412.0
Crystal size/mm3	0.25 × 0.15 × 0.1
Radiation	Cu Kα (λ = 1.54184)
2Θ range for data collection/°	5.81 to 154.958
Index ranges	-11 ≤ h ≤ 11, -8 ≤ k ≤ 8, -18 ≤ l ≤ 19
Reflections collected	26901
Independent reflections	3736 [Rint = 0.0445, Rsigma = 0.0185]
Data/restraints/parameters	3736/1/249
Goodness-of-fit on F2	1.069
Final R indexes [I>=2σ (I)]	R1 = 0.0289, wR2 = 0.0785
Final R indexes [all data]	R1 = 0.0293, wR2 = 0.0787
Largest diff. peak/hole / e Å-3	0.15/-0.15
Flack parameter	0.02(5)

Table S10 . X-ray crystallographic data of compound 4l

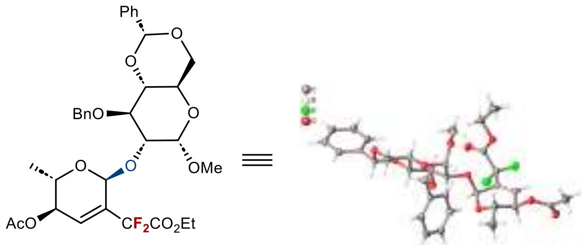

4l

Identification code	CDCC: 2469563
Empirical formula	$C_{33}H_{38}F_2O_{11}$
Formula weight	648.63
Temperature/K	100.00(10)
Crystal system	orthorhombic
Space group	P212121
a/Å	8.84700(10)
b/Å	17.1889(2)
c/Å	42.3975(3)
α/°	90
β/°	90
γ/°	90
Volume/Å3	6447.40(11)
Z	8
ρcalcg/cm3	1.336
μ/mm-1	0.910
F(000)	2736.0
Crystal size/mm3	0.21 × 0.13 × 0.03
Radiation	Cu Kα (λ = 1.54184)
2Θ range for data collection/°	4.168 to 149.64
Index ranges	-11 ≤ h ≤ 11, -21 ≤ k ≤ 21, -52 ≤ l ≤ 50
Reflections collected	72361
Independent reflections	12745 [Rint = 0.0651, Rsigma = 0.0331]
Data/restraints/parameters	12745/0/838
Goodness-of-fit on F2	1.039
Final R indexes [I>=2σ (I)]	R1 = 0.0375, wR2 = 0.0930
Final R indexes [all data]	R1 = 0.0399, wR2 = 0.0942
Largest diff. peak/hole / e Å-3	0.33/-0.22
Flack parameter	0.02(5)

Table S11 . X-ray crystallographic data of compound 4q

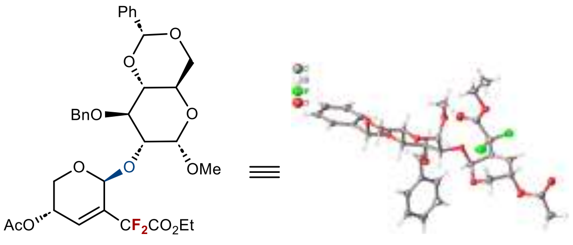

Identification code	CDCC: 2469571
Empirical formula	$C_{32}H_{36}F_2O_{11}$
Formula weight	634.61
Temperature/K	100.03(11)
Crystal system	orthorhombic
Space group	P212121
a/Å	9.07896(9)
b/Å	12.75487(10)
c/Å	26.3835(2)
α/°	90
β/°	90
γ/°	90
Volume/Å3	3055.23(5)
Z	4
ρcalcg/cm3	1.380
μ/mm-1	0.948
F(000)	1336.0
Crystal size/mm3	0.26 × 0.21 × 0.04
Radiation	Cu Kα (λ = 1.54184)
2Θ range for data collection/°	6.7 to 156.606
Index ranges	-10 ≤ h ≤ 11, -15 ≤ k ≤ 16, -32 ≤ l ≤ 33
Reflections collected	67707
Independent reflections	6204 [Rint = 0.0316, Rsigma = 0.0127]
Data/restraints/parameters	6204/2/414
Goodness-of-fit on F2	1.045
Final R indexes [I>=2σ (I)]	R1 = 0.0229, wR2 = 0.0582
Final R indexes [all data]	R1 = 0.0237, wR2 = 0.0586
Largest diff. peak/hole / e Å-3	0.17/-0.13
Flack parameter	-0.01(2)

6. Computational Methods

All data in this study were calculated with the Gaussian 16 Revision A.03 software package[4] and were optimized at the B3LYP level of density functional theory (DFT). [5] The basis set 6-31G* was selected for all atoms. Vibrational frequency analysis was computed to ensure the minimum have no imaginary frequency and the saddle points have only one imaginary frequency. To take the solvent effects into account, solvation-corrected single-point energy calculations were computed with the M06 functional in conjunction with the SMD[6] solvation model based on the gas-phase optimized geometries. In the M06-SMD calculations, a larger basis set 6-311++G(d, p) was employed for all non-metal atoms. Dichloromethane was used as the solvent. The relative free Gibbs energies (298 K, in kcal/mol) are used for the following discussion.

Coordinates for compound structures

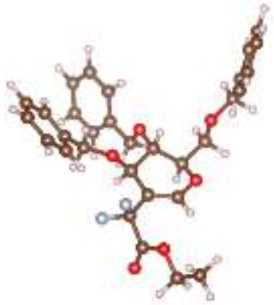

1a

C	0.550347000	-0.051949000	-0.781031000
C	0.638362000	-1.136242000	-1.867867000
O	-0.296005000	-2.203217000	-1.601389000
C	-1.534305000	-1.837966000	-1.210003000
C	-1.895128000	-0.614109000	-0.792073000
C	-0.880832000	0.495373000	-0.609540000
O	1.516520000	0.981788000	-0.957382000
C	2.014032000	-1.778742000	-1.971570000
O	-1.029610000	1.023997000	0.703012000
O	2.388690000	-2.270489000	-0.706664000
C	-0.893377000	2.439251000	0.810154000
C	1.507984000	1.718984000	-2.172244000
C	3.660180000	-2.896148000	-0.700935000
H	0.140794000	2.746035000	0.603985000
H	-1.541251000	2.919320000	0.057408000
H	-2.224534000	-2.672670000	-1.271584000
H	3.690467000	-3.670872000	-1.488527000
H	4.449424000	-2.165079000	-0.943290000
H	0.487865000	2.009846000	-2.461726000
H	-1.081864000	1.290533000	-1.341452000
H	0.825765000	-0.515810000	0.168663000
H	0.373761000	-0.719368000	-2.851084000
H	2.734690000	-1.028764000	-2.333680000

H	1.970729000	-2.594073000	-2.712567000
H	1.907260000	1.104219000	-2.996143000
C	-1.306524000	2.875811000	2.196266000
C	-0.617199000	3.908074000	2.841586000
C	-2.405771000	2.285685000	2.834435000
C	-1.023855000	4.357758000	4.099645000
H	0.245470000	4.361796000	2.358081000
C	-2.805660000	2.728632000	4.095205000
H	-2.931186000	1.473626000	2.342945000
C	-2.119855000	3.767506000	4.730450000
H	-0.478381000	5.160646000	4.589036000
H	-3.656590000	2.260924000	4.584144000
H	-2.434999000	4.110485000	5.712605000
C	2.360433000	2.961961000	-2.017090000
C	2.250851000	3.989746000	-2.962690000
C	3.279453000	3.094151000	-0.971008000
C	3.048512000	5.129727000	-2.868266000
H	1.533803000	3.900176000	-3.776843000
C	4.074516000	4.238953000	-0.873833000
H	3.359936000	2.302636000	-0.234307000
C	3.964236000	5.257922000	-1.820757000
H	2.949817000	5.920422000	-3.607370000
H	4.781751000	4.332478000	-0.053728000
H	4.583332000	6.147600000	-1.742520000
C	3.919489000	-3.519877000	0.651188000
C	5.229093000	-3.635248000	1.130539000
C	2.865220000	-4.028011000	1.419341000
C	5.485674000	-4.257577000	2.353349000
H	6.054196000	-3.232393000	0.546467000
C	3.120322000	-4.641396000	2.646495000
H	1.848539000	-3.924796000	1.054422000
C	4.430361000	-4.761822000	3.115714000
H	6.507713000	-4.338547000	2.714161000
H	2.293254000	-5.025144000	3.238217000
H	4.626908000	-5.239958000	4.071652000
C	-3.323235000	-0.299138000	-0.436756000
F	-3.588199000	-0.527782000	0.893789000
F	-3.553148000	1.032334000	-0.656974000
C	-4.378240000	-1.060725000	-1.275242000
O	-4.907837000	-0.599143000	-2.256346000
O	-4.599575000	-2.285600000	-0.773513000
C	-5.580694000	-3.087928000	-1.482685000
H	-6.527304000	-2.539603000	-1.494881000
H	-5.250372000	-3.206741000	-2.519533000

C	-5.694430000	-4.414255000	-0.756655000
H	-4.737226000	-4.945653000	-0.753233000
H	-6.010531000	-4.265802000	0.280495000
H	-6.436341000	-5.045545000	-1.257626000

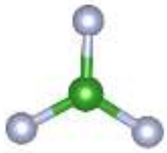

BF_3

B	0.000000000	0.000000000	0.000000000
F	0.000000000	1.318024000	0.000000000
F	-1.141442000	-0.659012000	0.000000000
F	1.141442000	-0.659012000	0.000000000

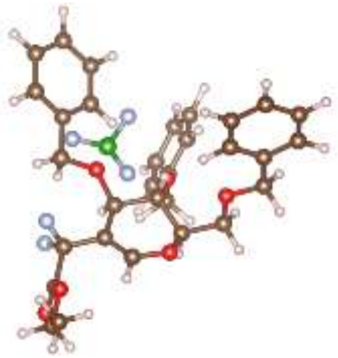

IM1

C	-0.270499000	0.224068000	-1.047011000
C	0.437904000	0.888738000	-2.240251000
O	1.841816000	1.096617000	-1.981681000
C	2.513794000	0.083191000	-1.415056000
C	1.962534000	-0.963038000	-0.778115000
C	0.476625000	-1.020462000	-0.526999000
O	-1.640995000	-0.057708000	-1.312718000
C	-0.143066000	2.259897000	-2.561035000
O	0.158947000	-1.135798000	0.898281000
O	-0.107445000	3.031489000	-1.387919000
C	-0.147241000	-2.459975000	1.448573000
C	-1.969316000	-0.848720000	-2.450557000
C	-0.544113000	4.365586000	-1.550256000
H	-0.295265000	-3.119654000	0.590108000
H	0.730946000	-2.796368000	1.999326000
H	3.584941000	0.208679000	-1.532292000
H	0.057828000	4.861534000	-2.332478000
H	-1.591868000	4.383146000	-1.898041000

H	-1.325143000	-1.737769000	-2.527173000
H	0.057658000	-1.922626000	-0.979477000
H	-0.299540000	0.957796000	-0.244594000
H	0.375776000	0.253398000	-3.134809000
H	-1.173715000	2.141530000	-2.933080000
H	0.458546000	2.722532000	-3.360294000
H	-1.817602000	-0.266570000	-3.373979000
C	-1.374237000	-2.412985000	2.318600000
C	-1.297628000	-2.814422000	3.655499000
C	-2.609114000	-2.012632000	1.790216000
C	-2.443732000	-2.838138000	4.453093000
H	-0.336437000	-3.100120000	4.074625000
C	-3.750814000	-2.027550000	2.587885000
H	-2.668872000	-1.666453000	0.762096000
C	-3.671815000	-2.446401000	3.919714000
H	-2.373540000	-3.152500000	5.490901000
H	-4.703007000	-1.713114000	2.169269000
H	-4.564303000	-2.460130000	4.539893000
C	-3.418047000	-1.277508000	-2.358182000
C	-3.838578000	-2.446549000	-3.003416000
C	-4.360105000	-0.498449000	-1.675289000
C	-5.179860000	-2.830623000	-2.973970000
H	-3.113125000	-3.063812000	-3.529894000
C	-5.700549000	-0.887710000	-1.639184000
H	-4.034658000	0.401528000	-1.164421000
C	-6.115213000	-2.051472000	-2.290021000
H	-5.491237000	-3.742451000	-3.476542000
H	-6.422171000	-0.277987000	-1.101783000
H	-7.159006000	-2.352157000	-2.260567000
C	-0.416269000	5.115073000	-0.240788000
C	-0.917655000	6.420603000	-0.155889000
C	0.198431000	4.543793000	0.878167000
C	-0.805416000	7.148840000	1.027487000
H	-1.401366000	6.870705000	-1.021420000
C	0.305515000	5.274652000	2.064800000
H	0.585057000	3.531919000	0.826604000
C	-0.191949000	6.575431000	2.144526000
H	-1.199619000	8.160566000	1.078947000
H	0.778960000	4.818326000	2.930033000
H	-0.106554000	7.138922000	3.069962000
C	2.800217000	-2.095081000	-0.263171000
F	3.000052000	-2.004507000	1.094212000
F	2.114177000	-3.271940000	-0.462033000
C	4.175859000	-2.276820000	-0.948193000

O	4.376646000	-3.075554000	-1.830095000
O	5.077783000	-1.432543000	-0.433121000
C	6.421988000	-1.529580000	-0.978728000
H	6.778902000	-2.552325000	-0.825555000
H	6.372946000	-1.350118000	-2.057388000
C	7.278385000	-0.506246000	-0.259209000
H	6.899948000	0.508112000	-0.420666000
H	7.296589000	-0.700259000	0.817526000
H	8.305078000	-0.555536000	-0.637802000
B	0.561555000	0.133048000	2.085750000
F	-0.652189000	0.665920000	2.344374000
F	1.126982000	-0.597767000	3.073413000
F	1.399404000	0.937285000	1.373678000

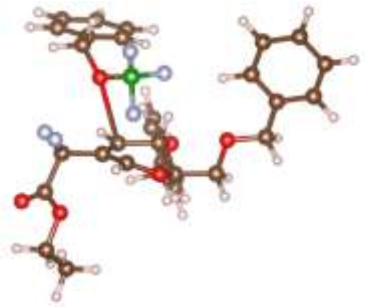

TS1

C	-0.044220000	-0.353774000	-0.933384000
C	-0.374589000	0.597440000	-2.095808000
O	-0.278656000	2.005203000	-1.661812000
C	0.569689000	2.340116000	-0.742201000
C	1.537580000	1.402102000	-0.218892000
C	1.236432000	0.092039000	-0.280862000
O	-0.038175000	-1.704138000	-1.350004000
C	-1.746389000	0.406633000	-2.714165000
O	0.435744000	1.101231000	2.368305000
O	-2.733476000	0.532203000	-1.731248000
C	0.651806000	0.568005000	3.658616000
C	1.008666000	-2.113573000	-2.235941000
C	-4.038483000	0.271412000	-2.228205000
H	1.564106000	1.019518000	4.072843000
H	-0.173723000	0.845551000	4.328329000
H	0.720728000	3.407720000	-0.660844000
H	-4.240540000	0.921824000	-3.096665000
H	-4.087165000	-0.769696000	-2.593497000
H	1.946588000	-1.601992000	-1.963136000
H	1.857519000	-0.646122000	0.216971000
H	-0.839149000	-0.278513000	-0.178989000

H	0.379330000	0.503098000	-2.887569000
H	-1.757598000	-0.593800000	-3.178636000
H	-1.881754000	1.150823000	-3.517707000
H	0.777343000	-1.828970000	-3.273434000
C	0.815722000	-0.943392000	3.637997000
C	1.889415000	-1.555702000	4.293709000
C	-0.118616000	-1.753886000	2.974964000
C	2.030152000	-2.946308000	4.299042000
H	2.626073000	-0.937643000	4.803206000
C	0.020316000	-3.142610000	2.976484000
H	-0.953545000	-1.284628000	2.463853000
C	1.095513000	-3.744698000	3.638940000
H	2.872292000	-3.402952000	4.813301000
H	-0.718881000	-3.757631000	2.468234000
H	1.200313000	-4.826881000	3.642801000
C	1.183264000	-3.610516000	-2.141195000
C	1.418897000	-4.364260000	-3.295838000
C	1.156769000	-4.253986000	-0.896727000
C	1.637891000	-5.740725000	-3.212818000
H	1.429352000	-3.873931000	-4.267212000
C	1.368007000	-5.629990000	-0.815085000
H	0.954685000	-3.679984000	0.002682000
C	1.612410000	-6.376565000	-1.970848000
H	1.818798000	-6.314775000	-4.117583000
H	1.341989000	-6.120011000	0.154425000
H	1.776961000	-7.448566000	-1.903398000
C	-5.080859000	0.503894000	-1.157138000
C	-6.433656000	0.476034000	-1.522273000
C	-4.733133000	0.727860000	0.178048000
C	-7.429789000	0.665269000	-0.565724000
H	-6.710670000	0.306982000	-2.561864000
C	-5.735288000	0.922452000	1.133402000
H	-3.691161000	0.756174000	0.474612000
C	-7.081329000	0.890685000	0.768924000
H	-8.475381000	0.641946000	-0.862252000
H	-5.452343000	1.101311000	2.167236000
H	-7.855513000	1.042901000	1.516529000
C	2.756864000	1.945689000	0.490481000
F	2.425492000	3.050606000	1.221144000
F	3.285321000	1.010839000	1.307926000
C	3.863766000	2.378861000	-0.507222000
O	4.966575000	1.898086000	-0.556512000
O	3.389331000	3.352815000	-1.300826000
C	4.325562000	3.897287000	-2.272987000

H	5.196294000	4.276509000	-1.730590000
H	4.661288000	3.081750000	-2.920805000
C	3.605538000	4.988195000	-3.040469000
H	2.733711000	4.589928000	-3.569785000
H	3.271245000	5.784974000	-2.368805000
H	4.284739000	5.424752000	-3.780390000
B	-0.832970000	1.653399000	2.018276000
F	-1.722521000	0.678527000	1.499183000
F	-1.444297000	2.404643000	2.989414000
F	-0.560581000	2.577186000	0.855285000

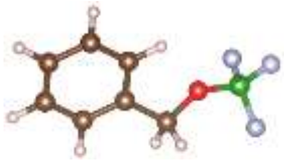

BnOBF$_3$

O	1.277542000	0.575045000	0.742059000
C	0.300940000	1.241570000	0.015854000
H	0.153634000	2.260080000	0.425910000
H	0.610554000	1.382827000	-1.033325000
C	-1.058796000	0.546114000	0.026992000
C	-2.248873000	1.284424000	0.014200000
C	-1.139172000	-0.854409000	0.023930000
C	-3.494273000	0.650528000	-0.009331000
H	-2.198495000	2.373061000	0.030797000
C	-2.381768000	-1.489369000	0.003111000
H	-0.210205000	-1.416841000	0.026283000
C	-3.565425000	-0.743874000	-0.013710000
H	-4.407065000	1.244645000	-0.013969000
H	-2.428971000	-2.577255000	-0.000746000
H	-4.532065000	-1.244505000	-0.025858000
B	2.347420000	-0.126877000	0.017812000
F	1.883672000	-1.386549000	-0.465075000
F	2.780847000	0.632578000	-1.093946000
F	3.399857000	-0.334682000	0.914478000

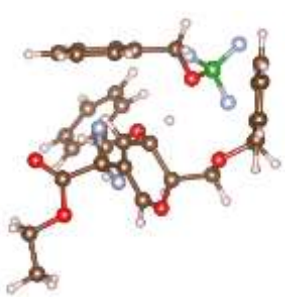

IM1’

C	-0.493386000	0.166338000	-0.825175000
C	-0.182047000	-0.101836000	-2.326866000
O	0.139199000	-1.527795000	-2.639889000
C	0.440002000	-2.359664000	-1.710343000
C	0.248150000	-2.147849000	-0.335446000
C	-0.384783000	-0.992235000	0.061695000
O	-1.640046000	0.959424000	-0.686828000
C	0.990225000	0.705488000	-2.848428000
O	1.076556000	2.166442000	0.453254000
O	2.121963000	0.355782000	-2.087573000
C	1.303628000	2.048779000	1.848213000
C	-2.910245000	0.320595000	-0.750304000
C	3.163848000	1.353082000	-2.161041000
H	2.275772000	1.561853000	2.006660000
H	1.366734000	3.049630000	2.292449000
H	0.775843000	-3.322357000	-2.087917000
H	3.595780000	1.325896000	-3.174653000
H	2.722094000	2.338476000	-1.993345000
H	-3.034134000	-0.348606000	0.116125000
H	-0.746959000	-0.878558000	1.081322000
H	0.326728000	0.849822000	-0.392740000
H	-1.068722000	0.091177000	-2.933920000
H	0.724278000	1.761822000	-2.715581000
H	1.136360000	0.495660000	-3.921309000
H	-2.988045000	-0.302783000	-1.658336000
C	0.220249000	1.240664000	2.542932000
C	0.529490000	0.062362000	3.234341000
C	-1.119886000	1.658442000	2.483834000
C	-0.473577000	-0.693518000	3.852646000
H	1.563008000	-0.274162000	3.278957000
C	-2.121319000	0.907231000	3.098464000
H	-1.355806000	2.567690000	1.941399000
C	-1.802890000	-0.274366000	3.781239000
H	-0.217116000	-1.612364000	4.372937000
H	-3.153840000	1.244230000	3.047498000
H	-2.585739000	-0.859159000	4.257625000
C	-3.990486000	1.381218000	-0.751753000
C	-5.318324000	0.995705000	-0.977931000
C	-3.691906000	2.728614000	-0.515243000
C	-6.342269000	1.941509000	-0.963838000
H	-5.554700000	-0.050654000	-1.166084000
C	-4.723113000	3.672781000	-0.506203000
H	-2.666039000	3.040970000	-0.346553000
C	-6.045269000	3.286515000	-0.727438000

H	-7.368539000	1.630238000	-1.140796000
H	-4.483220000	4.716951000	-0.325028000
H	-6.840792000	4.026926000	-0.718727000
C	4.218157000	1.058691000	-1.127298000
C	5.030531000	-0.077440000	-1.239245000
C	4.404109000	1.929969000	-0.046273000
C	6.012037000	-0.345250000	-0.285214000
H	4.890971000	-0.753737000	-2.079970000
C	5.394706000	1.666809000	0.903665000
H	3.766865000	2.804980000	0.051681000
C	6.197474000	0.530256000	0.788419000
H	6.637219000	-1.229431000	-0.382138000
H	5.537122000	2.352127000	1.735175000
H	6.967082000	0.327958000	1.529086000
C	0.575712000	-3.284413000	0.611527000
F	1.646471000	-3.981016000	0.109716000
F	0.905585000	-2.798033000	1.825670000
C	-0.593332000	-4.285233000	0.794341000
O	-1.195982000	-4.432149000	1.826331000
O	-0.825212000	-4.917206000	-0.363043000
C	-1.897348000	-5.905152000	-0.346598000
H	-1.656404000	-6.653080000	0.413985000
H	-2.820766000	-5.401730000	-0.045379000
C	-1.989057000	-6.503368000	-1.735830000
H	-2.224148000	-5.737228000	-2.481744000
H	-1.049596000	-6.989152000	-2.017332000
H	-2.784341000	-7.255832000	-1.757112000
B	0.765788000	3.525432000	-0.072168000
F	-0.480315000	3.971392000	0.408066000
F	1.781565000	4.402065000	0.314137000
F	0.722005000	3.395695000	-1.476311000

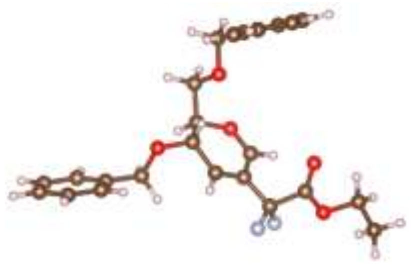

IM2

C	1.119177000	0.183159000	0.312128000
C	0.942831000	0.603515000	1.790991000
O	-0.221155000	-0.057815000	2.467091000
C	-0.813019000	-1.075938000	1.981230000

C	-0.478040000	-1.691255000	0.738019000
C	0.489595000	-1.130544000	-0.028295000
O	2.461591000	0.307199000	-0.084269000
C	0.706483000	2.096755000	1.935421000
O	-0.386397000	2.416380000	1.115297000
C	3.366065000	-0.732338000	0.367286000
C	-0.665012000	3.833329000	1.040369000
H	-1.597285000	-1.470603000	2.621369000
H	-0.926876000	4.192399000	2.045375000
H	0.245779000	4.356560000	0.716162000
H	2.939245000	-1.710779000	0.090456000
H	0.758613000	-1.576623000	-0.982963000
H	0.570516000	0.923159000	-0.294898000
H	1.789286000	0.286248000	2.404205000
H	1.631068000	2.608091000	1.619826000
H	0.522606000	2.344026000	2.992111000
H	3.464699000	-0.712820000	1.461056000
C	4.703558000	-0.517744000	-0.279534000
C	5.846804000	-0.321671000	0.503943000
C	4.821091000	-0.525953000	-1.677602000
C	7.093249000	-0.142432000	-0.097900000
H	5.764439000	-0.311255000	1.588400000
C	6.063136000	-0.339052000	-2.278928000
H	3.936742000	-0.670393000	-2.292651000
C	7.202069000	-0.149637000	-1.489371000
H	7.975040000	0.006118000	0.518229000
H	6.146614000	-0.344827000	-3.361746000
H	8.170920000	-0.008650000	-1.959503000
C	-1.792164000	4.053641000	0.070739000
C	-1.525103000	4.323722000	-1.277349000
C	-3.122879000	3.966119000	0.499460000
C	-2.570681000	4.504893000	-2.183467000
H	-0.494607000	4.404676000	-1.616349000
C	-4.170013000	4.145543000	-0.404355000
H	-3.338585000	3.764784000	1.546250000
C	-3.894484000	4.415263000	-1.747257000
H	-2.353317000	4.724964000	-3.224776000
H	-5.198655000	4.084990000	-0.060531000
H	-4.709439000	4.565041000	-2.449794000
C	-1.253852000	-2.930996000	0.338289000
F	-0.706755000	-3.480534000	-0.768456000
F	-1.172361000	-3.837345000	1.362518000
C	-2.755048000	-2.607576000	0.149855000
O	-3.262174000	-1.724957000	0.817118000

O	-3.335238000	-3.393889000	-0.723711000
C	-4.784878000	-3.220958000	-0.916330000
H	-5.263928000	-3.370025000	0.054786000
H	-4.952125000	-2.187828000	-1.232003000
C	-5.225277000	-4.232781000	-1.951561000
H	-4.714869000	-4.070226000	-2.905422000
H	-5.025701000	-5.254181000	-1.614695000
H	-6.302794000	-4.129659000	-2.116341000

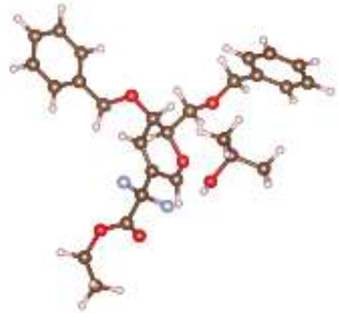

TS2

C	-0.109380000	1.014320000	-0.203240000
C	0.252202000	0.570850000	-1.636277000
O	0.266665000	-0.893530000	-1.810220000
C	-0.404585000	-1.717574000	-1.036859000
C	-1.244950000	-1.186142000	0.064543000
C	-1.132104000	0.100755000	0.418987000
O	-0.465252000	2.371459000	-0.157174000
C	1.605051000	1.092490000	-2.089462000
O	2.578594000	0.679067000	-1.157662000
C	-1.704294000	2.754496000	-0.773614000
C	3.895634000	1.200349000	-1.414010000
H	-0.796272000	-2.576944000	-1.578383000
H	4.216773000	0.904531000	-2.423963000
H	3.852441000	2.298809000	-1.379119000
H	-2.452339000	1.964380000	-0.634445000
H	-1.739564000	0.511039000	1.221473000
H	0.801145000	0.950262000	0.407774000
H	-0.513855000	0.918423000	-2.335937000
H	1.538591000	2.190305000	-2.135344000
H	1.826229000	0.714041000	-3.098798000
H	-1.558012000	2.866642000	-1.859825000
C	4.838440000	0.654719000	-0.374690000
C	5.750730000	-0.357240000	-0.697151000
C	4.792360000	1.132480000	0.941028000
C	6.604694000	-0.885225000	0.277424000
H	5.802622000	-0.729600000	-1.722725000

C	5.639251000	0.607932000	1.917818000
H	4.097194000	1.928804000	1.196787000
C	6.546938000	-0.403244000	1.586801000
H	7.319773000	-1.653884000	0.011930000
H	5.598907000	0.997113000	2.931479000
H	7.210422000	-0.802843000	2.343877000
C	-2.185823000	-2.138091000	0.765000000
F	-1.444334000	-3.251995000	1.184741000
F	-2.690202000	-1.562184000	1.875364000
C	-3.293832000	-2.703504000	-0.152799000
O	-2.999776000	-3.174845000	-1.233806000
O	-4.491499000	-2.601436000	0.389669000
C	-5.624579000	-3.150599000	-0.366195000
H	-6.467299000	-2.555908000	-0.018535000
H	-5.446074000	-2.956652000	-1.424449000
C	-5.799213000	-4.633005000	-0.076878000
H	-4.946337000	-5.207011000	-0.435913000
H	-5.925952000	-4.804156000	0.999200000
H	-6.694643000	-4.991848000	-0.587125000
C	3.146119000	-3.378718000	-0.057641000
H	4.156584000	-3.021267000	0.167529000
H	3.105000000	-3.659451000	-1.111165000
H	2.948332000	-4.267161000	0.554755000
C	2.154297000	-2.262310000	0.241756000
H	2.341016000	-1.396912000	-0.396028000
C	2.086996000	-1.858028000	1.703724000
H	3.059053000	-1.455388000	2.009993000
H	1.336545000	-1.080273000	1.880824000
H	1.862608000	-2.718282000	2.348645000
O	0.810235000	-2.746292000	-0.224544000
H	0.326174000	-3.202658000	0.492761000
C	-2.201075000	4.052119000	-0.178904000
C	-1.507109000	5.239519000	-0.468678000
C	-3.318142000	4.093771000	0.674252000
C	-1.922250000	6.450166000	0.078202000
H	-0.634849000	5.214762000	-1.126635000
C	-3.731239000	5.311146000	1.220919000
H	-3.860452000	3.180324000	0.903479000
C	-3.035001000	6.491902000	0.925656000
H	-1.374757000	7.365304000	-0.156741000
H	-4.601555000	5.333744000	1.876368000
H	-3.363172000	7.433980000	1.348943000

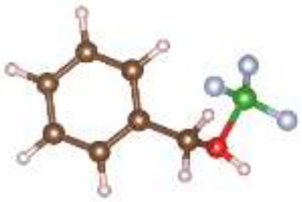

$BnOHBF_3$

O	1.321000000	1.128000000	0.185000000
C	0.275000000	1.314000000	-0.840000000
H	0.107000000	2.392000000	-0.906000000
H	0.672000000	0.940000000	-1.786000000
C	-0.976000000	0.594000000	-0.429000000
C	-1.991000000	1.279000000	0.252000000
C	-1.141000000	-0.767000000	-0.720000000
C	-3.158000000	0.616000000	0.634000000
H	-1.869000000	2.335000000	0.480000000
C	-2.306000000	-1.429000000	-0.336000000
H	-0.348000000	-1.302000000	-1.233000000
C	-3.315000000	-0.740000000	0.339000000
H	-3.941000000	1.157000000	1.158000000
H	-2.425000000	-2.485000000	-0.563000000
H	-4.224000000	-1.257000000	0.635000000
B	2.211000000	-0.364000000	0.231000000
F	1.603000000	-1.080000000	1.196000000
F	2.074000000	-0.847000000	-1.040000000
F	3.439000000	0.150000000	0.525000000
H	2.021000000	1.795000000	0.064000000

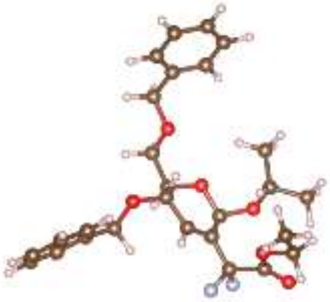

P1

C	-0.545029000	0.758977000	-0.255563000
C	0.038506000	0.705126000	-1.680646000
O	0.730946000	-0.524835000	-1.848204000
C	-0.041236000	-1.713678000	-1.787161000
C	-1.083345000	-1.641682000	-0.672210000
C	-1.296416000	-0.517614000	0.018422000
O	-1.340439000	1.918849000	-0.014652000
C	1.020776000	1.831494000	-1.965149000

O	2.051982000	1.807560000	-0.998924000
C	-2.552968000	2.036891000	-0.752814000
C	2.972950000	2.881988000	-1.149828000
H	-0.564592000	-1.862076000	-2.743433000
H	3.401453000	2.854732000	-2.166347000
H	2.450853000	3.846293000	-1.041893000
H	-3.044558000	1.054417000	-0.834929000
H	-2.058759000	-0.481327000	0.792346000
H	0.293014000	0.866317000	0.441043000
H	-0.779579000	0.777461000	-2.416421000
H	0.477218000	2.789371000	-1.929981000
H	1.433294000	1.704843000	-2.978974000
H	-2.354940000	2.380404000	-1.779706000
C	-3.477291000	3.017729000	-0.063822000
C	-4.447189000	3.696549000	-0.811656000
C	-3.413629000	3.230467000	1.317632000
C	-5.346703000	4.563605000	-0.190613000
H	-4.498600000	3.547361000	-1.888544000
C	-4.307905000	4.104297000	1.938727000
H	-2.650894000	2.719640000	1.895401000
C	-5.278948000	4.770672000	1.188876000
H	-6.093789000	5.083196000	-0.784914000
H	-4.244356000	4.265252000	3.011907000
H	-5.974302000	5.450376000	1.674173000
C	4.064018000	2.756719000	-0.115280000
C	4.231003000	3.730475000	0.874322000
C	4.930908000	1.655304000	-0.136787000
C	5.249477000	3.614344000	1.823681000
H	3.558958000	4.585140000	0.903585000
C	5.943824000	1.532707000	0.812988000
H	4.803386000	0.891140000	-0.899013000
C	6.107258000	2.514509000	1.794917000
H	5.368659000	4.380296000	2.585428000
H	6.609357000	0.673839000	0.786047000
H	6.899867000	2.421055000	2.532654000
C	-1.972297000	-2.846752000	-0.472689000
F	-3.150777000	-2.467628000	0.133769000
F	-2.292750000	-3.377623000	-1.684712000
C	-1.427914000	-3.995708000	0.410188000
O	-1.420198000	-5.155763000	0.080538000
O	-1.037159000	-3.513021000	1.596801000
C	-0.621446000	-4.498907000	2.578673000
H	-1.435058000	-5.218845000	2.709227000
H	0.244382000	-5.037806000	2.182800000

C	-0.298240000	-3.755599000	3.860094000
H	0.510129000	-3.034519000	3.702415000
H	-1.175344000	-3.216861000	4.232186000
H	0.020686000	-4.468293000	4.628364000
C	3.026707000	-2.092889000	-0.848623000
H	3.753371000	-2.291839000	-0.051009000
H	2.828120000	-1.021425000	-0.887442000
H	3.470929000	-2.402688000	-1.801671000
C	1.748560000	-2.895766000	-0.591577000
H	1.250757000	-2.521207000	0.313905000
C	2.049681000	-4.386170000	-0.440723000
H	2.718023000	-4.554790000	0.411496000
H	1.135207000	-4.967693000	-0.297862000
H	2.547831000	-4.759101000	-1.342823000
O	0.821412000	-2.806282000	-1.703034000

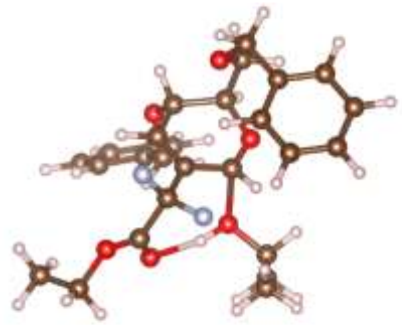

TS3

C	-0.643131000	-1.514061000	-1.417251000
C	0.181071000	-2.313103000	-0.383969000
O	0.814428000	-1.496287000	0.677489000
C	0.999946000	-0.228079000	0.562897000
C	0.576322000	0.521252000	-0.608778000
C	-0.189404000	-0.081542000	-1.534034000
O	-2.043996000	-1.613385000	-1.253187000
C	1.331441000	-3.085433000	-1.023790000
O	2.011183000	-2.212970000	-1.901813000
C	-2.629170000	-1.002657000	-0.077891000
C	3.433348000	-2.421782000	-2.007304000
H	1.760930000	0.159955000	1.226176000
H	3.653744000	-3.493464000	-2.094983000
H	3.707008000	-1.944474000	-2.952501000
H	-2.531887000	0.088852000	-0.146158000
H	-0.536871000	0.463582000	-2.408537000
H	-0.462154000	-1.984183000	-2.389736000
H	-0.472135000	-2.993222000	0.165815000
H	0.910812000	-3.945044000	-1.569278000

H	1.985957000	-3.467442000	-0.230106000
H	-2.094927000	-1.329394000	0.822987000
C	-4.074531000	-1.411355000	-0.019707000
C	-5.034498000	-0.722630000	-0.772539000
C	-4.475142000	-2.501654000	0.761873000
C	-6.371547000	-1.116946000	-0.745014000
H	-4.732079000	0.125226000	-1.383013000
C	-5.812694000	-2.897318000	0.794201000
H	-3.737482000	-3.042174000	1.351277000
C	-6.762146000	-2.205184000	0.039628000
H	-7.108766000	-0.575469000	-1.330790000
H	-6.113598000	-3.741771000	1.407491000
H	-7.804269000	-2.510434000	0.065092000
C	4.178924000	-1.801391000	-0.847668000
C	4.153672000	-0.407313000	-0.674990000
C	4.878198000	-2.584955000	0.076293000
C	4.808141000	0.186168000	0.404571000
H	3.634075000	0.213490000	-1.401695000
C	5.539992000	-1.992261000	1.156526000
H	4.918922000	-3.664094000	-0.054256000
C	5.502932000	-0.607904000	1.324164000
H	4.791900000	1.266742000	0.518205000
H	6.085538000	-2.612522000	1.861834000
H	6.023315000	-0.146220000	2.158480000
C	1.028939000	1.961817000	-0.761629000
F	2.049736000	2.217611000	0.124545000
F	1.507604000	2.149613000	-2.013504000
C	-0.075458000	3.007749000	-0.444161000
O	-0.869750000	2.831889000	0.467930000
O	0.004015000	4.056201000	-1.223003000
C	-0.933353000	5.169075000	-0.990135000
H	-1.100153000	5.249239000	0.085393000
H	-0.378377000	6.037201000	-1.346591000
C	-2.221943000	4.952501000	-1.761486000
H	-2.023695000	4.824262000	-2.829763000
H	-2.766897000	4.080778000	-1.387239000
H	-2.863264000	5.831565000	-1.636836000
C	-1.271257000	0.613813000	4.177294000
H	-0.993723000	0.648180000	5.236437000
H	-1.815315000	-0.316770000	3.992306000
H	-1.942264000	1.456475000	3.975008000
C	-0.021006000	0.692666000	3.305975000
H	0.616416000	-0.176131000	3.505134000
C	0.770219000	1.983890000	3.497014000

H	1.135520000	2.052575000	4.526513000
H	1.634880000	2.035900000	2.826900000
H	0.137666000	2.859155000	3.307159000
O	-0.418151000	0.521931000	1.908735000
H	-0.723929000	1.385409000	1.540161000

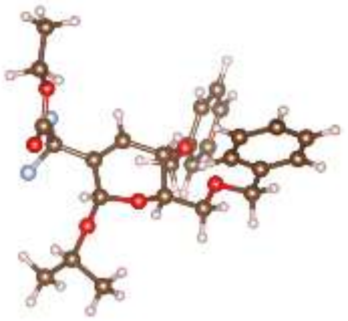

P2

C	0.014727000	-0.829735000	0.137542000
C	-0.338201000	-0.466547000	-1.316663000
O	-0.409973000	0.958762000	-1.436550000
C	0.840731000	1.600709000	-1.266352000
C	1.514014000	1.157840000	0.022285000
C	1.136351000	0.040660000	0.648402000
O	0.271358000	-2.221964000	0.315888000
C	-1.670121000	-1.045862000	-1.762706000
O	-2.685872000	-0.613820000	-0.881486000
C	1.454986000	-2.737810000	-0.286865000
C	-3.967783000	-1.114915000	-1.210527000
H	0.602438000	2.670271000	-1.229005000
H	-4.184764000	-0.901696000	-2.273079000
H	-3.996710000	-2.211547000	-1.095079000
H	2.287962000	-2.030779000	-0.146113000
H	1.608209000	-0.263278000	1.579787000
H	-0.875135000	-0.647170000	0.751286000
H	0.440859000	-0.841188000	-1.994197000
H	-1.594438000	-2.145273000	-1.762340000
H	-1.883361000	-0.714549000	-2.791739000
H	1.324066000	-2.859866000	-1.372564000
C	1.799602000	-4.074302000	0.333445000
C	2.535000000	-5.010979000	-0.403113000
C	1.436195000	-4.380663000	1.649649000
C	2.912348000	-6.227359000	0.166948000
H	2.813507000	-4.787970000	-1.431310000
C	1.805689000	-5.600950000	2.218031000
H	0.851051000	-3.663719000	2.215522000
C	2.547381000	-6.526549000	1.481301000

H	3.483051000	-6.943939000	-0.418045000
H	1.512009000	-5.829055000	3.239521000
H	2.834371000	-7.475966000	1.925551000
C	-5.011880000	-0.465457000	-0.330894000
C	-6.177532000	-1.160496000	0.009906000
C	-4.848405000	0.851329000	0.116225000
C	-7.171388000	-0.548966000	0.776133000
H	-6.308249000	-2.188370000	-0.322629000
C	-5.837744000	1.460133000	0.889421000
H	-3.937014000	1.383407000	-0.136831000
C	-7.003379000	0.764365000	1.218941000
H	-8.071059000	-1.101564000	1.034320000
H	-5.698277000	2.481377000	1.235016000
H	-7.773169000	1.240626000	1.820482000
C	2.574948000	2.064167000	0.599052000
F	3.364719000	1.387777000	1.482709000
F	3.382313000	2.562930000	-0.383710000
C	1.925303000	3.281032000	1.300931000
O	1.489780000	4.224039000	0.678982000
O	1.863402000	3.118449000	2.623044000
C	1.201306000	4.181917000	3.359158000
H	1.733854000	5.117931000	3.165645000
H	0.183747000	4.288225000	2.970596000
C	1.222918000	3.798980000	4.825906000
H	0.692301000	2.856319000	4.992648000
H	2.250370000	3.686542000	5.185846000
H	0.732935000	4.580150000	5.417161000
C	0.569873000	1.849429000	-4.408771000
H	0.551573000	2.536647000	-5.263080000
H	-0.402372000	1.887261000	-3.909729000
H	0.722112000	0.832557000	-4.789754000
C	1.696310000	2.225212000	-3.444882000
H	1.526840000	3.244719000	-3.066239000
C	3.077915000	2.155812000	-4.086041000
H	3.143929000	2.843833000	-4.936105000
H	3.845778000	2.421685000	-3.353635000
H	3.281304000	1.141043000	-4.446825000
O	1.739308000	1.333409000	-2.312985000

TS4

C	0.203645000	-1.538493000	-0.193112000
C	-0.165033000	-1.837761000	-1.654040000
O	-0.024347000	-0.611183000	-2.482420000

C	0.915504000	0.231125000	-2.225684000
C	1.778146000	0.189341000	-1.109037000
C	1.407874000	-0.652134000	-0.091897000
O	0.246067000	-2.716909000	0.581876000
C	-1.588672000	-2.329900000	-1.834717000
O	-2.453348000	-1.448226000	-1.170861000
C	1.310797000	-3.661386000	0.329553000
C	-3.822854000	-1.893318000	-1.181021000
H	0.973260000	1.027292000	-2.965092000
H	-4.187579000	-1.914020000	-2.218027000
H	-3.862199000	-2.920827000	-0.788547000
H	1.308209000	-3.992997000	-0.717117000
H	1.902238000	-0.597691000	0.871419000
H	-0.607022000	-0.933203000	0.242535000
H	0.531762000	-2.545824000	-2.114483000
H	-1.642048000	-3.348253000	-1.416292000
H	-1.812361000	-2.397037000	-2.911912000
H	1.025163000	-4.519831000	0.943203000
C	-4.655621000	-0.966995000	-0.337128000
C	-5.443394000	0.026761000	-0.929913000
C	-4.648054000	-1.089072000	1.059372000
C	-6.215548000	0.883151000	-0.142362000
H	-5.460221000	0.124587000	-2.012899000
C	-5.415838000	-0.233319000	1.848668000
H	-4.045841000	-1.864855000	1.526814000
C	-6.202557000	0.753679000	1.247629000
H	-6.830739000	1.644310000	-0.613682000
H	-5.412220000	-0.343623000	2.929522000
H	-6.810477000	1.412803000	1.861061000
C	2.899385000	1.193867000	-0.993027000
C	2.395511000	2.638058000	-0.991731000
O	1.220627000	2.944039000	-1.126262000
O	3.402560000	3.487472000	-0.856253000
C	3.099278000	4.921622000	-0.840375000
H	3.993984000	5.377566000	-1.267174000
H	2.246123000	5.098053000	-1.497832000
C	2.838281000	5.400001000	0.576773000
H	1.937589000	4.936454000	0.990872000
H	3.687030000	5.175594000	1.229972000
H	2.689334000	6.485164000	0.570464000
C	-2.528022000	2.436954000	0.845219000
H	-3.327365000	2.784924000	1.508206000
H	-2.933549000	1.649919000	0.202881000
H	-2.223955000	3.283146000	0.214296000

C	-1.339453000	1.911114000	1.648037000
H	-1.668446000	1.064847000	2.261869000
C	-0.708410000	2.971891000	2.549717000
H	-1.431273000	3.347104000	3.282595000
H	0.148986000	2.559382000	3.091883000
H	-0.363333000	3.828765000	1.955387000
O	-0.350878000	1.342400000	0.765354000
H	-0.059023000	2.041107000	0.151011000
C	2.677888000	-3.149473000	0.716137000
C	2.950978000	-2.825407000	2.057125000
C	3.690834000	-2.992208000	-0.241173000
C	4.205298000	-2.345691000	2.425330000
H	2.173734000	-2.953087000	2.806363000
C	4.952829000	-2.517489000	0.128431000
H	3.498623000	-3.261818000	-1.277690000
C	5.209165000	-2.189872000	1.460140000
H	4.409756000	-2.105568000	3.464687000
H	5.733672000	-2.414173000	-0.619565000
H	6.190407000	-1.826974000	1.752101000
H	3.620002000	1.089594000	-1.813900000
H	3.470591000	1.018161000	-0.075888000

TS5

C	0.107324000	-0.240348000	-2.102105000
C	0.639550000	-0.033642000	-0.673248000
O	0.063424000	1.207745000	-0.059413000
C	-1.115726000	1.594459000	-0.337251000
C	-1.906922000	1.124125000	-1.440856000
C	-1.316602000	0.217481000	-2.258343000
O	0.310801000	-1.551987000	-2.567062000
C	2.142964000	0.131767000	-0.606584000
O	2.499937000	1.152986000	-1.509623000
C	-0.402573000	-2.621257000	-1.897238000
C	3.919407000	1.334436000	-1.660116000
H	-1.464534000	2.401256000	0.304610000
H	4.378005000	0.386223000	-1.976442000
H	4.007474000	2.045466000	-2.487122000
H	-0.670807000	-3.308352000	-2.706377000
H	-1.837888000	-0.159720000	-3.137037000
H	0.708746000	0.389813000	-2.776546000
H	0.292013000	-0.814502000	0.006038000
H	2.594583000	-0.834659000	-0.877973000
H	2.441720000	0.371788000	0.422786000

H	-1.334051000	-2.244538000	-1.453446000
C	0.431665000	-3.328892000	-0.852351000
C	1.725977000	-3.770613000	-1.164764000
C	-0.092874000	-3.590967000	0.419306000
C	2.482966000	-4.458007000	-0.216277000
H	2.134805000	-3.576913000	-2.153019000
C	0.663366000	-4.288060000	1.366105000
H	-1.092743000	-3.244026000	0.669190000
C	1.952034000	-4.720301000	1.050567000
H	3.482865000	-4.800105000	-0.467985000
H	0.244549000	-4.493974000	2.347209000
H	2.540090000	-5.262961000	1.785038000
C	4.593800000	1.859378000	-0.412052000
C	4.152234000	3.053660000	0.176580000
C	5.668367000	1.174024000	0.165067000
C	4.777229000	3.552189000	1.318214000
H	3.317341000	3.591148000	-0.266667000
C	6.301815000	1.676167000	1.304830000
H	6.019466000	0.247219000	-0.283657000
C	5.855952000	2.864662000	1.883504000
H	4.431581000	4.481079000	1.763530000
H	7.139777000	1.138283000	1.738999000
H	6.347405000	3.257360000	2.769087000
C	-3.268872000	1.725858000	-1.702162000
C	-4.227263000	1.642250000	-0.517424000
O	-3.889038000	1.305531000	0.608026000
O	-5.443183000	2.020805000	-0.870932000
C	-6.491802000	2.036966000	0.155761000
H	-6.037923000	2.338576000	1.101519000
H	-7.173064000	2.815305000	-0.190686000
C	-7.173426000	0.683850000	0.252314000
H	-7.581208000	0.379263000	-0.716305000
H	-6.479428000	-0.084352000	0.606413000
H	-8.001737000	0.747910000	0.966117000
C	-2.776970000	-1.536178000	3.316652000
H	-2.568941000	-1.746456000	4.371411000
H	-2.931338000	-2.488674000	2.799334000
H	-3.712255000	-0.963271000	3.269092000
C	-1.630746000	-0.751232000	2.679667000
H	-0.714387000	-1.350573000	2.724082000
C	-1.387737000	0.595020000	3.363982000
H	-1.147658000	0.457520000	4.423860000
H	-0.551648000	1.125376000	2.894818000
H	-2.282265000	1.227764000	3.302807000

O	-1.868167000	-0.575341000	1.265086000
H	-2.715544000	-0.101580000	1.166629000
H	-3.177370000	2.788038000	-1.970055000
H	-3.734649000	1.246581000	-2.568569000

7. References

[1] M. C. Belhomme, T. Poisson, X. Pannecoucke, *Org. Lett.* **2013**, *15*, 3428.

[2] B. Wang, D. C. Xiong, X. S. Ye, *Org. Lett.* **2015**, *17*, 5698.

[3] S. K. Madhusudan, G. Agnihotri, D. S. Negi, A. K. Misra, *Carbohydr. Res.* **2005**, *340*, 1373.

[4] Frisch, M. J.; Trucks, G. W.; Schlegel, H. B.; Scuseria, G. E.; Robb, M. A.; Cheeseman, J. R.; Scalmani, G.; Barone, V.; Petersson, G. A.; Nakatsuji, H.; Li, X.; Caricato, M.; Marenich, A. V.; Bloino, J.; Janesko, B. G.; Gomperts, R.; Mennucci, B.; Hratchian, H. P.; Ortiz, J. V.; Izmaylov, A. F.; Sonnenberg, J. L.; Williams-Young, D.; Ding, F.; Lipparini, F.; Egidi, F.; Goings, J.; Peng, B.; Petrone, A.; Henderson, T.; Ranasinghe, D.; Zakrzewski, V. G.; Gao, J.; Rega, N.; Zheng, G.; Liang, W.; Hada, M.; Ehara, M.; Toyota, K.; Fukuda, R.; Hasegawa, J.; Ishida, M.; Nakajima, T.; Honda, Y.; Kitao, O.; Nakai, H.; Vreven, T.; Throssell, K.; Montgomery, J. A. Jr.; Peralta, J. E.; Ogliaro, F.; Bearpark, M. J.; Heyd, J. J.; Brothers, E. N.; Kudin, K. N.; Staroverov, V. N.; Keith, T. A.; Kobayashi, R.; Normand, J.; Raghavachari, K.; Rendell, A. P.; Burant, J. C.; Iyengar, S. S.; Tomasi, J.; Cossi, M.; Millam, J. M.; Klene, M.; Adamo, C.; Cammi, R.; Ochterski, J. W.; Martin, R. L.; Morokuma, K.; Farkas, O.; Foresman, J. B.; Fox, D. J. Gaussian, Inc., Wallingford CT, **2016**.

[5] (a) Becke, A. D. Density-Functional Thermochemistry. III. The Role of Exact Exchange. *J. Chem. Phys.* **1993**, *98*, 5648. (b) Lee, C.; Yang, W.; Parr, R. G. Development of the Colle-Salvetti Correlation-Energy Formula into a Functional of the Electron Density. *Phys. Rev. B: Condens. Matter Mater. Phys.* **1988**, *37*, 785. (c) Becke, A. D. Density-Functional Exchange-Energy Approximation with Correct Asymptotic Behavior. *Phys. Rev. A: At., Mol., Opt. Phys.* **1988**, *38*, 3098.

[6] A.-V. Marenich, C.-J. Cramer and D.-G. Truhlar, *J. Phys. Chem. B* **2009**, *113*, 6378-6396.

8. NMR Spectra

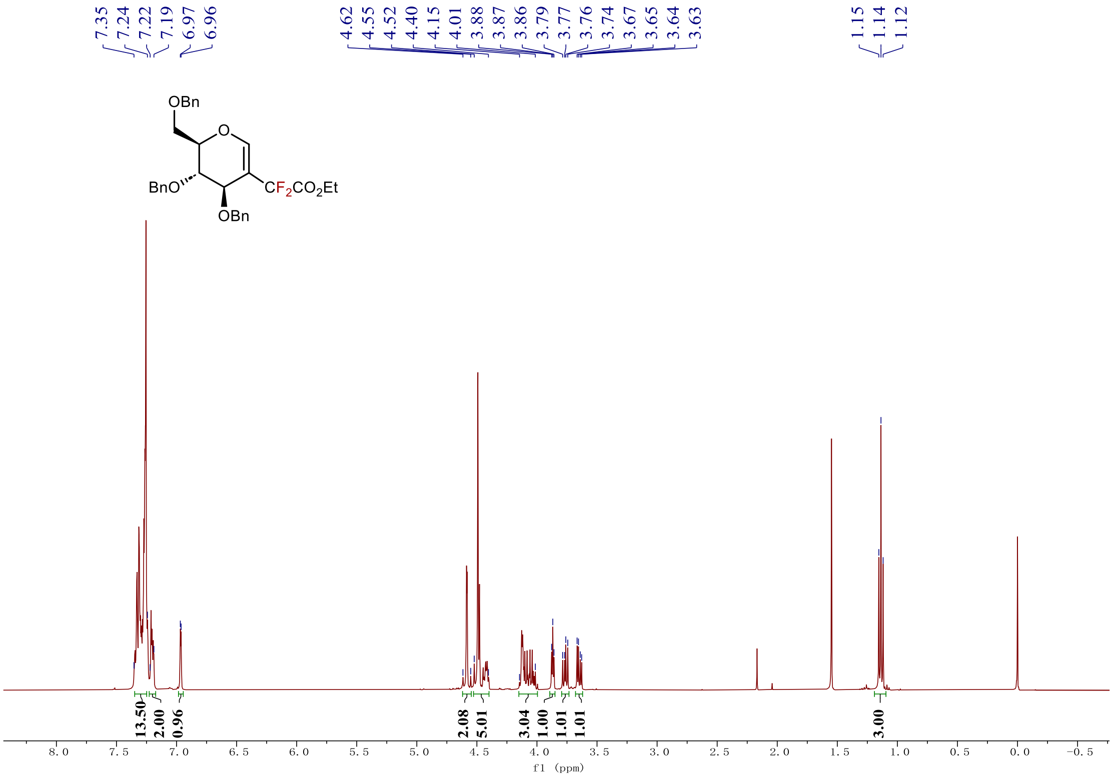

^{1}H NMR spectrum of Compound **1a**

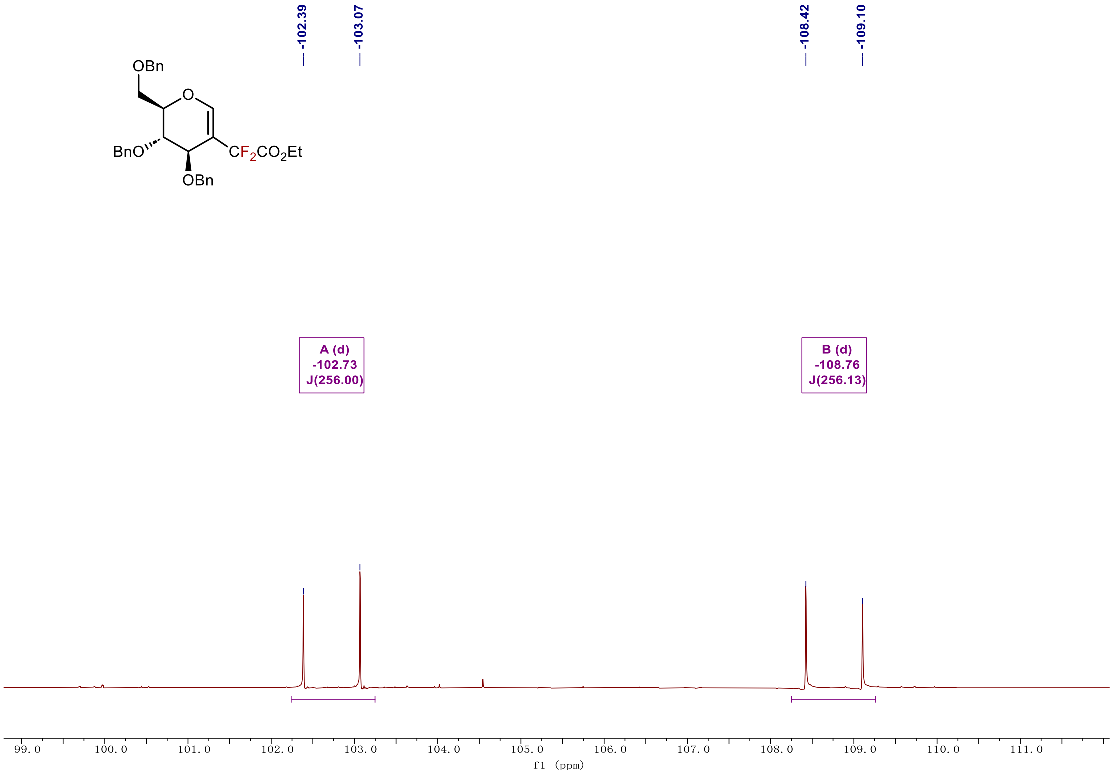

^{19}F NMR spectrum of Compound **1a**

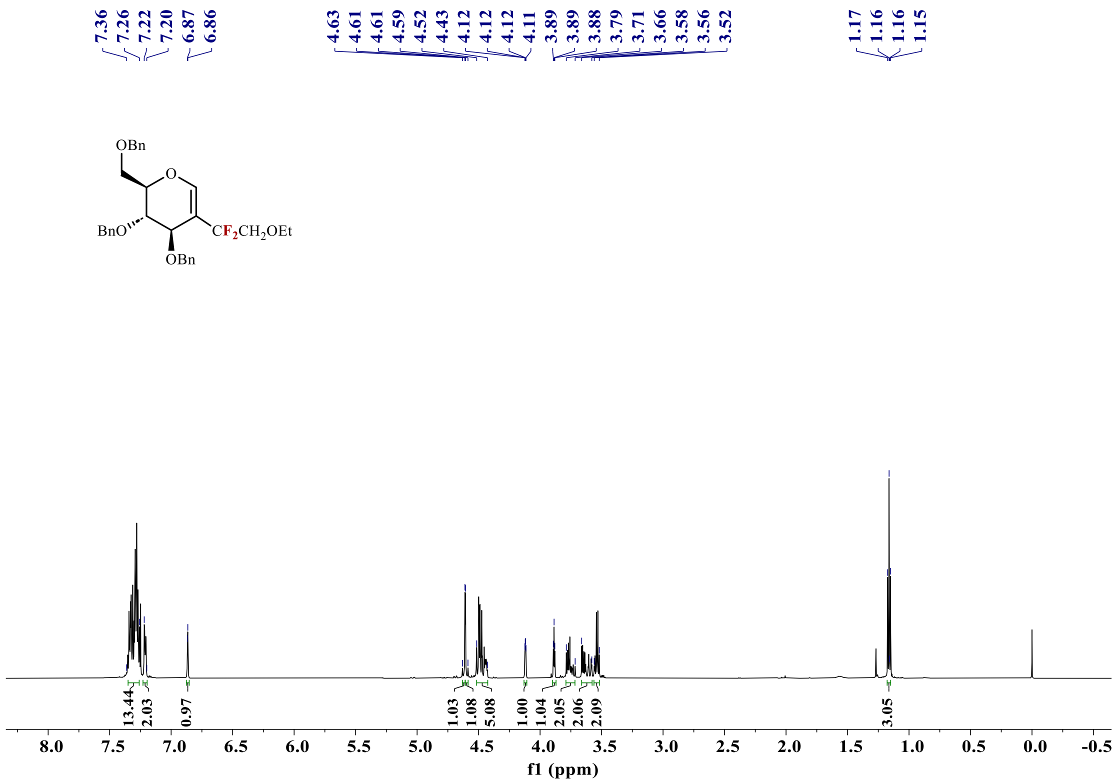

^{1}H NMR spectrum of Compound **1b**

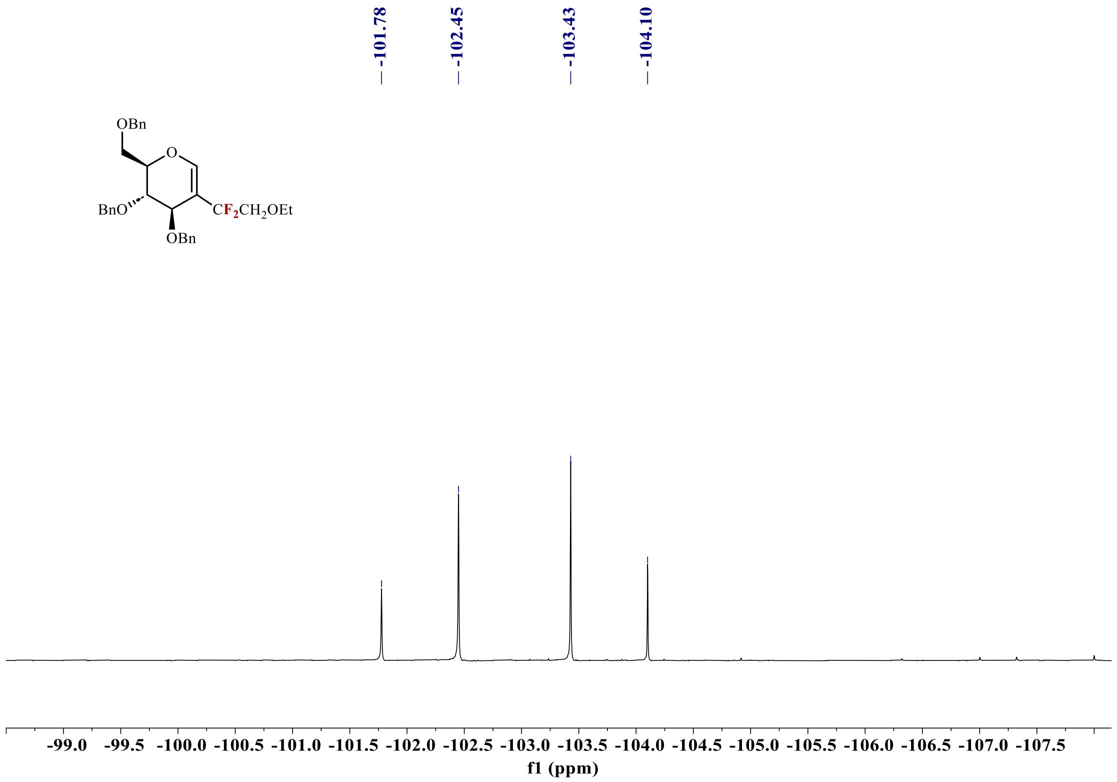

^{19}F NMR spectrum of Compound **1b**

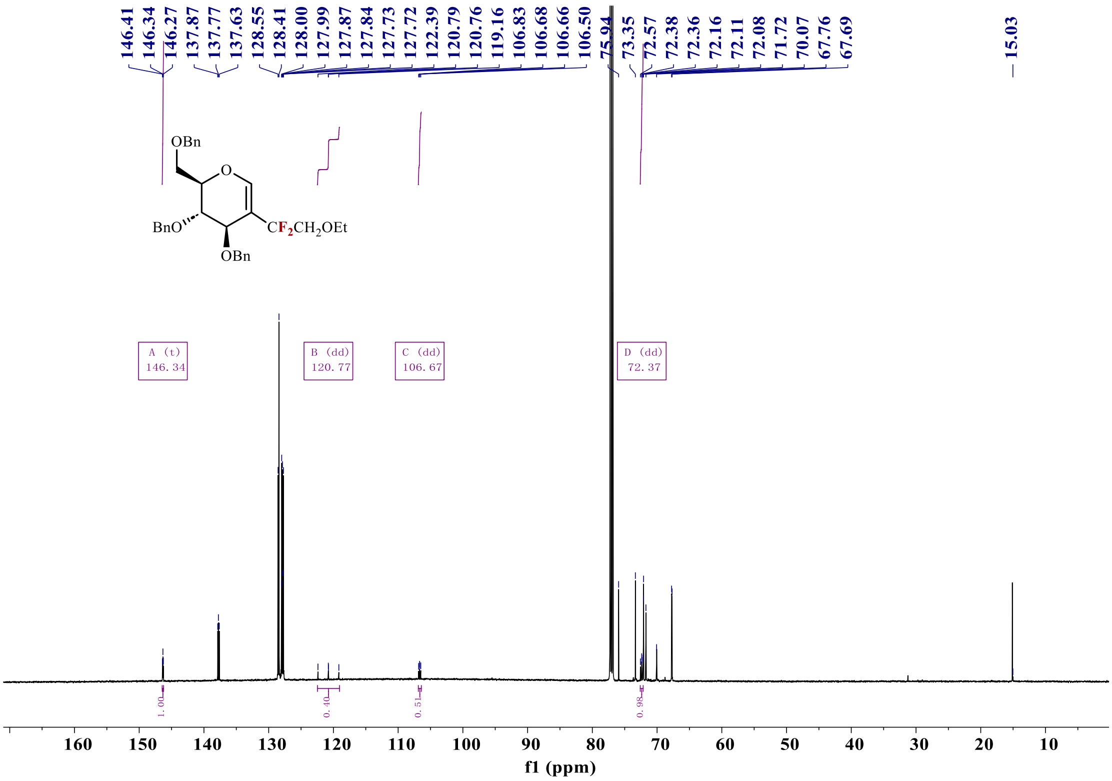

^{13}C NMR spectrum of Compound **1b**

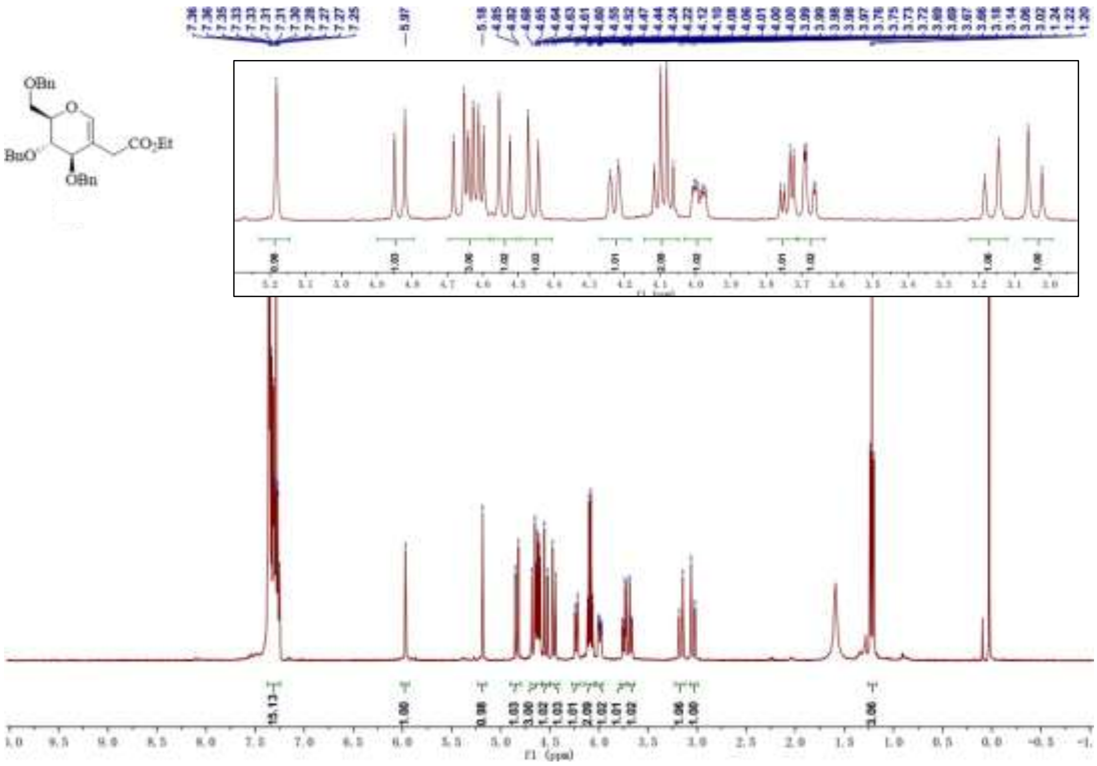

^{1}H NMR spectrum of Compound **1d**

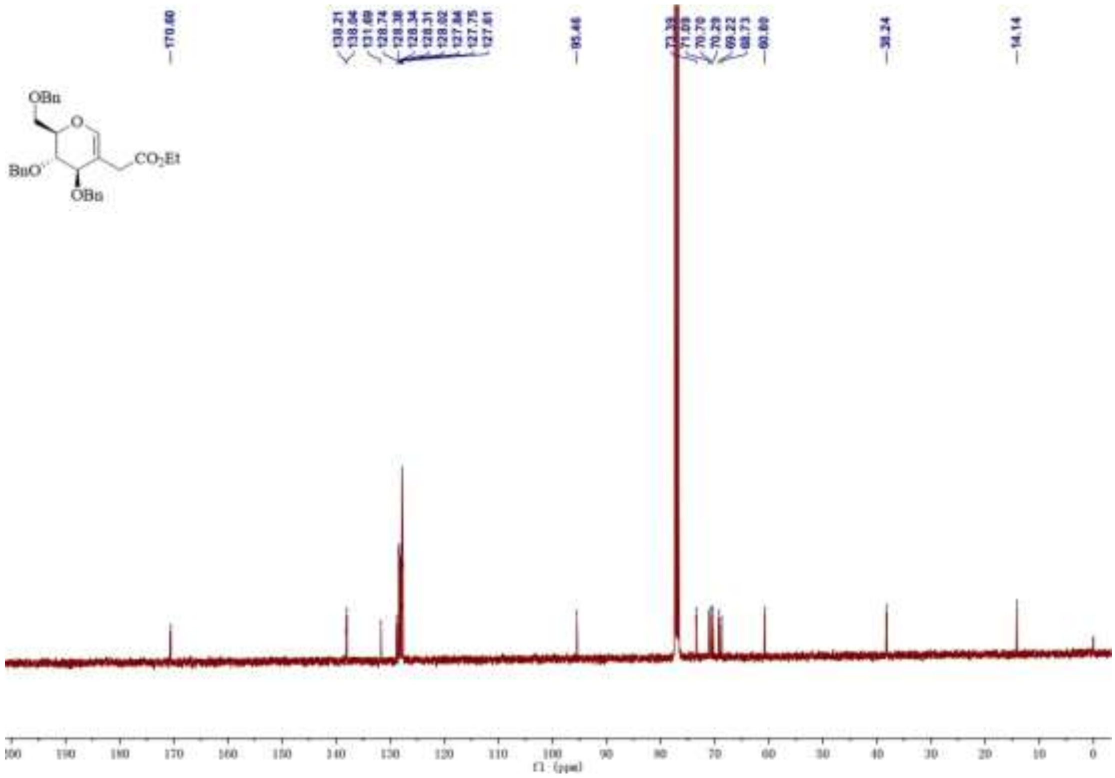

^{13}C NMR spectrum of Compound **1d**

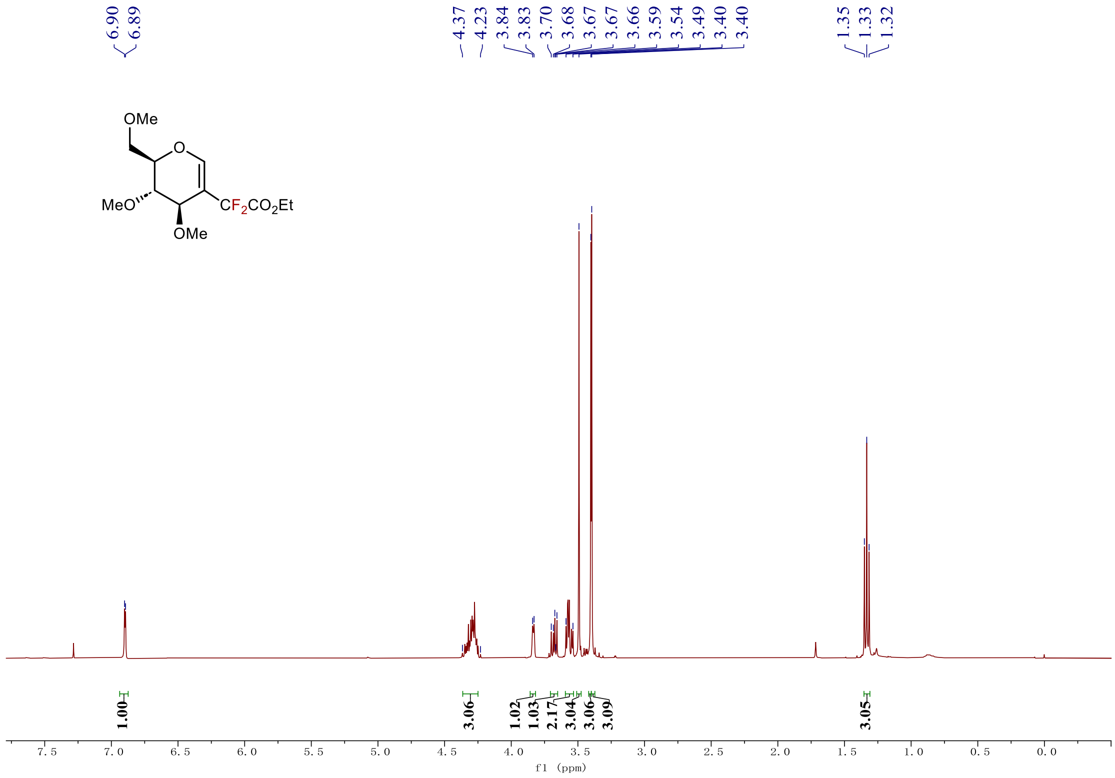

^{1}H NMR spectrum of Compound **1f**

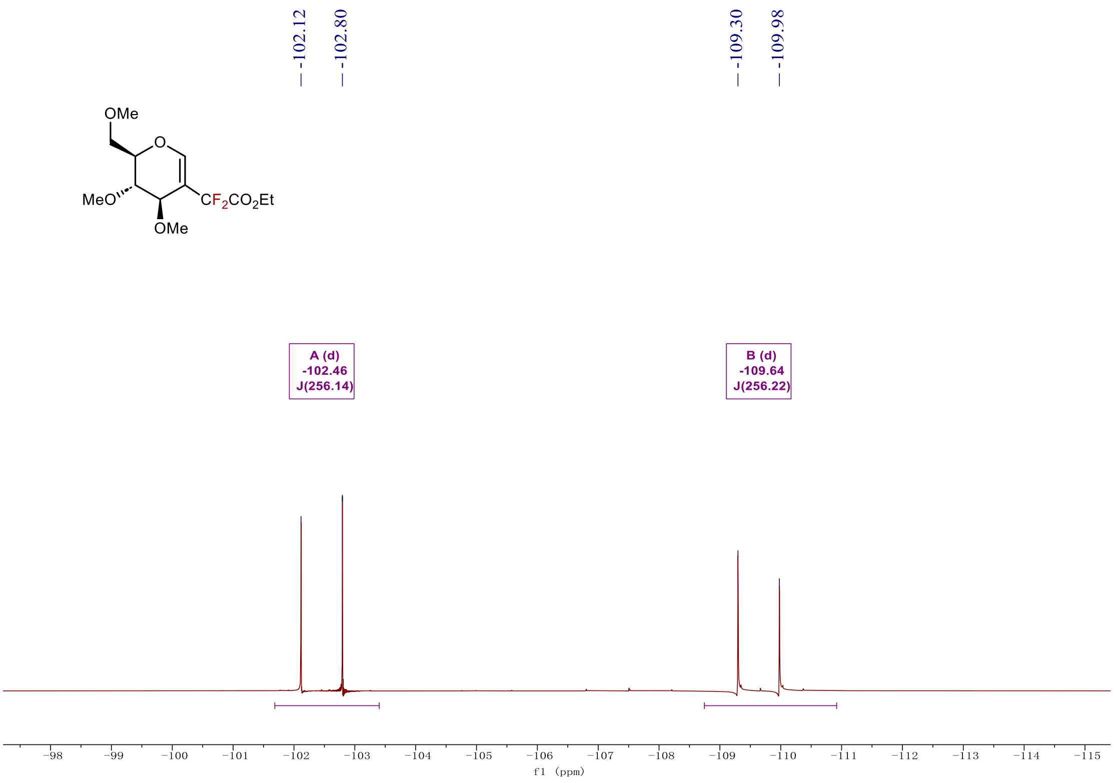

^{19}F NMR of Compound **1f**

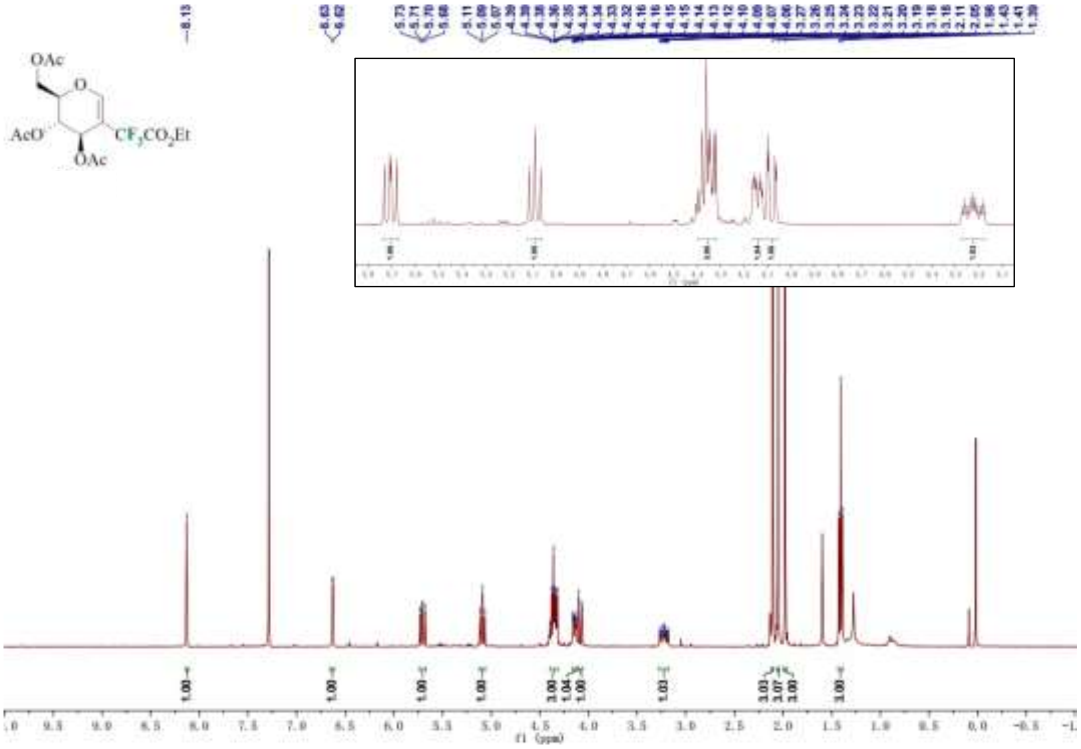

^{1}H NMR spectrum of Compound **1g**

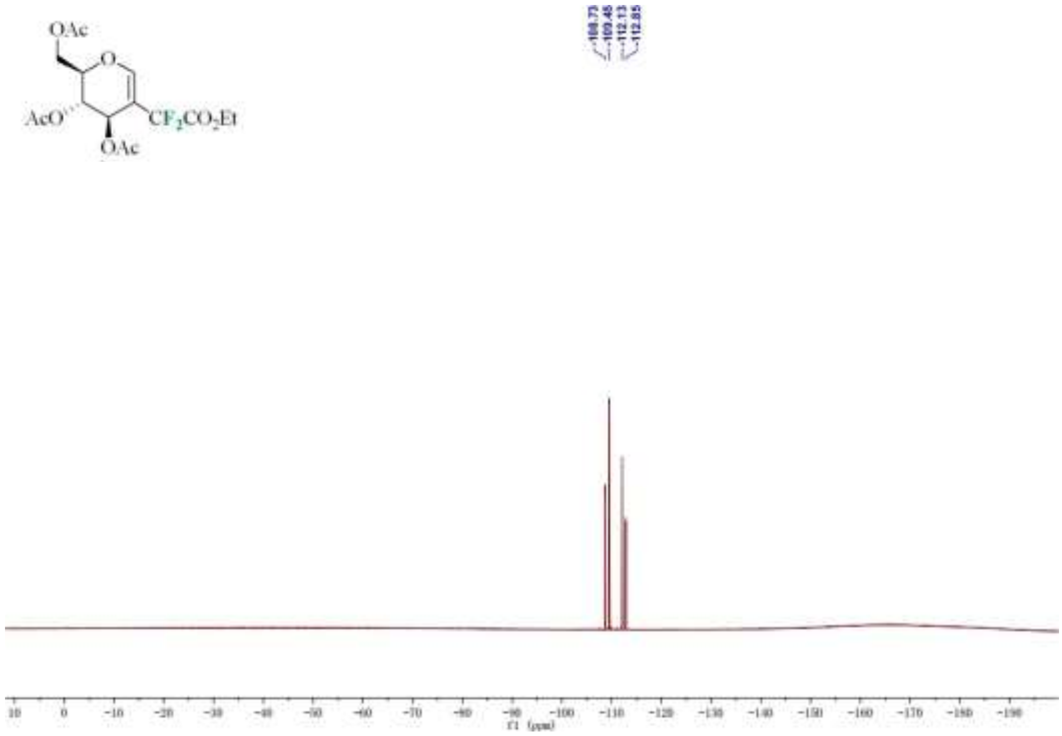

^{19}F NMR spectrum of Compound **1g**

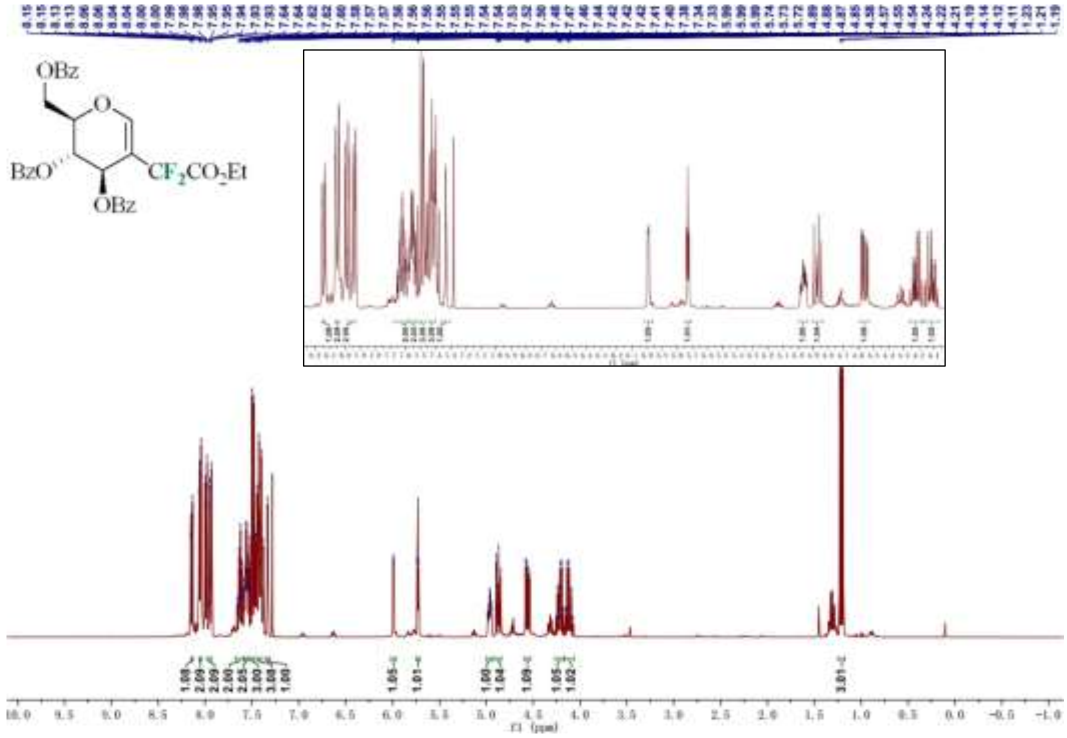

^{1}H NMR spectrum of Compound **1h**

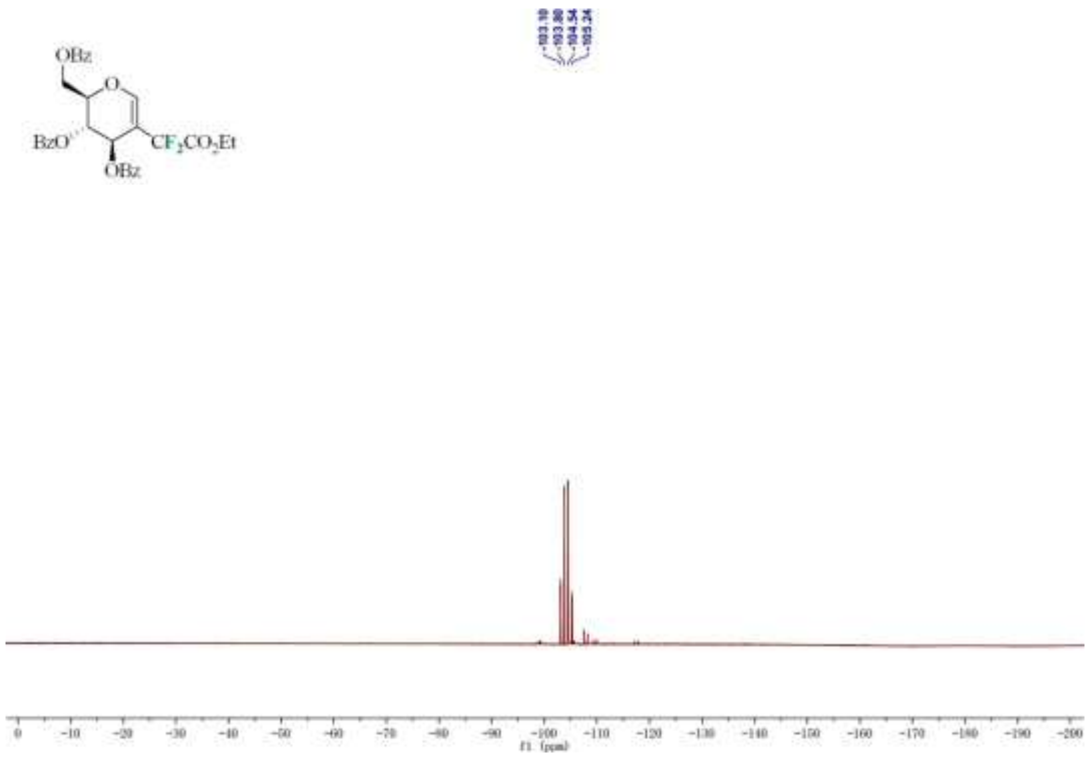

^{19}F NMR spectrum of Compound **1h**

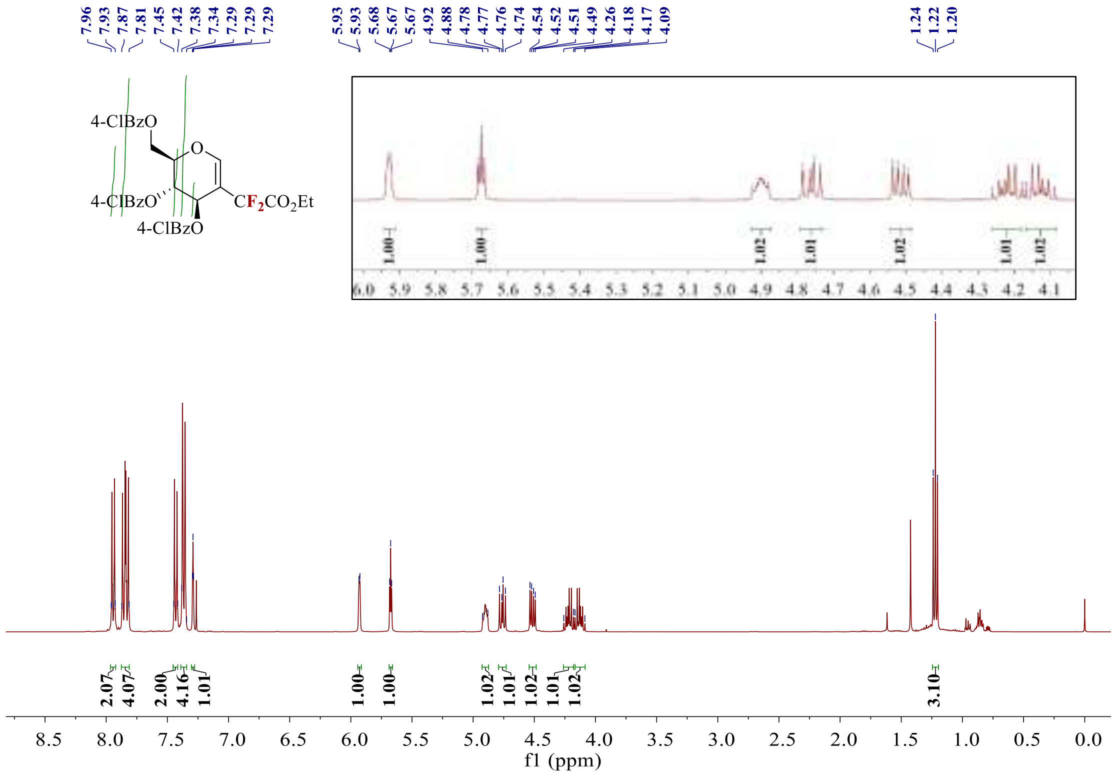

^{1}H NMR spectrum of Compound **1i**

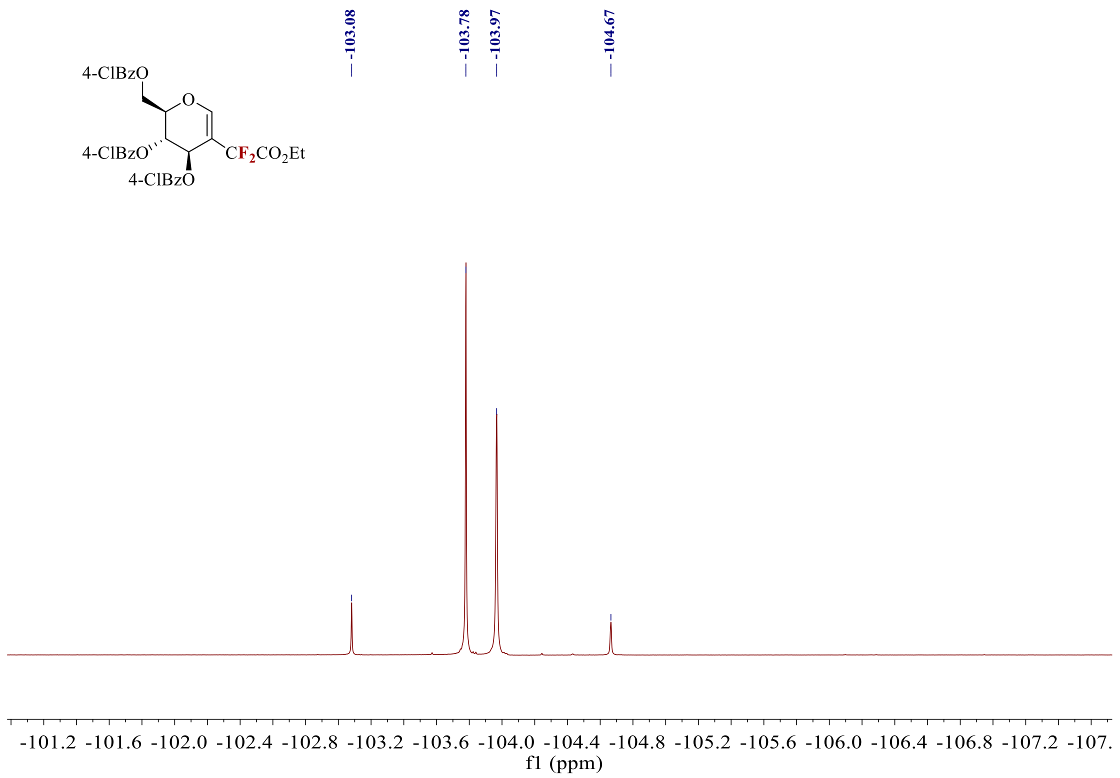

^{19}F NMR spectrum of Compound **1i**

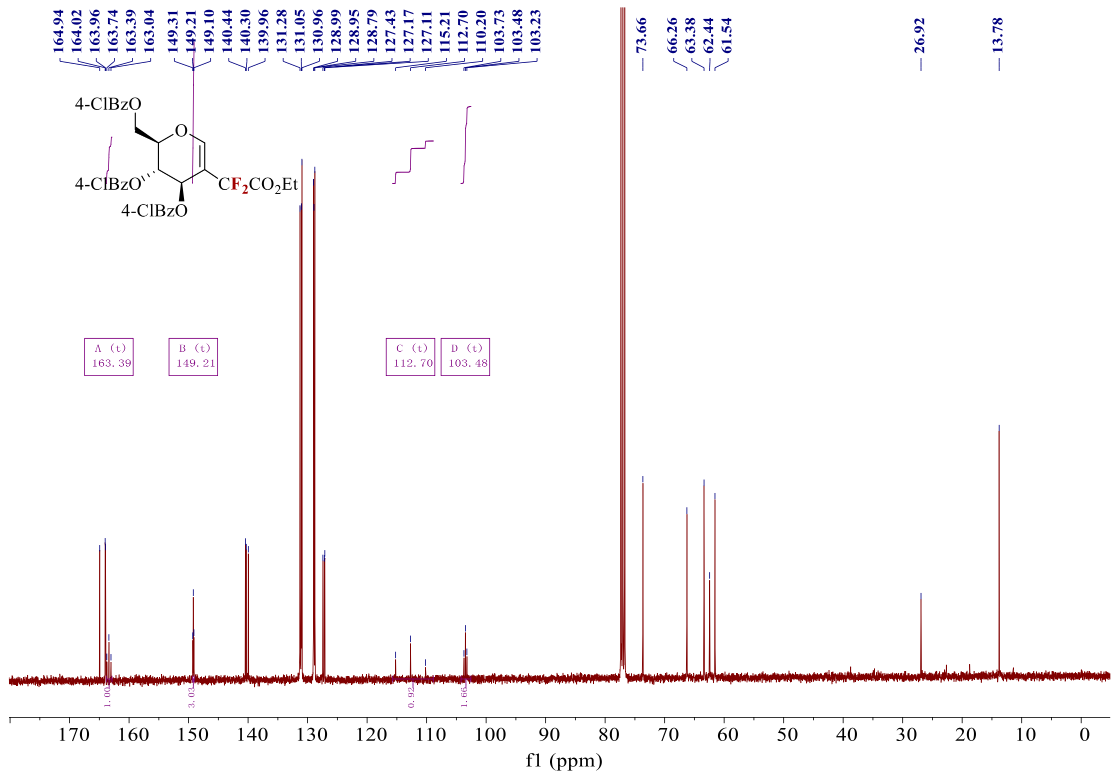

^{13}C NMR spectrum of Compound **1i**

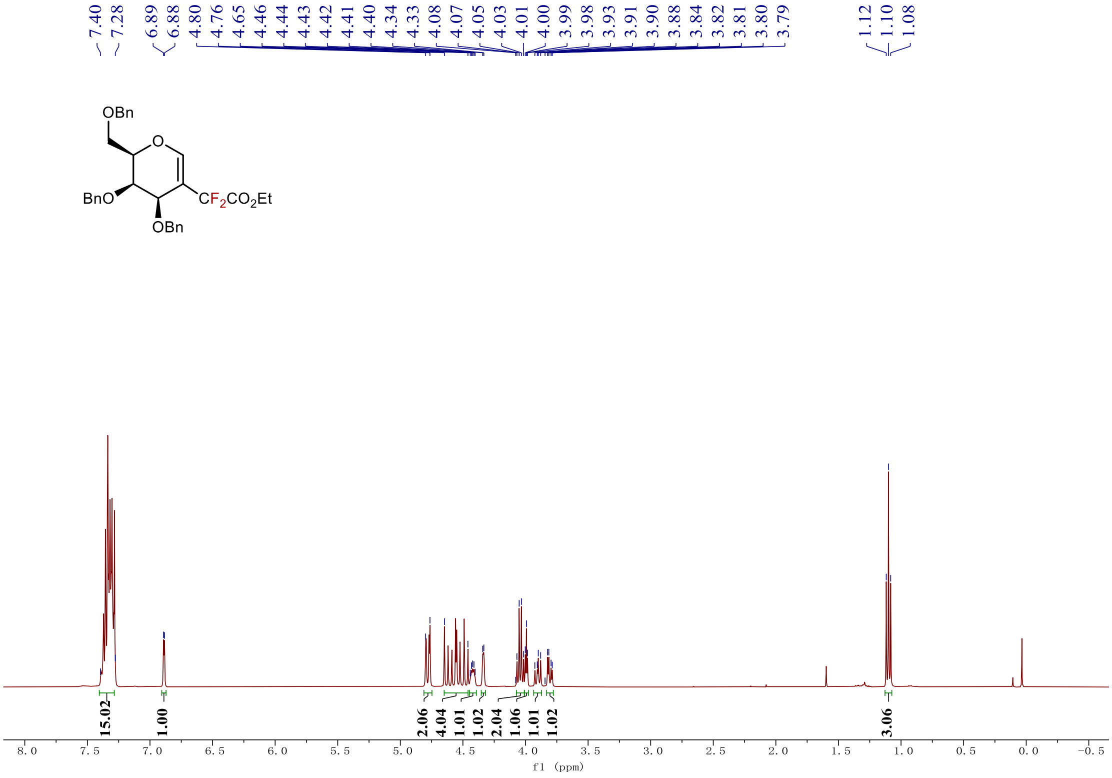

^{1}H NMR spectrum of Compound **1j**

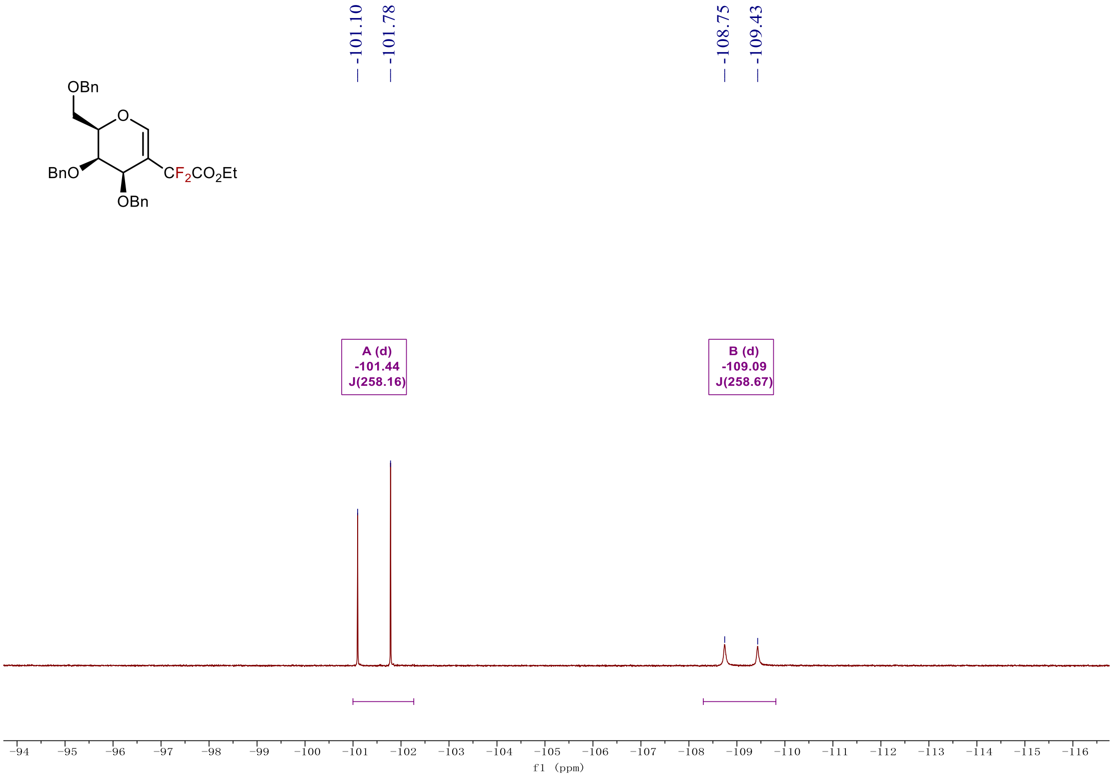

^{19}F NMR spectrum of Compound **1j**

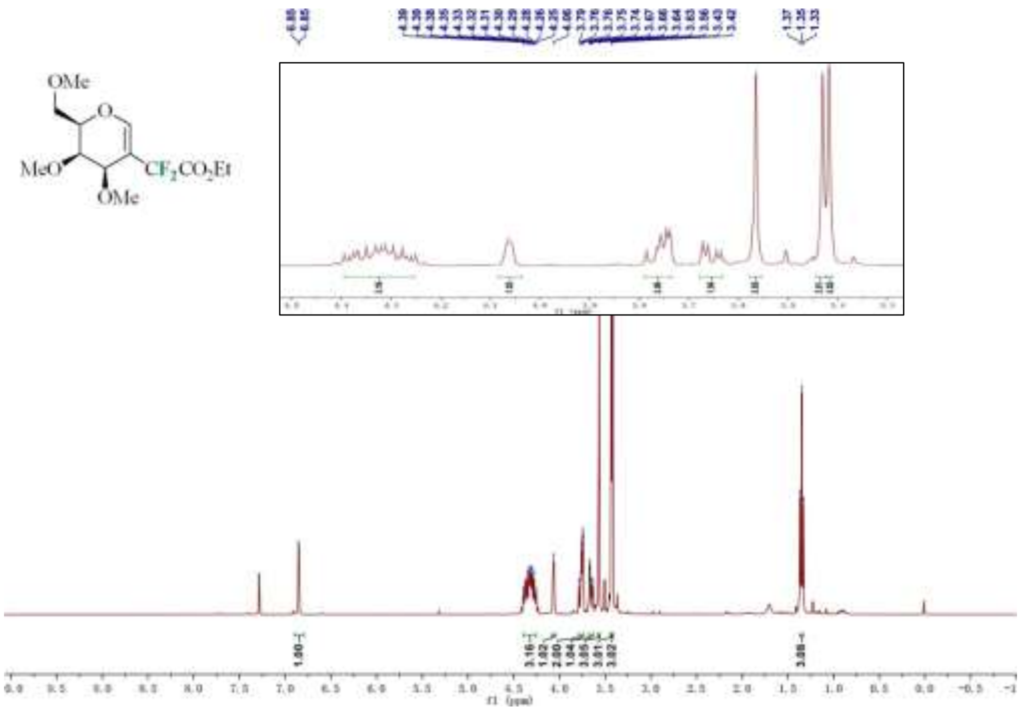

^{1}H NMR spectrum of Compound **1k**

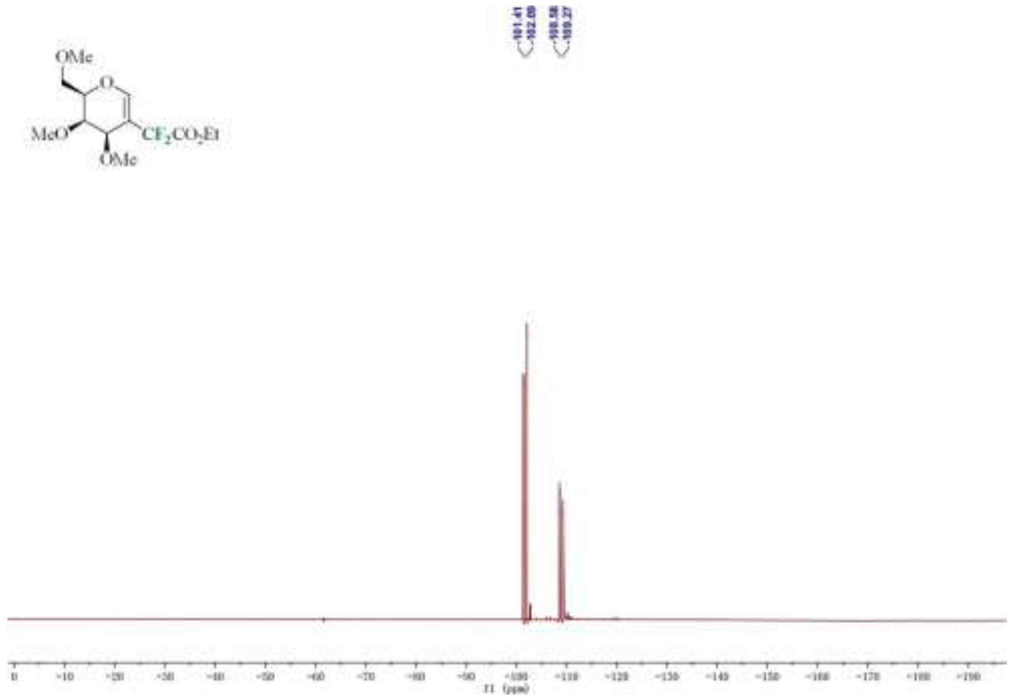

^{19}F NMR spectrum of Compound **1k**

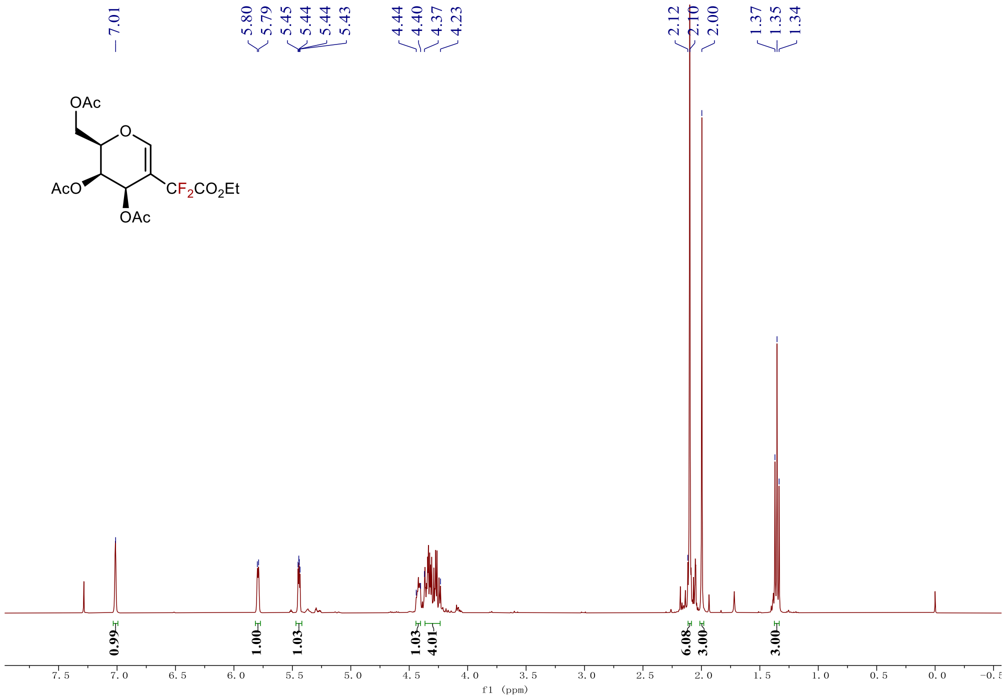

^{1}H NMR spectrum of Compound **1l**

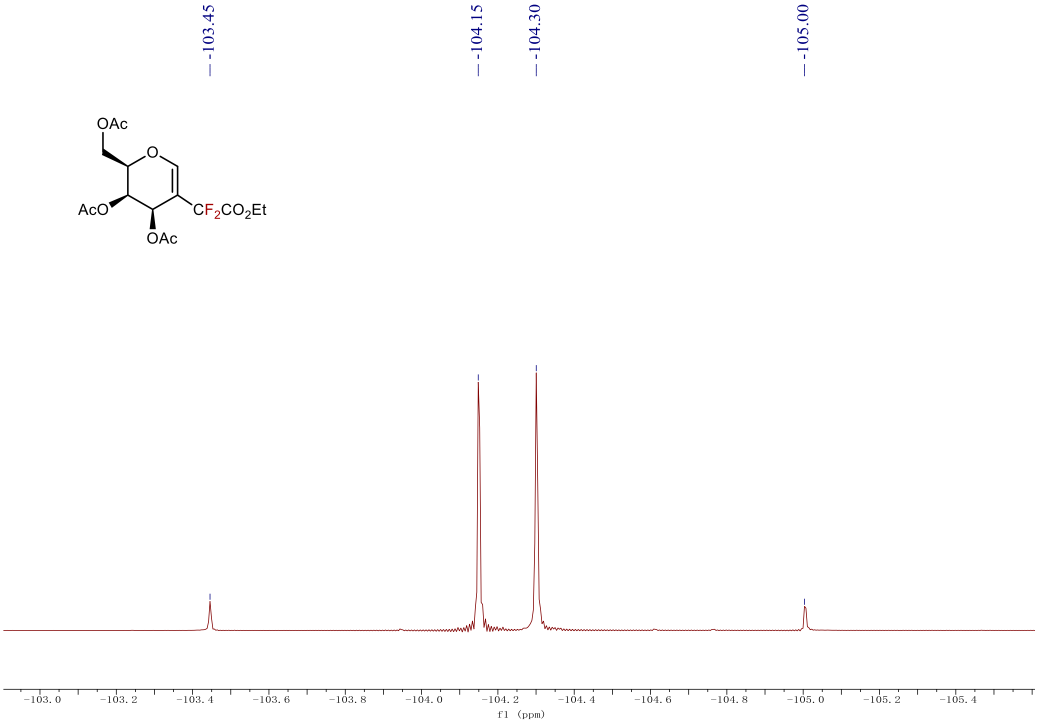

^{19}F NMR spectrum of Compound **1l**

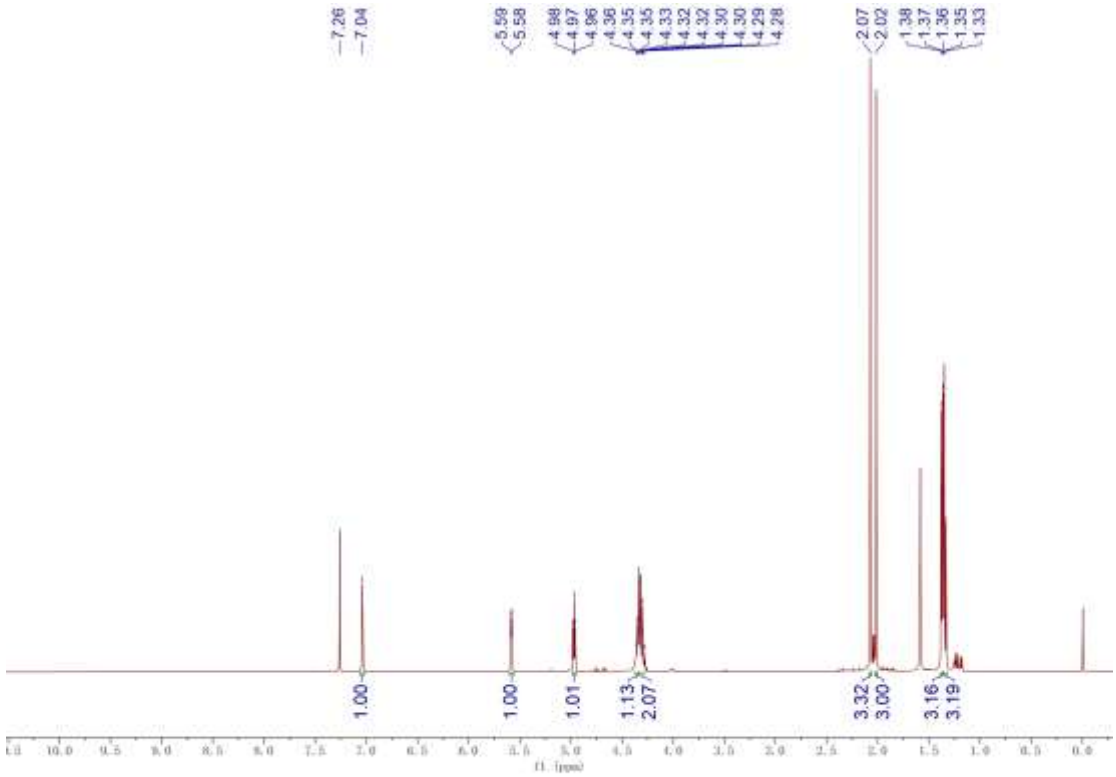

^{1}H NMR spectrum of Compound **1m**

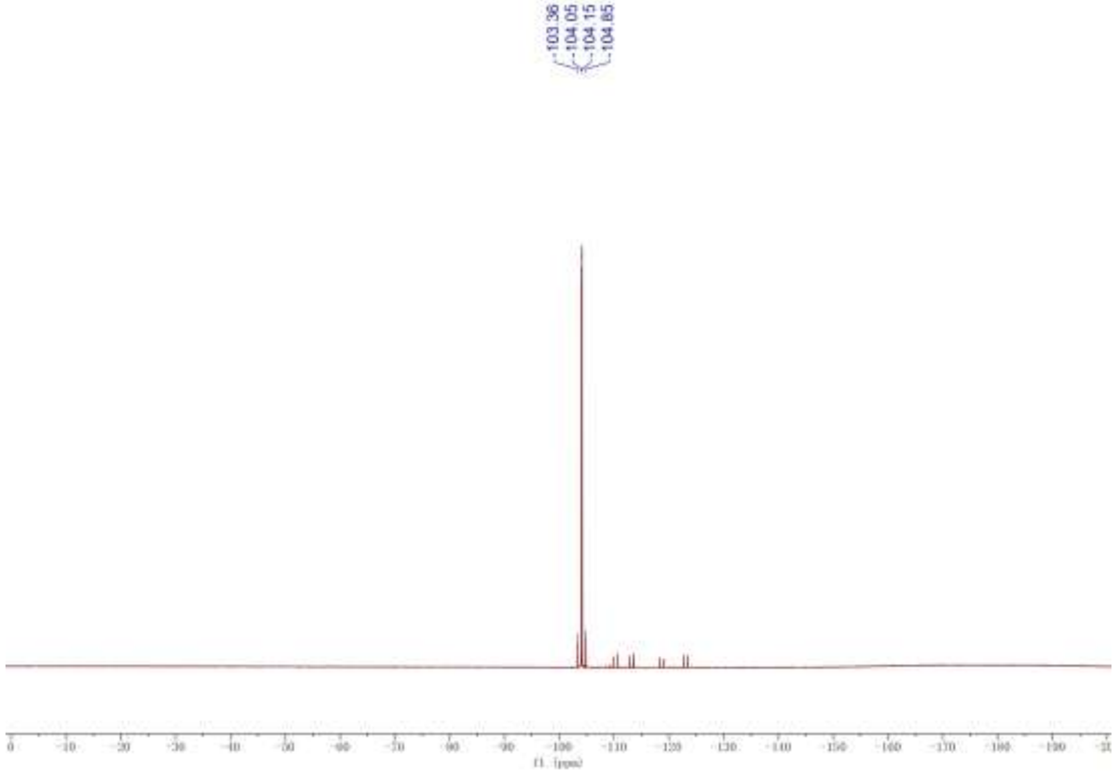

^{19}F NMR spectrum of Compound **1m**

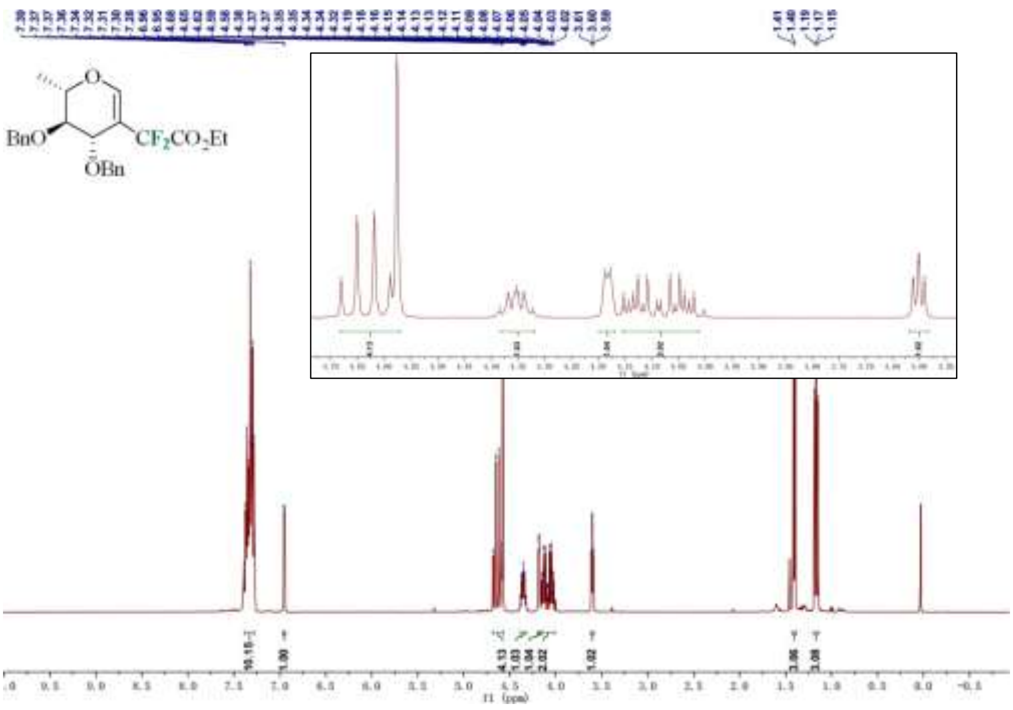

^{1}H NMR spectrum of Compound **1n**

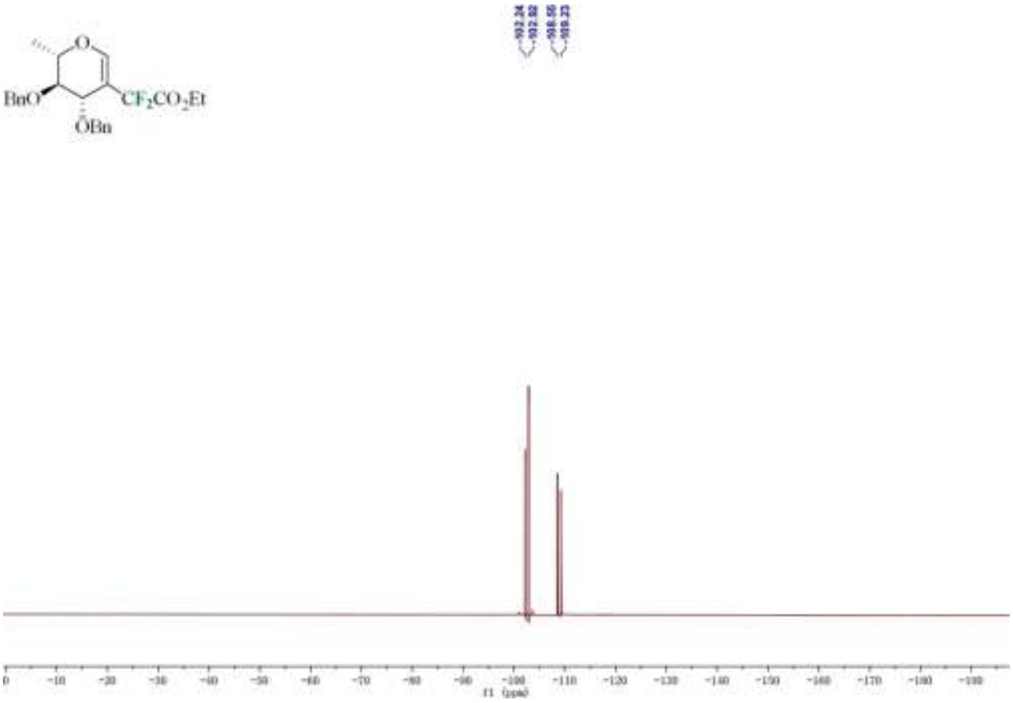

^{19}F NMR spectrum of Compound **1n**

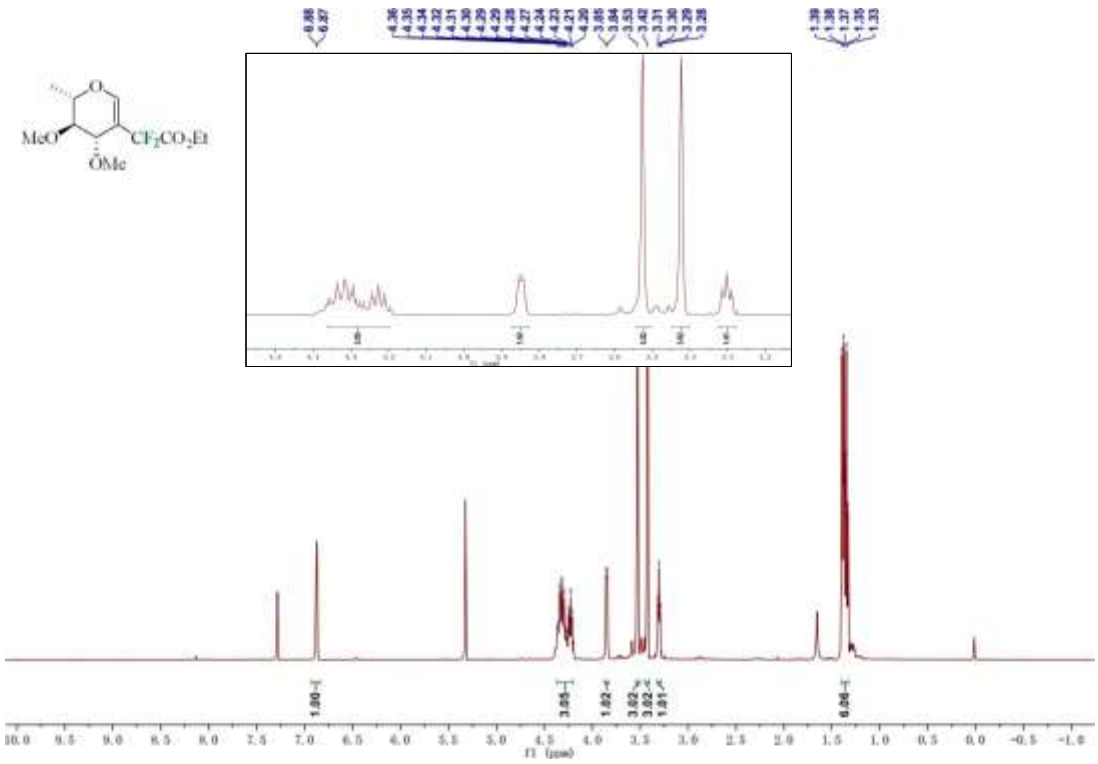

^{1}H NMR of spectrum Compound **1o**

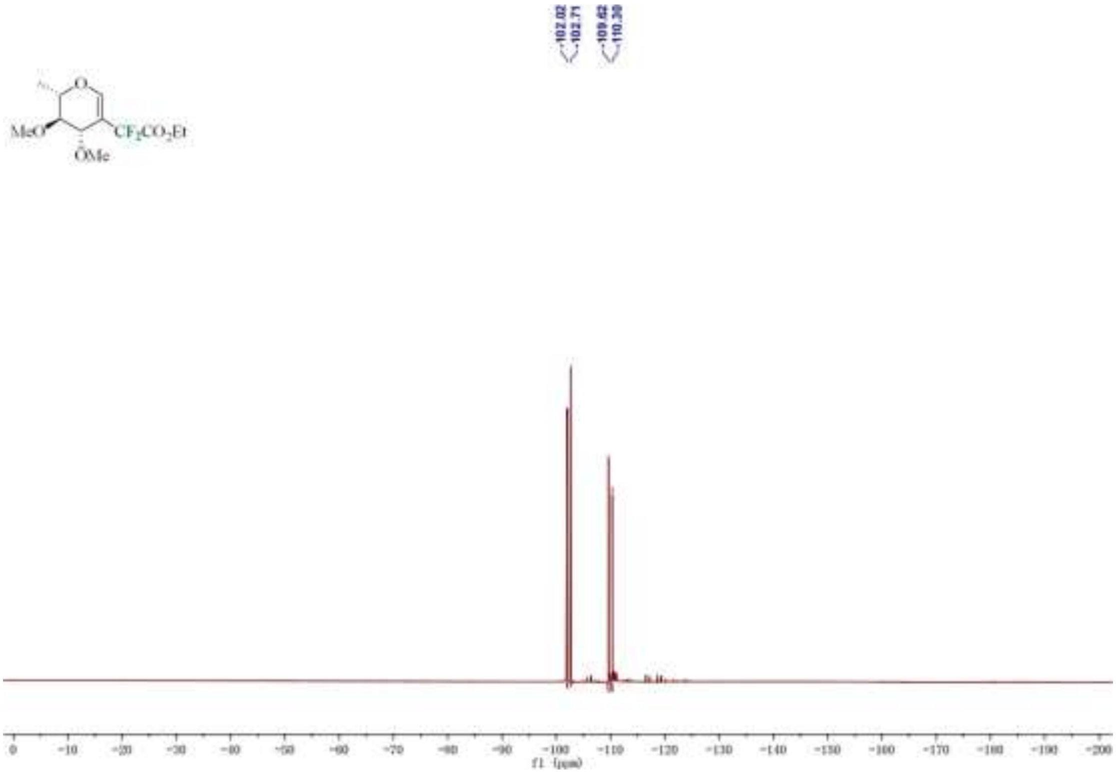

^{19}F NMR spectrum of Compound **1o**

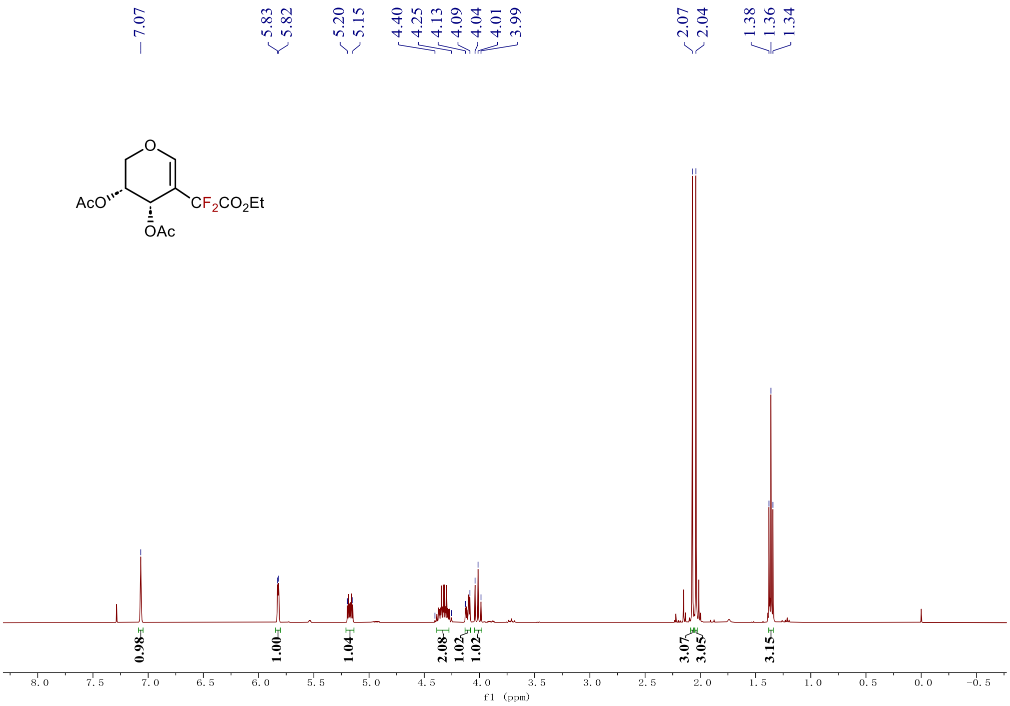

^{1}H NMR spectrum of Compound **1p**

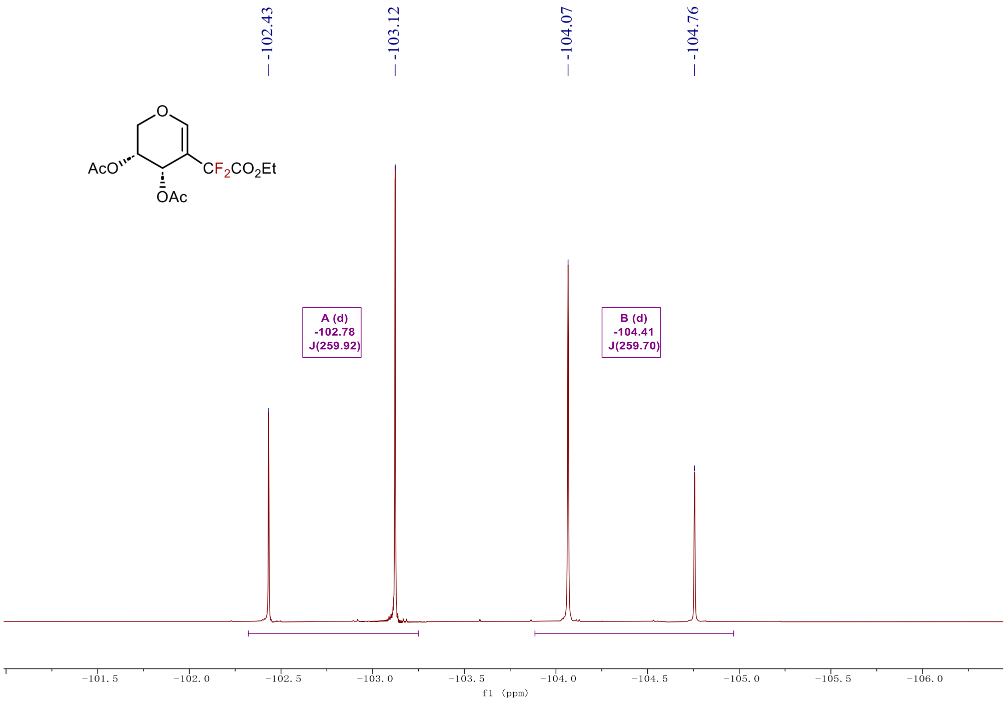

^{19}F NMR spectrum of Compound **1p**

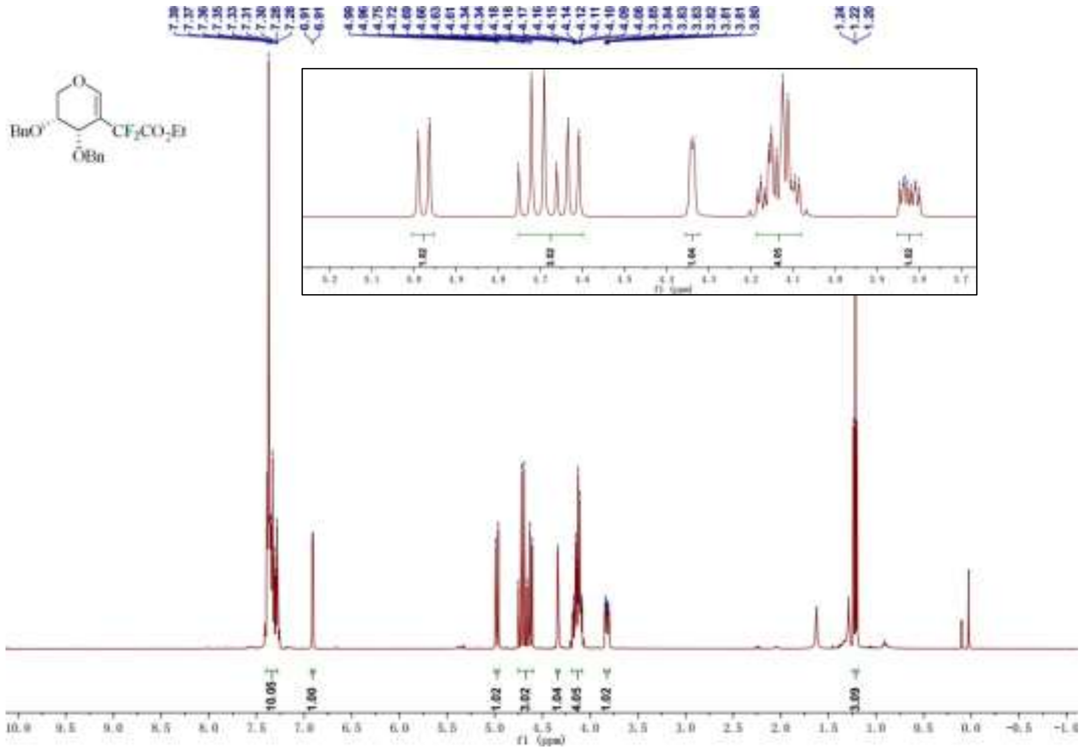

^{1}H NMR spectrum of Compound **1q**

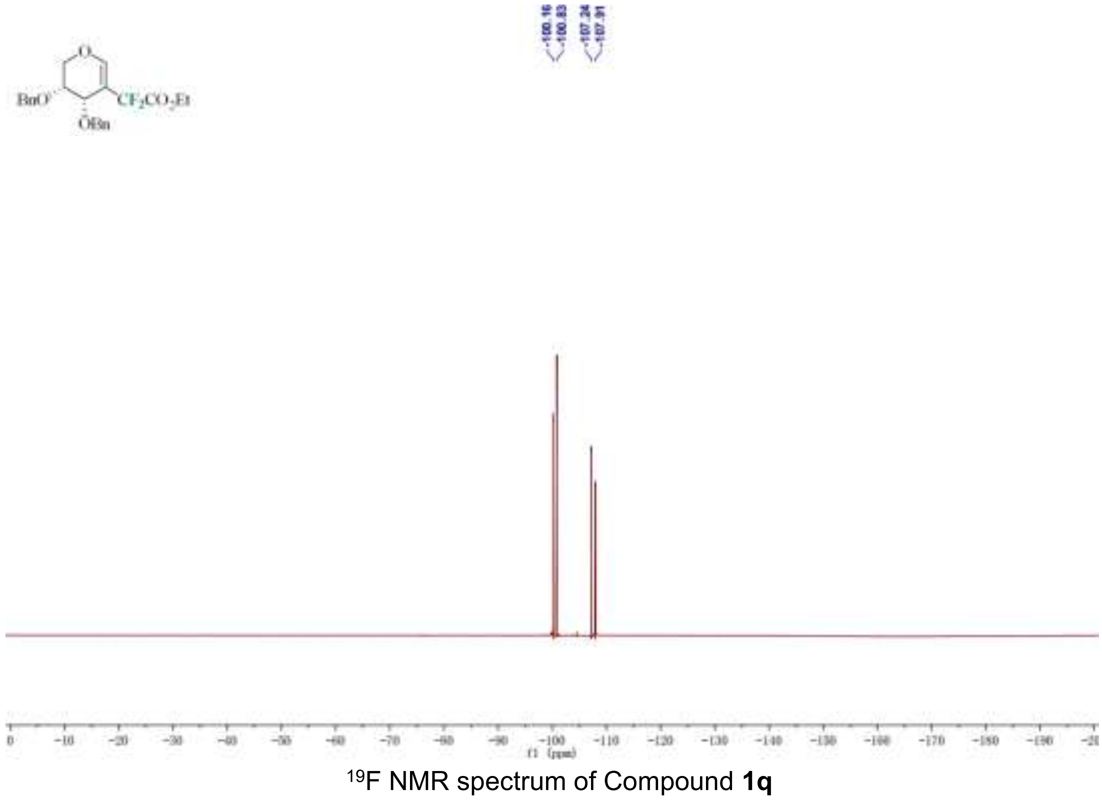

^{19}F NMR spectrum of Compound **1q**

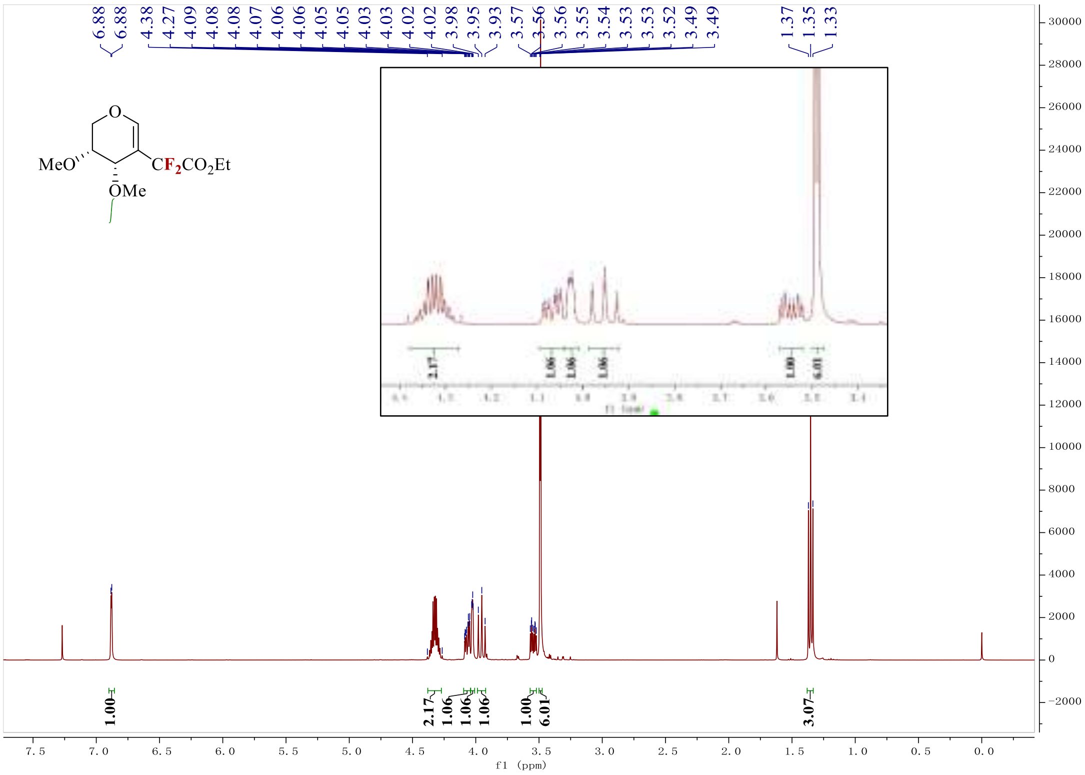

^{1}H NMR of spectrum Compound **1r**

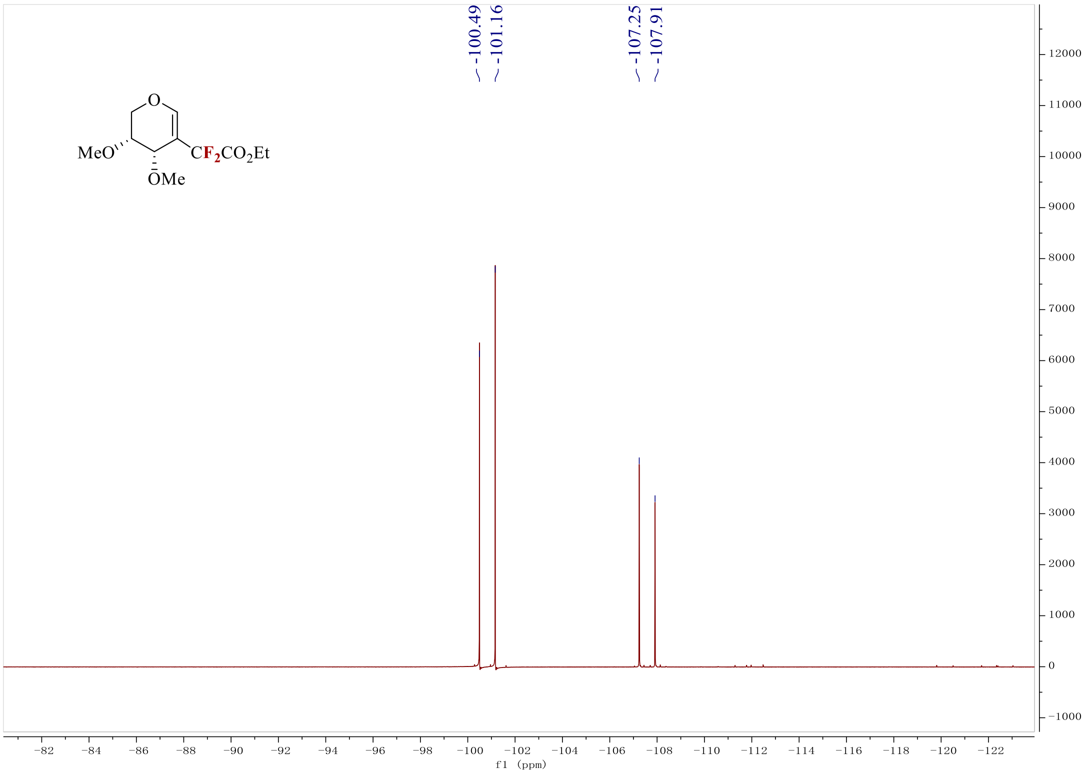

^{19}F NMR spectrum of Compound **1r**

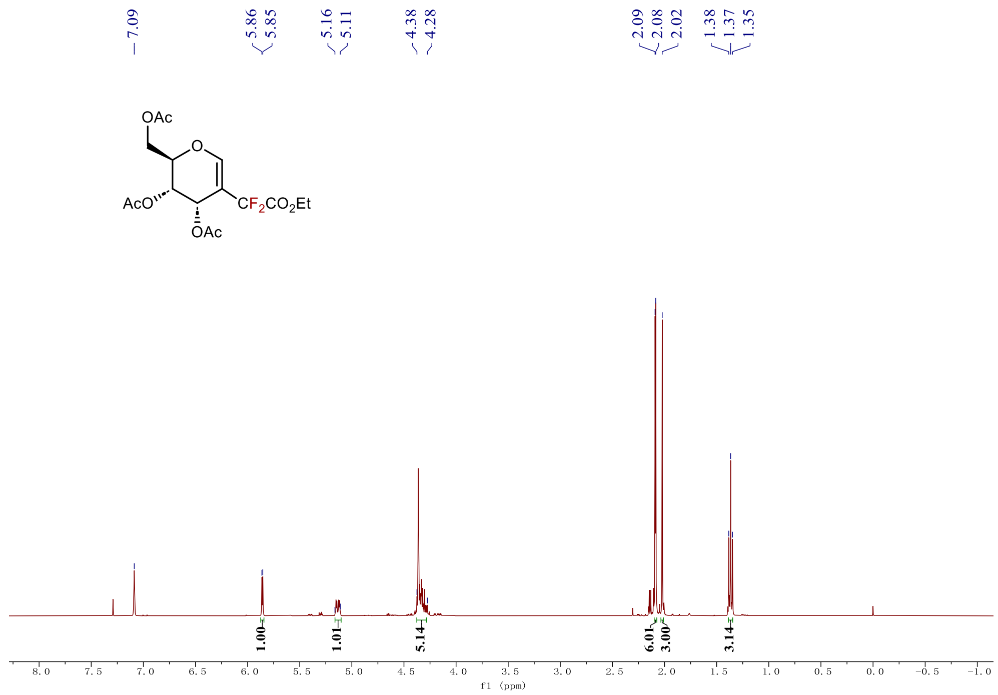

^{1}H NMR spectrum of Compound **1s**

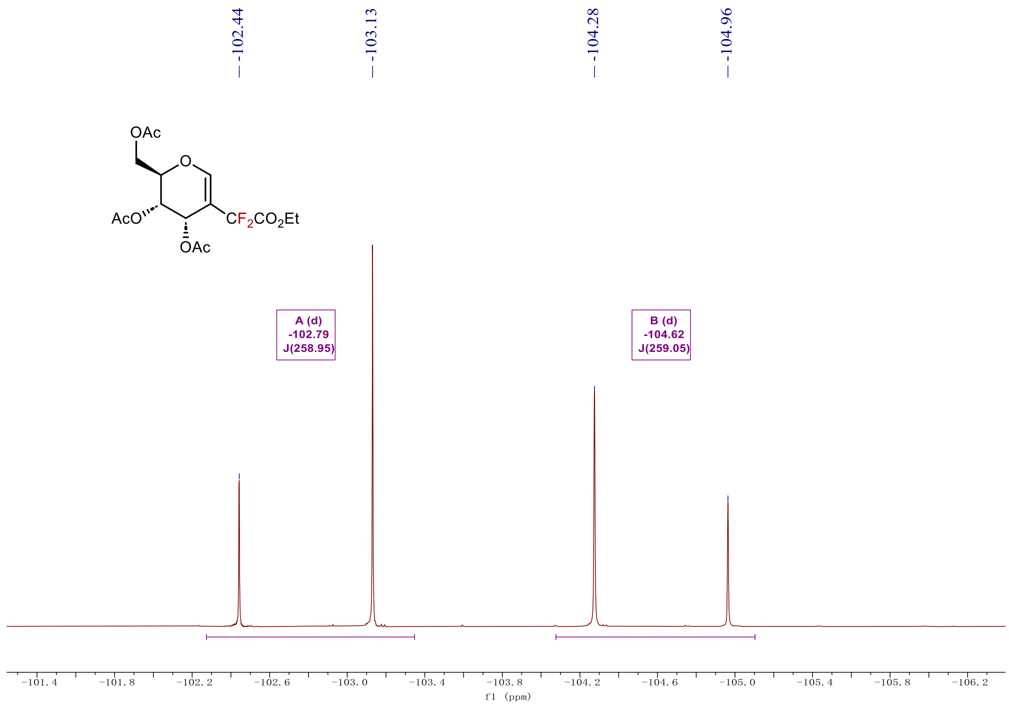

^{19}F NMR spectrum of Compound **1s**

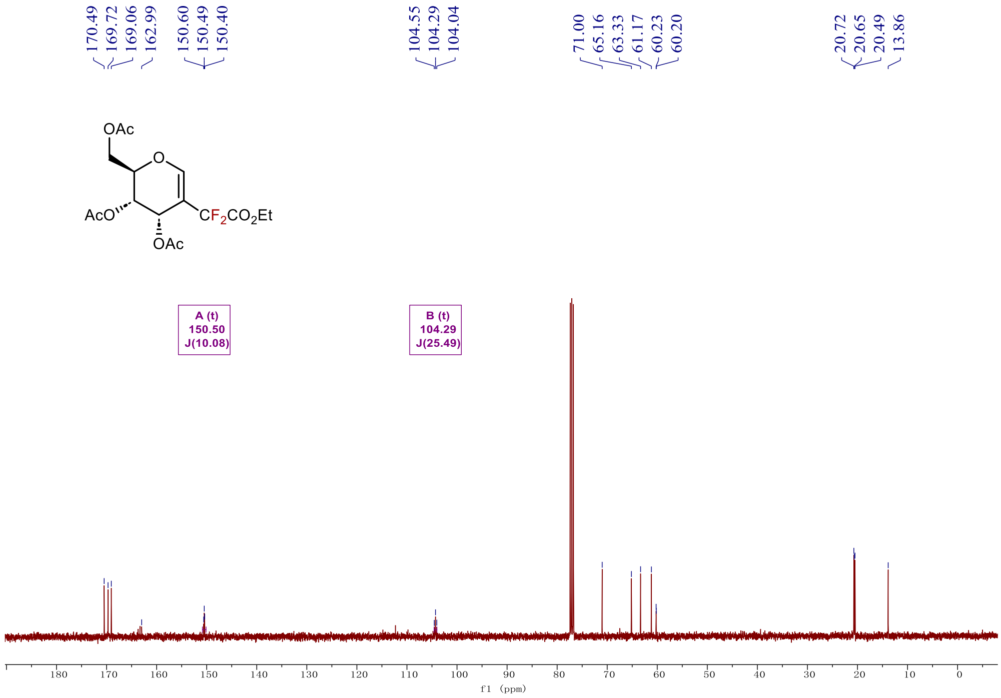

^{13}C NMR spectrum of Compound **1s**

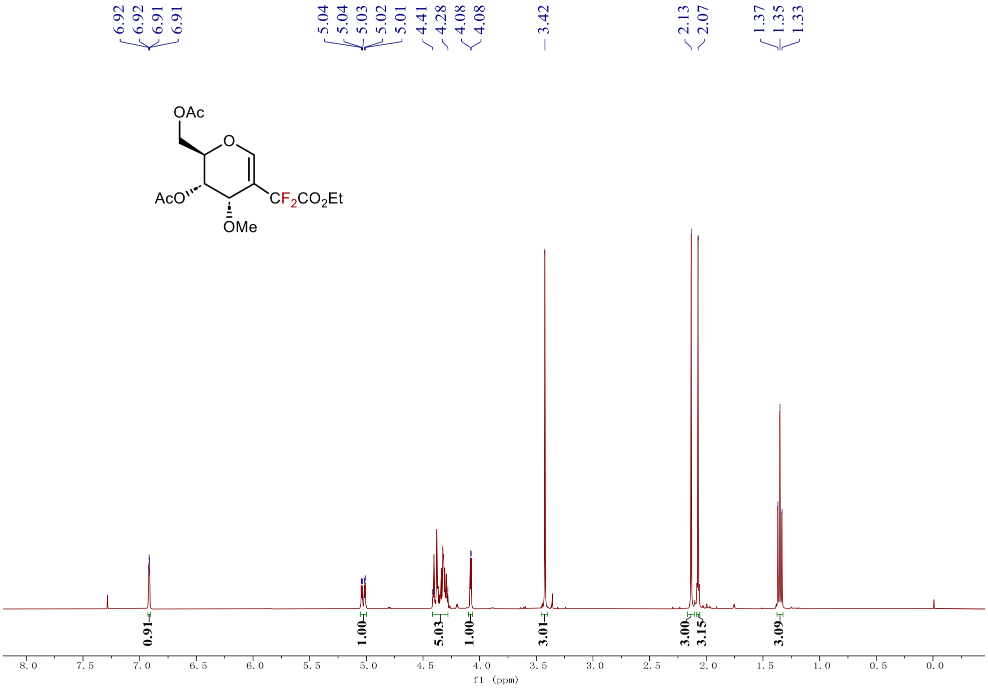

^{1}H NMR spectrum of Compound **1t**

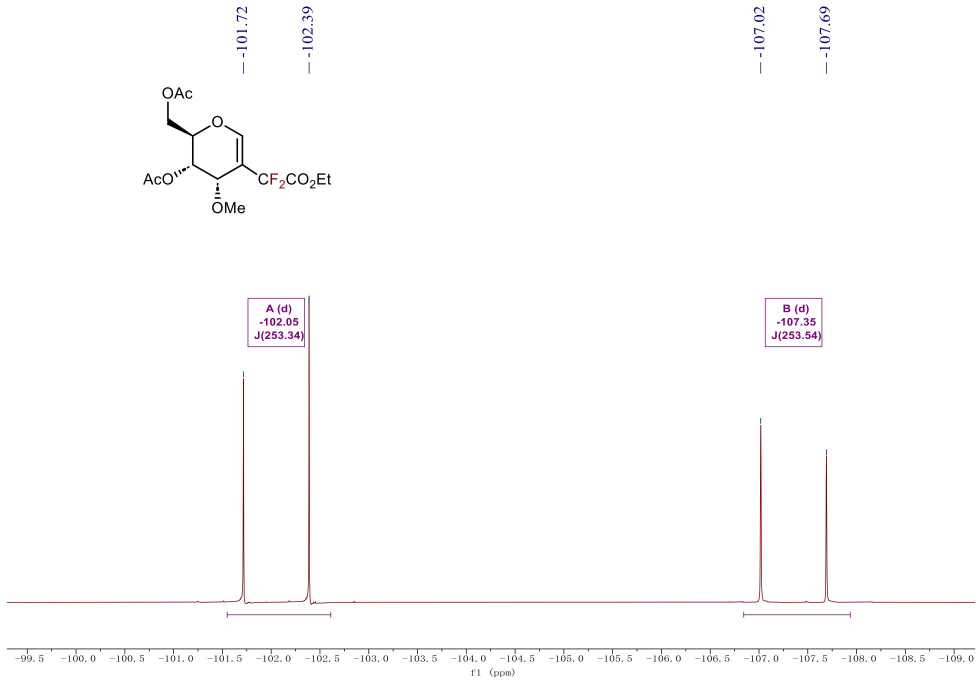

^{19}F NMR spectrum of Compound **1t**

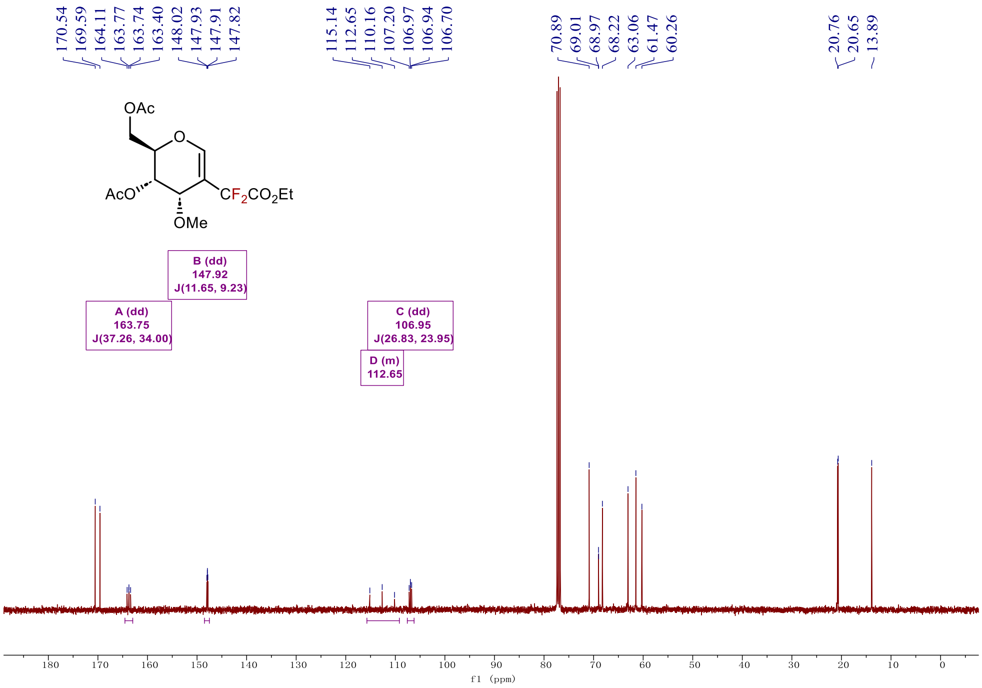

^{13}C NMR spectrum of Compound **1t**

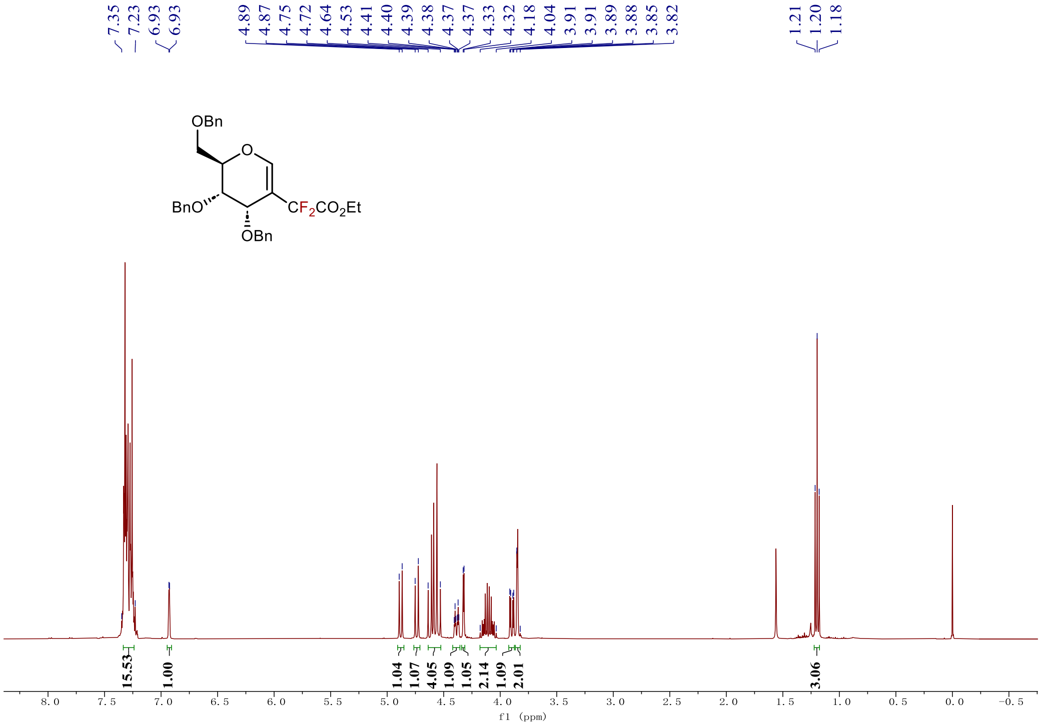

^1H NMR spectrum of Compound **1u**

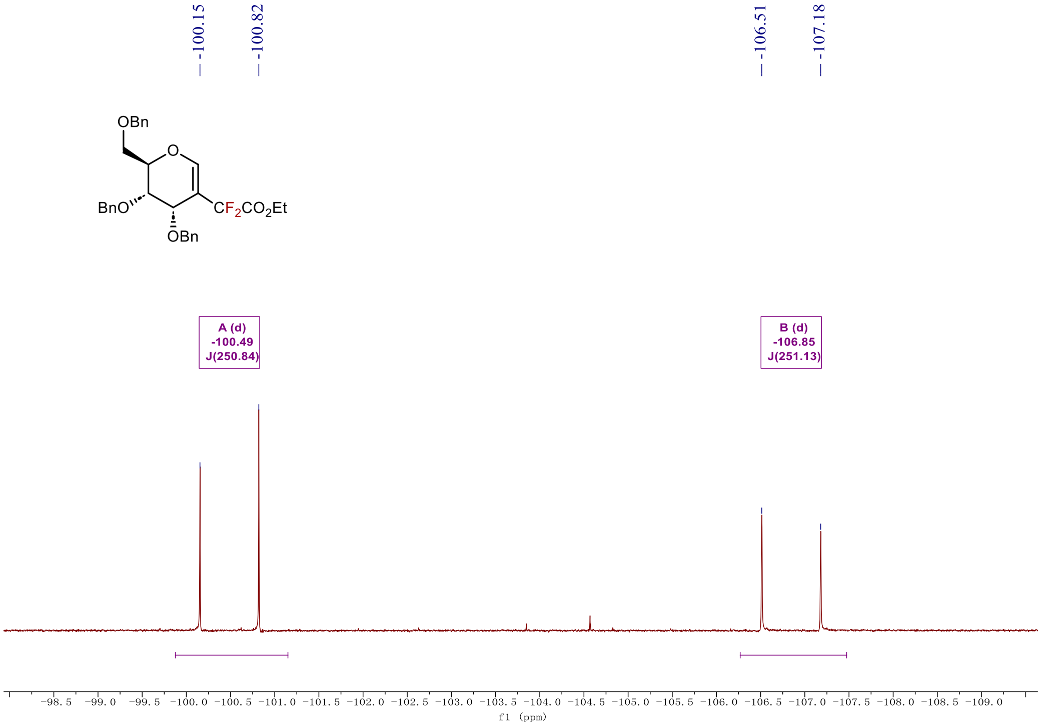

^{19}F NMR spectrum of Compound **1u**

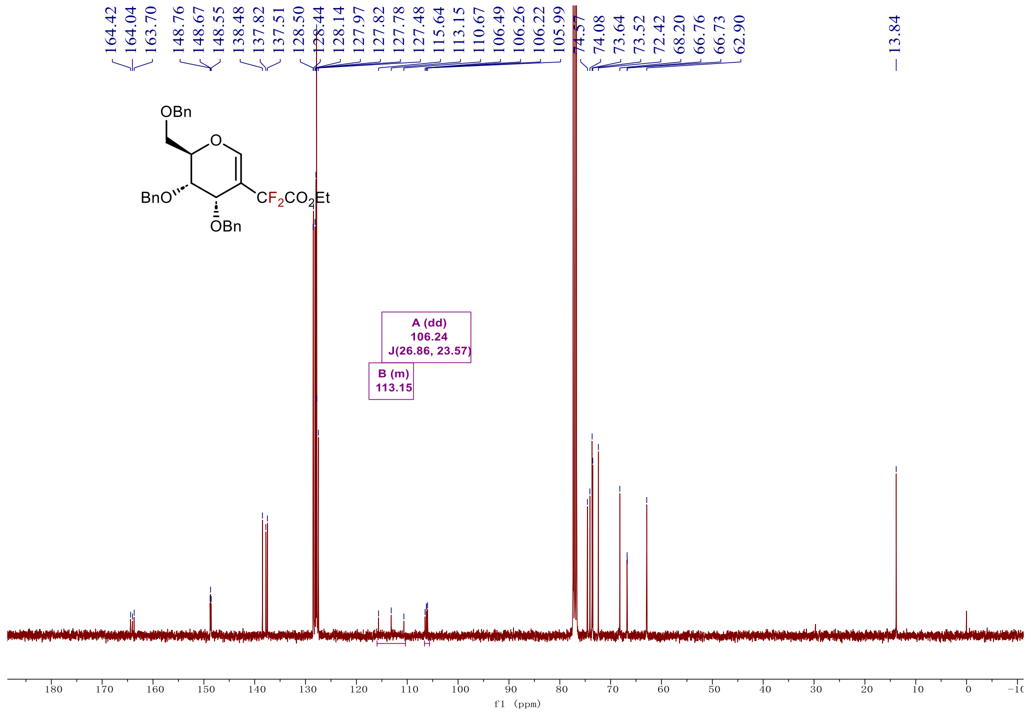

^{13}C NMR spectrum of Compound **1u**

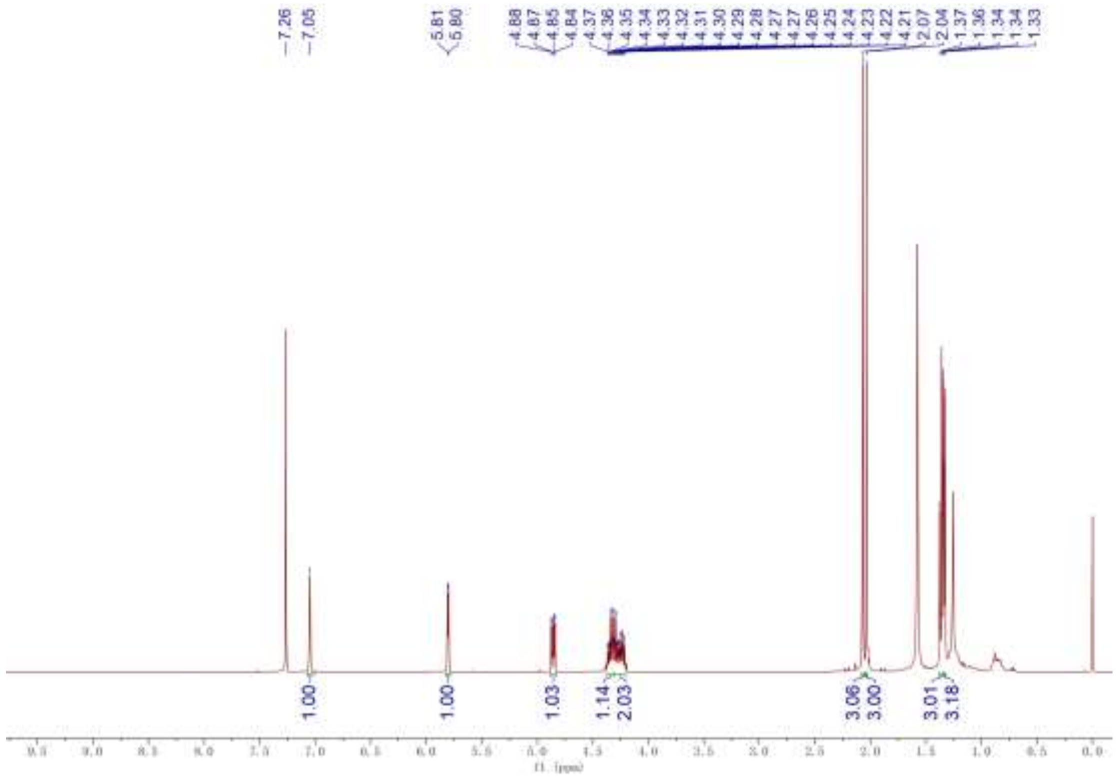

^{1}H NMR spectrum of Compound **1v**

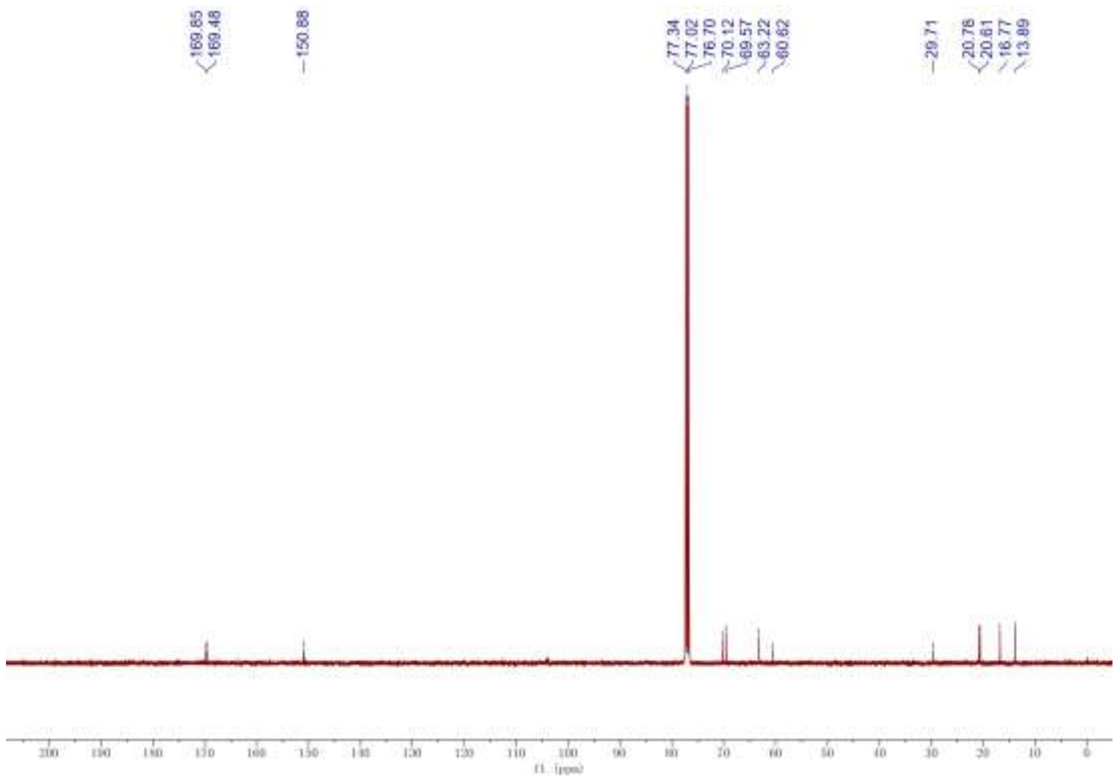

^{13}C NMR spectrum of Compound **1v**

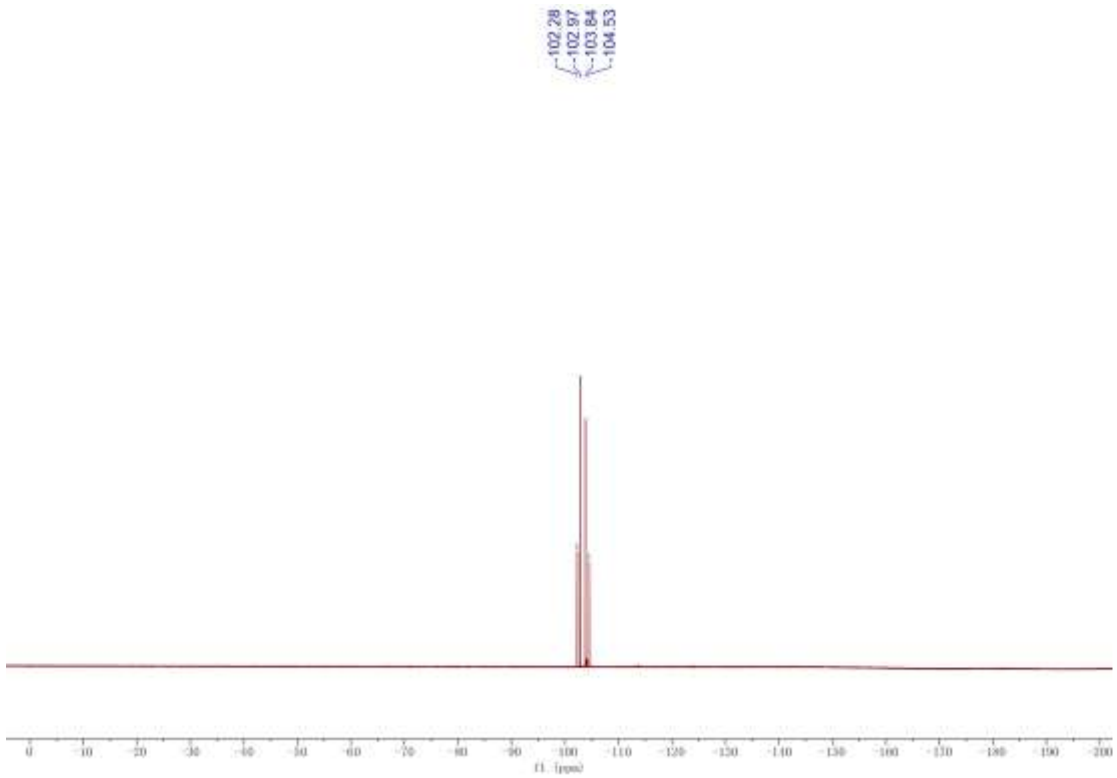

^{19}F NMR spectrum of Compound **1v**

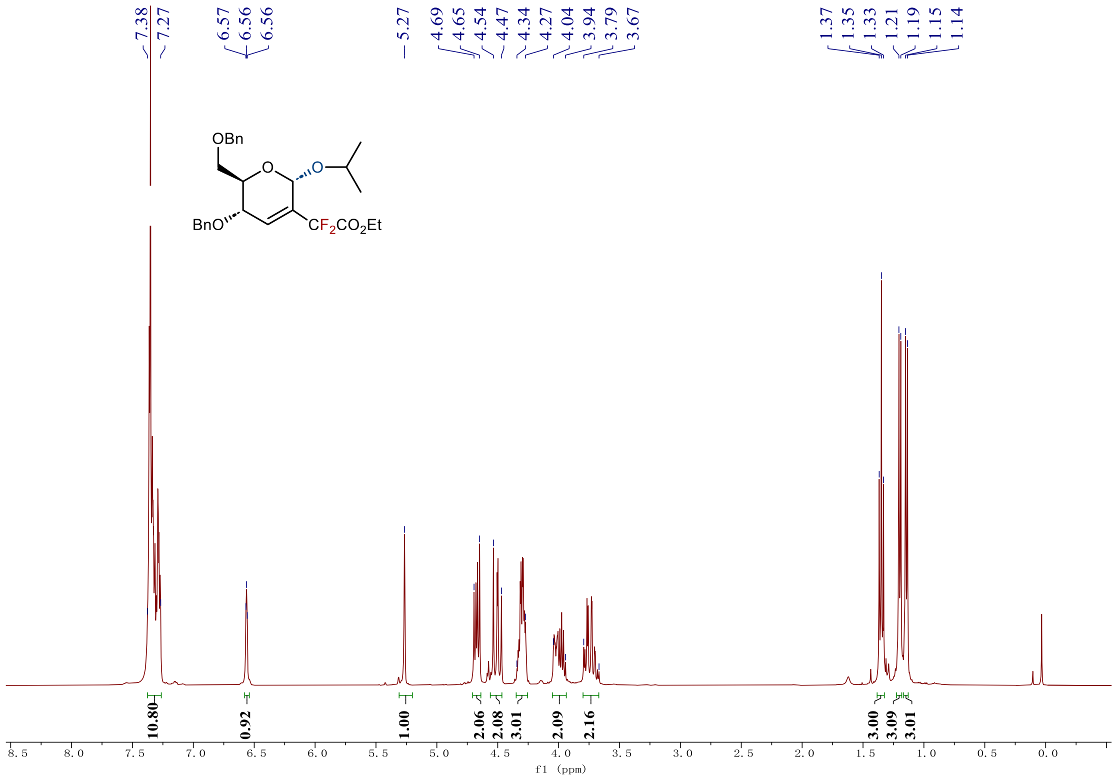

^{1}H NMR spectrum of Compound **2a**

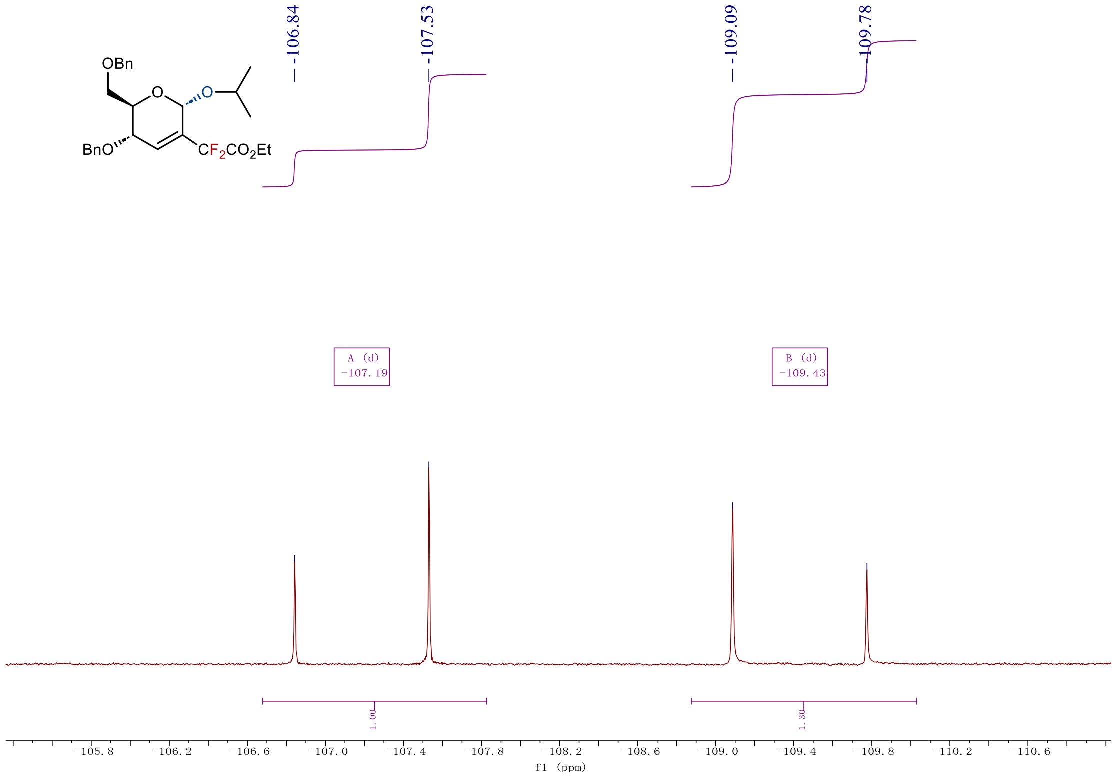

^{19}F NMR spectrum of Compound **2a**

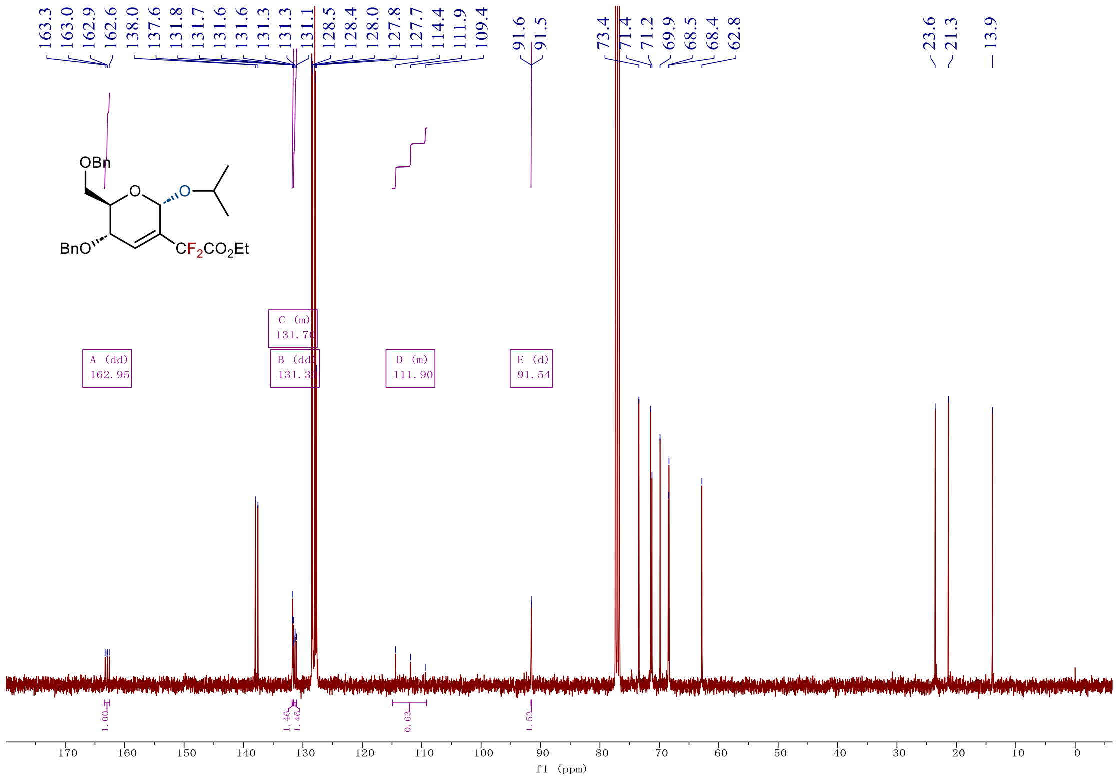

^{13}C NMR spectrum of Compound **2a**

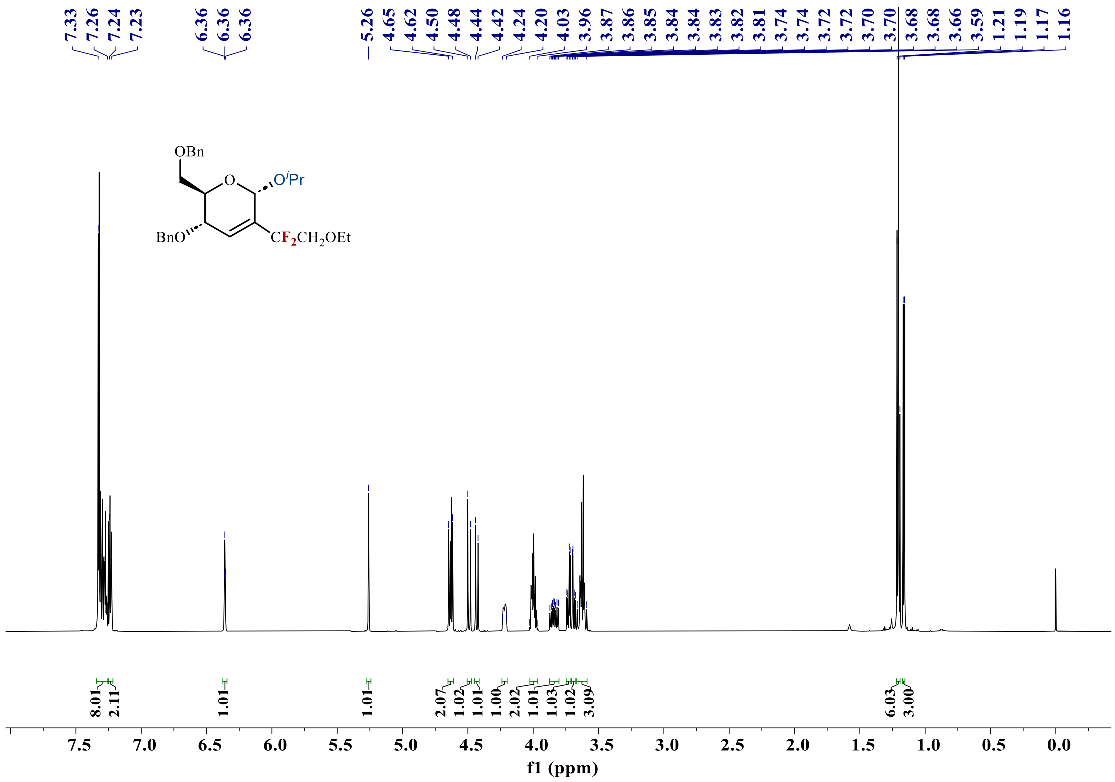

^{1}H NMR spectrum of Compound **2b**

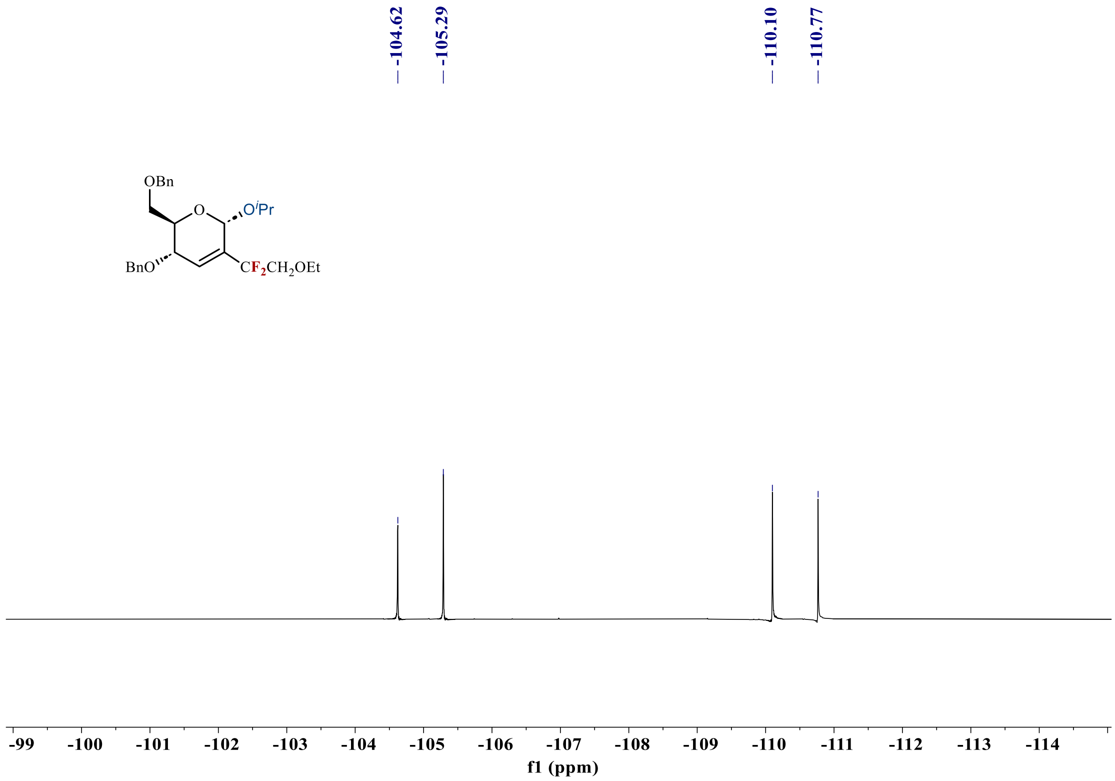

^{19}F NMR spectrum of Compound **2b**

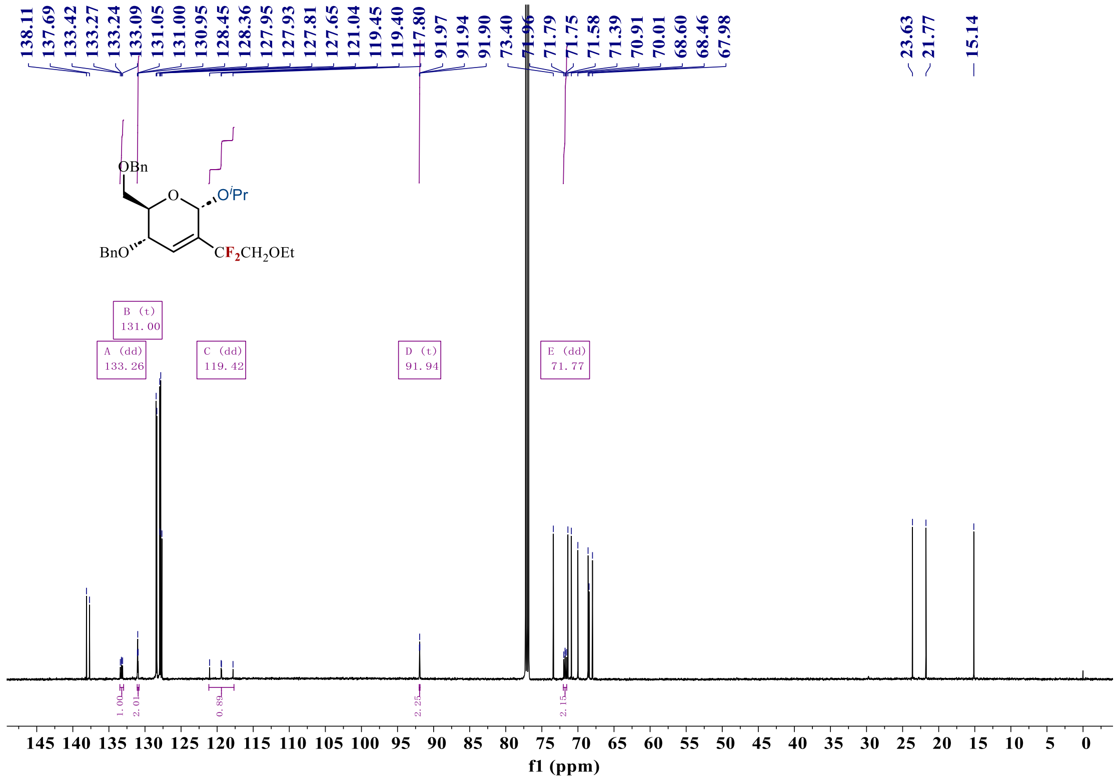

^{13}C NMR spectrum of Compound **2b**

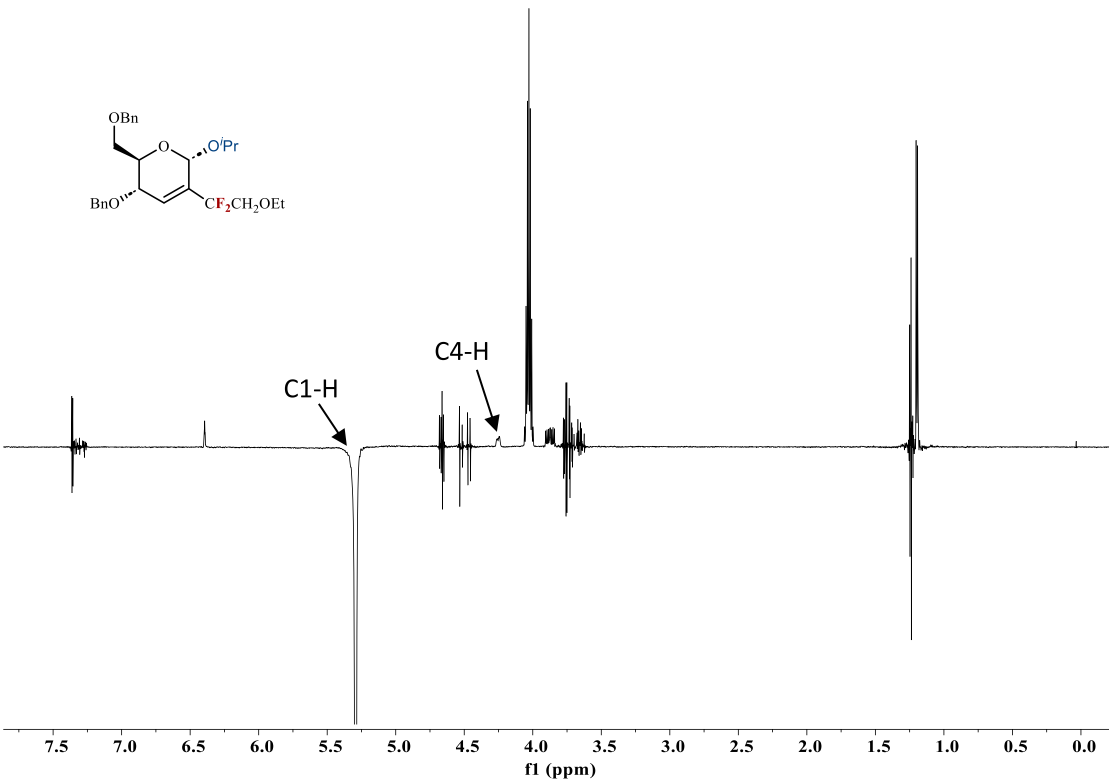

1D NOE spectrum of Compound **2b**

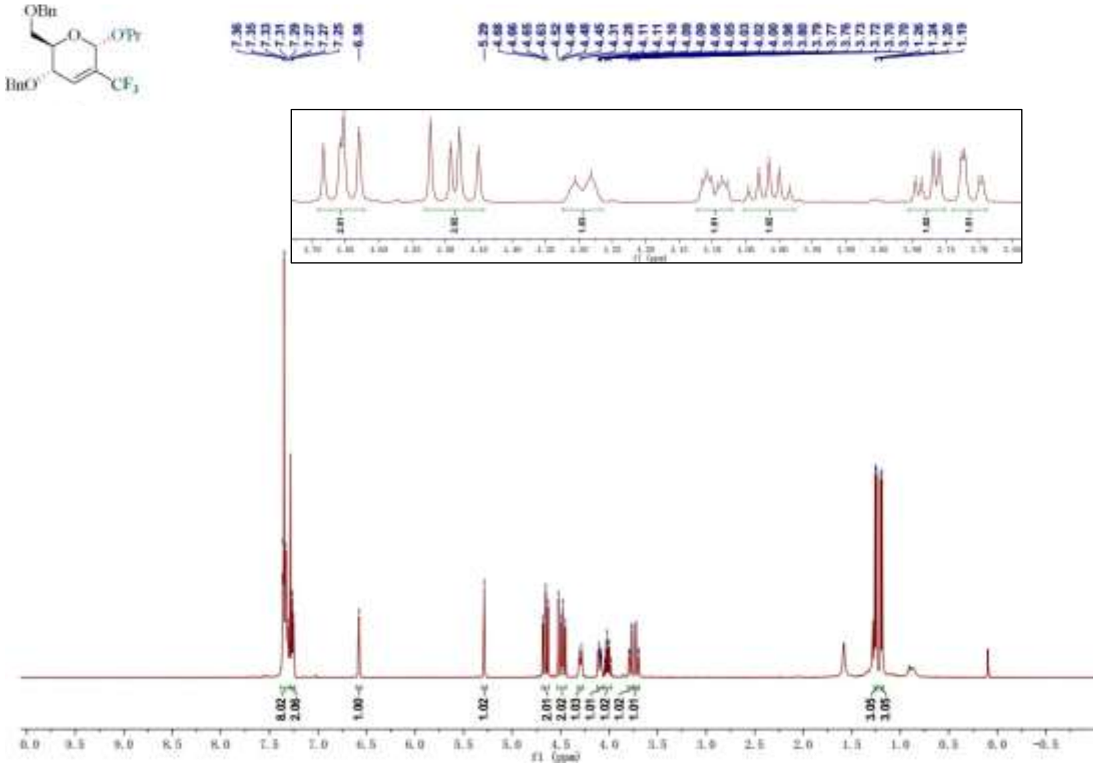

^{1}H NMR spectrum of Compound **2c**

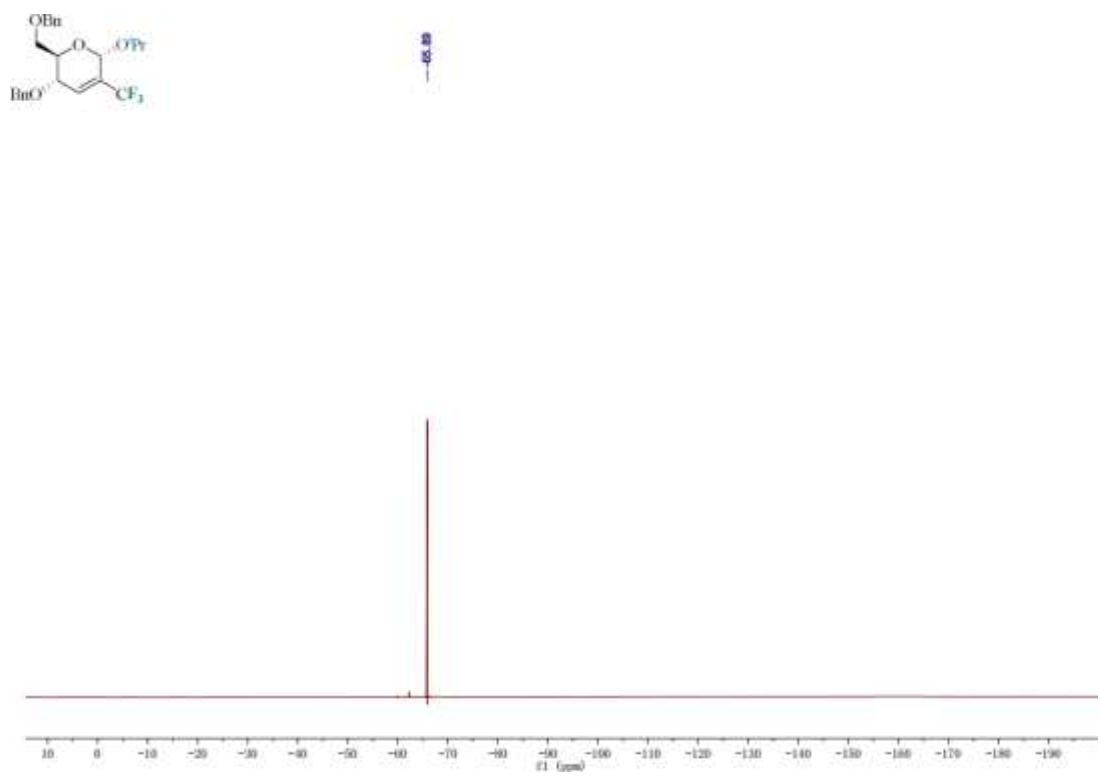

^{19}F NMR spectrum of Compound **2c**

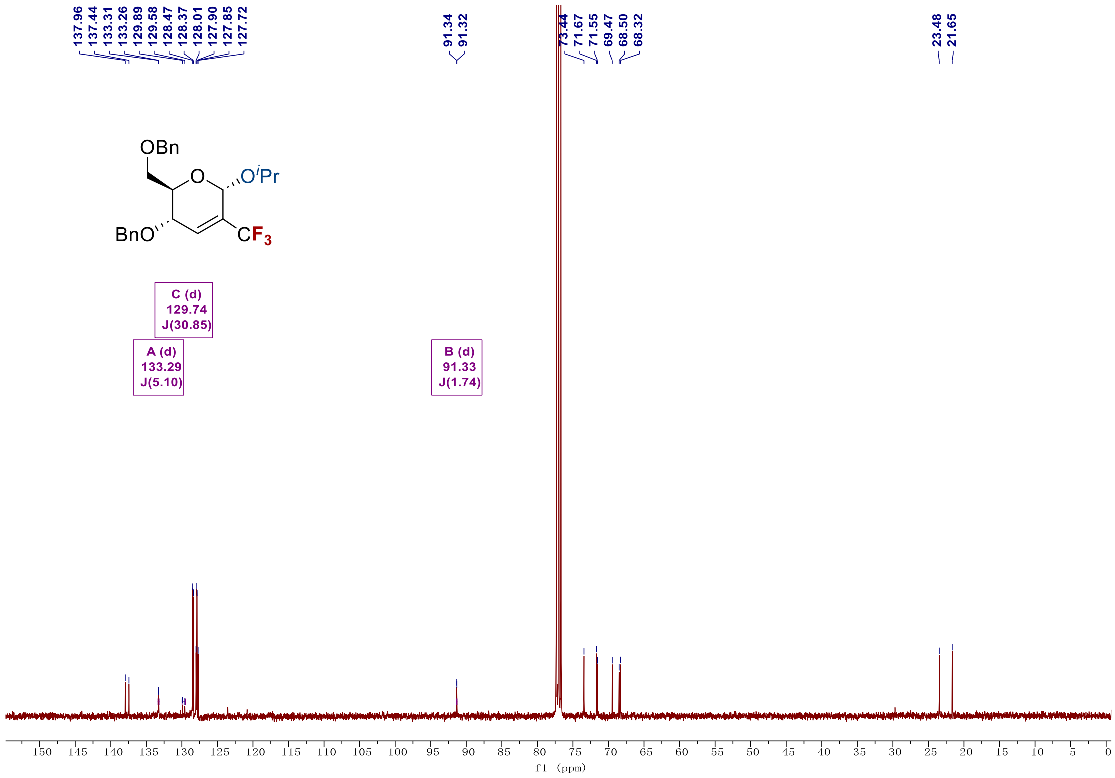

^{13}C NMR spectrum of Compound **2c**

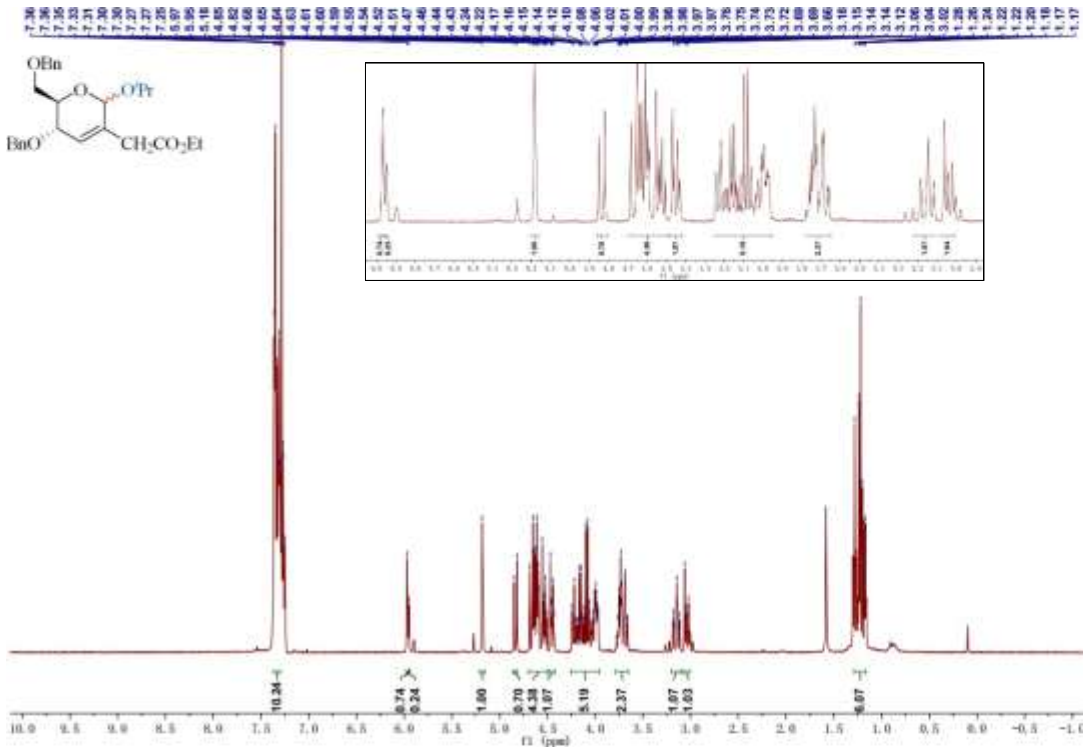

^{1}H NMR spectrum of Compound **2d**

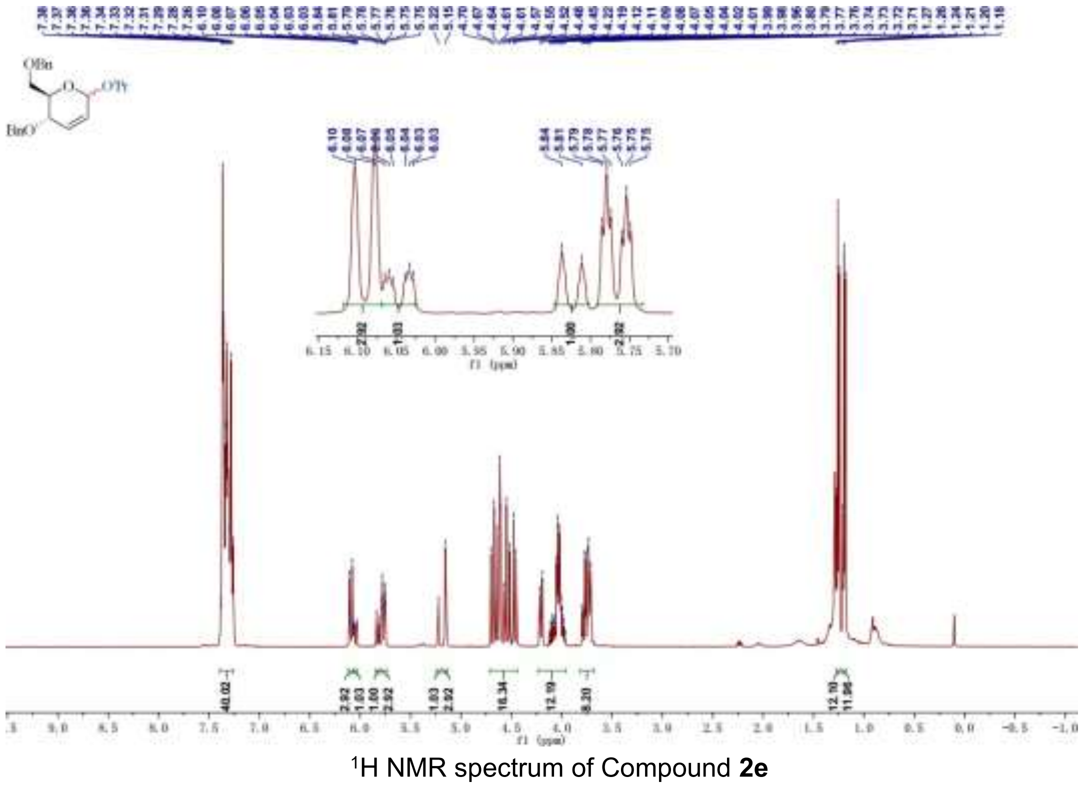

^{1}H NMR spectrum of Compound **2e**

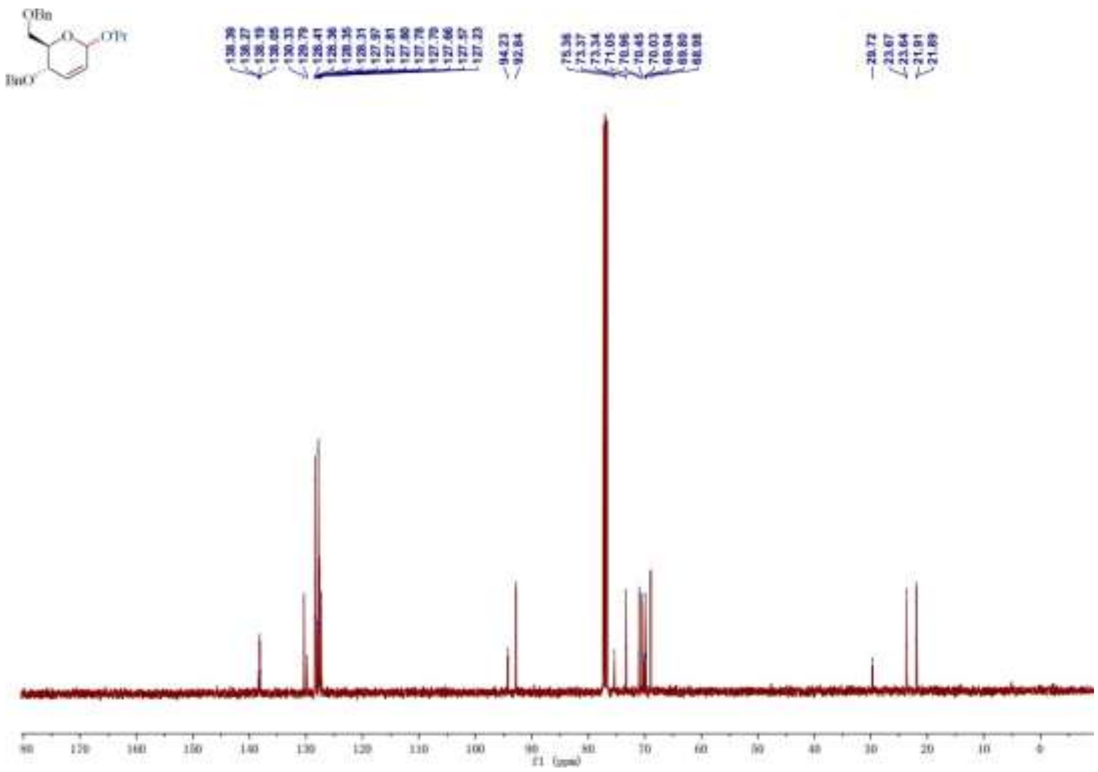

^{13}C NMR spectrum of Compound **2e**

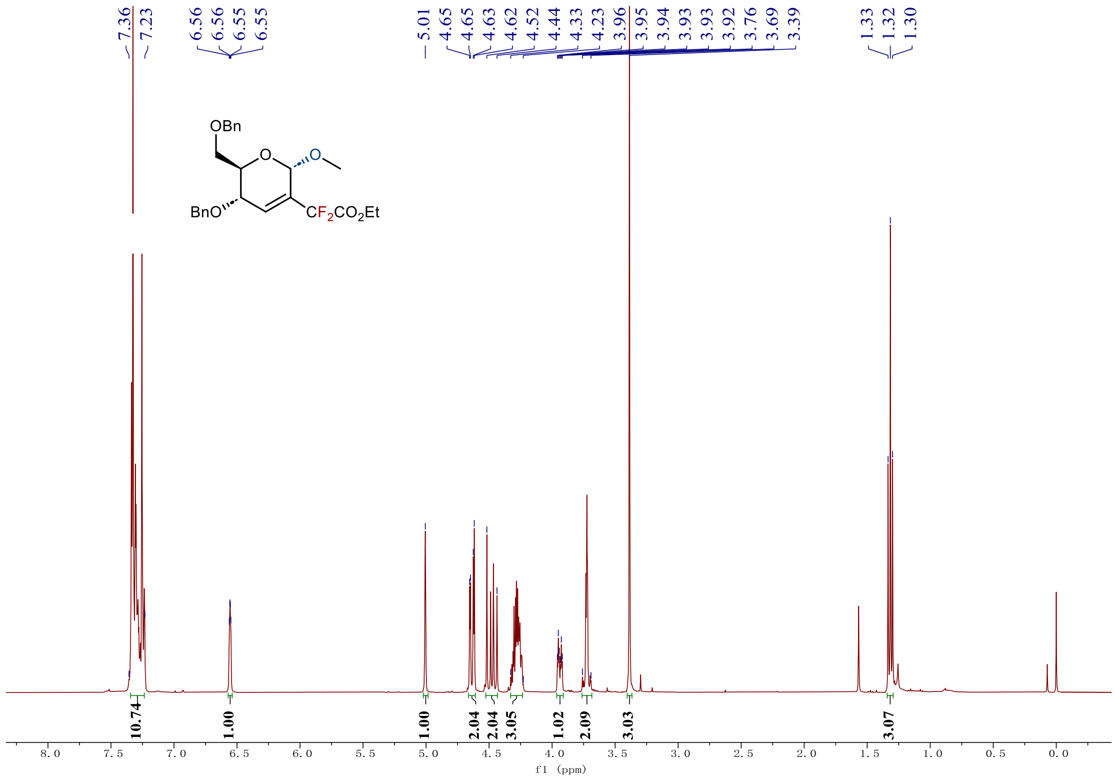

^{1}H NMR spectrum of Compound **3a**

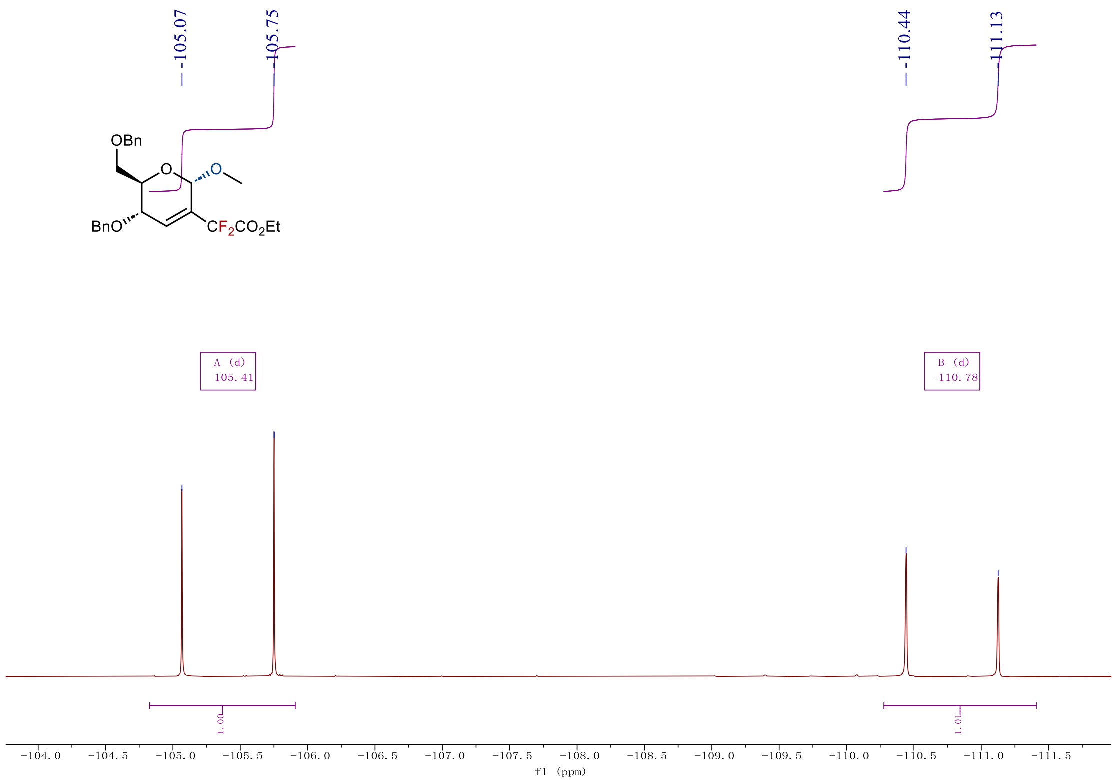

^{19}F NMR spectrum of Compound **3a**

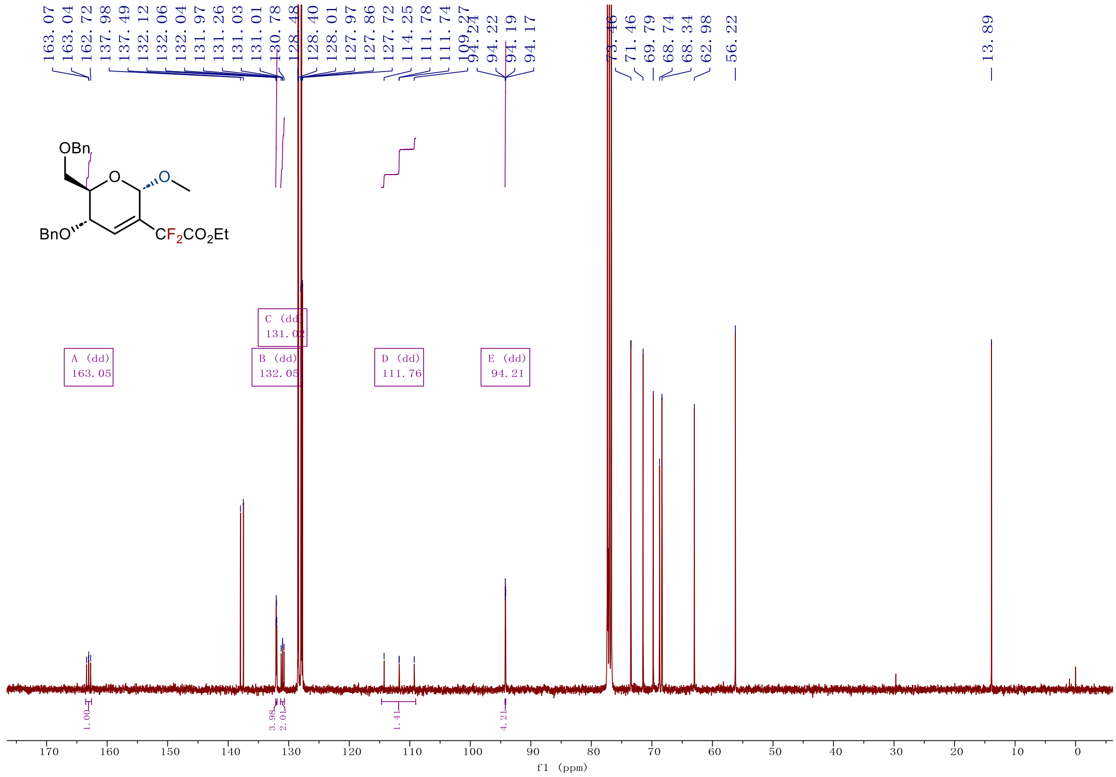

^{13}C NMR spectrum of Compound **3a**

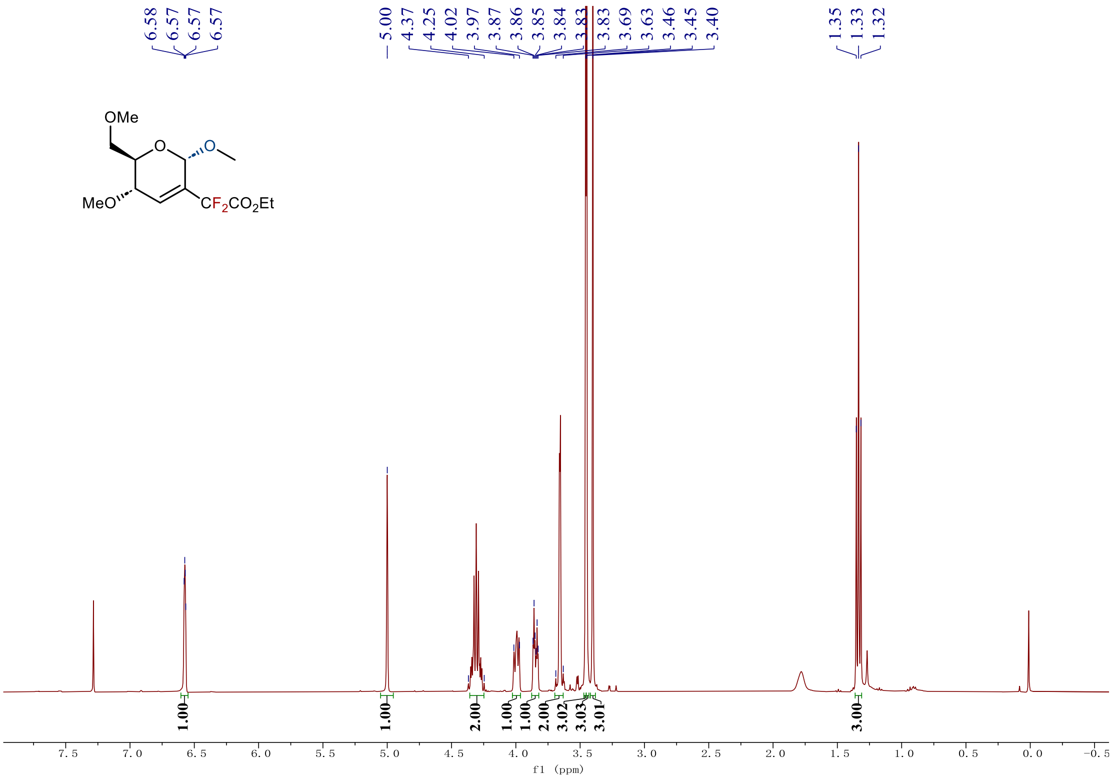

^1H NMR spectrum of Compound **3b**

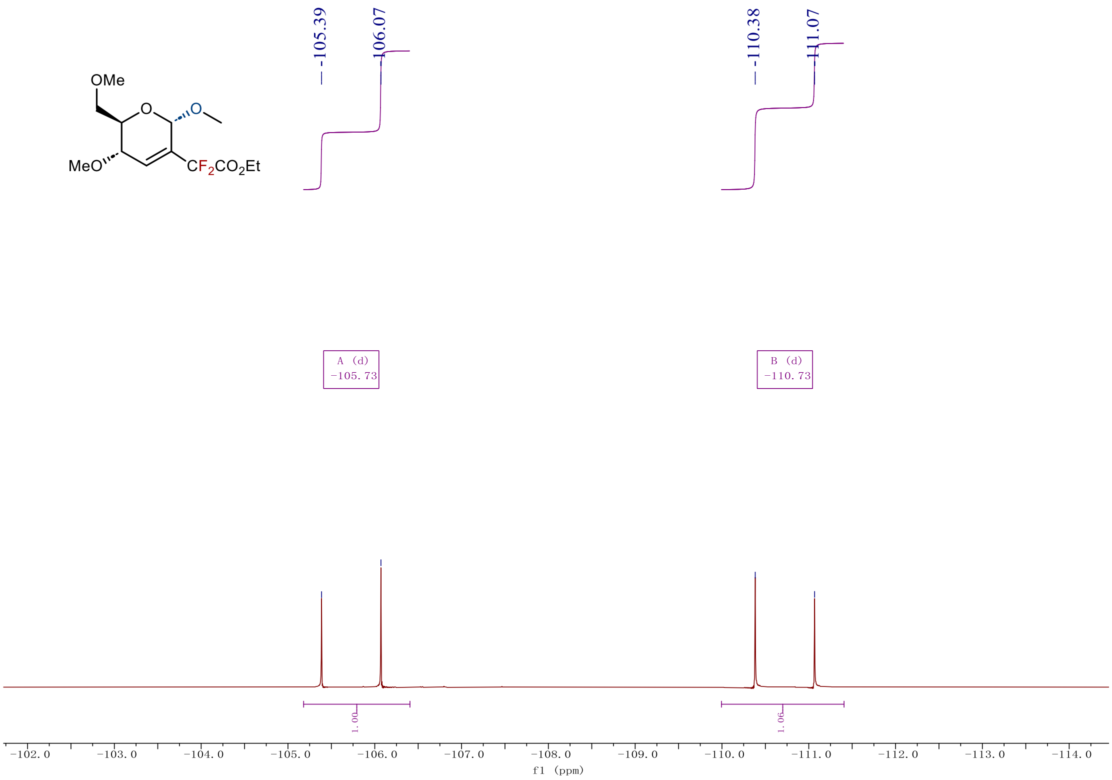

^{19}F NMR spectrum of Compound **3b**

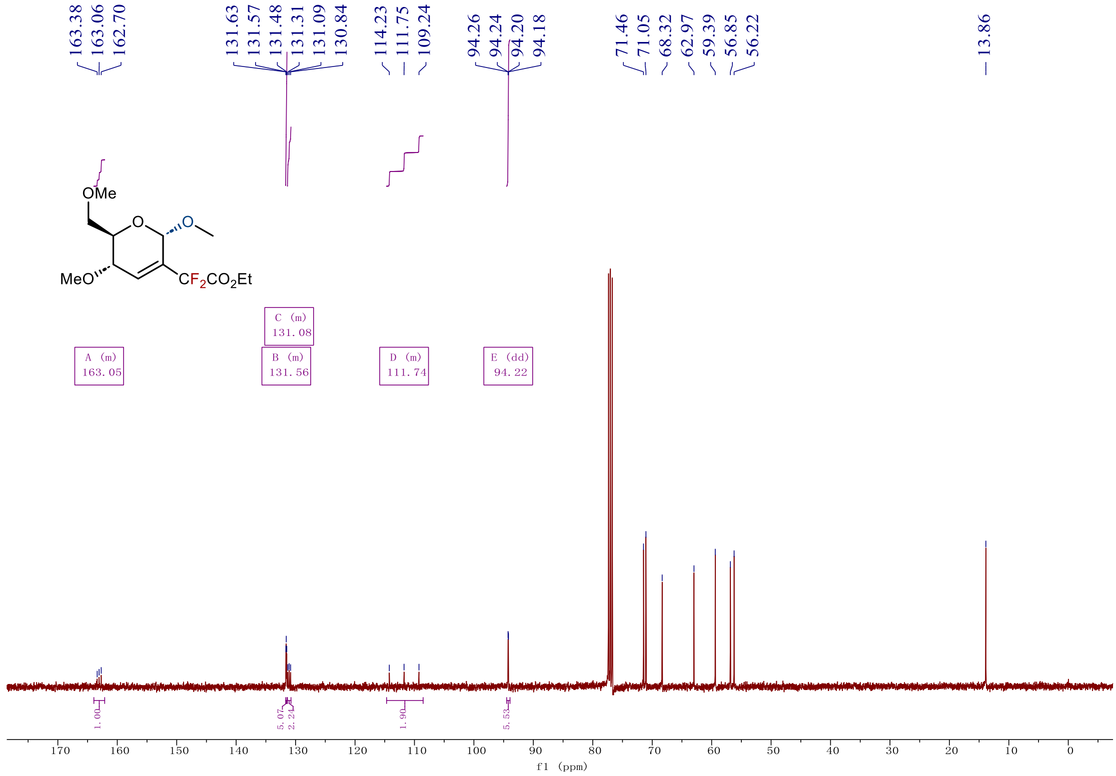

^{13}C NMR spectrum of Compound **3b**

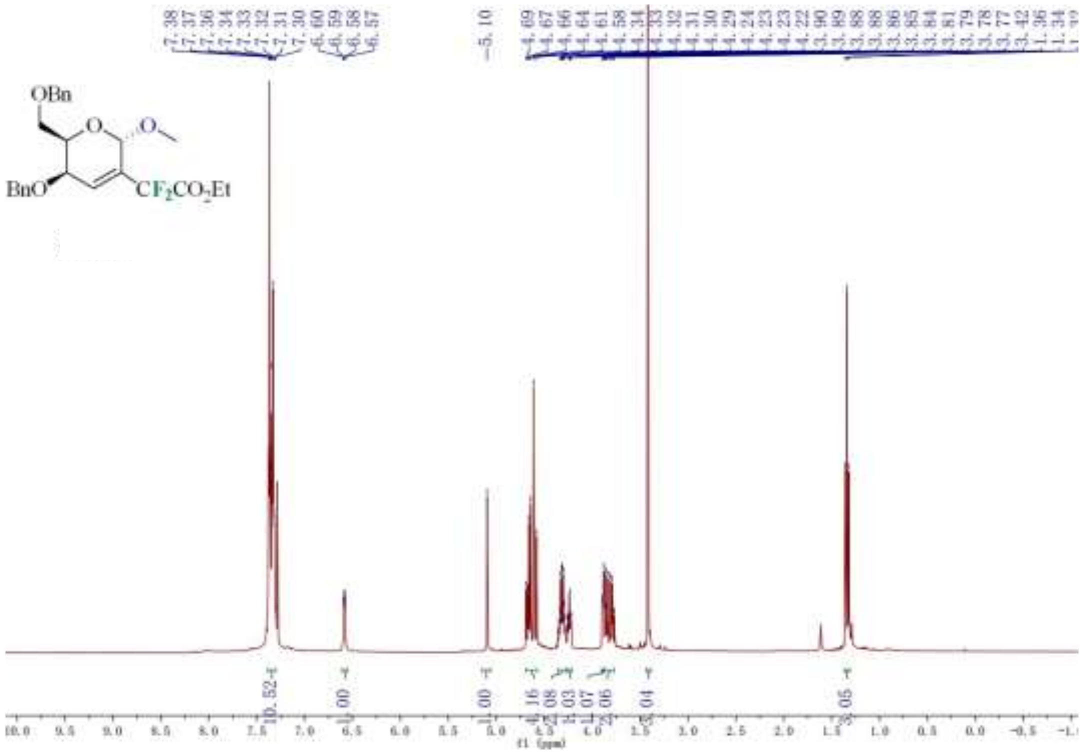

^{1}H NMR spectrum of Compound **3c**

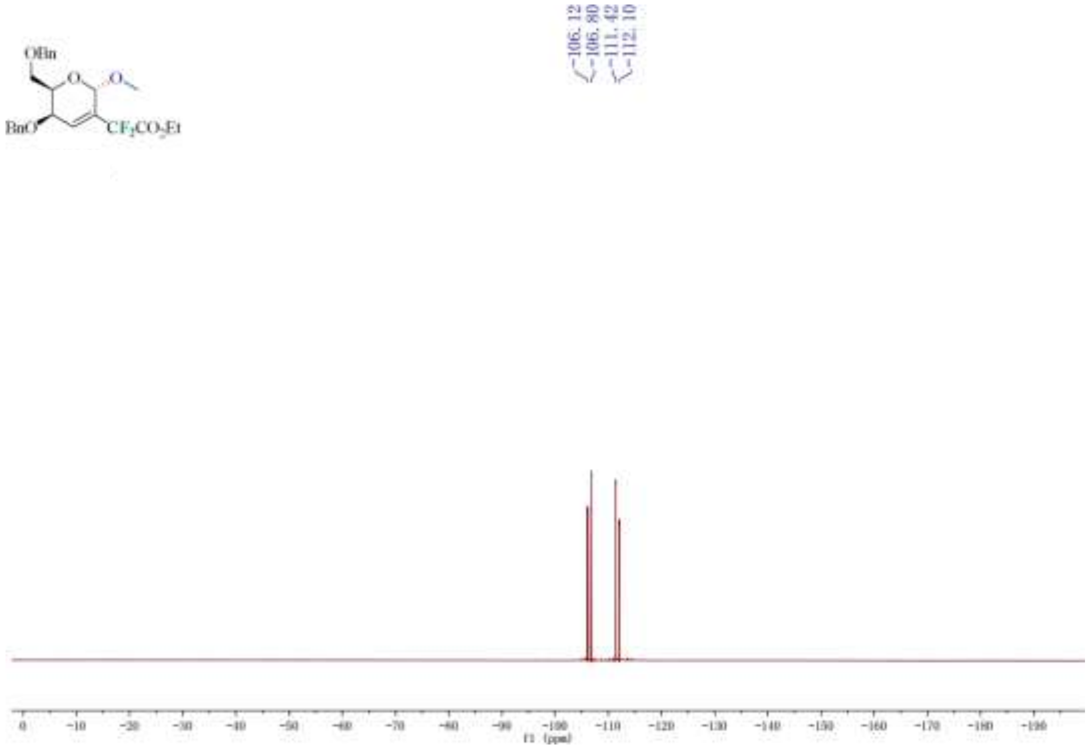

^{19}F NMR spectrum of Compound **3c**

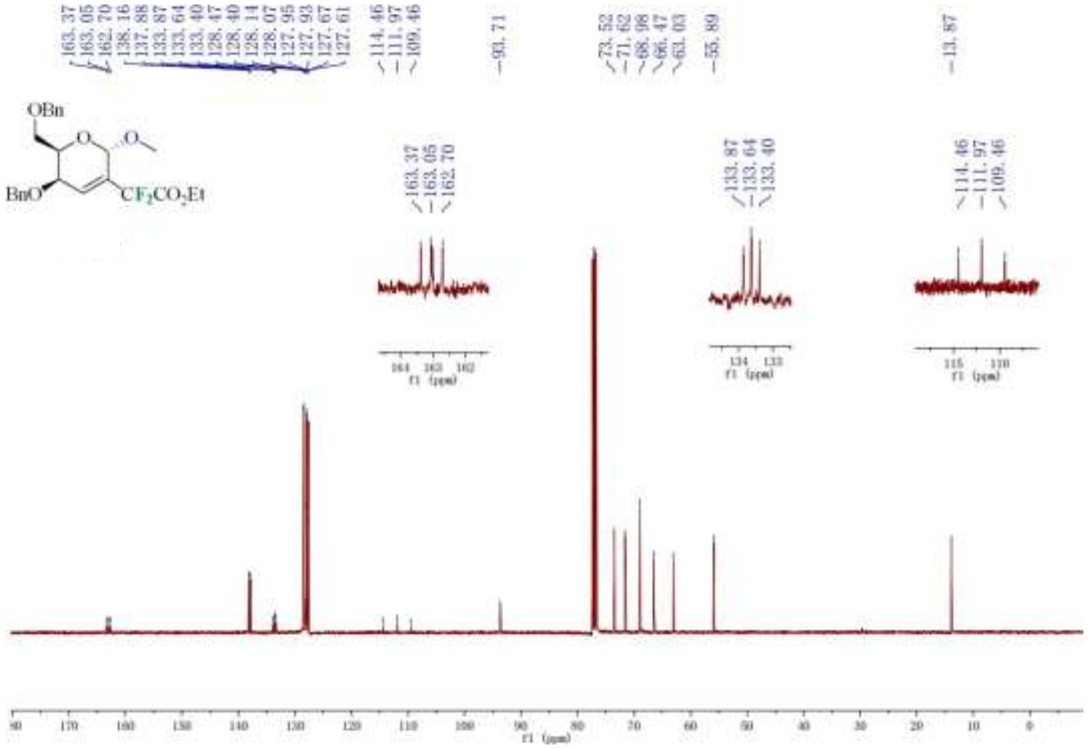

^{13}C NMR spectrum of Compound **3c**

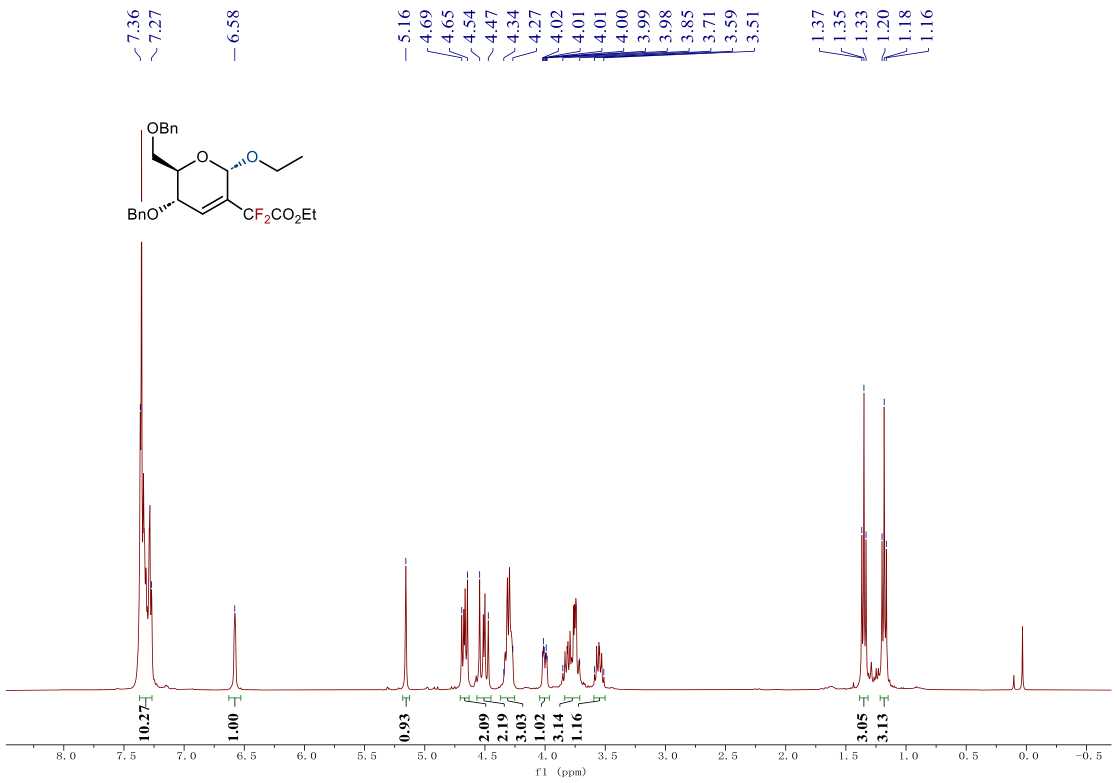

^{1}H NMR spectrum of Compound **3d**

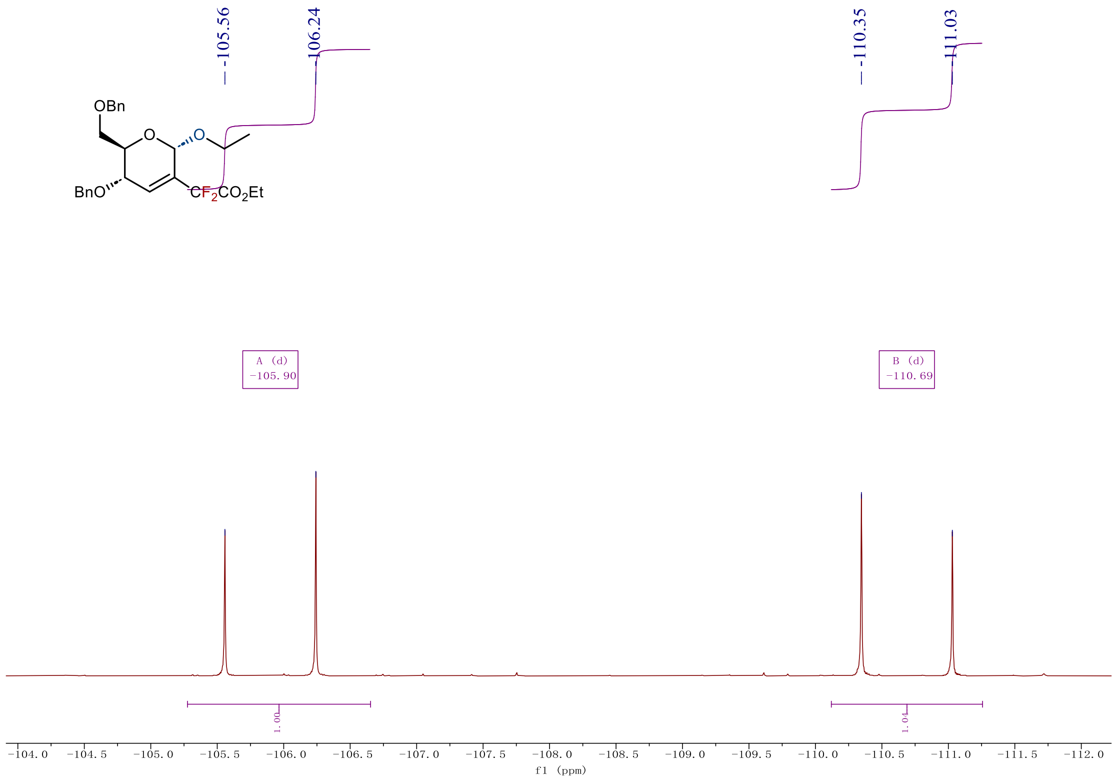

^{19}F NMR spectrum of Compound **3d**

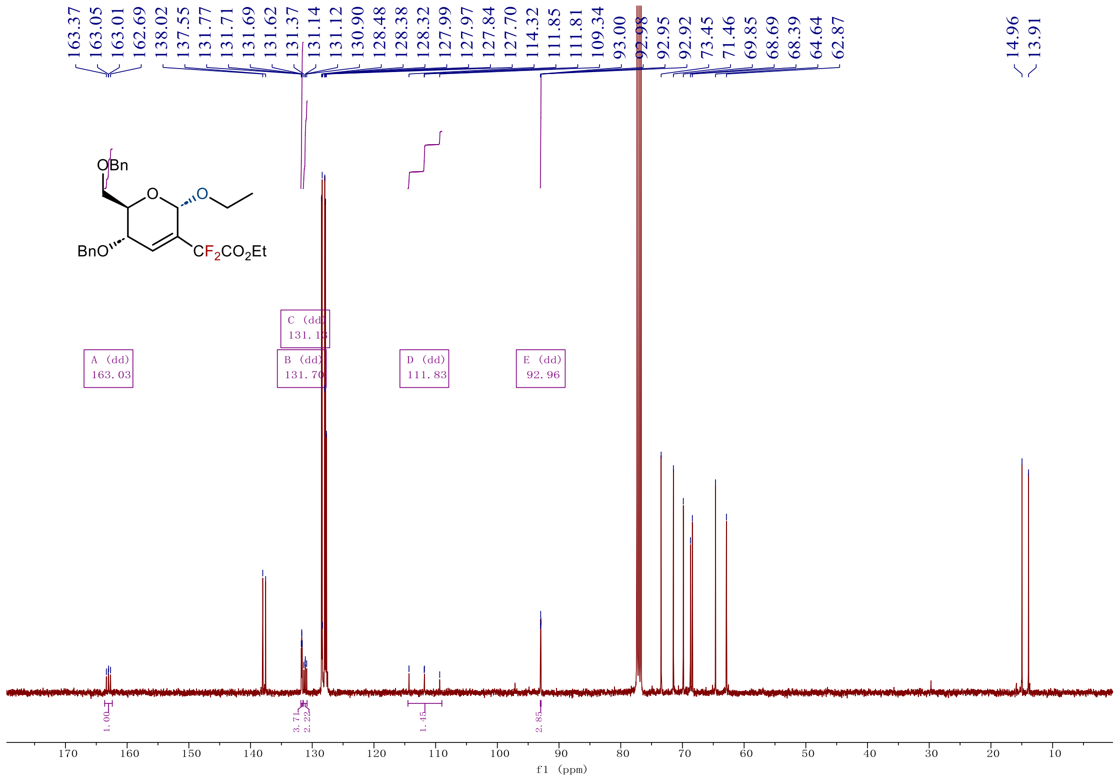

^{13}C NMR spectrum of Compound **3d**

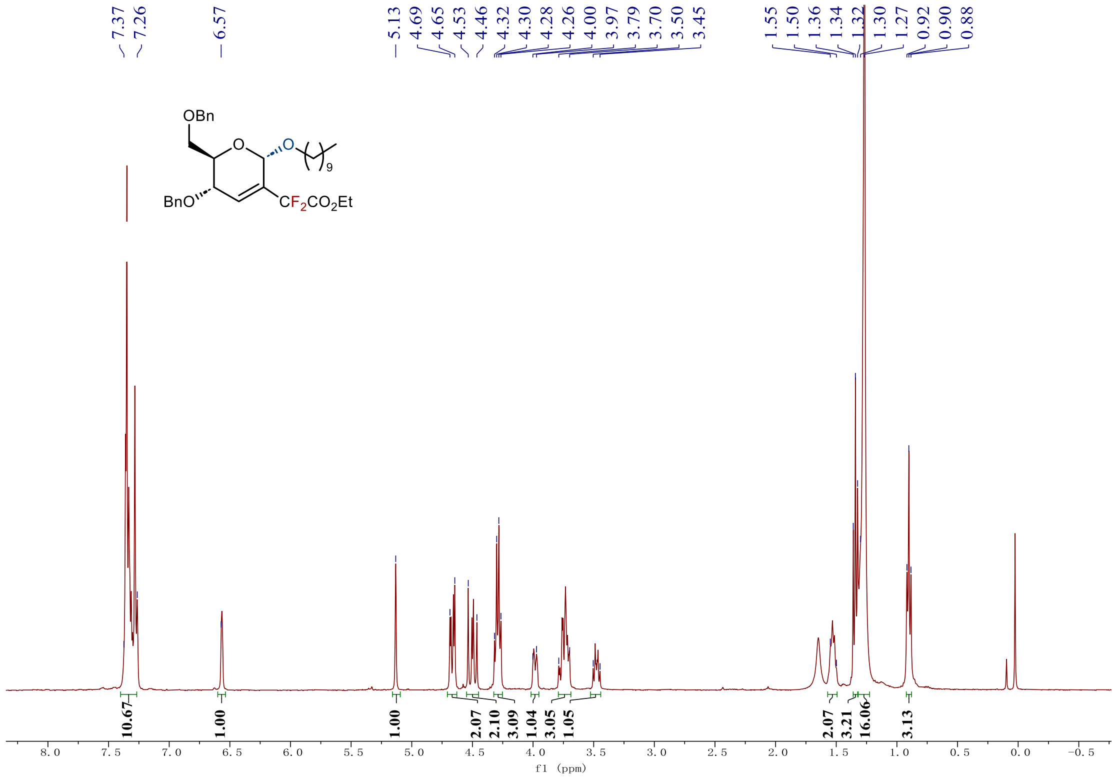

^{1}H NMR spectrum of Compound **3e**

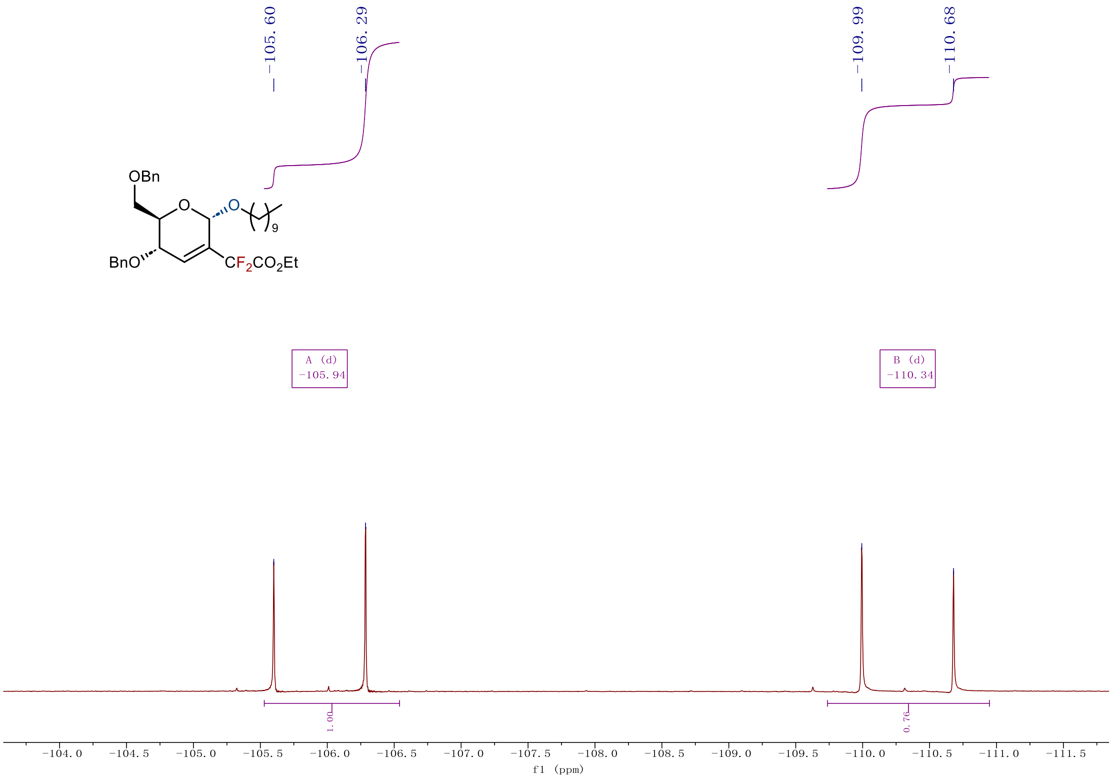

^{19}F NMR spectrum of Compound **3e**

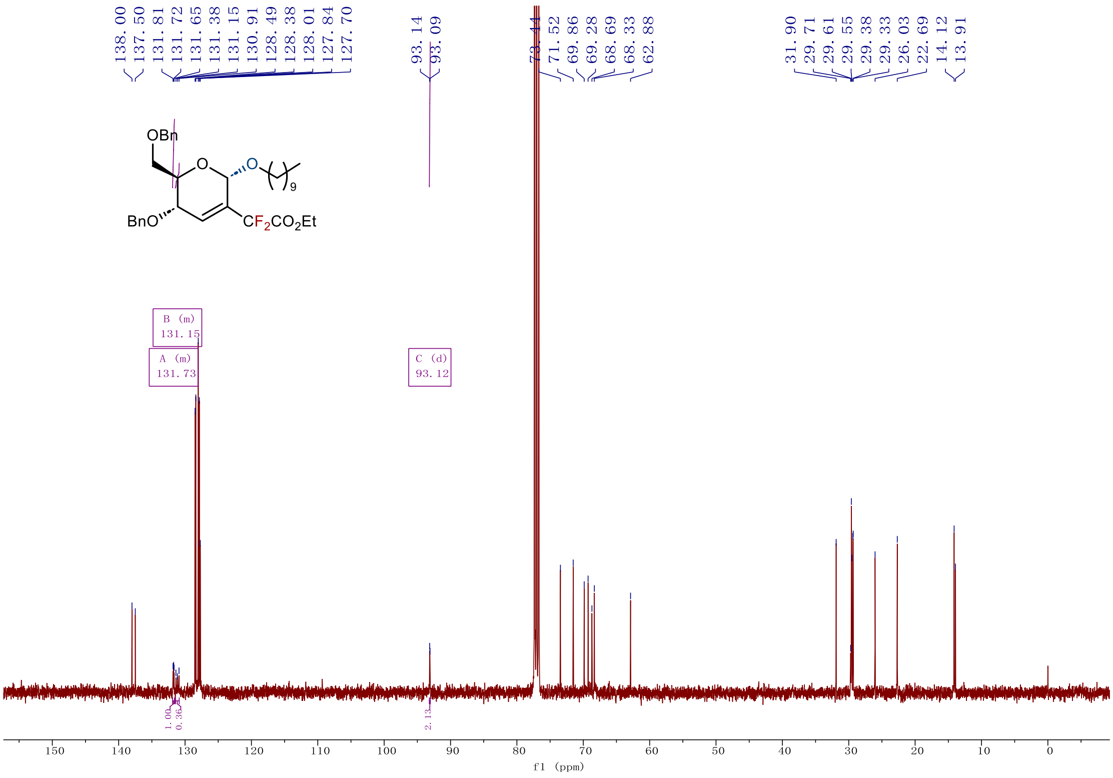

[13] C NMR spectrum of Compound **3e**

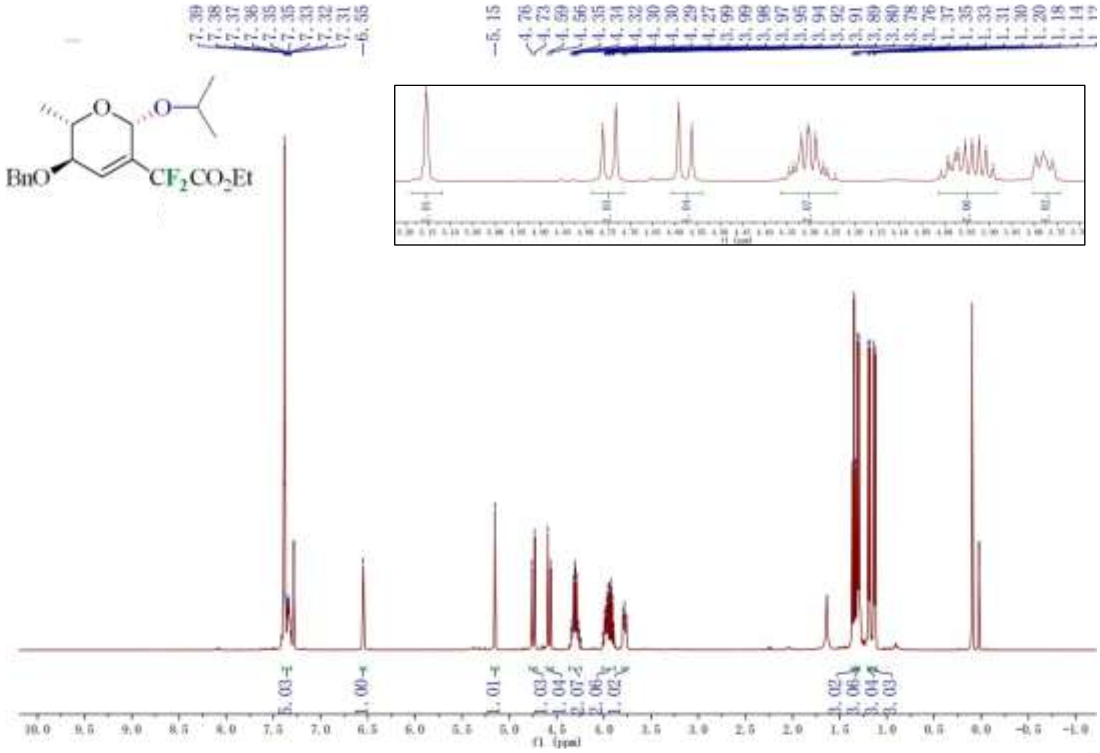

[1] H NMR spectrum of Compound **3f**

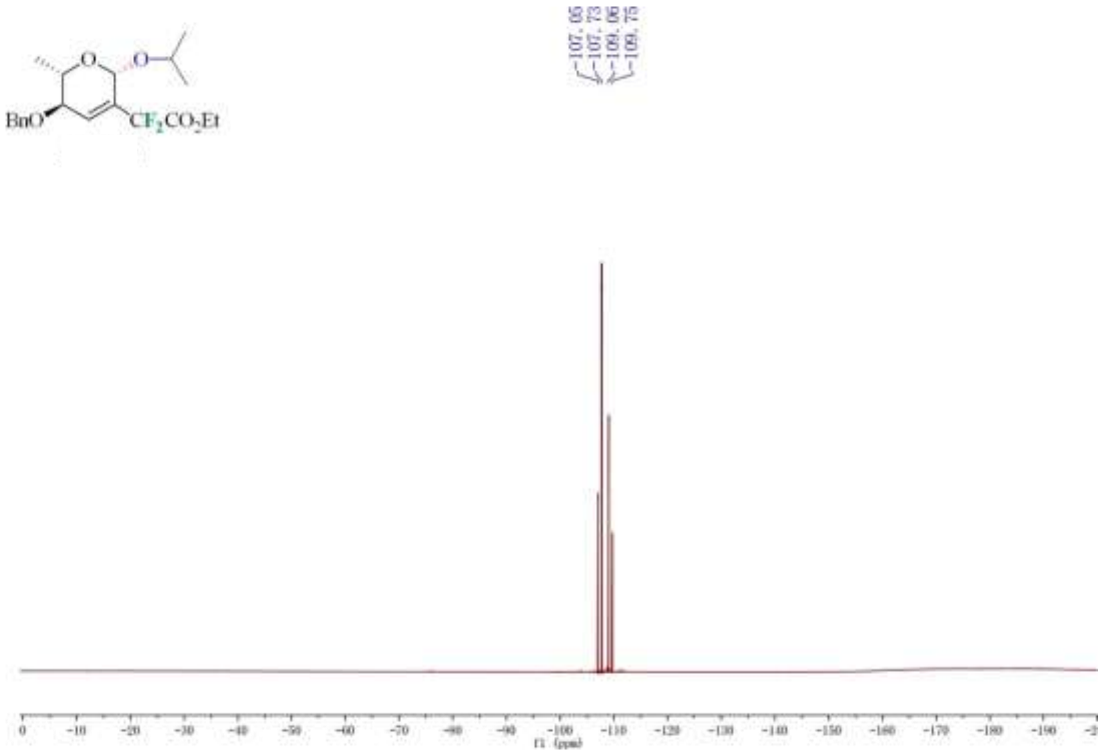

[19] F NMR spectrum of Compound **3f**

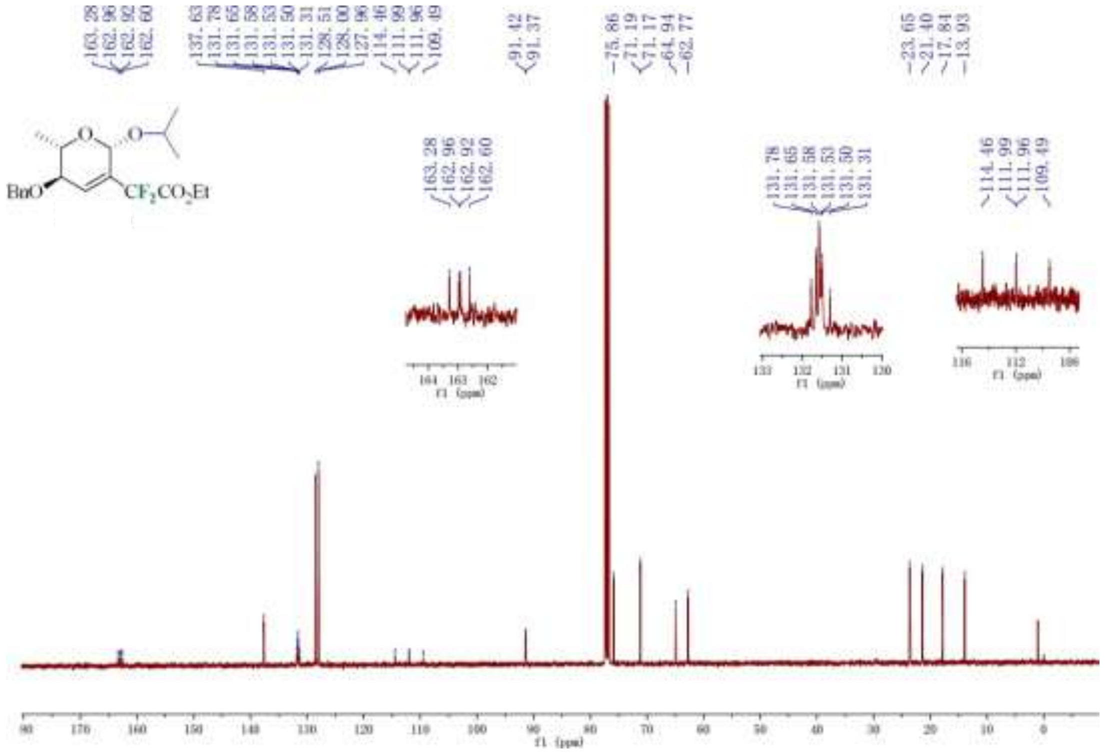

^{13}C NMR spectrum of Compound **3f**

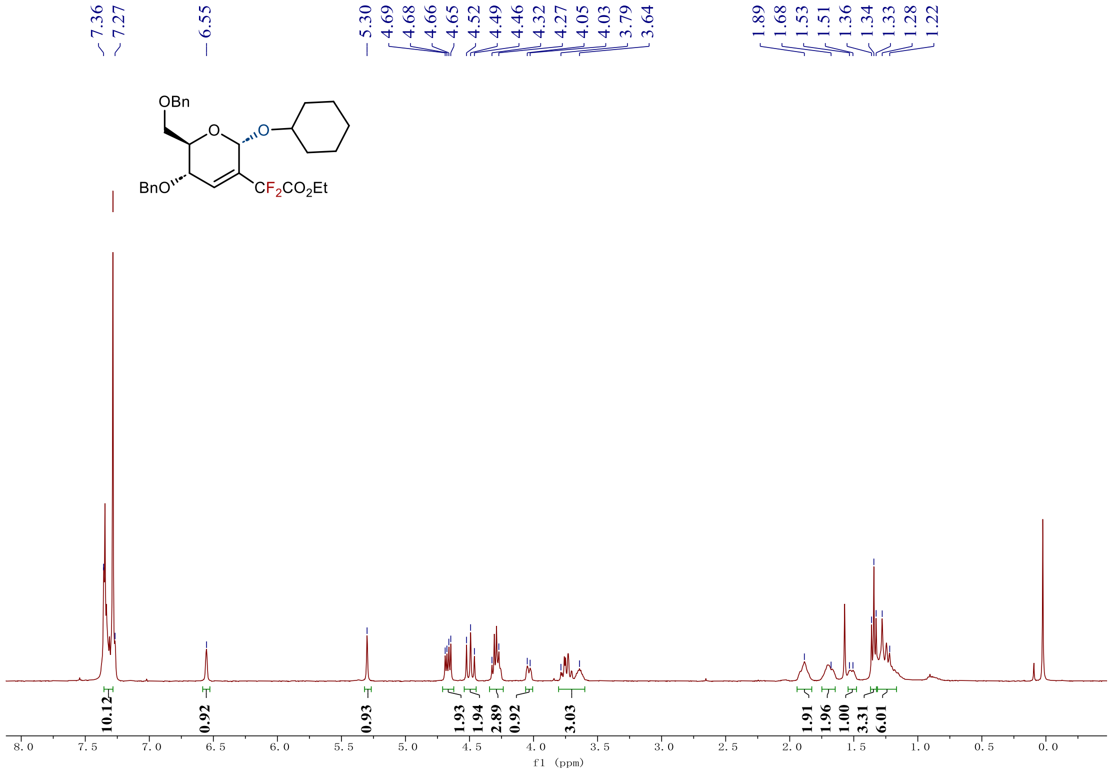

^{1}H NMR spectrum of Compound **3g**

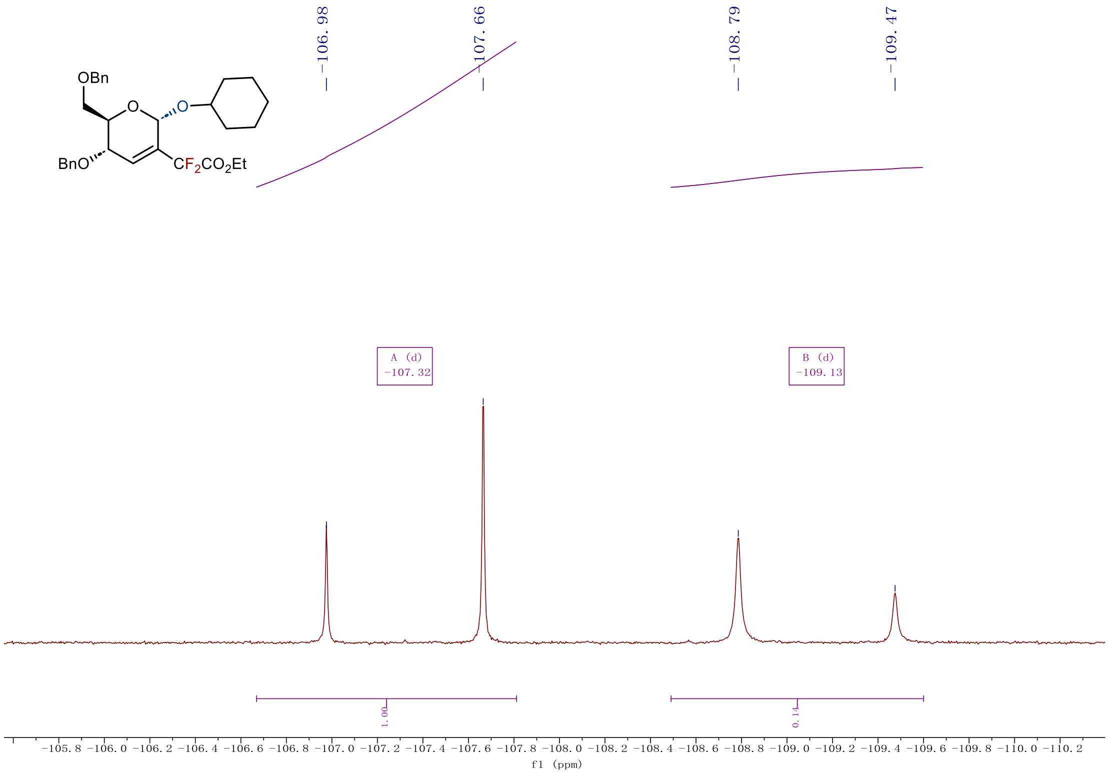

^{19}F NMR spectrum of Compound **3g**

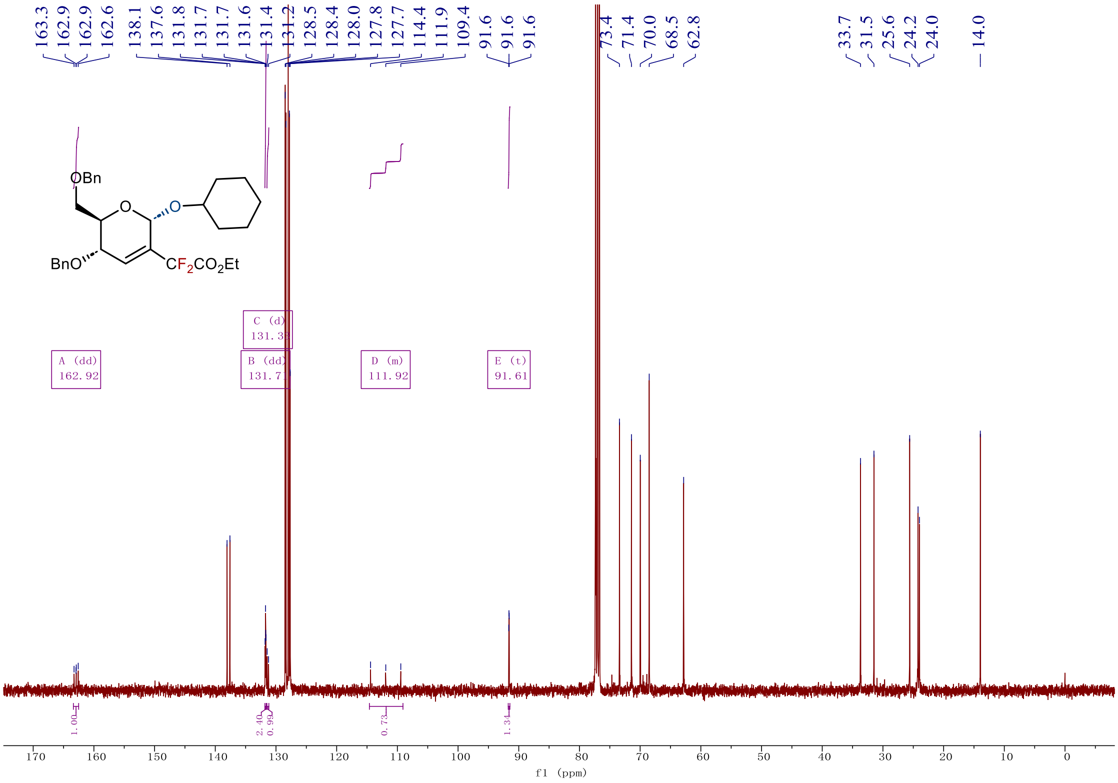

^{13}C NMR spectrum of Compound **3g**

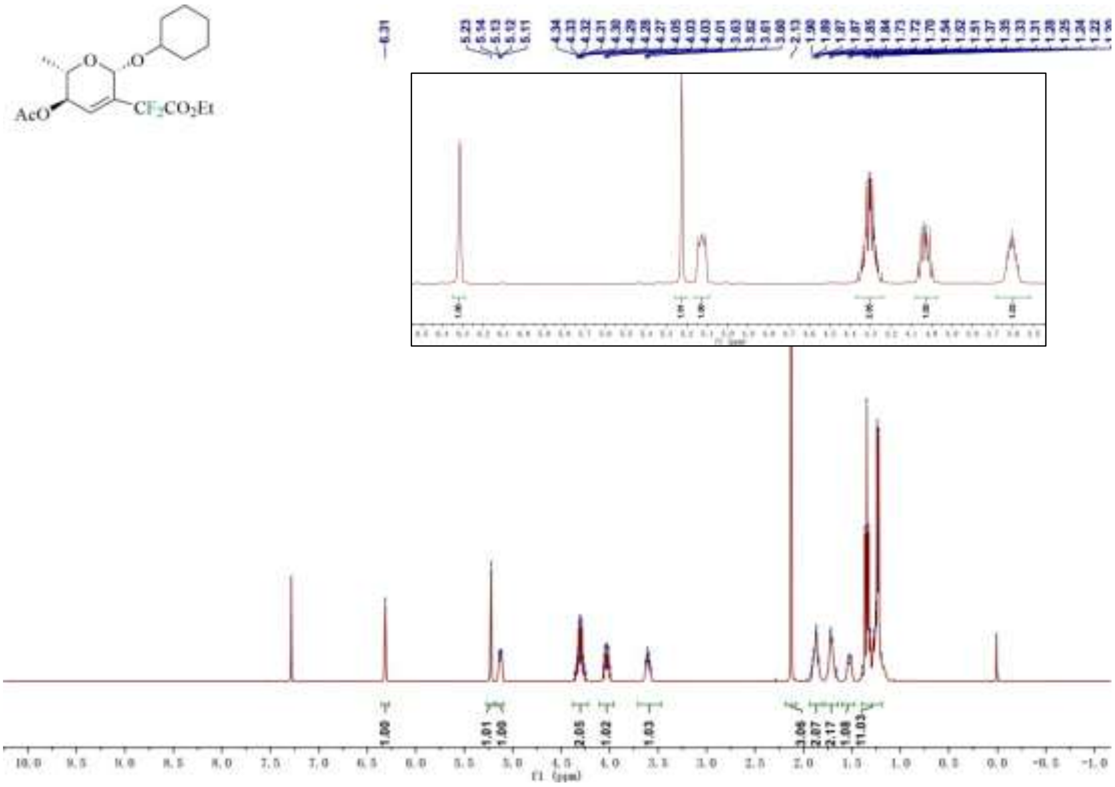

^{1}H NMR spectrum of Compound **3h**

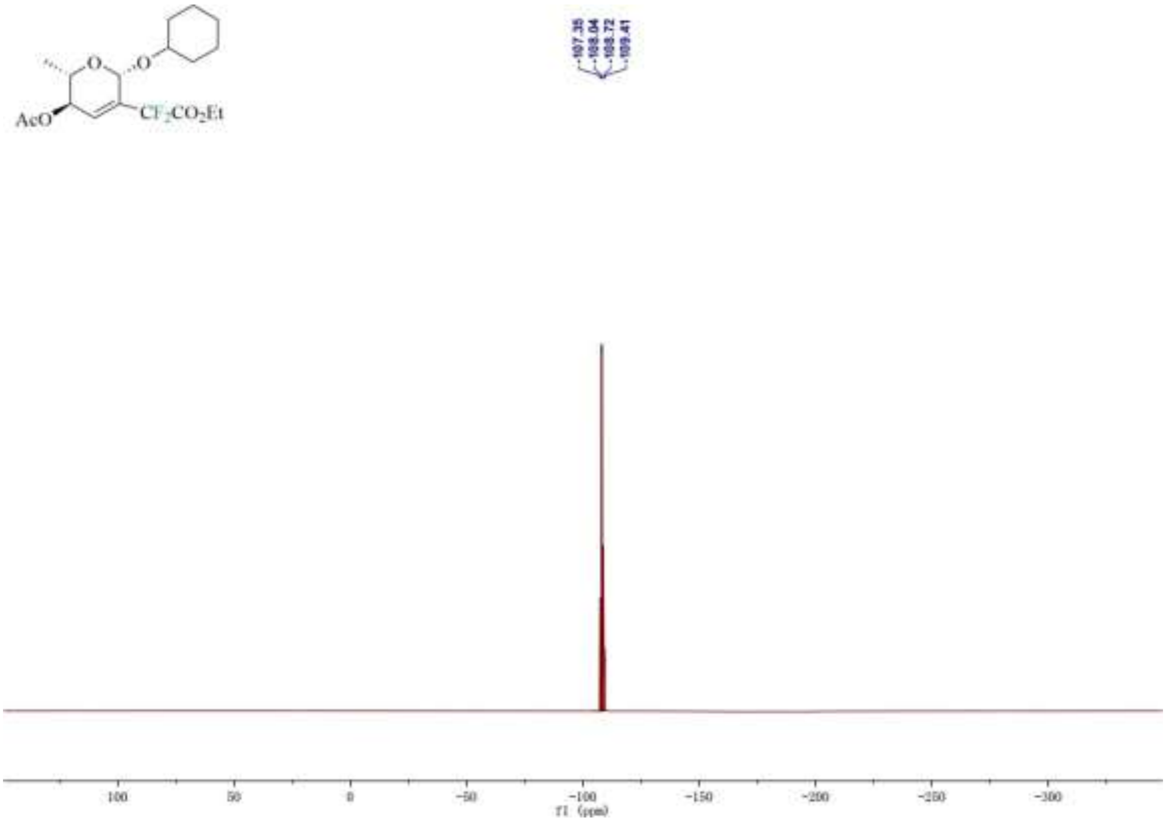

^{19}F NMR spectrum of Compound **3h**

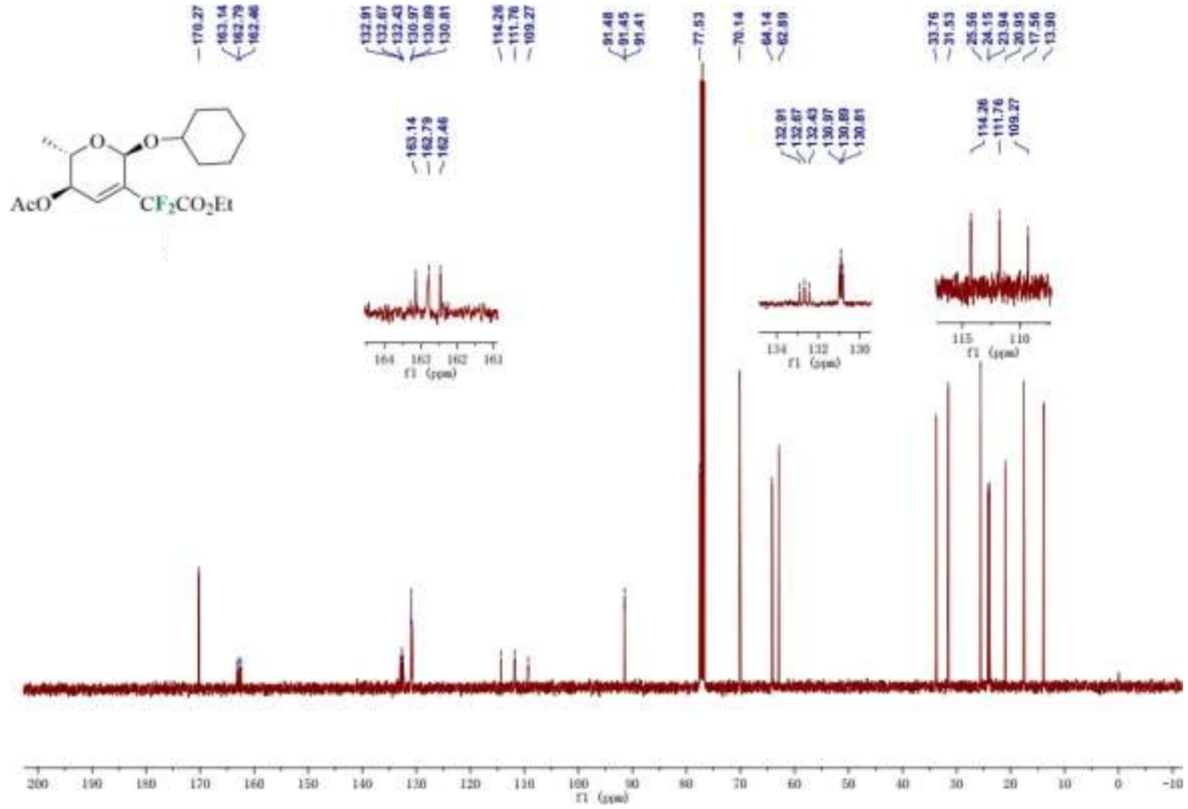

^{13}C NMR spectrum of Compound **3h**

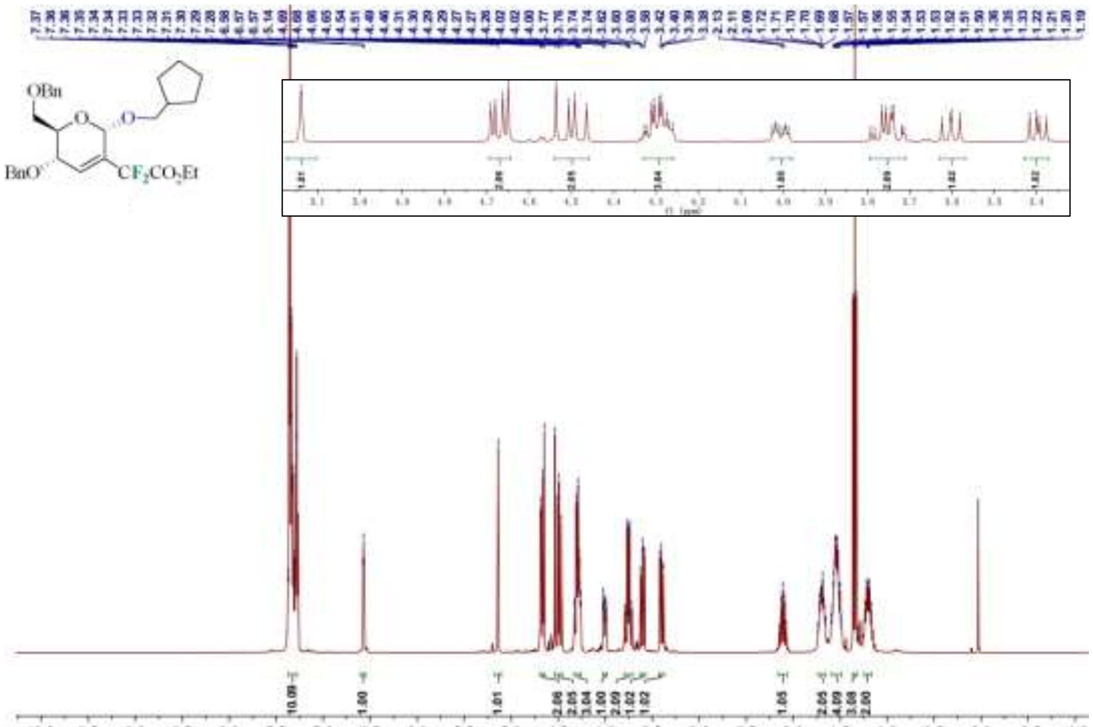

^{1}H NMR spectrum of Compound **3i**

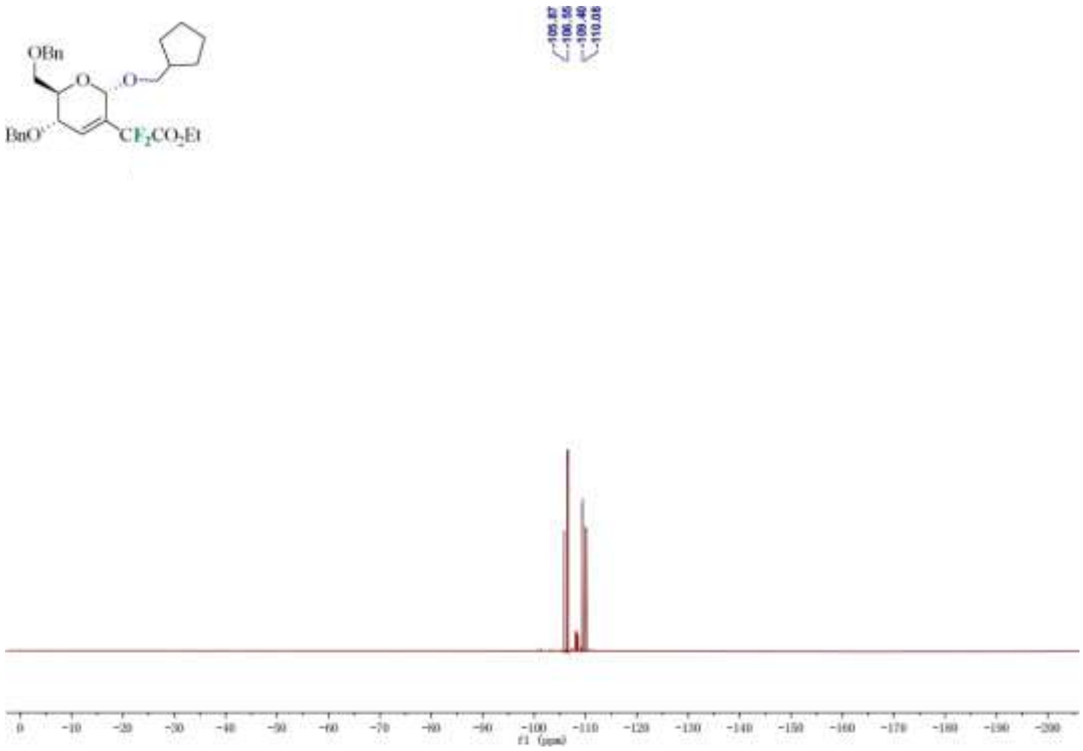

^{19}F NMR spectrum of Compound **3i**

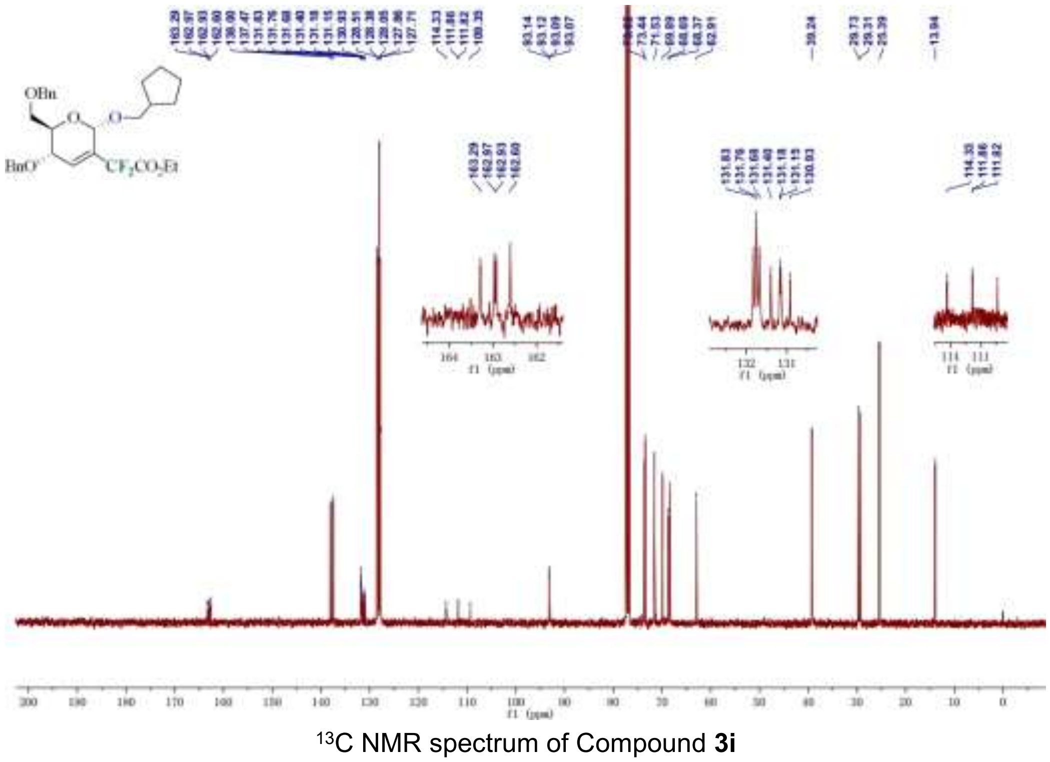

^{13}C NMR spectrum of Compound **3i**

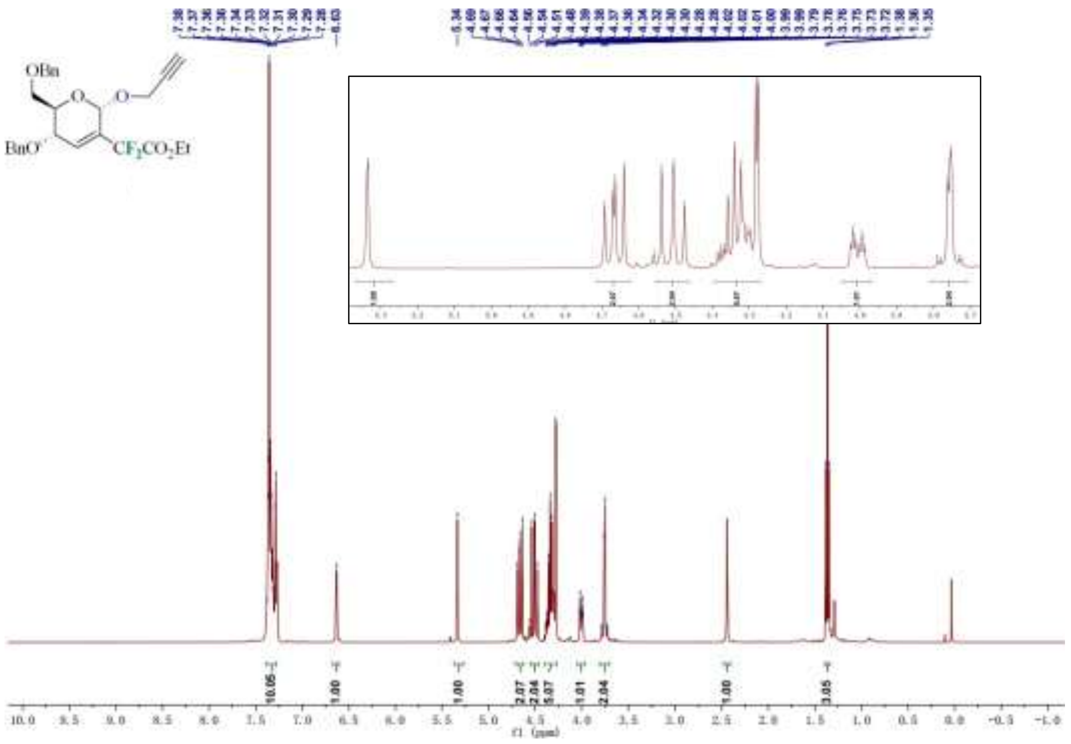

^{1}H NMR spectrum of Compound **3j**

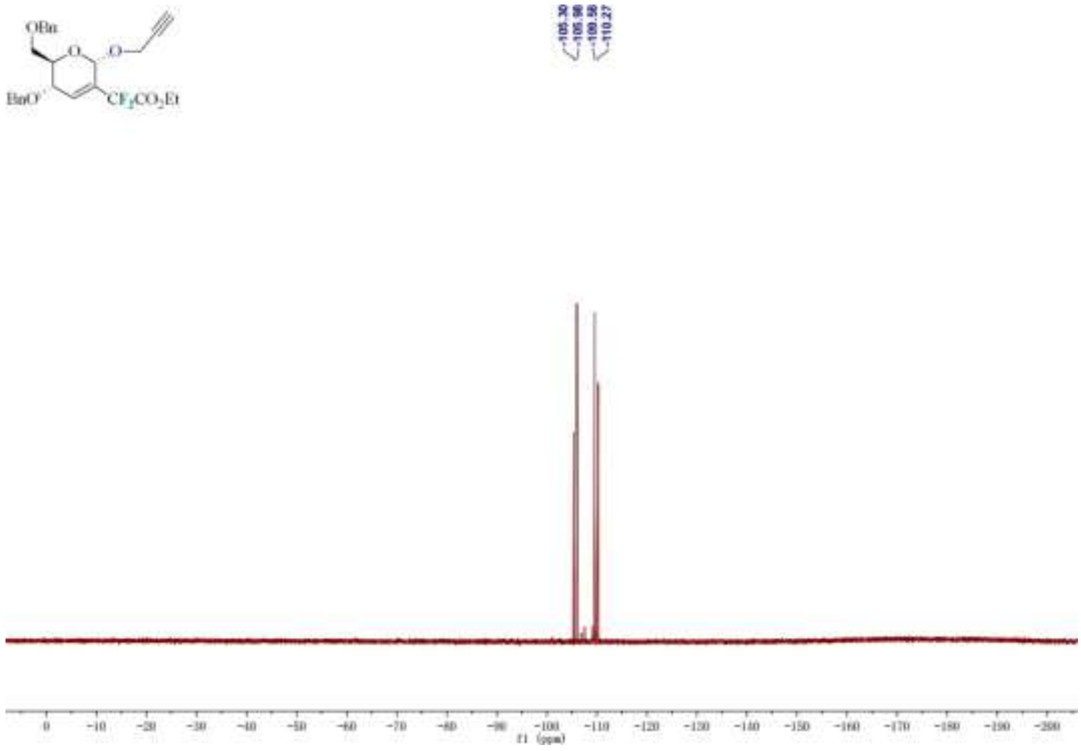

^{19}F NMR spectrum of Compound **3j**

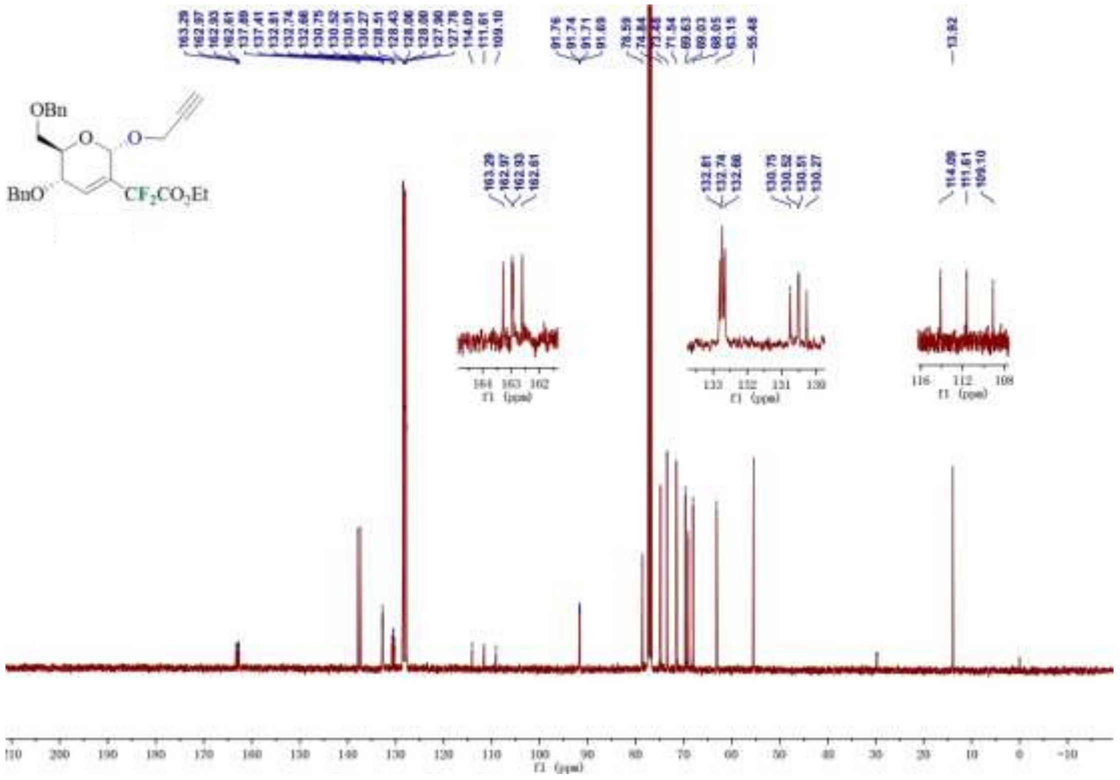

^{13}C NMR spectrum of Compound **3j**

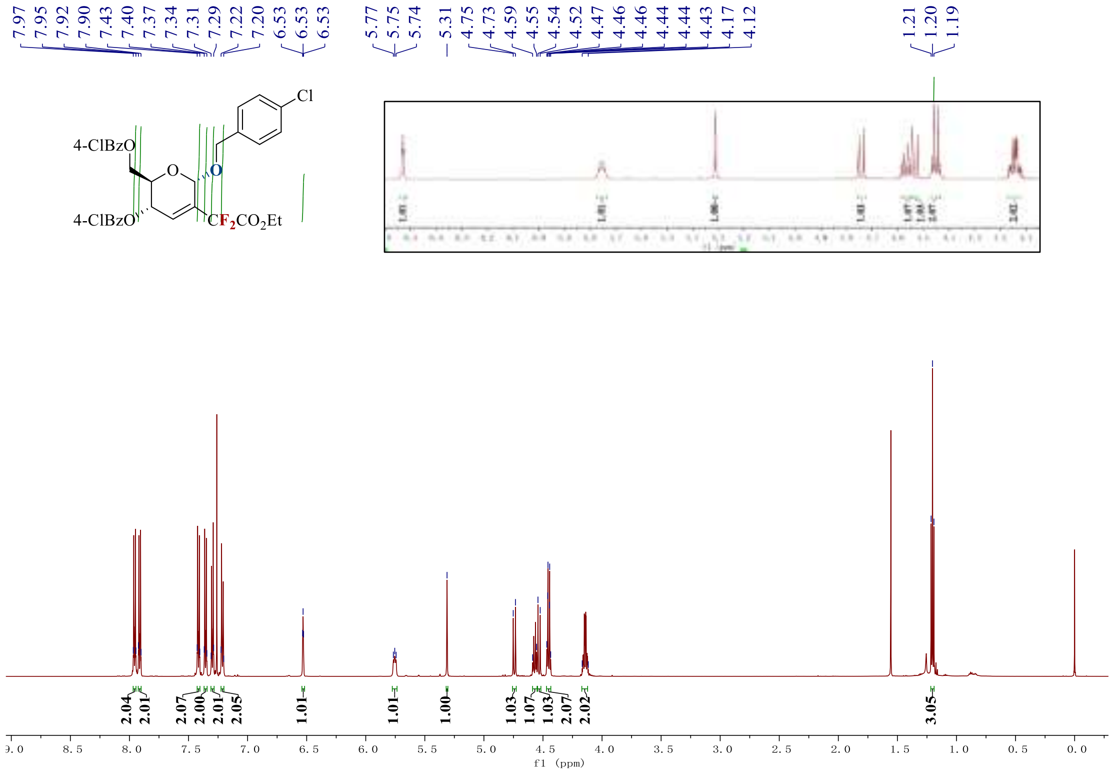

^1H NMR spectrum of Compound **3k**

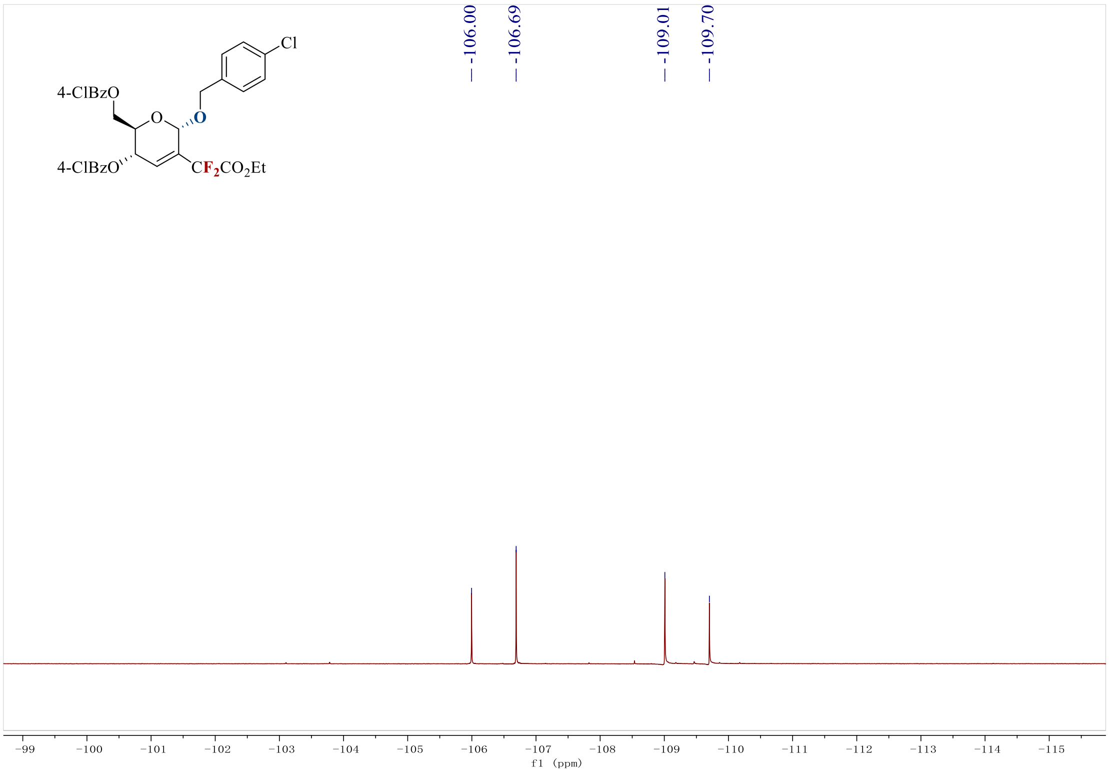

^{19}F NMR spectrum of Compound **3k**

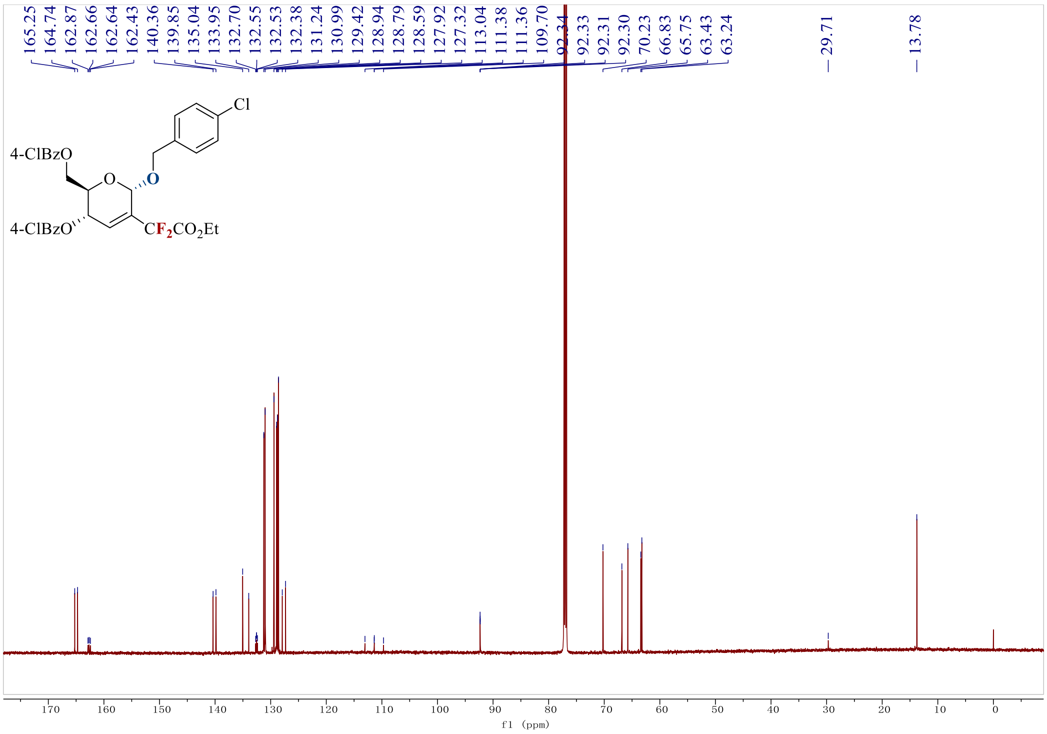

^{13}C NMR spectrum of Compound **3k**

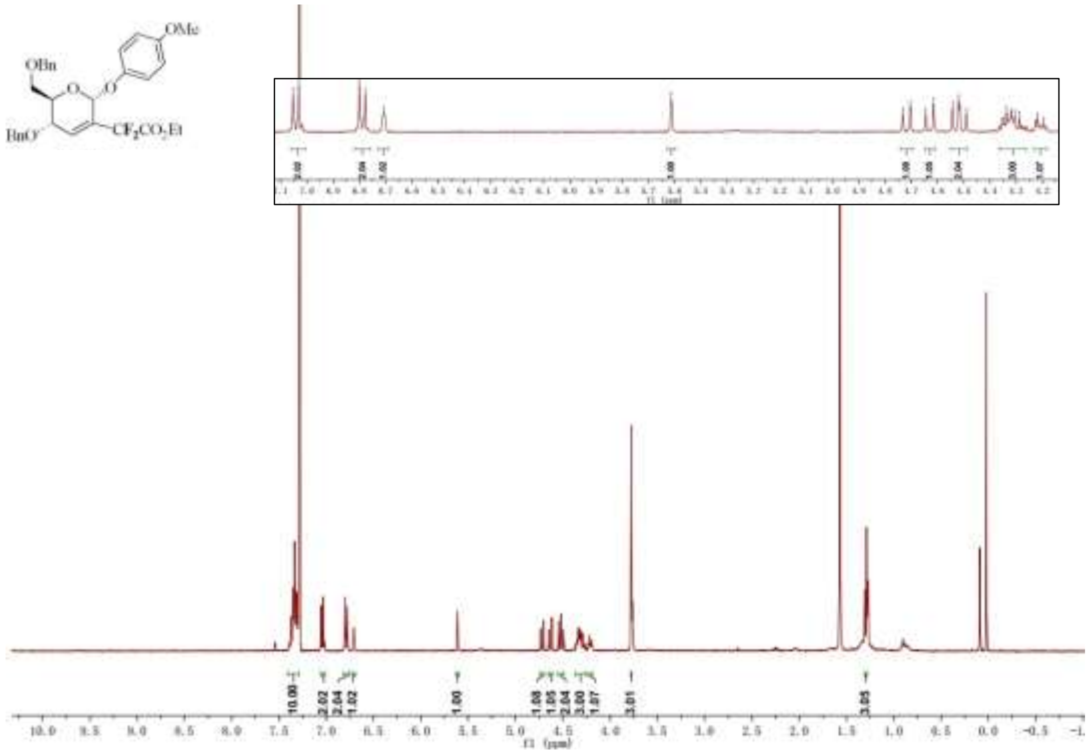

^{1}H NMR spectrum of Compound **3l**

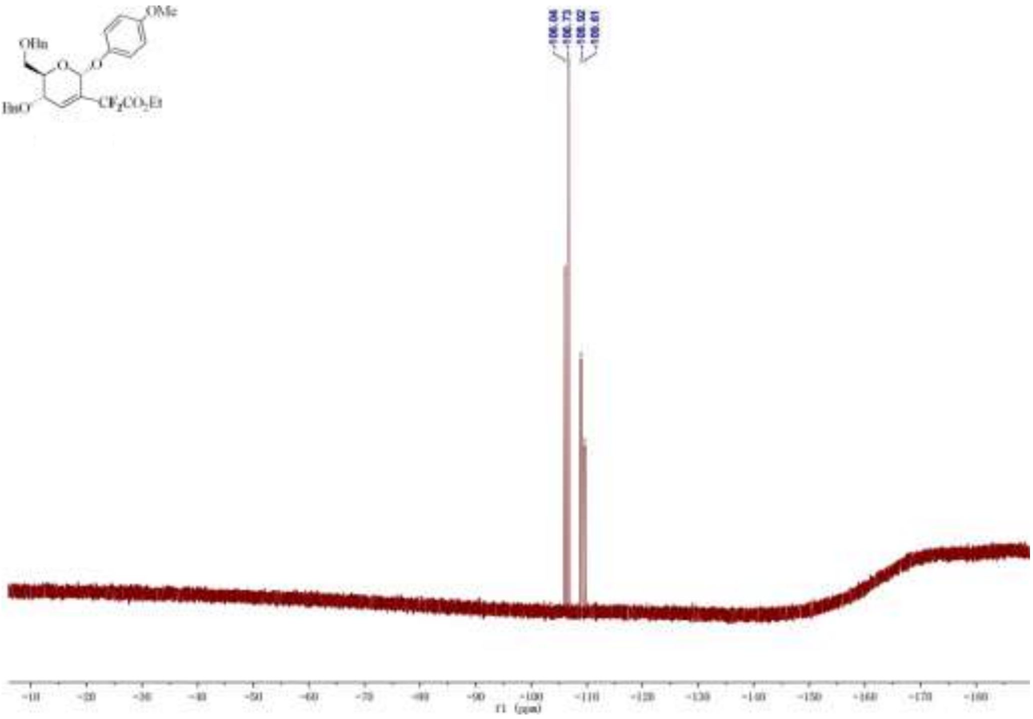

^{19}F NMR spectrum of Compound **3l**

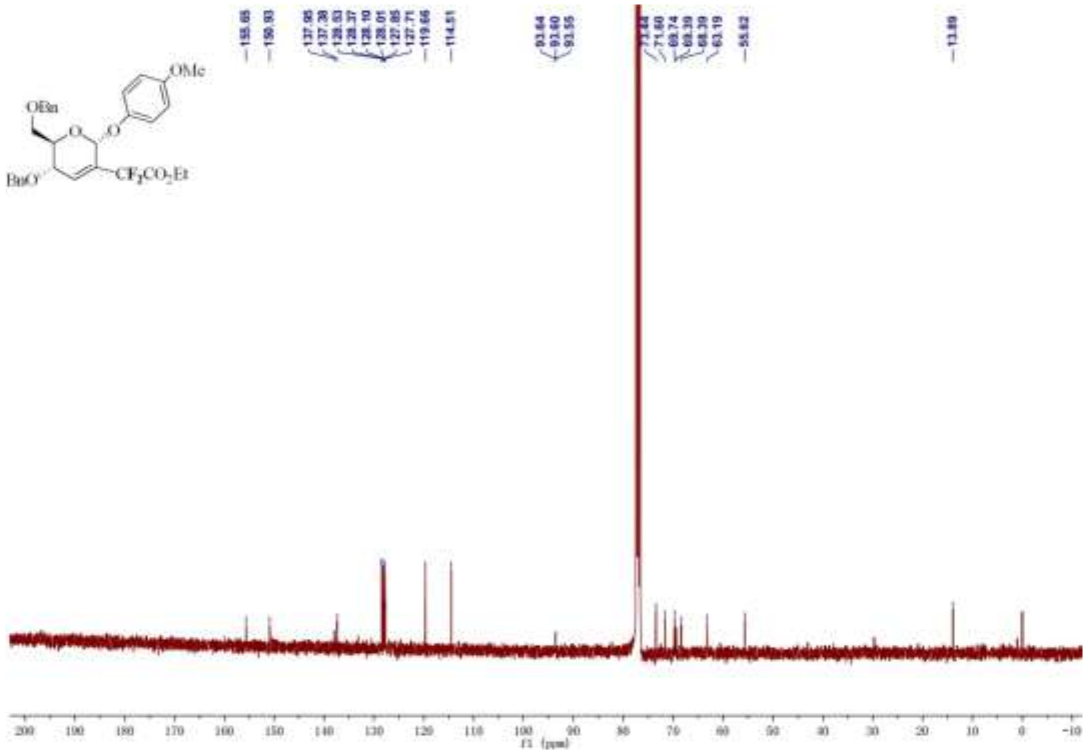

^{13}C NMR spectrum of Compound **3l**

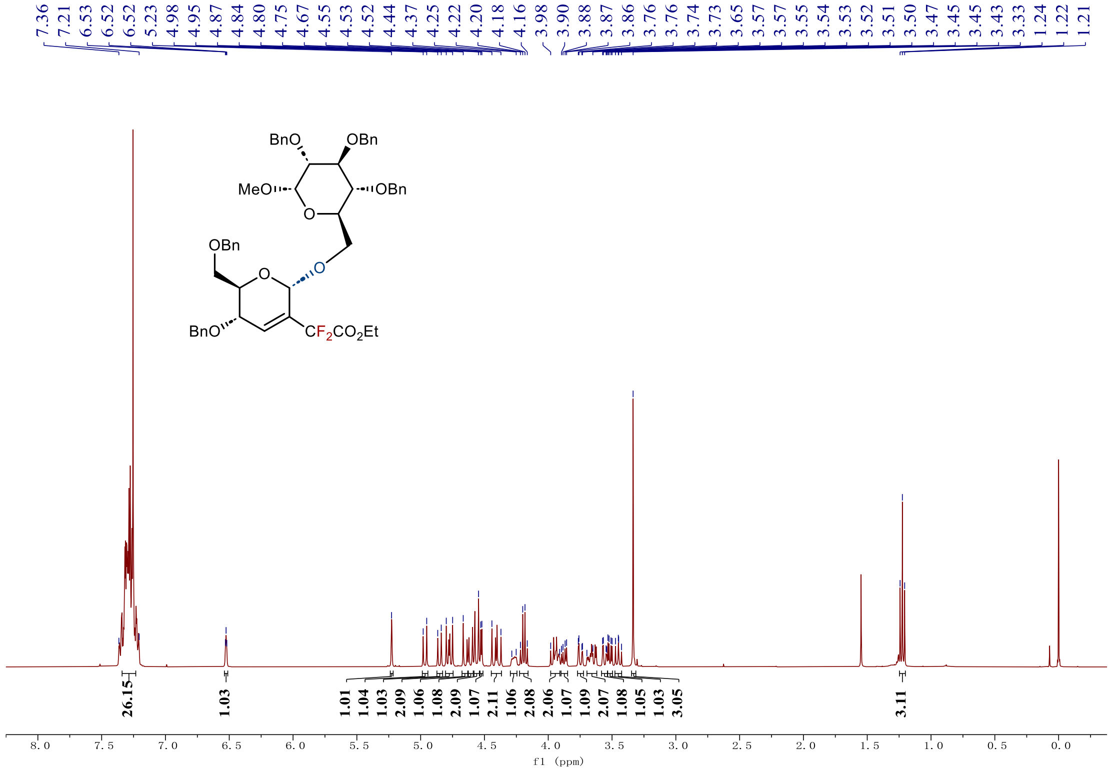

^{1}H NMR spectrum of Compound **4a**

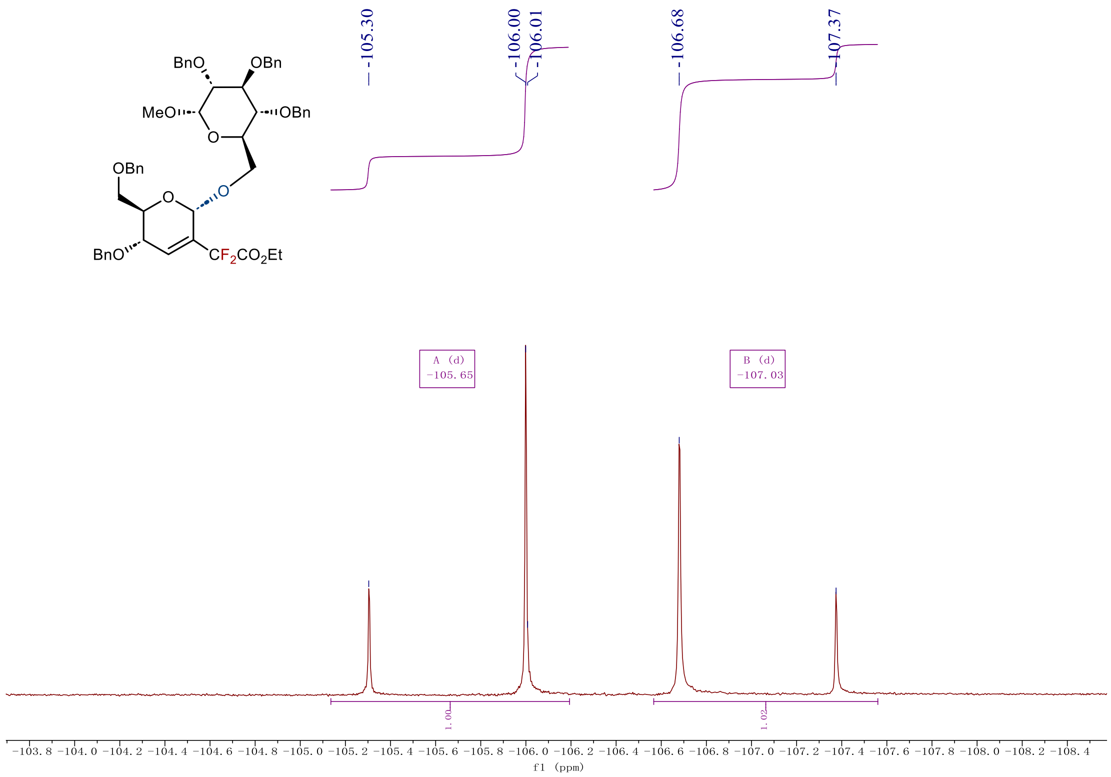

^{19}F NMR spectrum of Compound **4a**

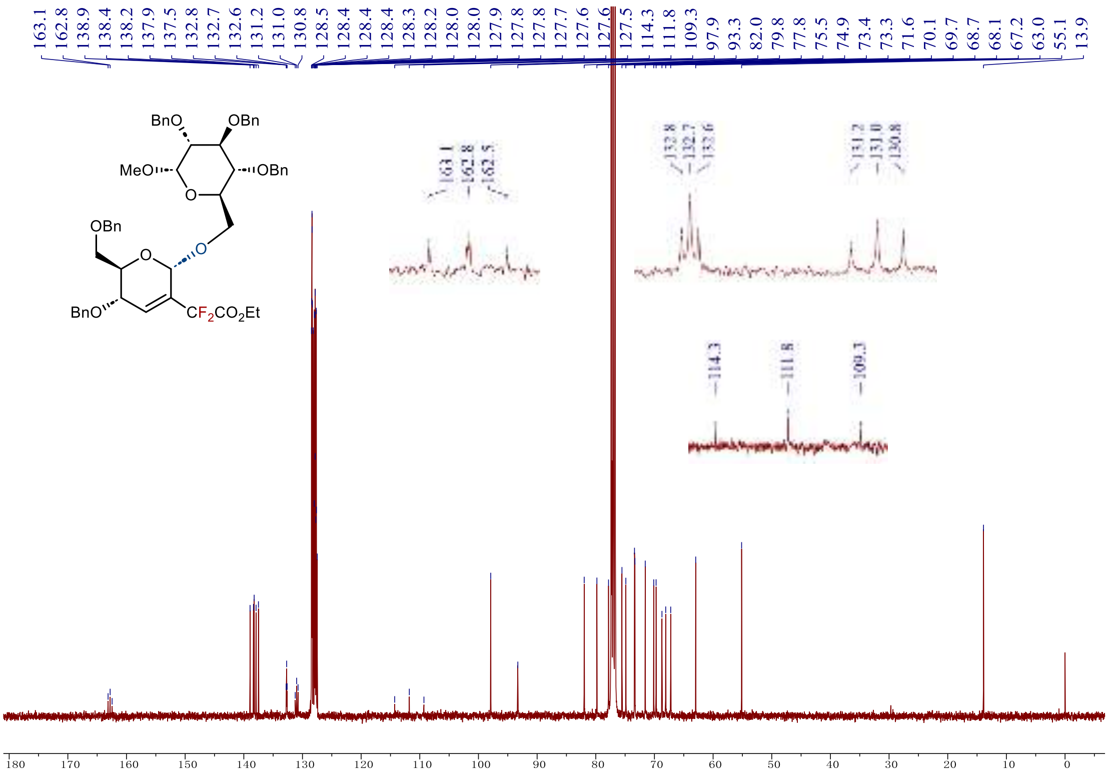

^{13}C NMR spectrum of Compound **4a**

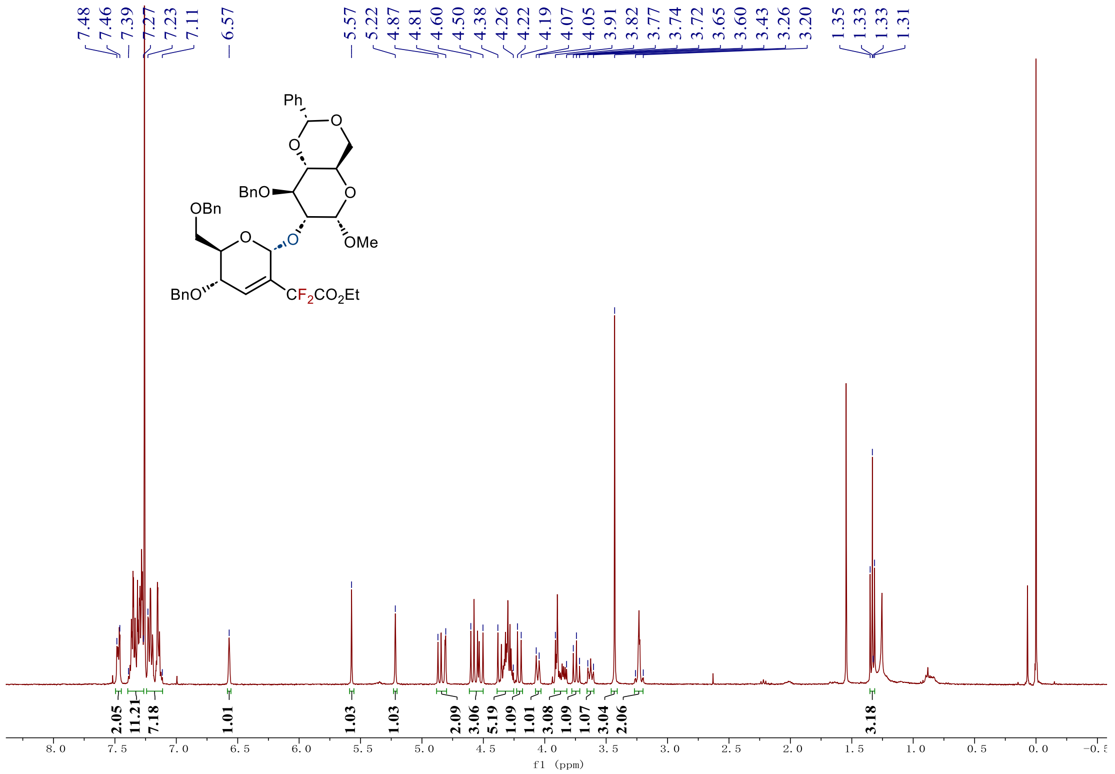

^1H NMR spectrum of Compound **4b**

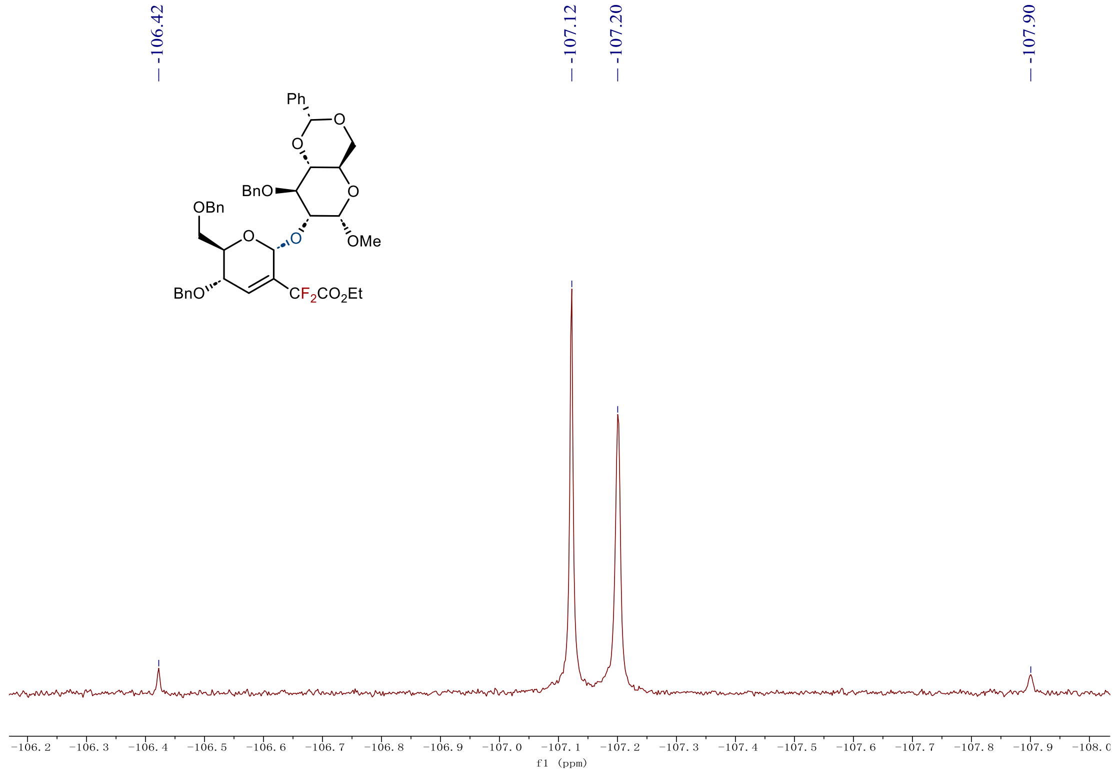

^{19}F NMR spectrum of Compound **4b**

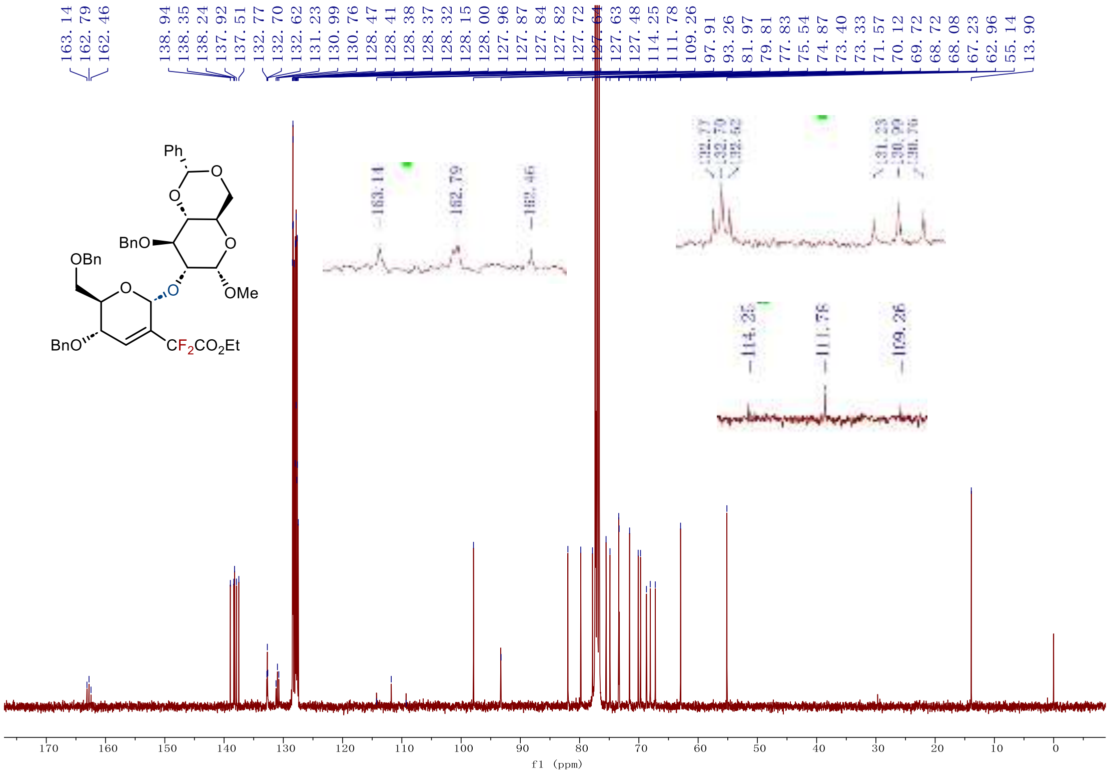

^{13}C NMR spectrum of Compound **4b**

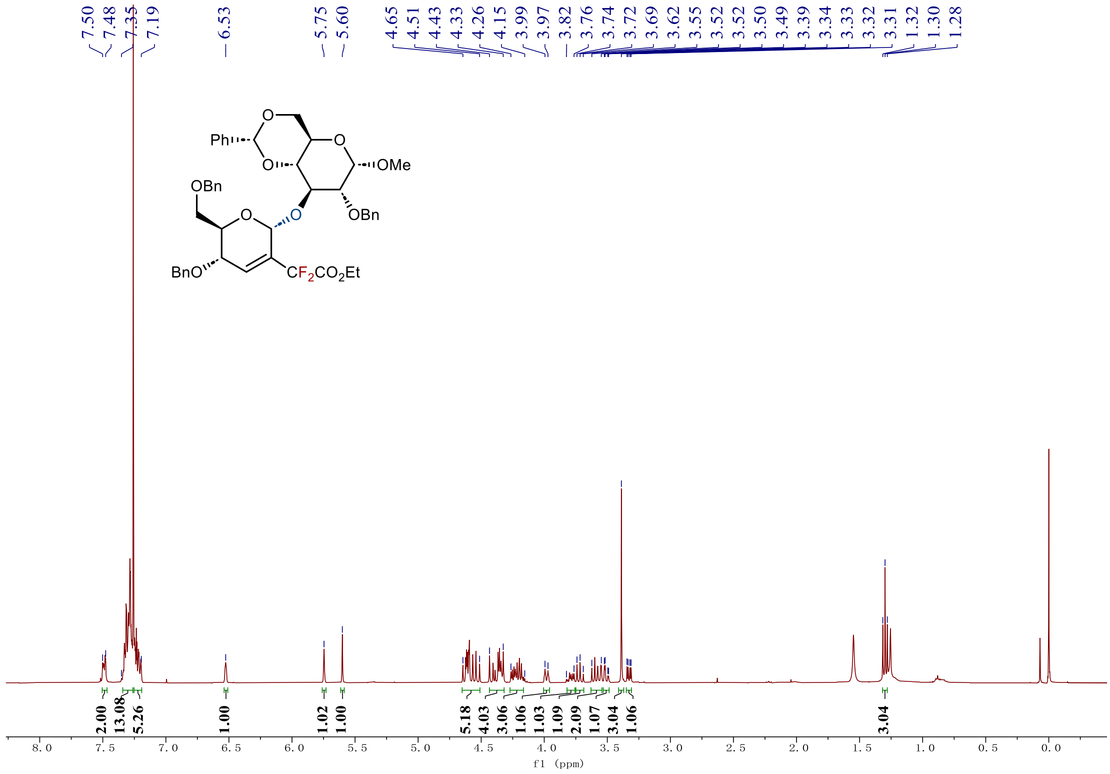

^{1}H NMR spectrum of Compound **4c**

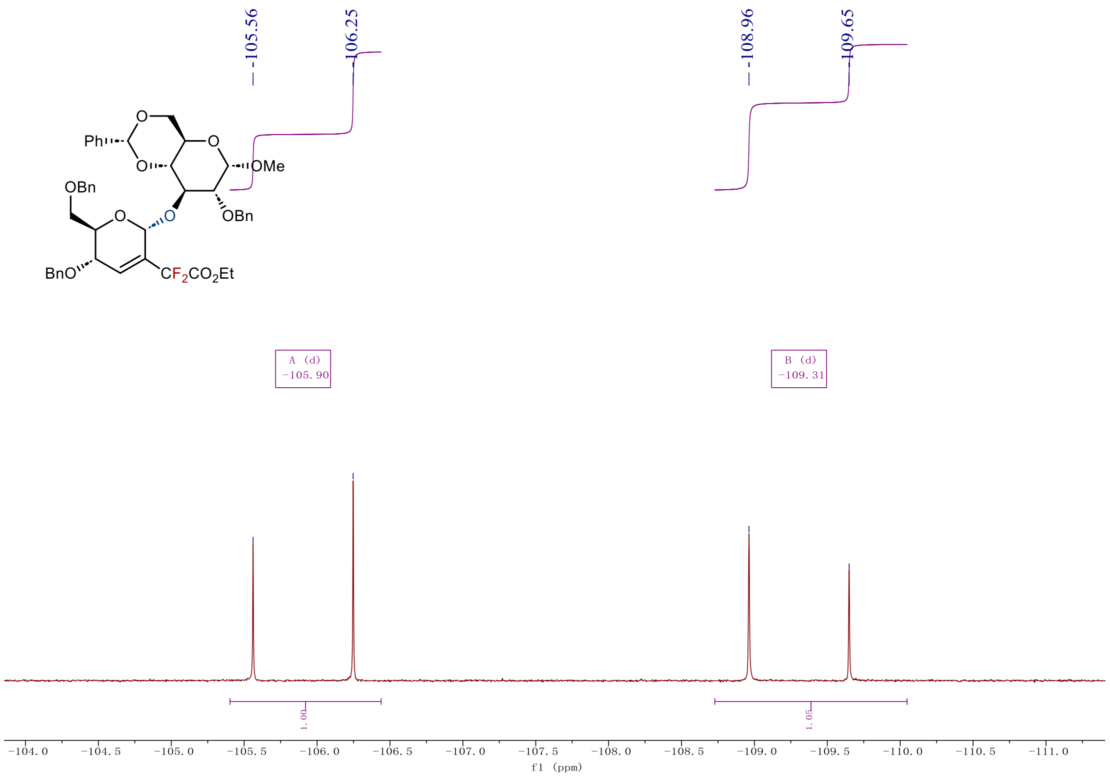

^{19}F NMR spectrum of Compound **4c**

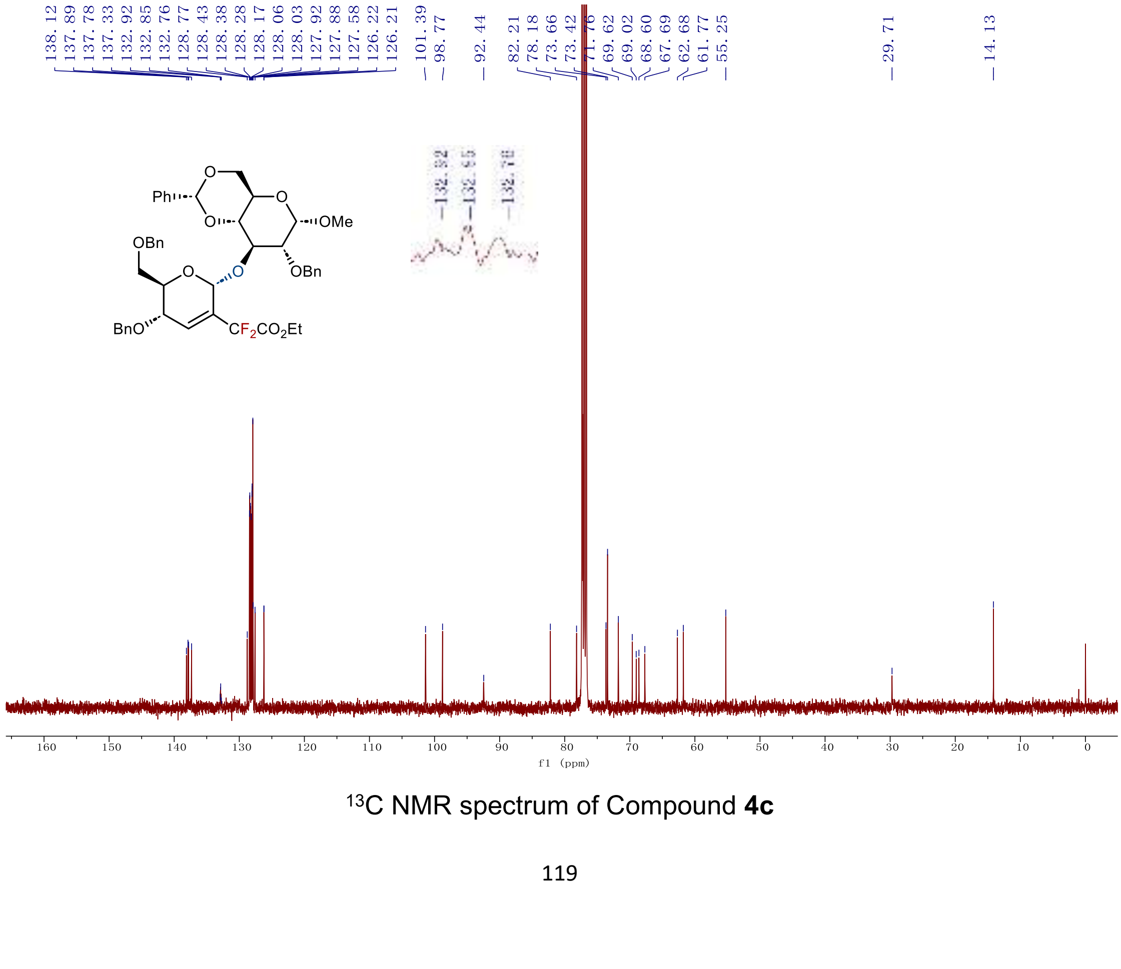

^{13}C NMR spectrum of Compound **4c**

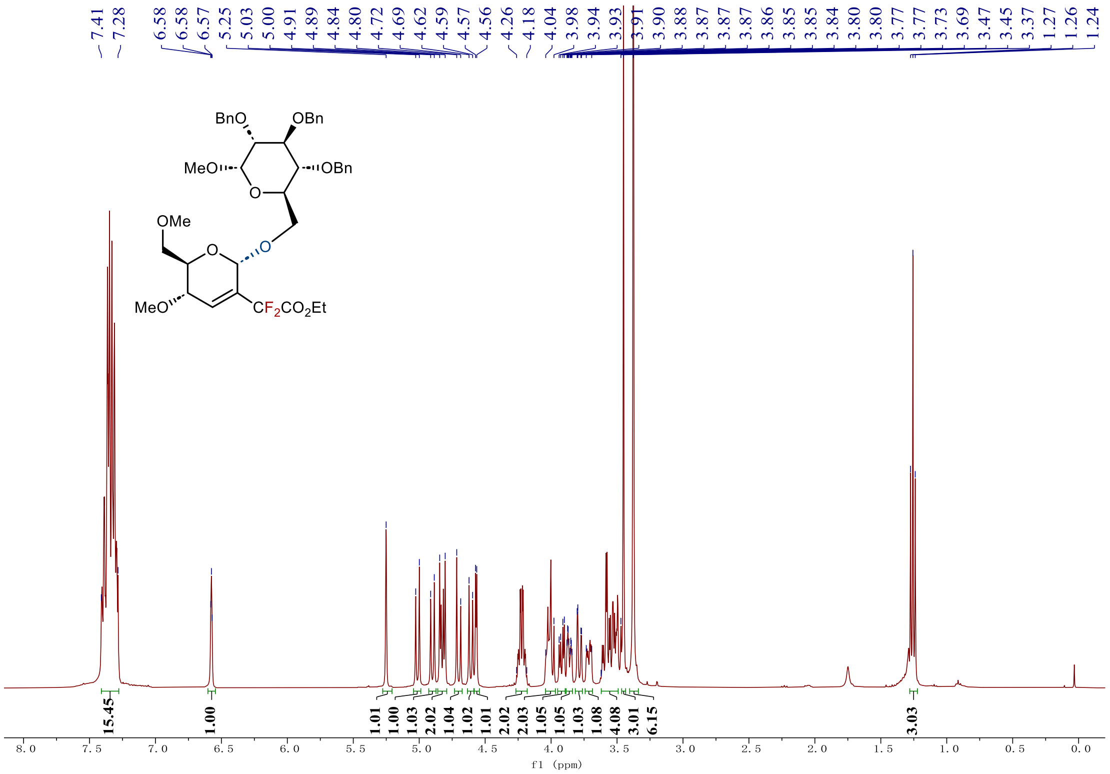

^{1}H NMR spectrum of Compound **4d**

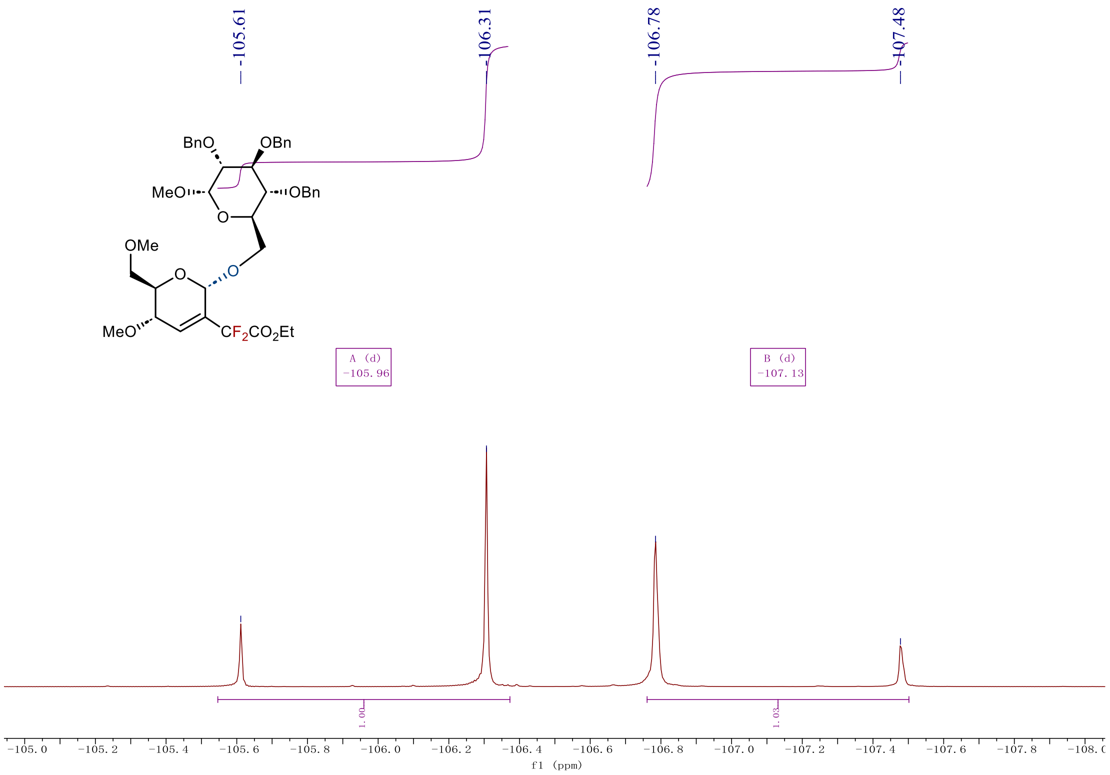

19F NMR spectrum of Compound **4d**

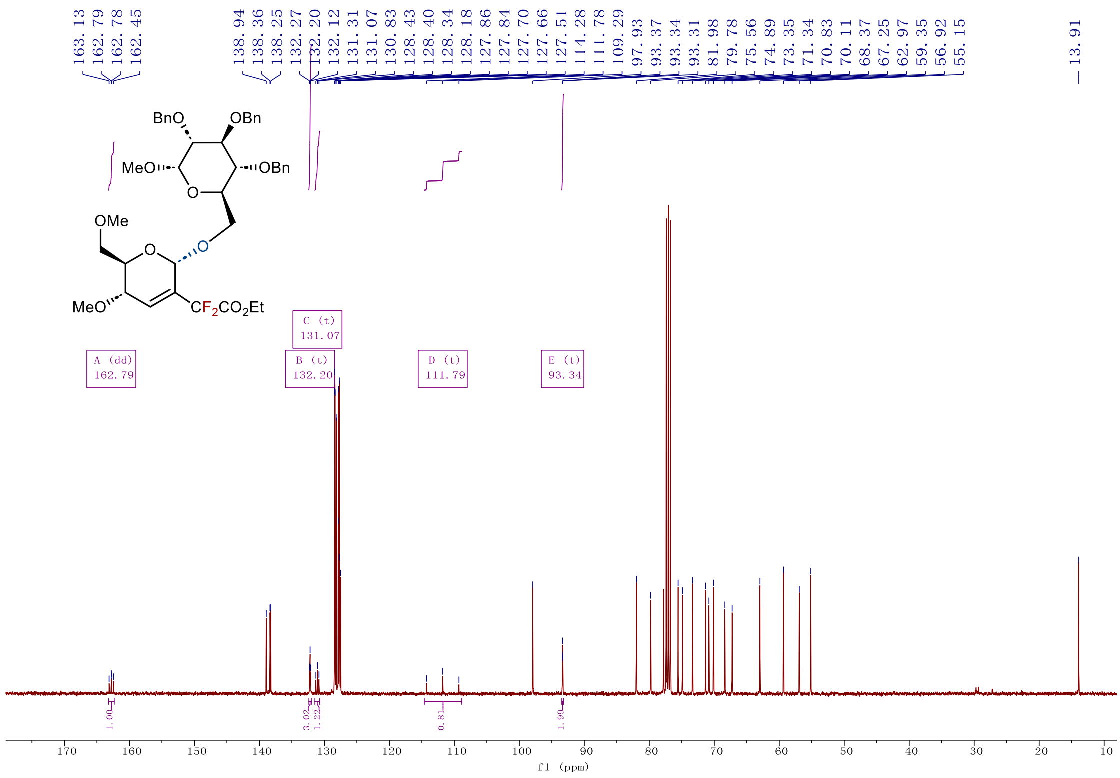

13C NMR spectrum of Compound **4d**

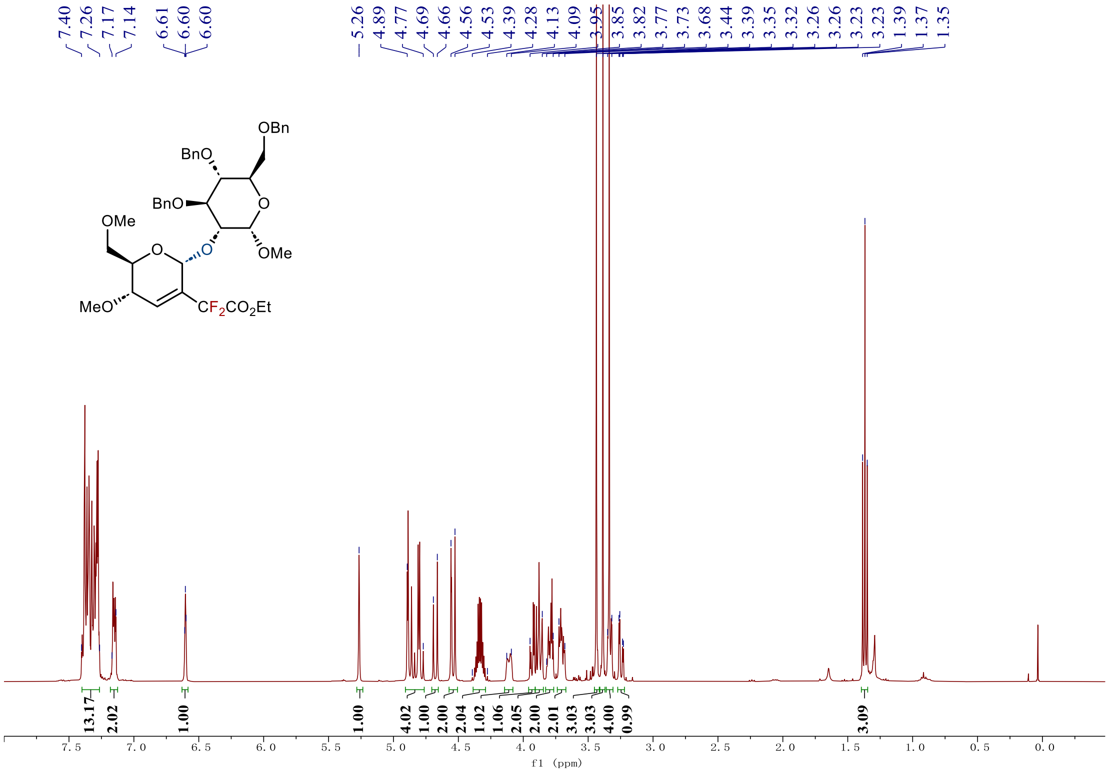

1H NMR spectrum of Compound **4e**

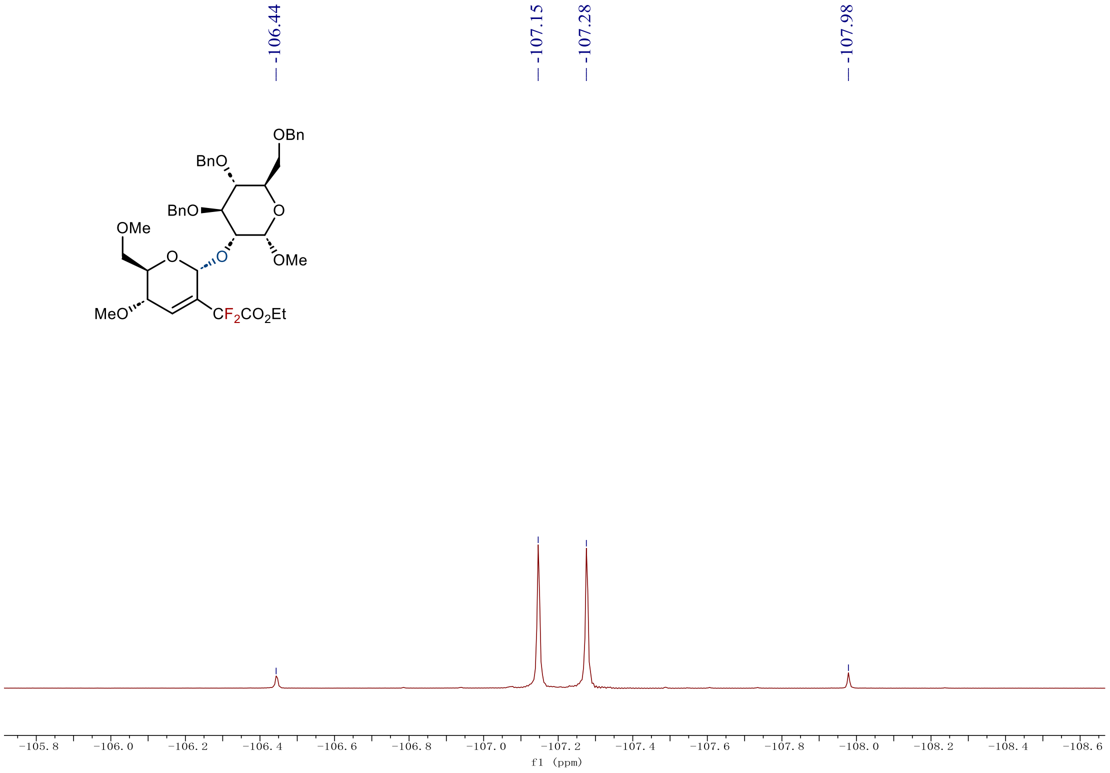

^{19}F NMR spectrum of Compound **4e**

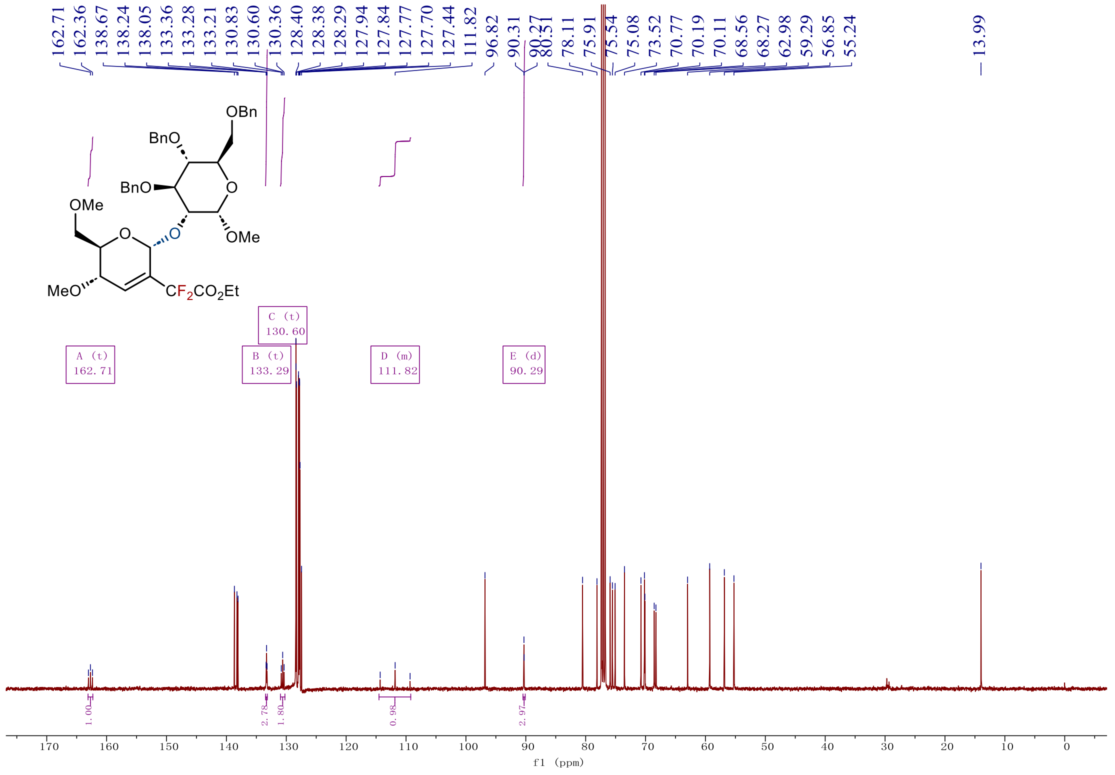

^{13}C NMR spectrum of Compound **4e**

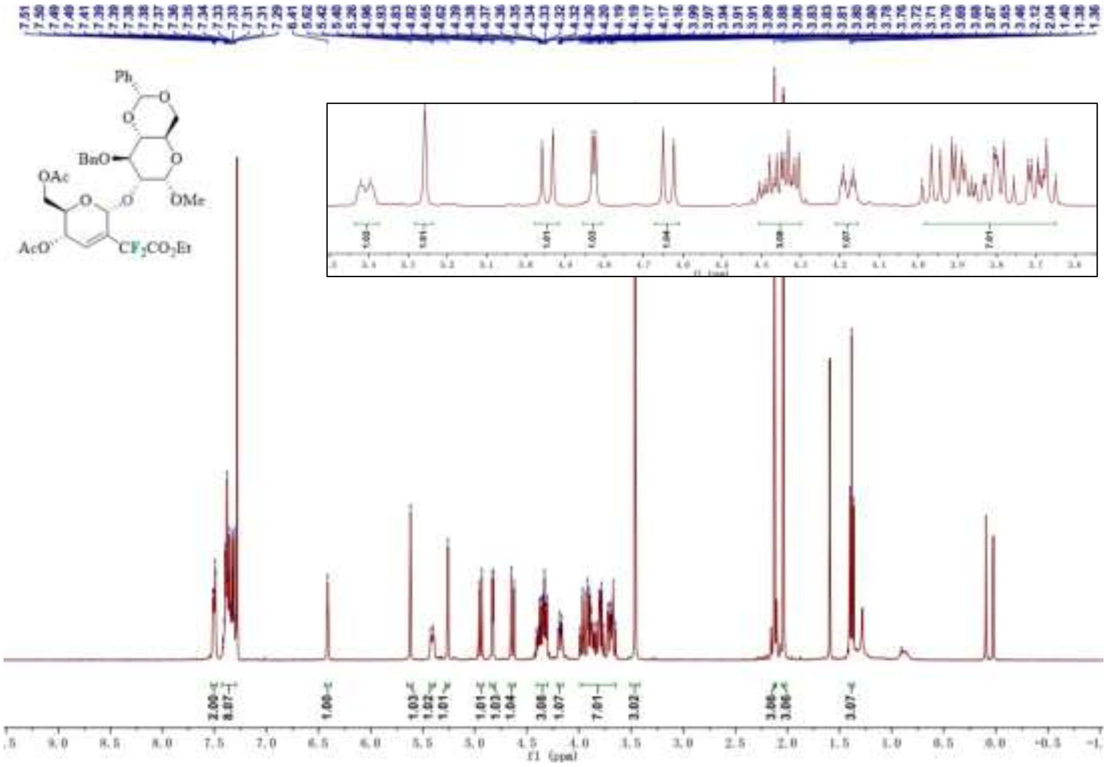

^{1}H NMR spectrum of Compound **4f**

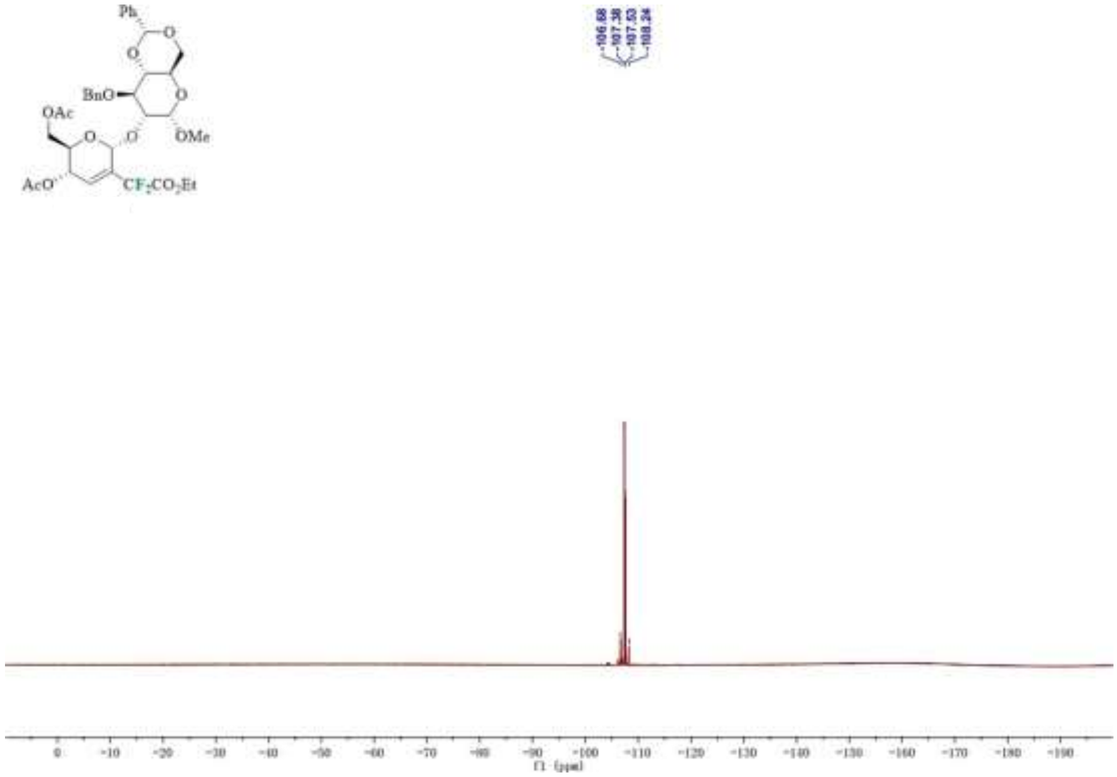

^{19}F NMR spectrum of Compound **4f**

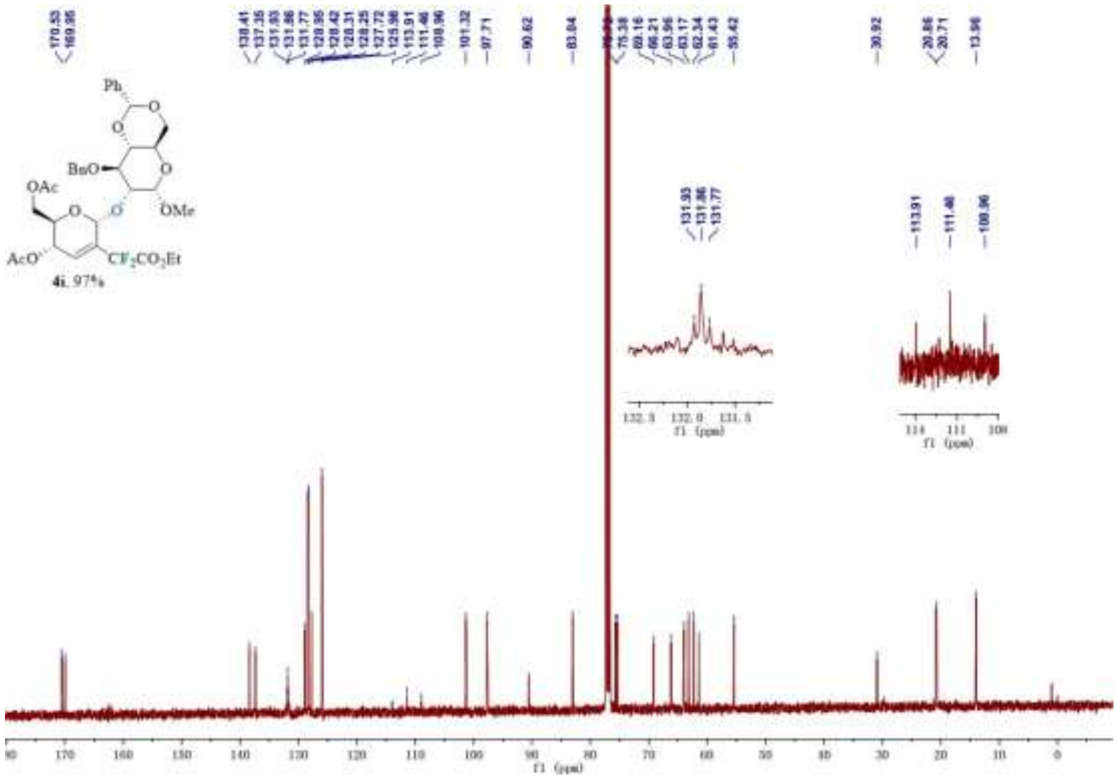

^{13}C NMR spectrum of Compound **4f**

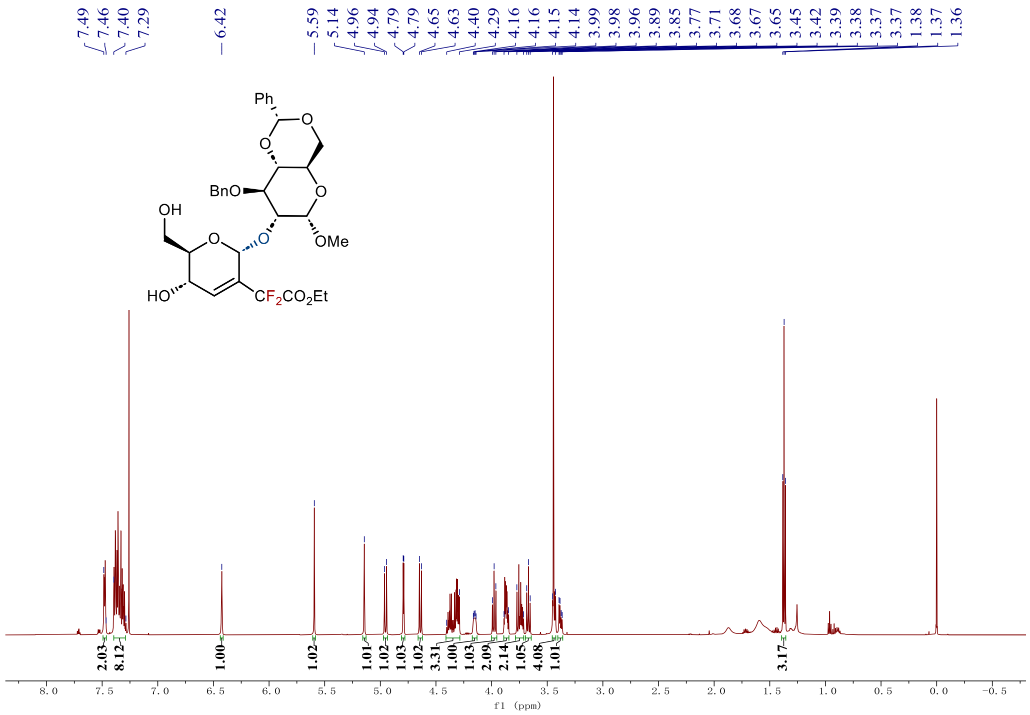

^{1}H NMR spectrum of Compound **4f-OH**

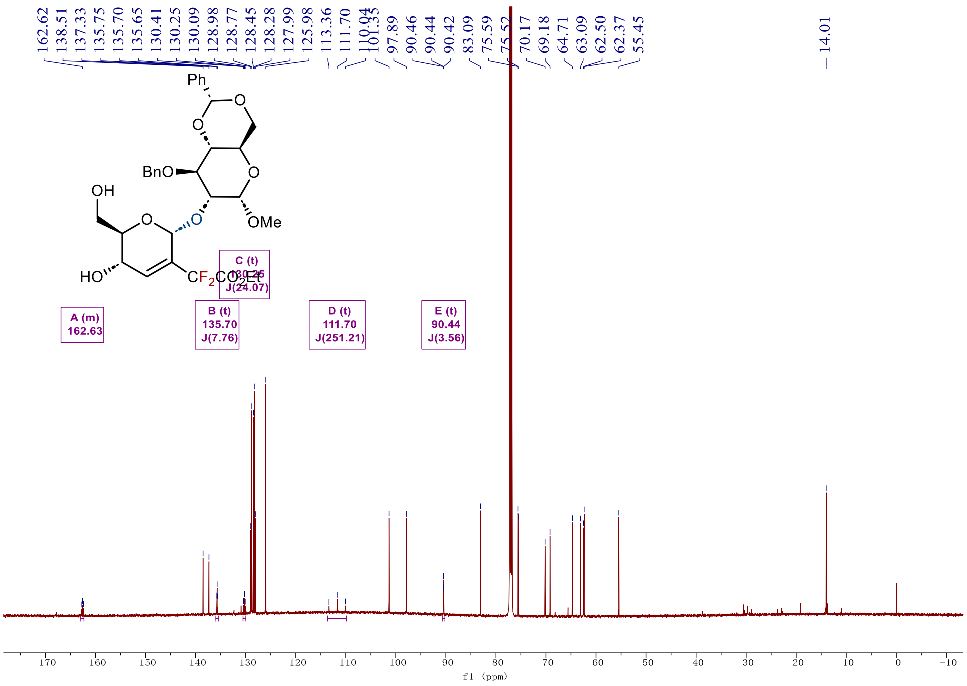

^{13}C NMR spectrum of Compound **4f-OH**

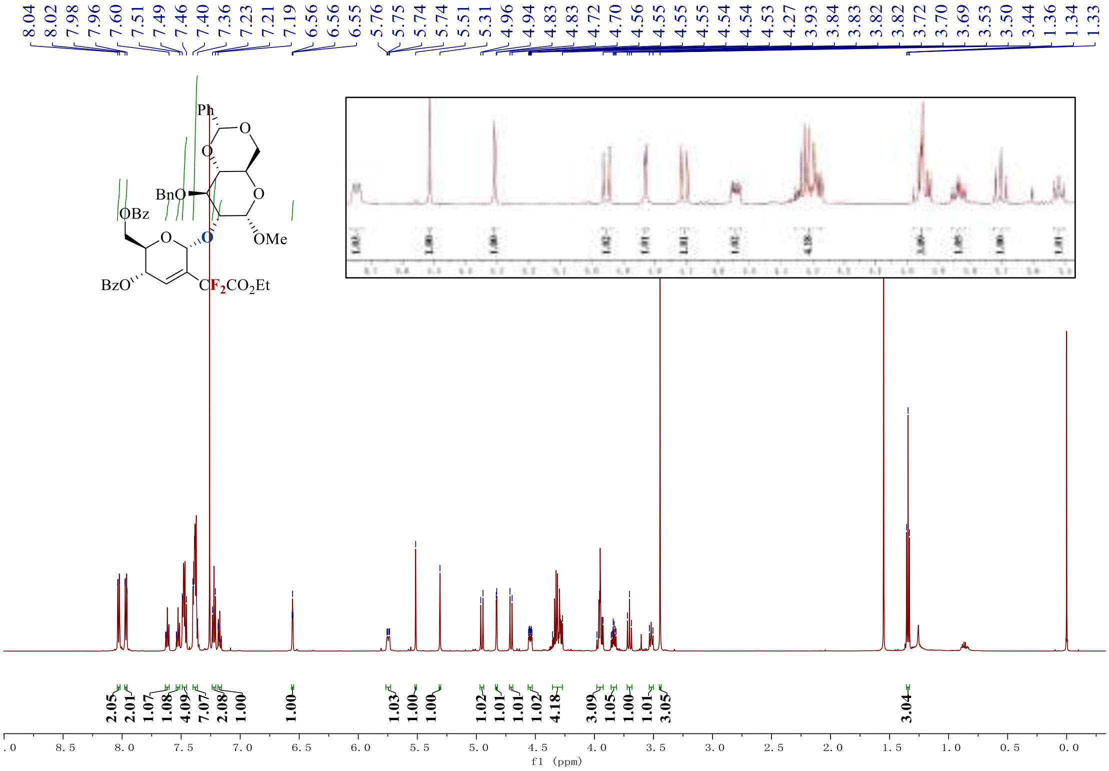

^{1}H NMR spectrum of Compound **4g**

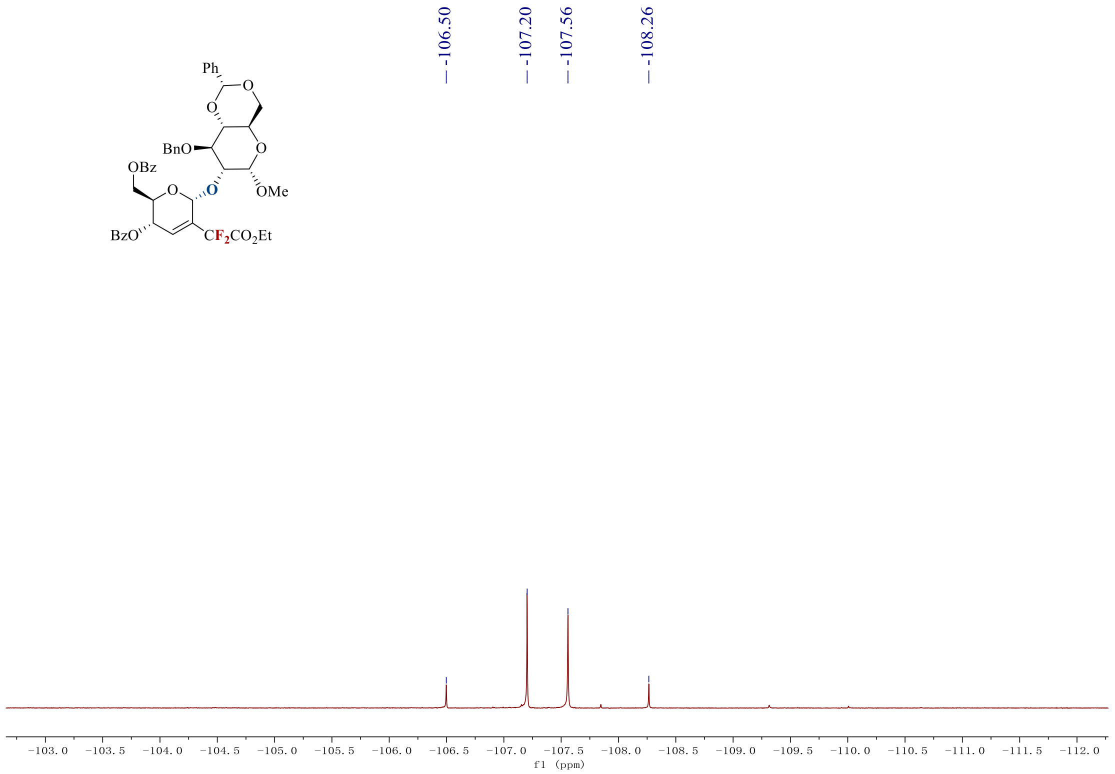

^{19}F NMR spectrum of Compound **4g**

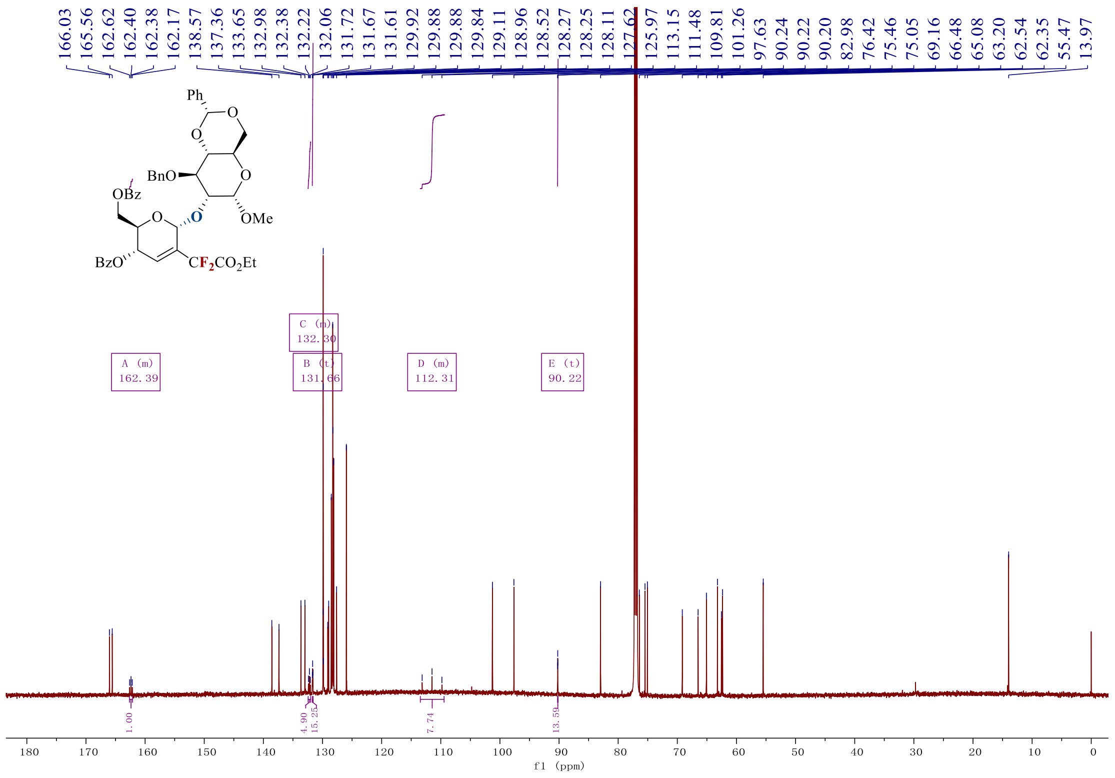

^{13}C NMR spectrum of Compound **4g**

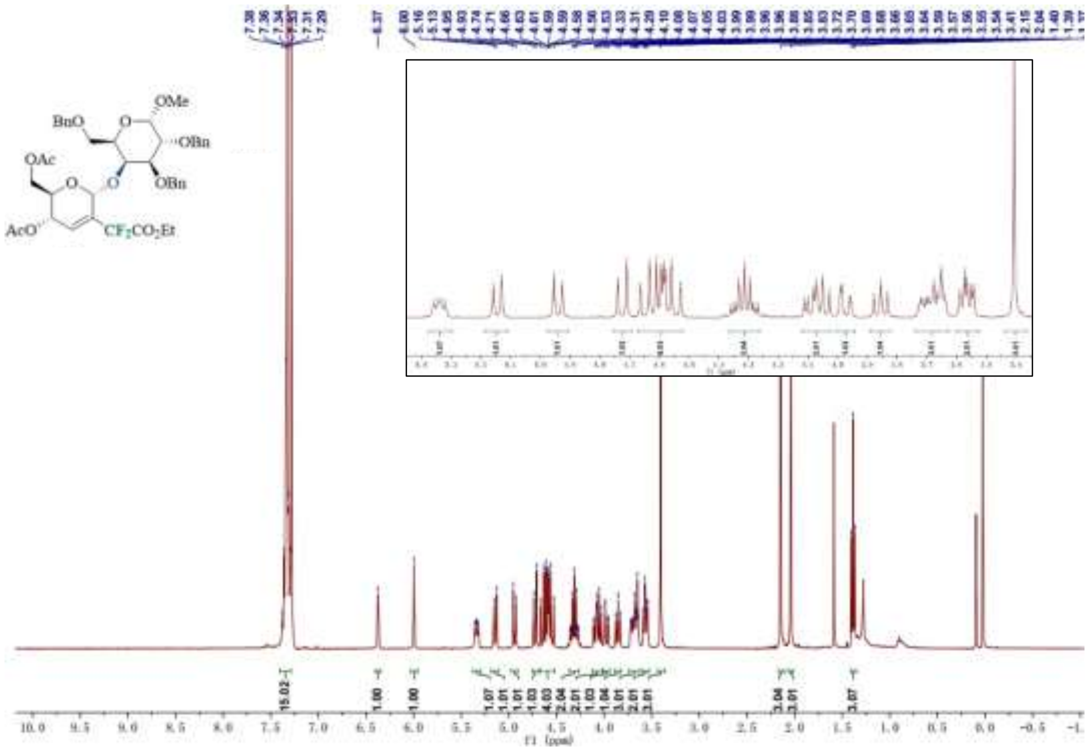

^1H NMR spectrum of Compound **4h**

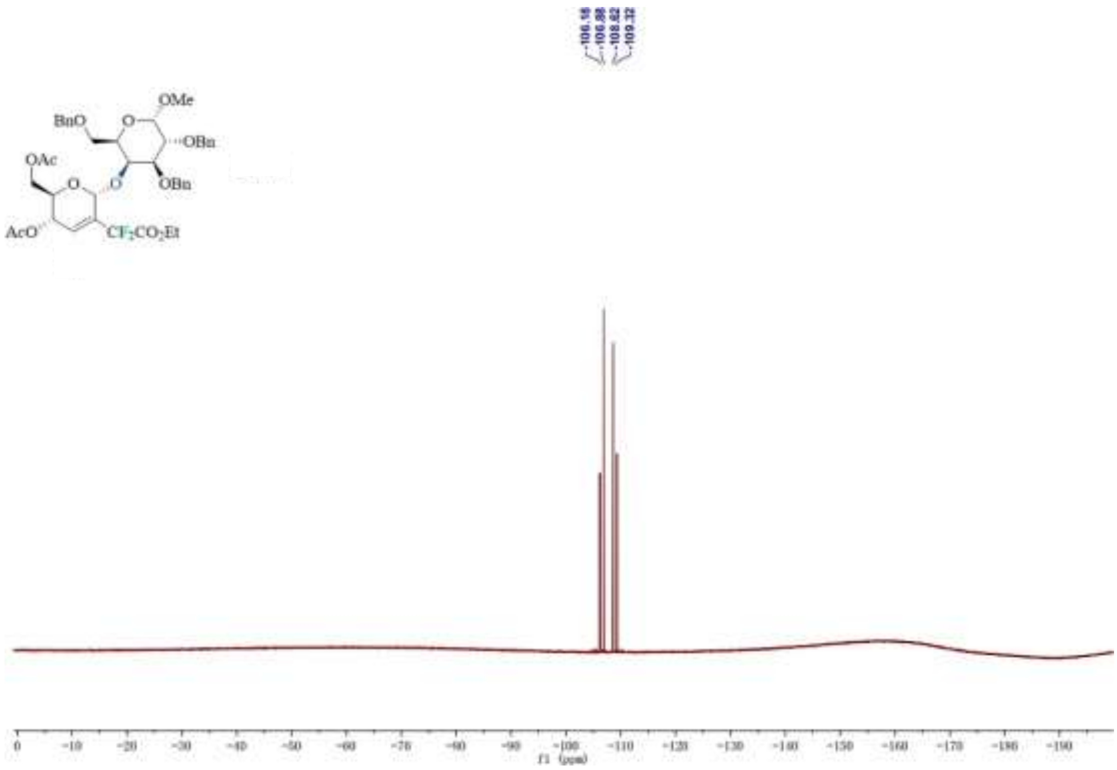

^{19}F NMR spectrum of Compound **4h**

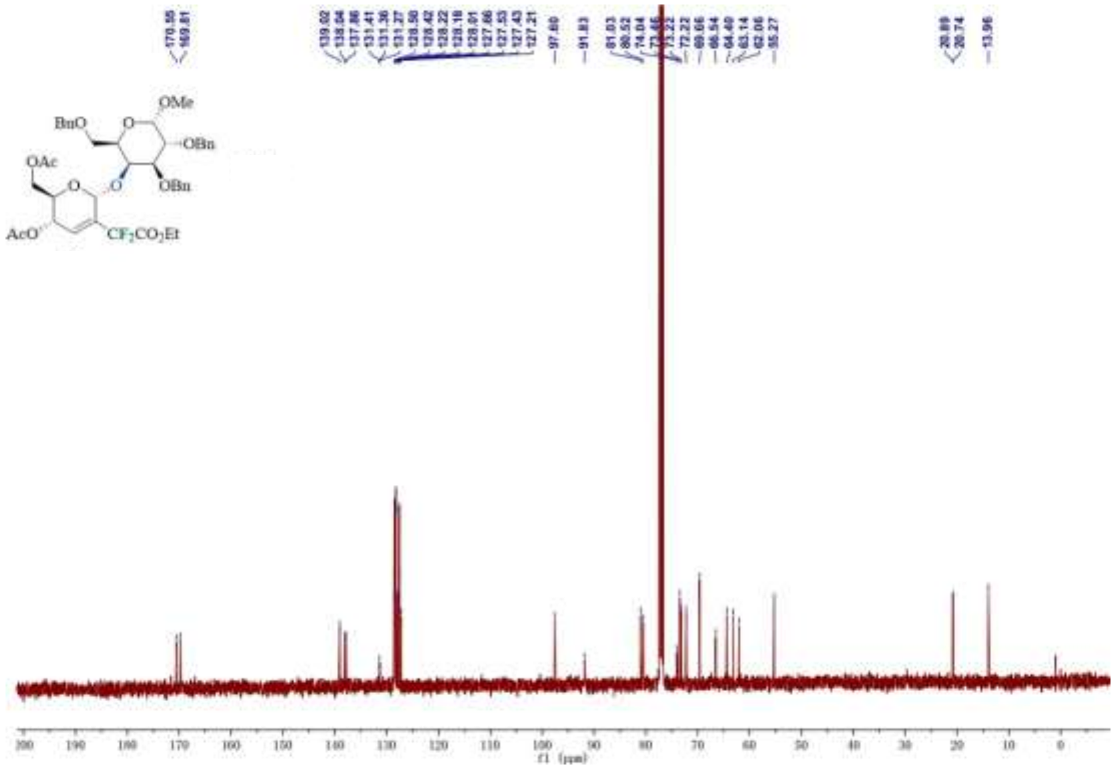

^{13}C NMR spectrum of Compound **4h**

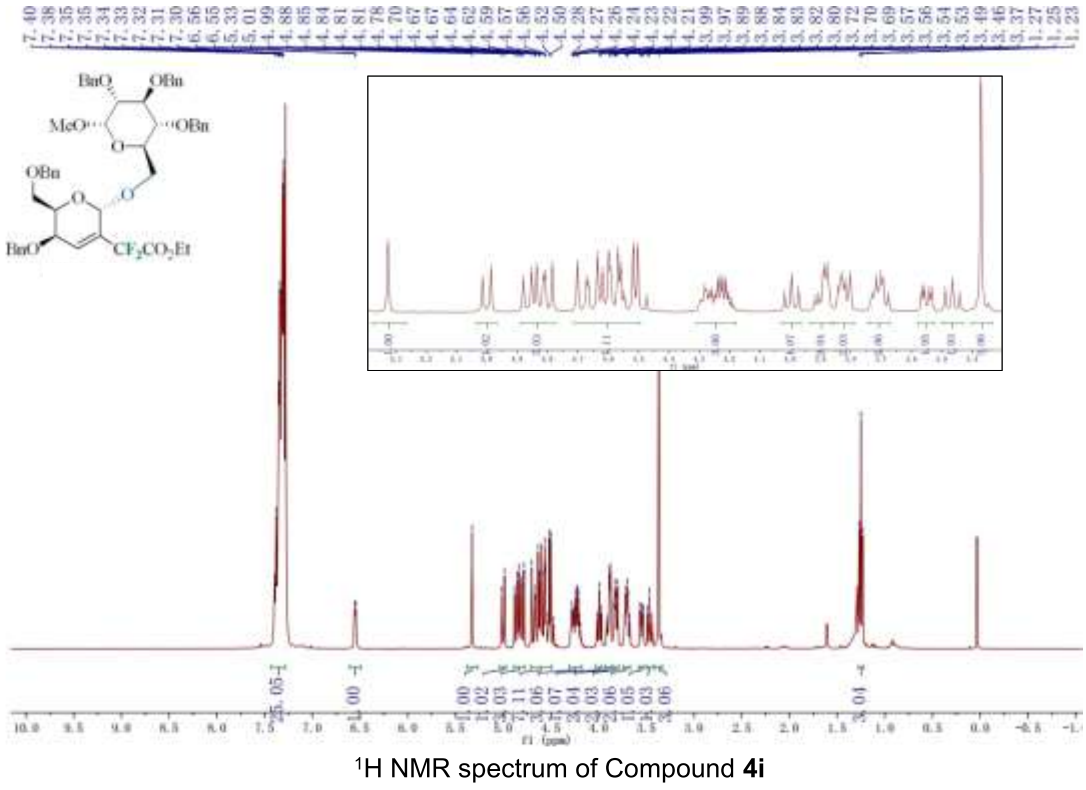

^{1}H NMR spectrum of Compound **4i**

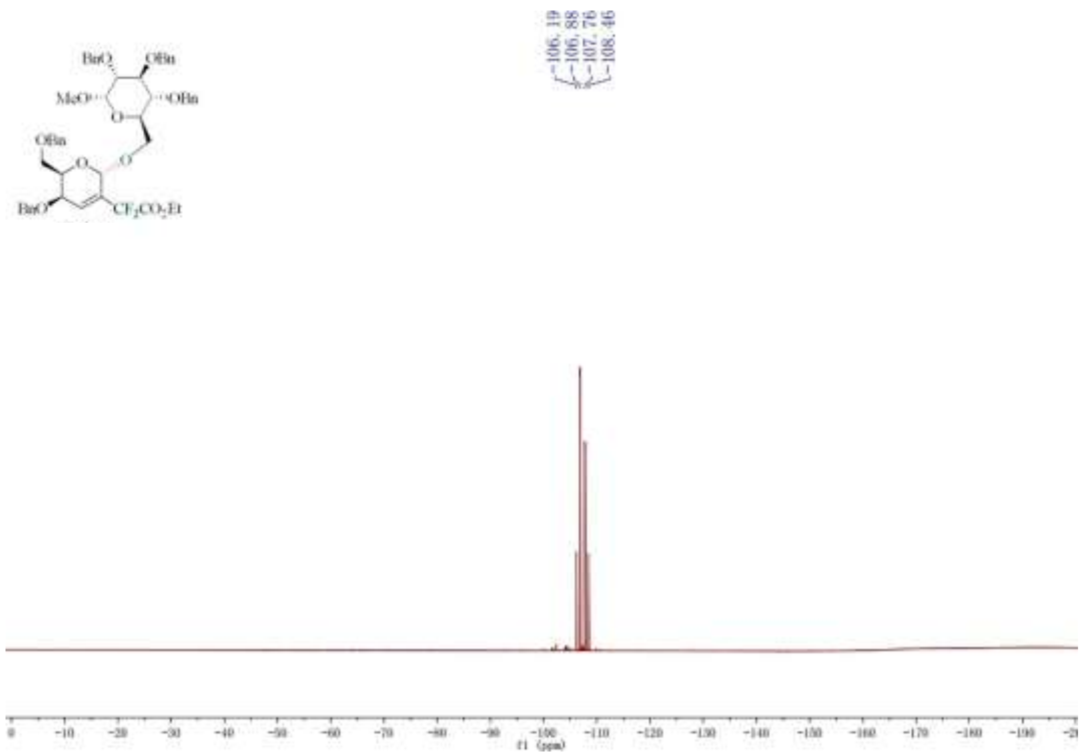

^{19}F NMR spectrum of Compound **4i**

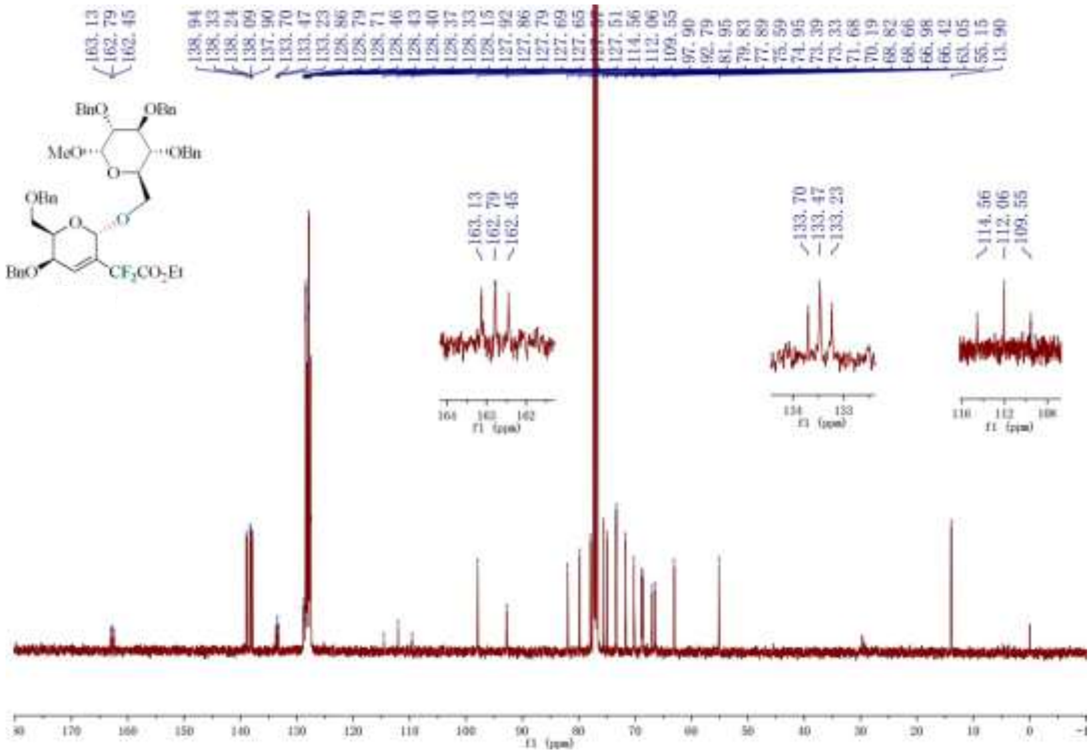

^{13}C NMR spectrum of Compound **4i**

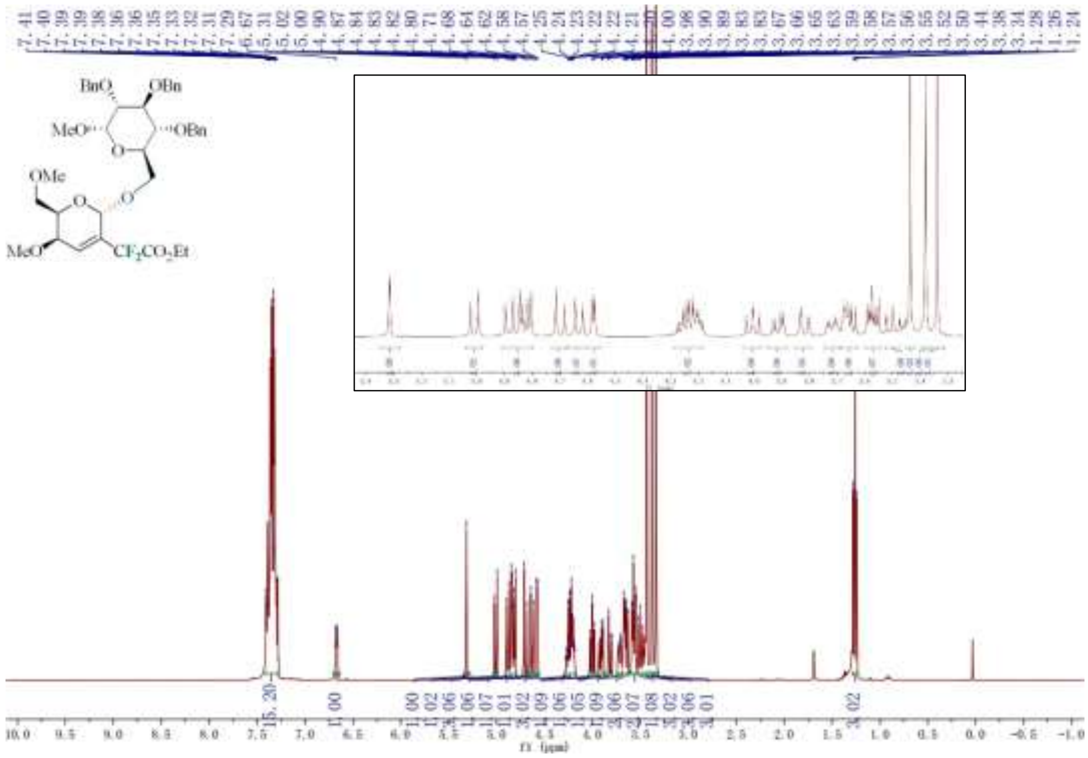

^{1}H NMR spectrum of Compound **4j**

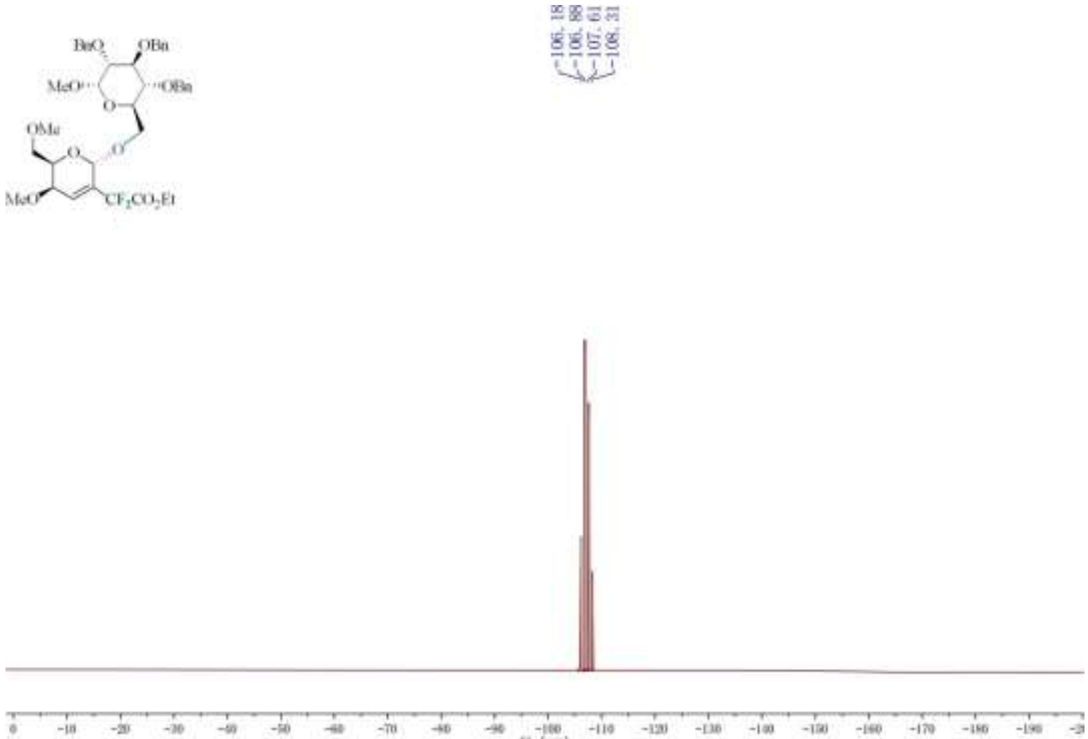

^{19}F NMR spectrum of Compound **4j**

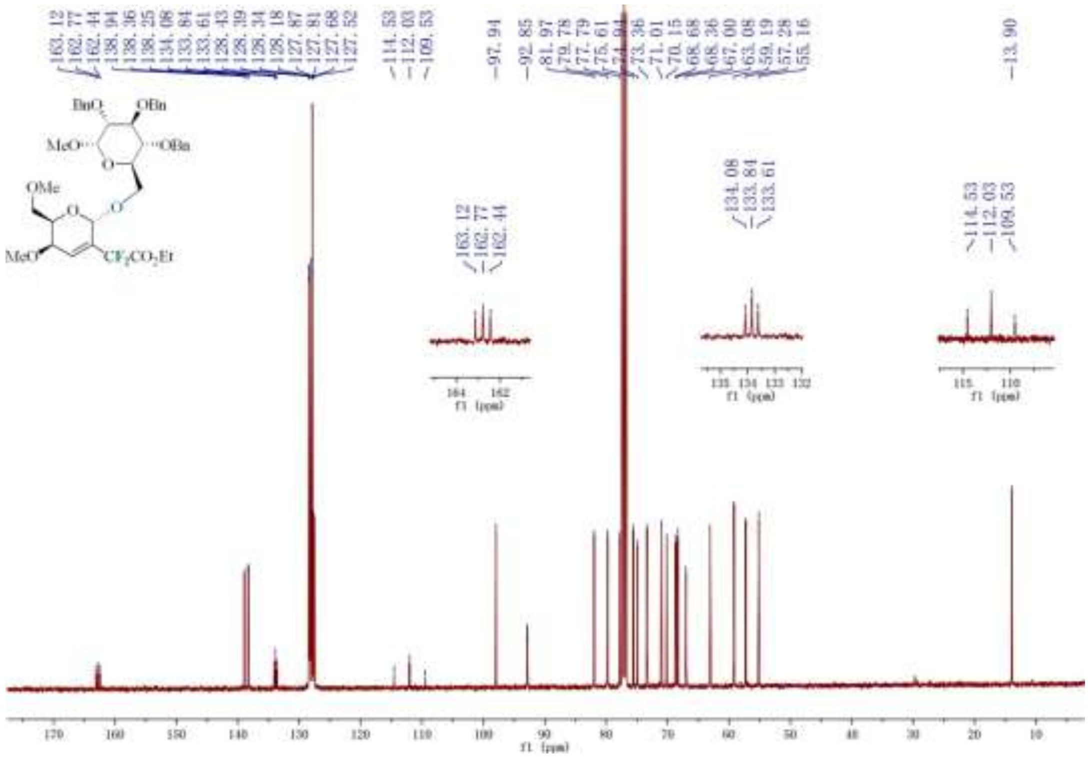

^{13}C NMR spectrum of Compound **4j**

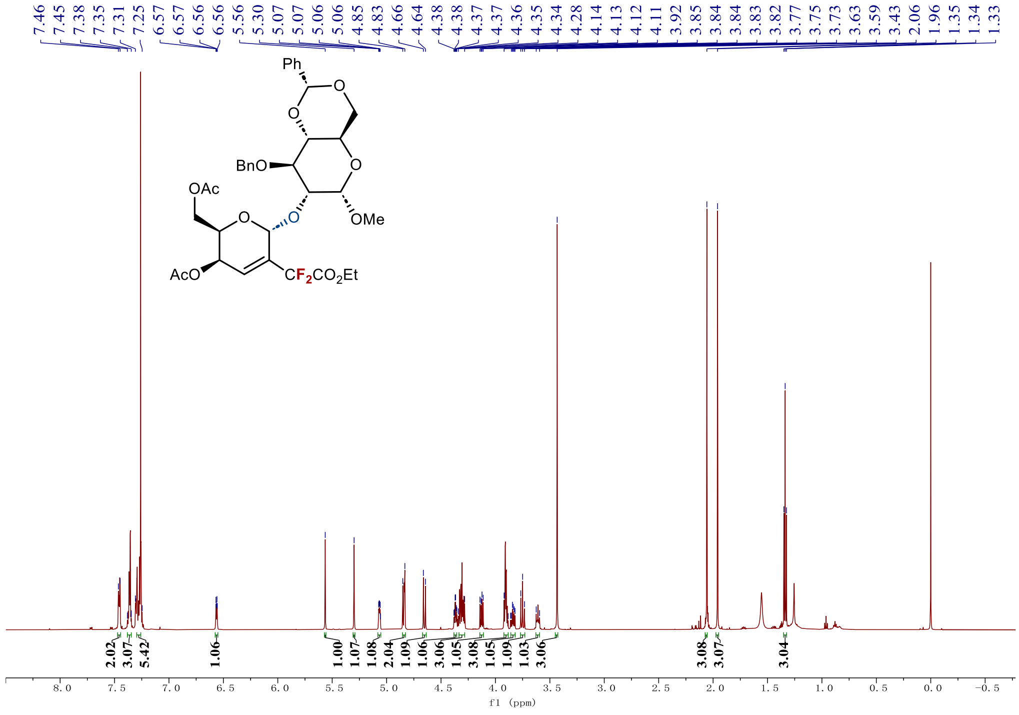

^{1}H NMR spectrum of Compound **4k**

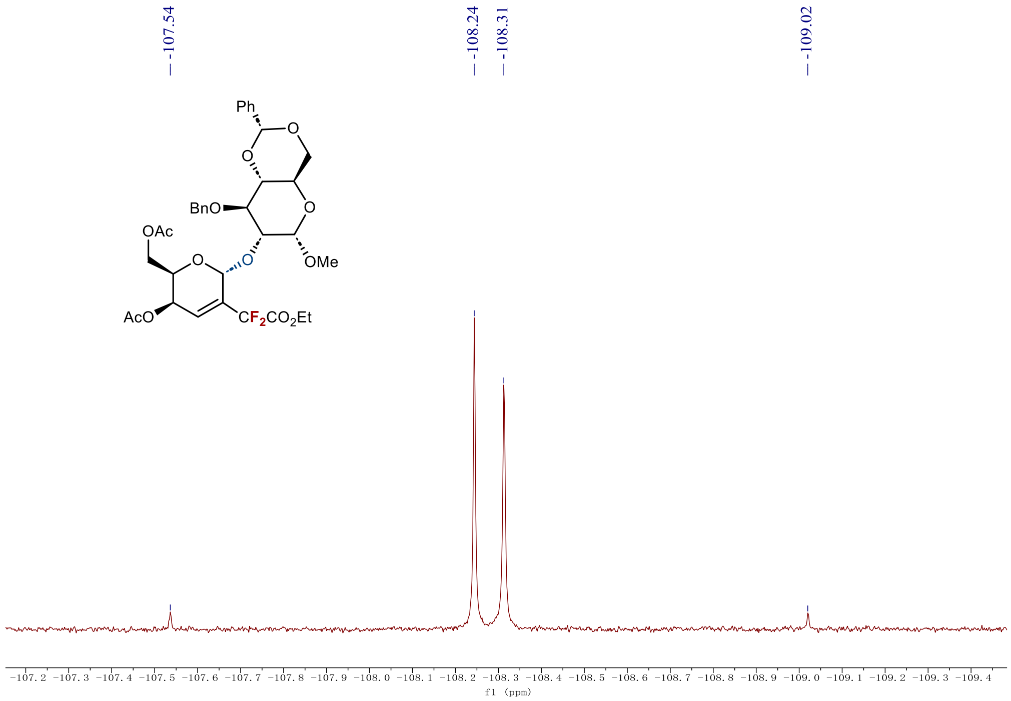

^{19}F NMR spectrum of Compound **4k**

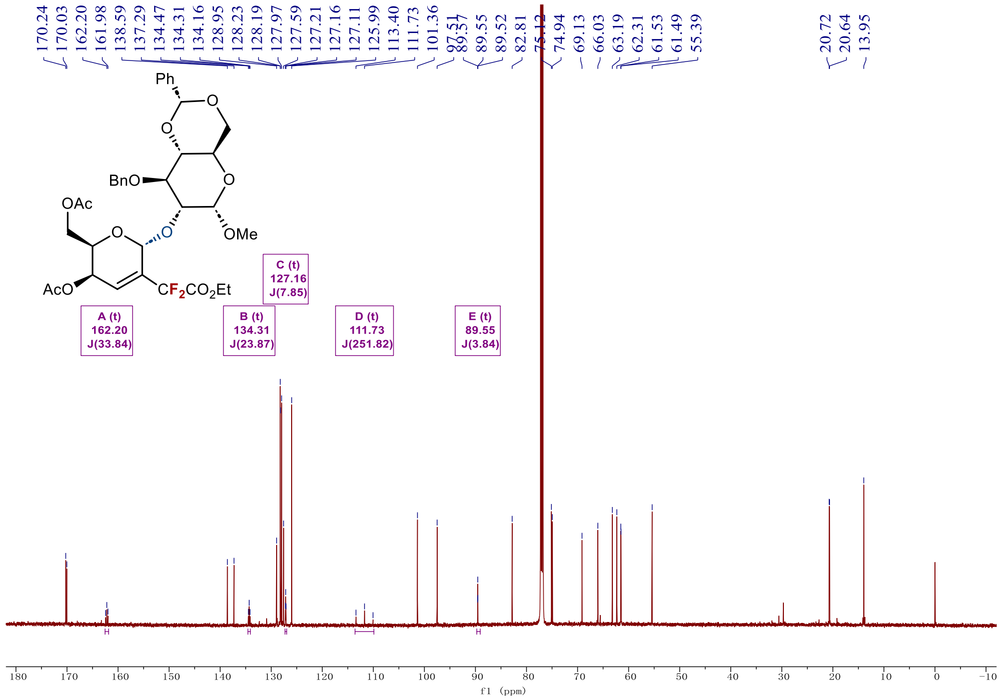

^{13}C NMR spectrum of Compound **4k**

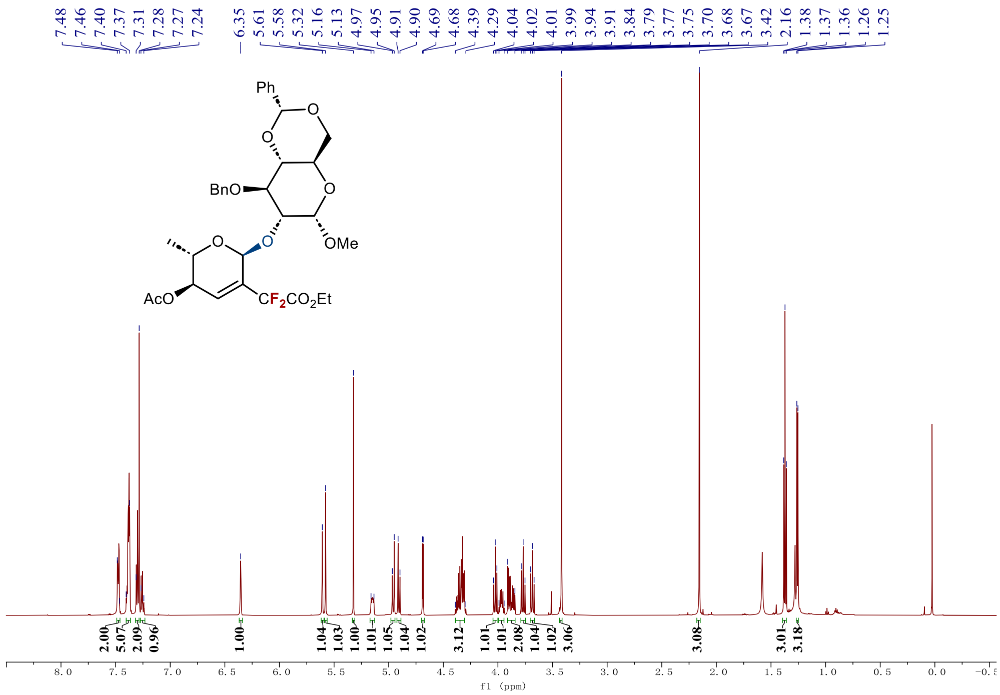

^{1}H NMR spectrum of Compound **4l**

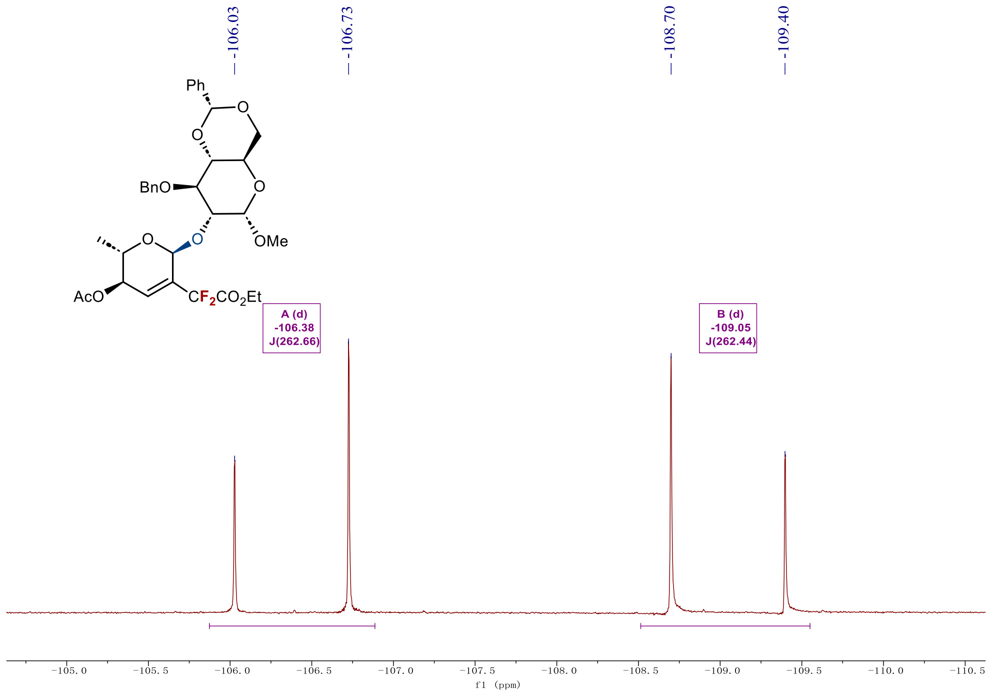

^{19}F NMR spectrum of Compound **4l**

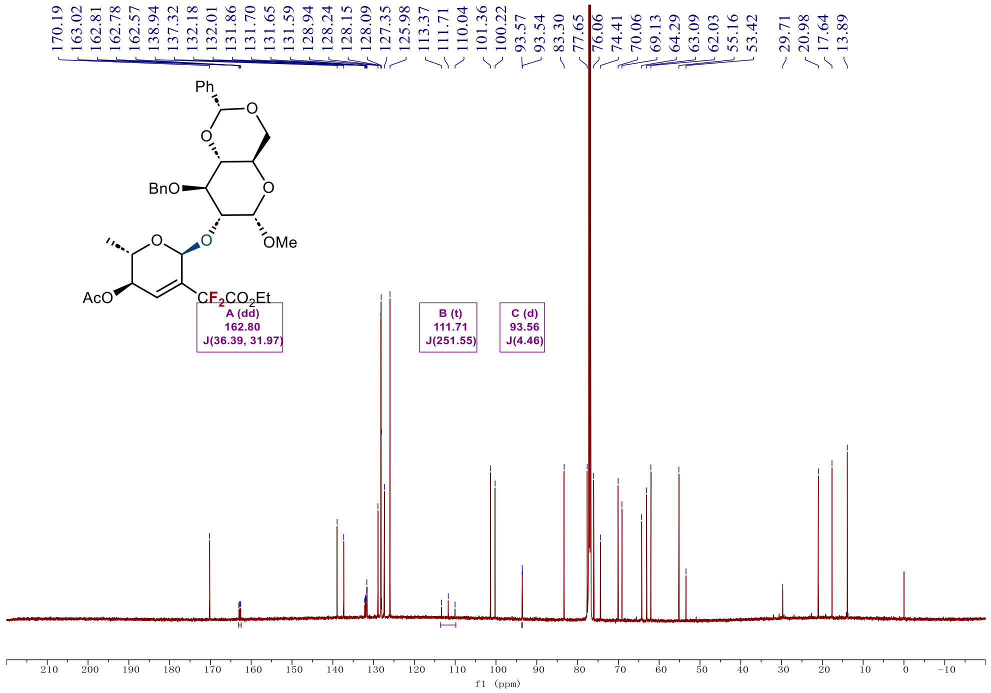

^{13}C NMR spectrum of Compound **4l**

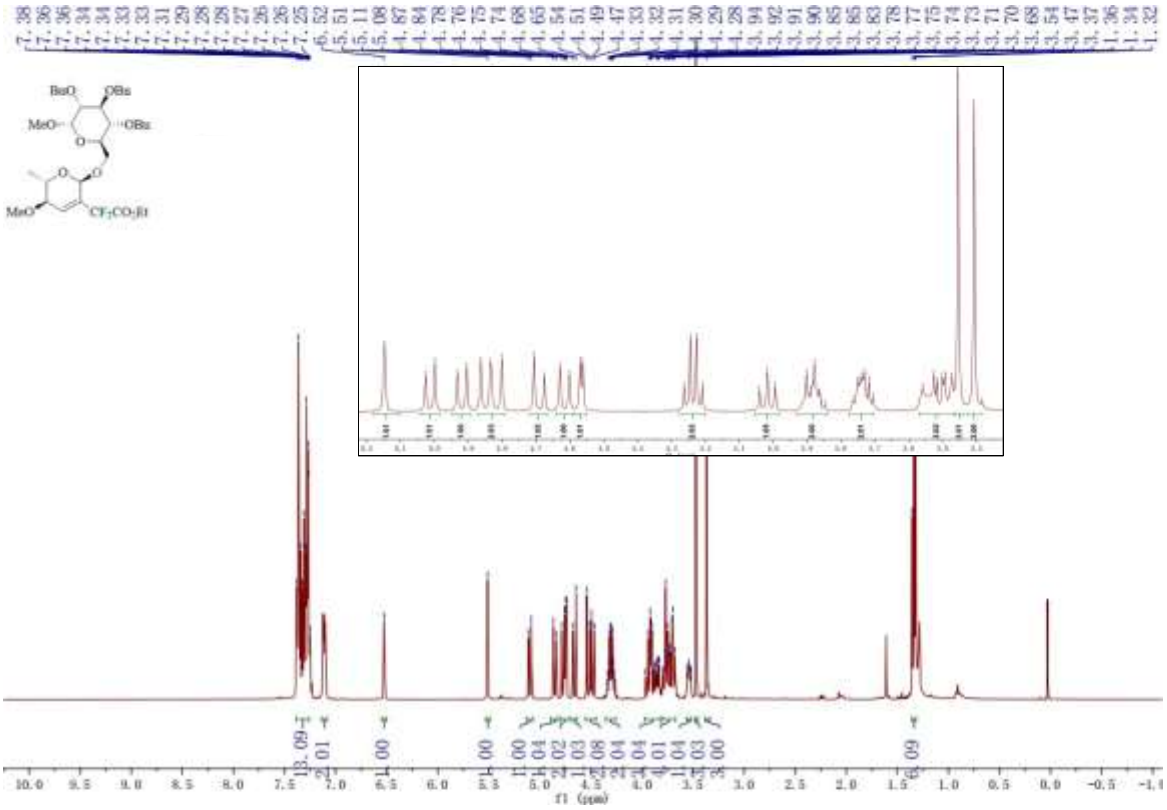

^{1}H NMR spectrum of Compound **4m**

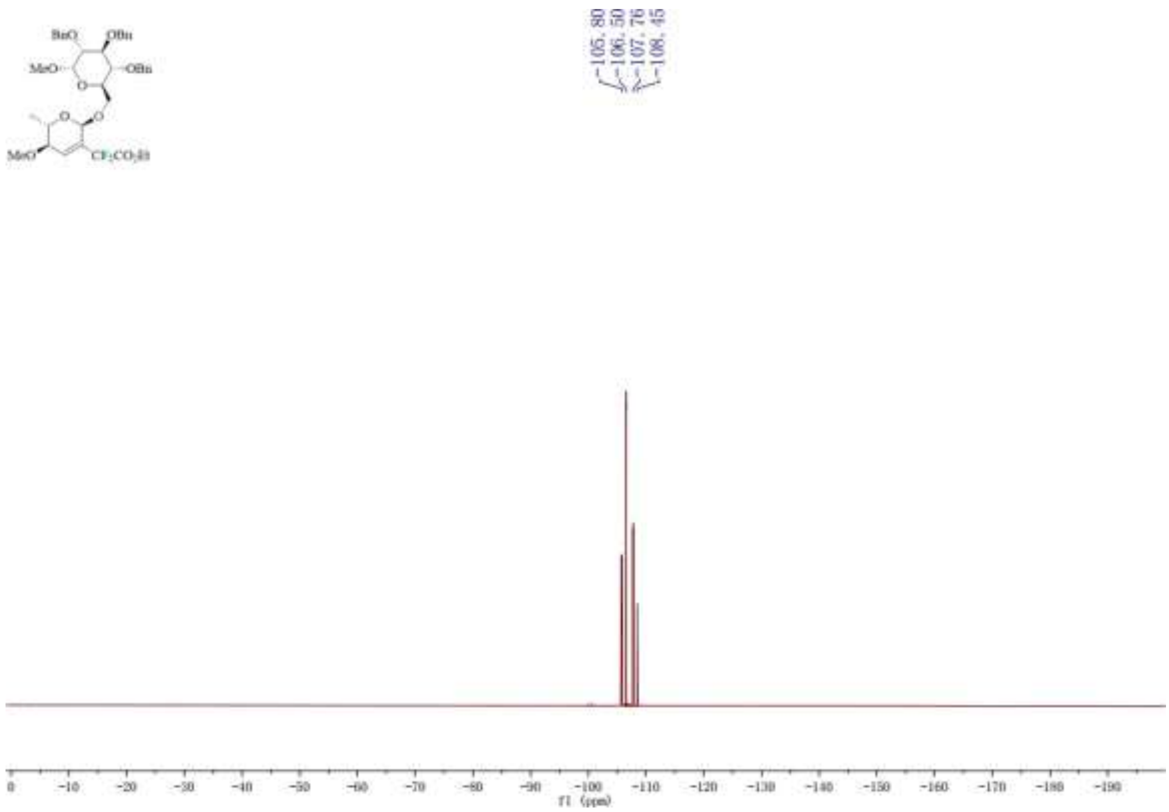

^{19}F NMR spectrum of Compound **4m**

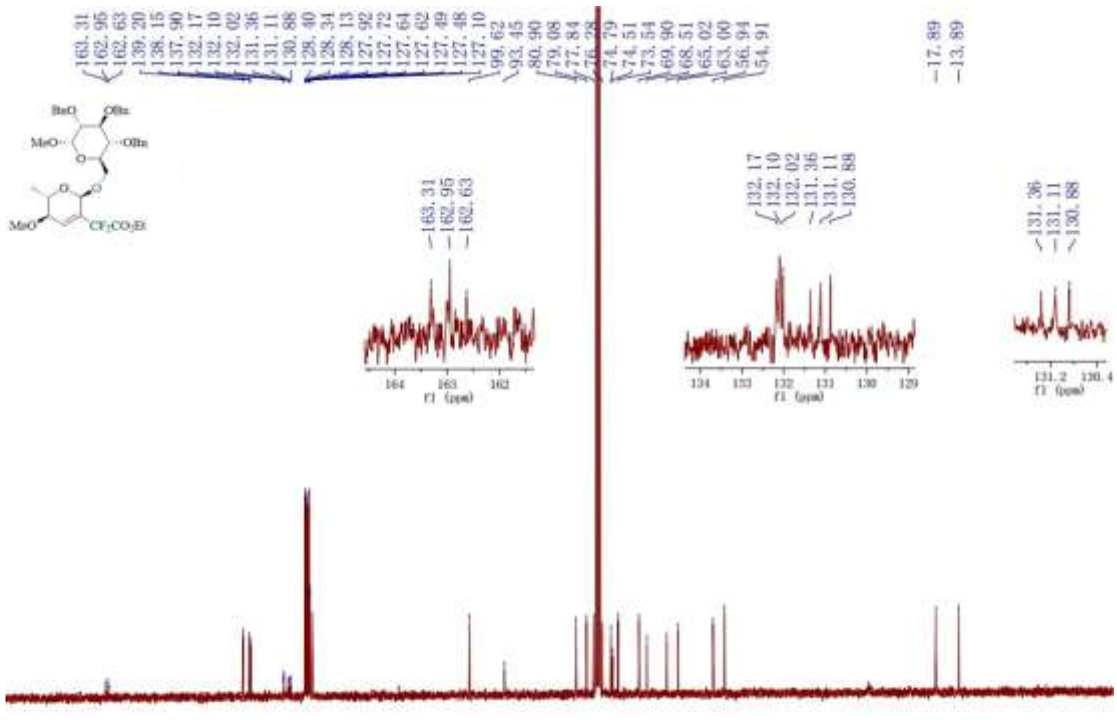

^{13}C NMR spectrum of Compound **4m**

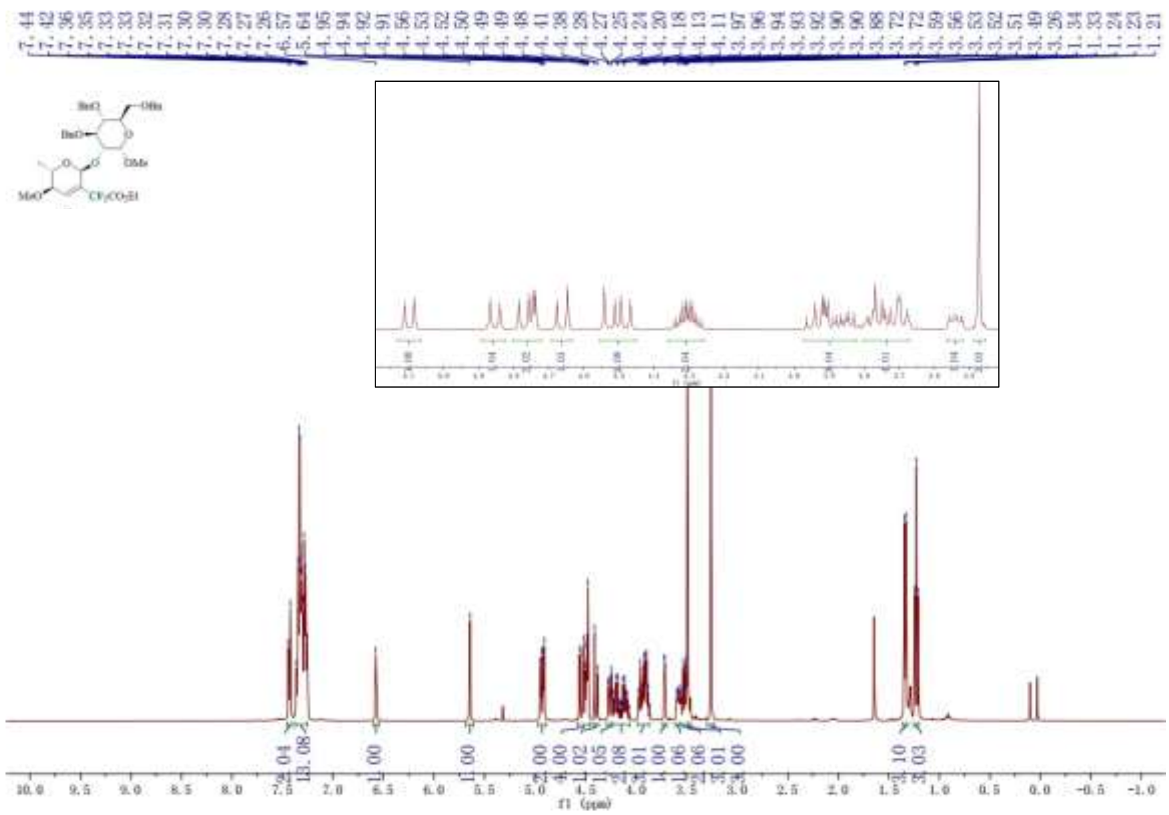

^{1}H NMR spectrum of Compound **4n**

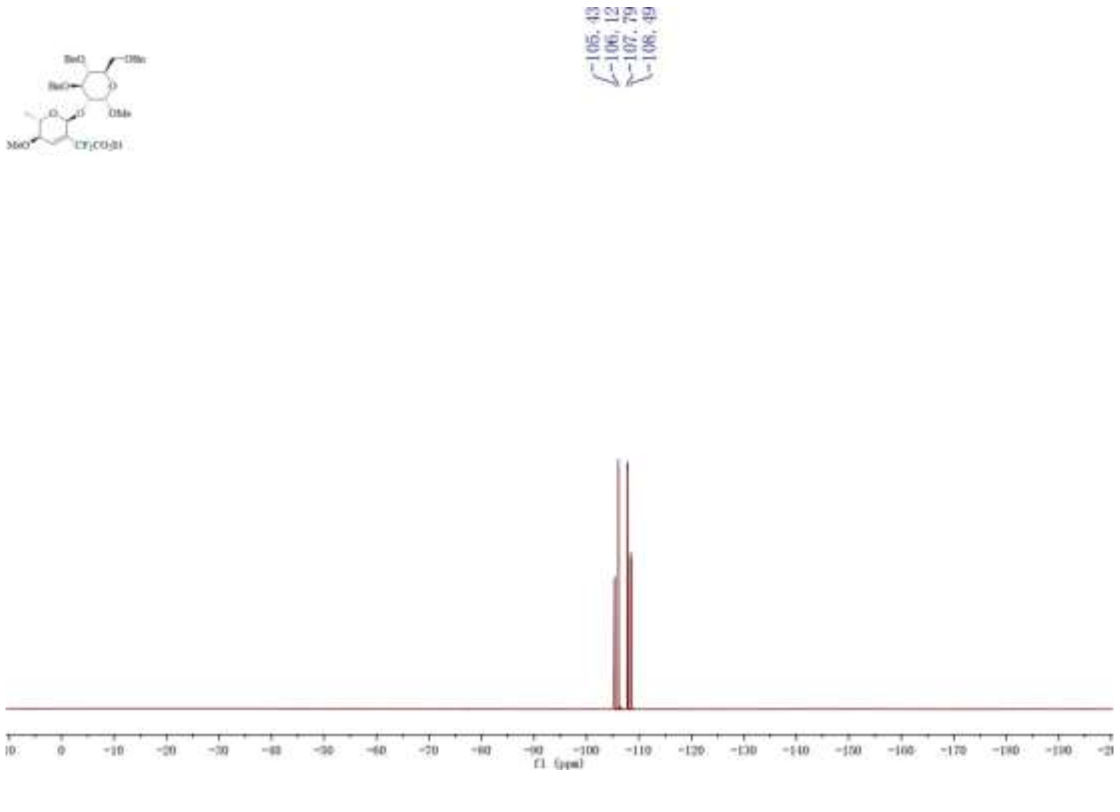

^{19}F NMR spectrum of Compound **4n**

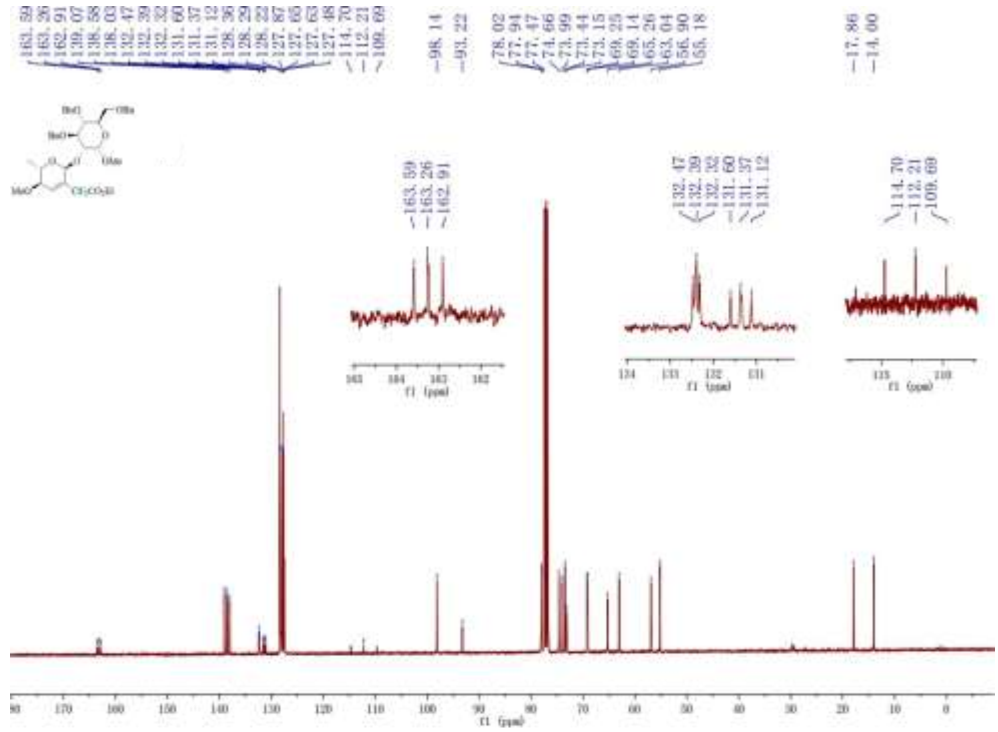

^{13}C NMR spectrum of Compound **4n**

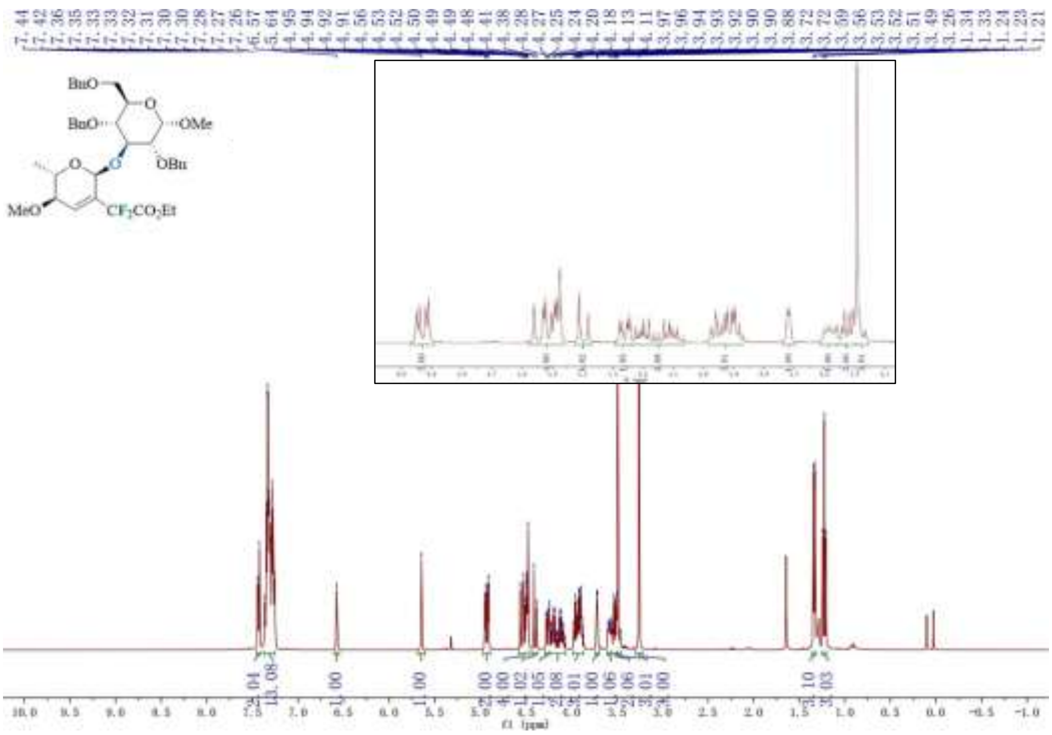

^{1}HNMR spectrum of Compound **4o**

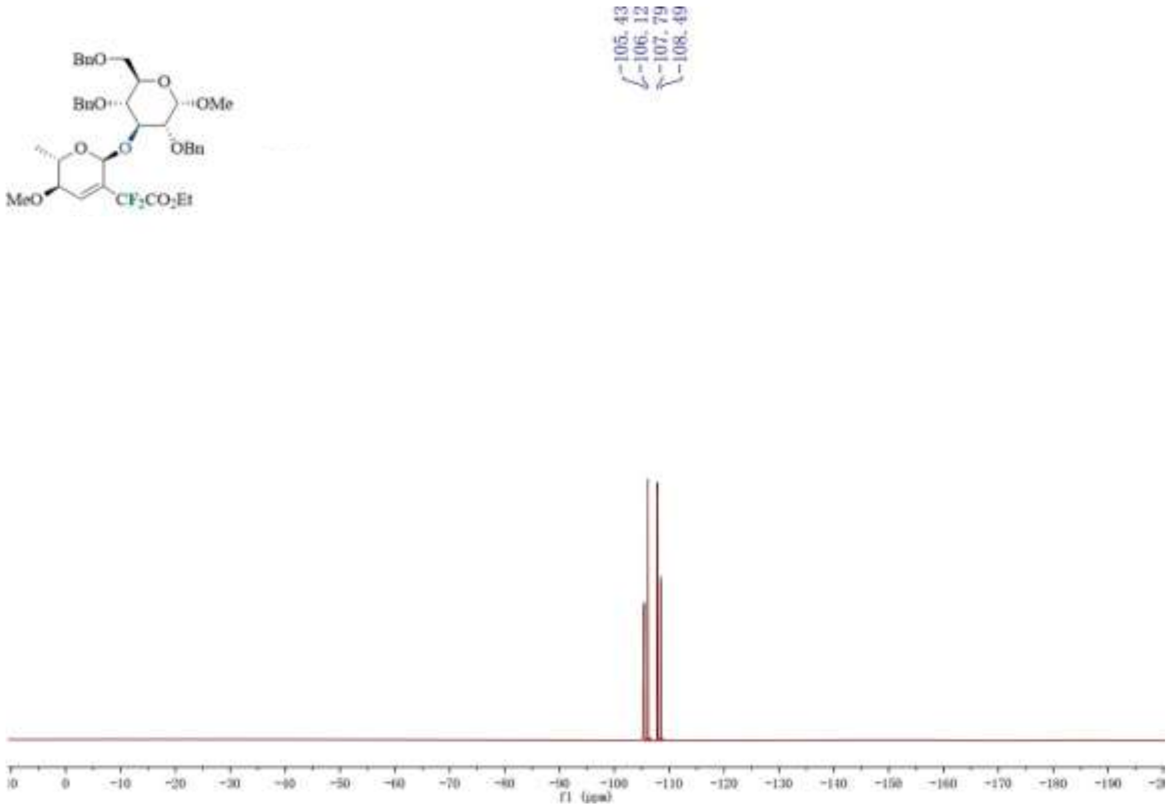

^{19}F NMR spectrum of Compound **4o**

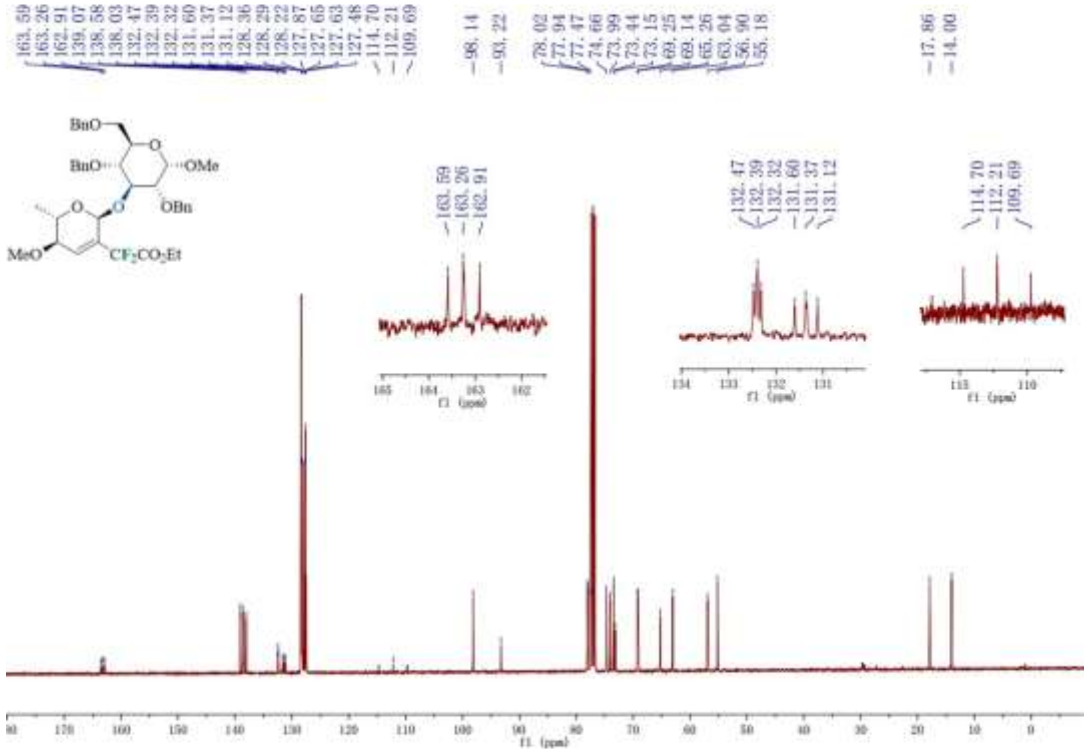

^{13}C NMR spectrum of Compound **4o**

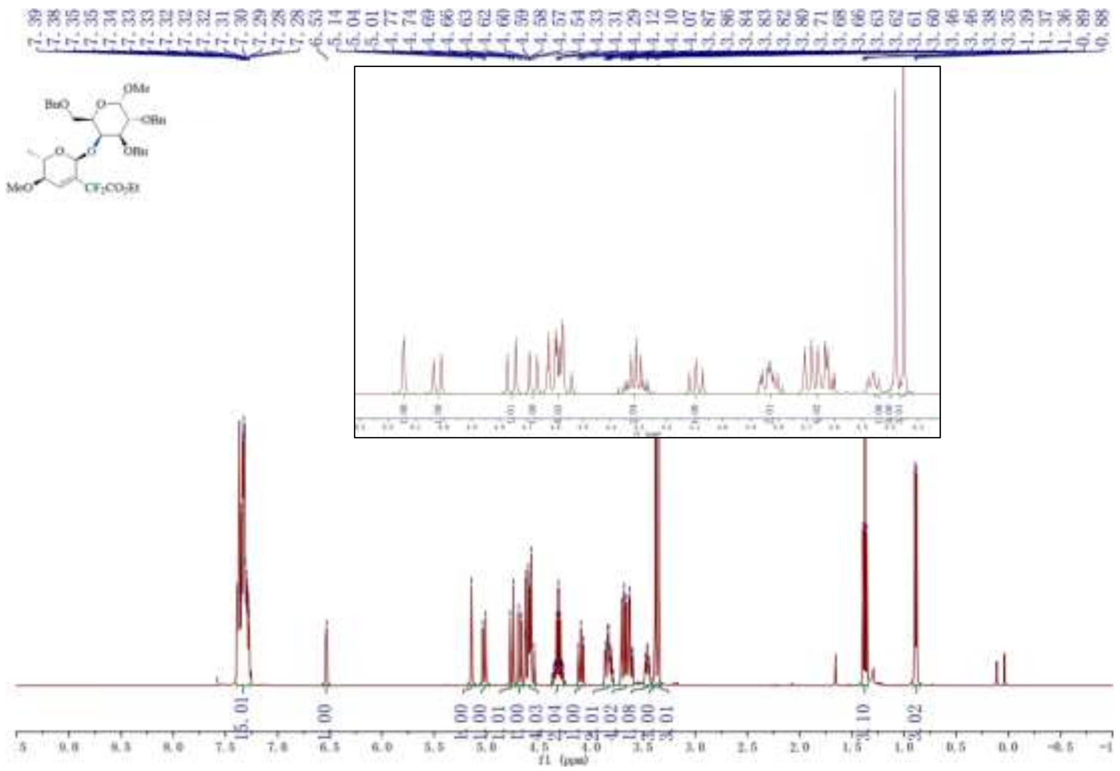

^{1}H NMR spectrum of Compound **4p**

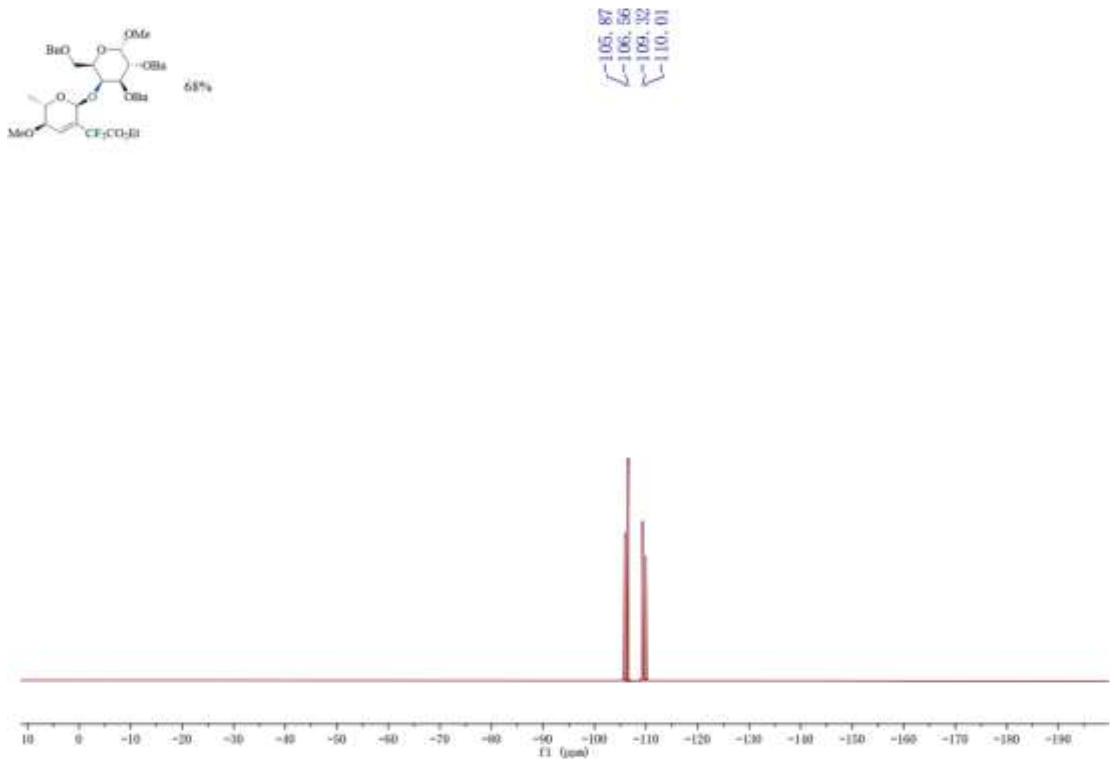

^{19}F NMR spectrum of Compound **4p**

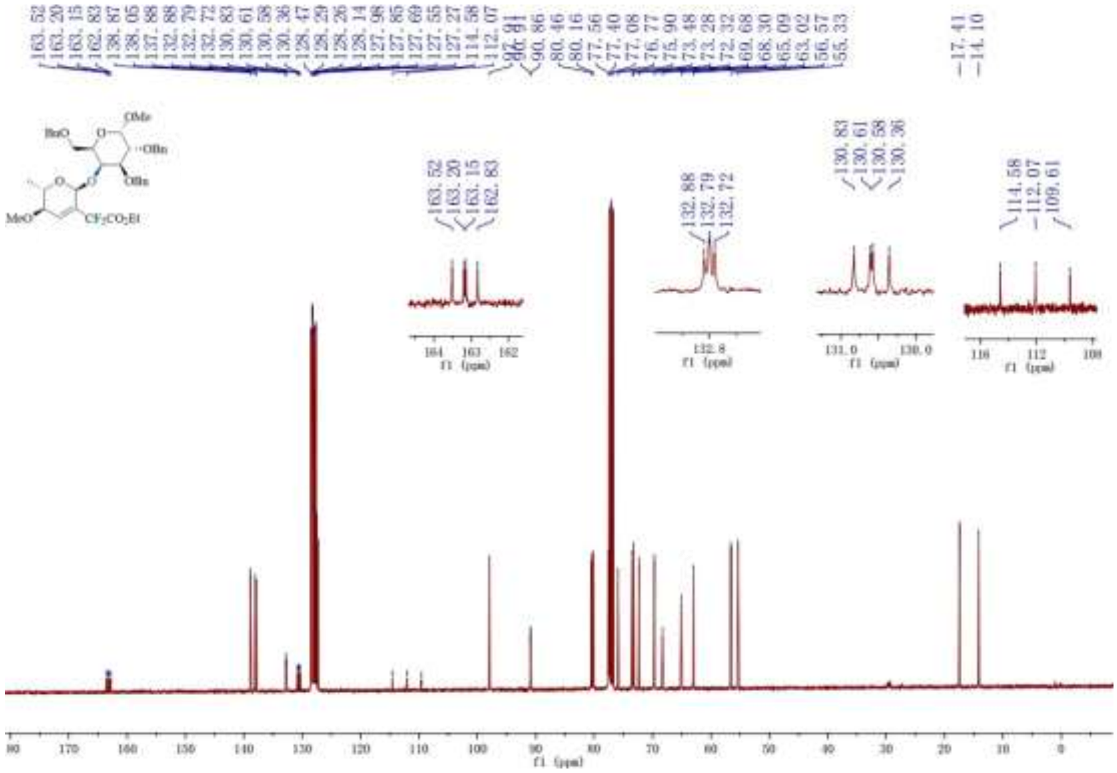

^{13}C NMR spectrum of Compound **4p**

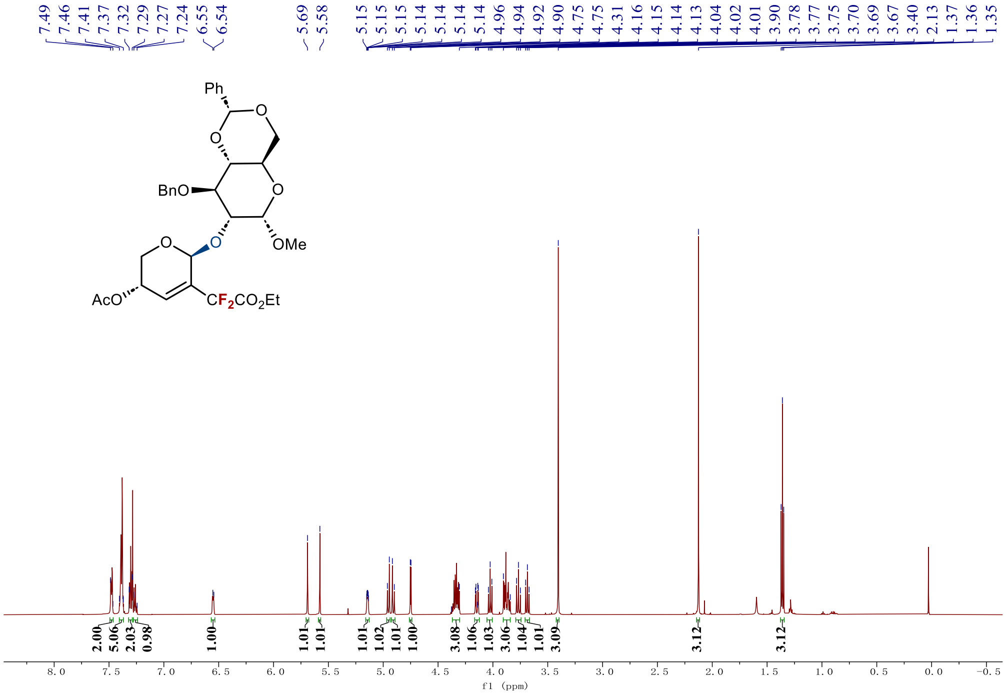

^{1}H NMR spectrum of Compound **4q**

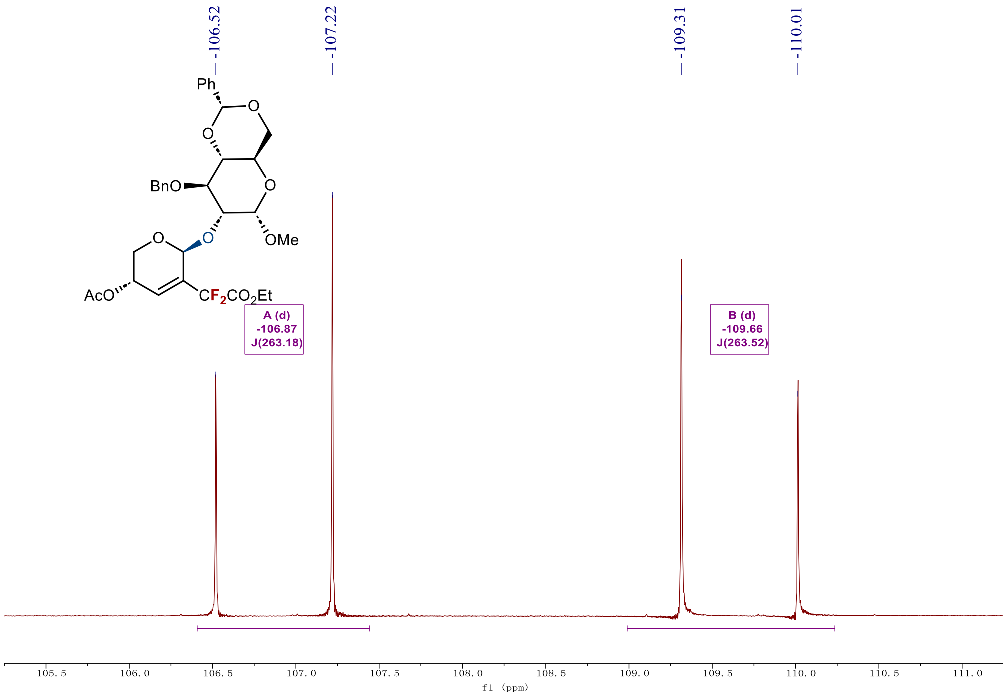

^{19}F NMR spectrum of Compound **4q**

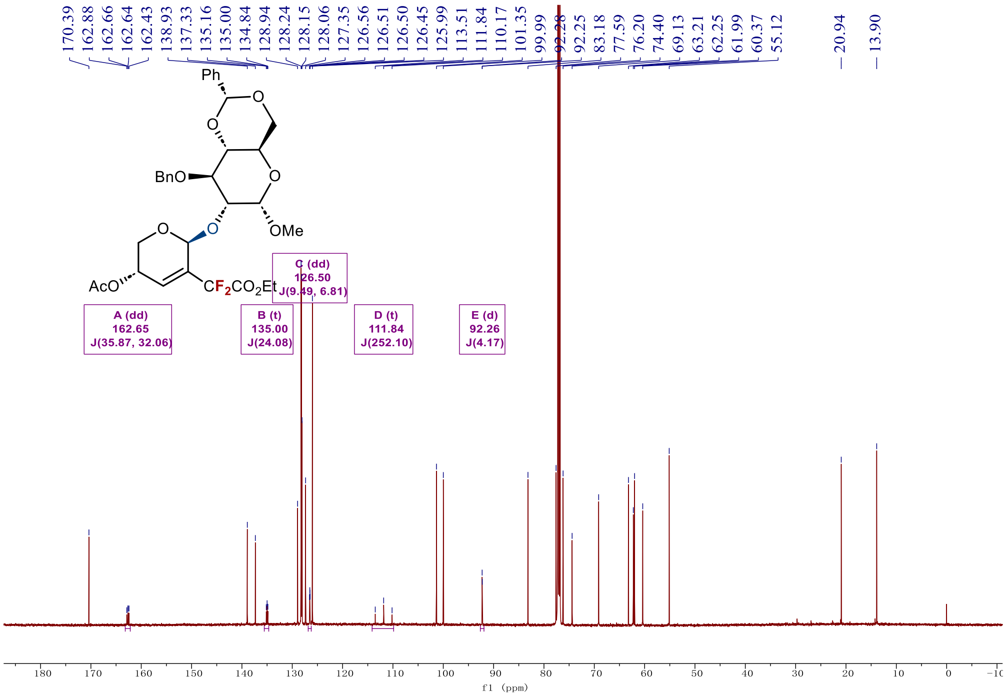

^{13}C NMR spectrum of Compound **4q**

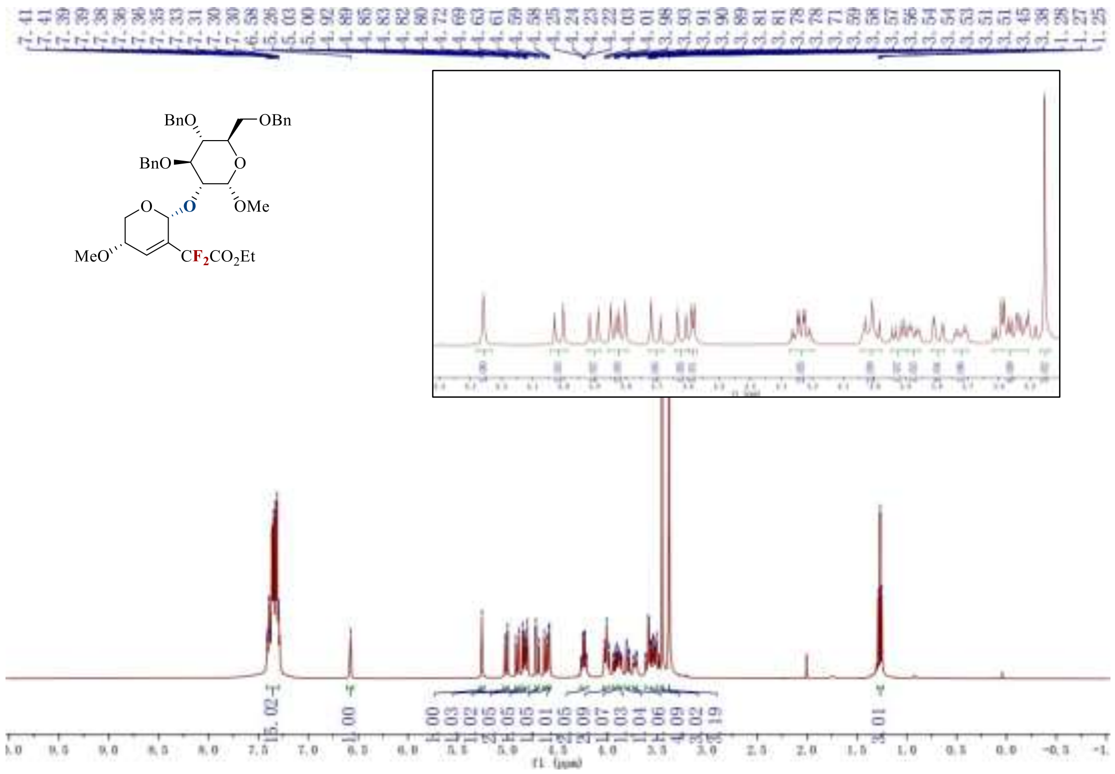

^{1}H NMR spectrum of Compound **4r**

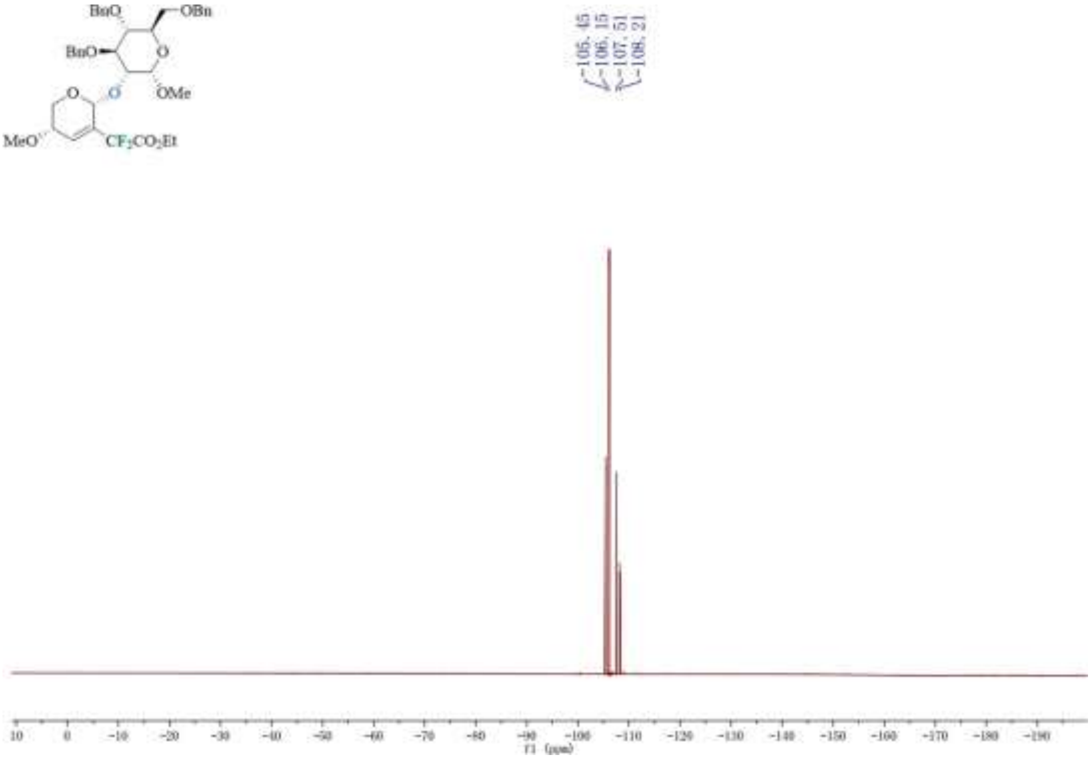

^{19}F NMR spectrum of Compound **4r**

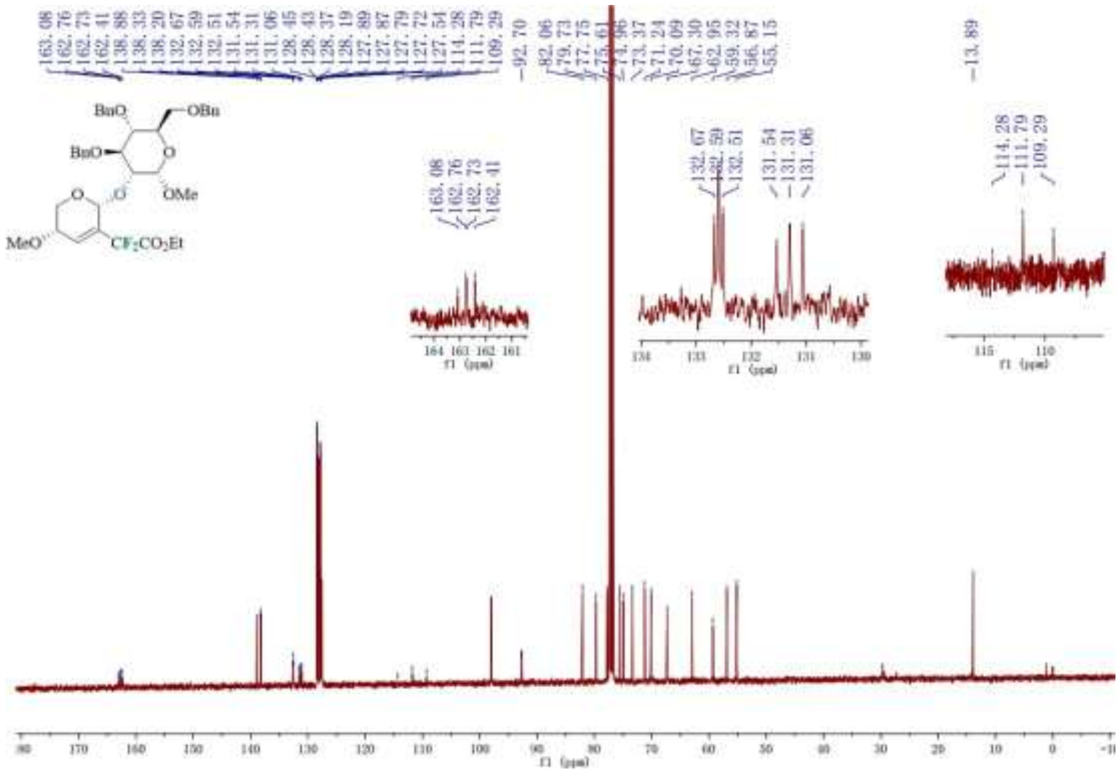

^{13}C NMR spectrum of Compound **4r**

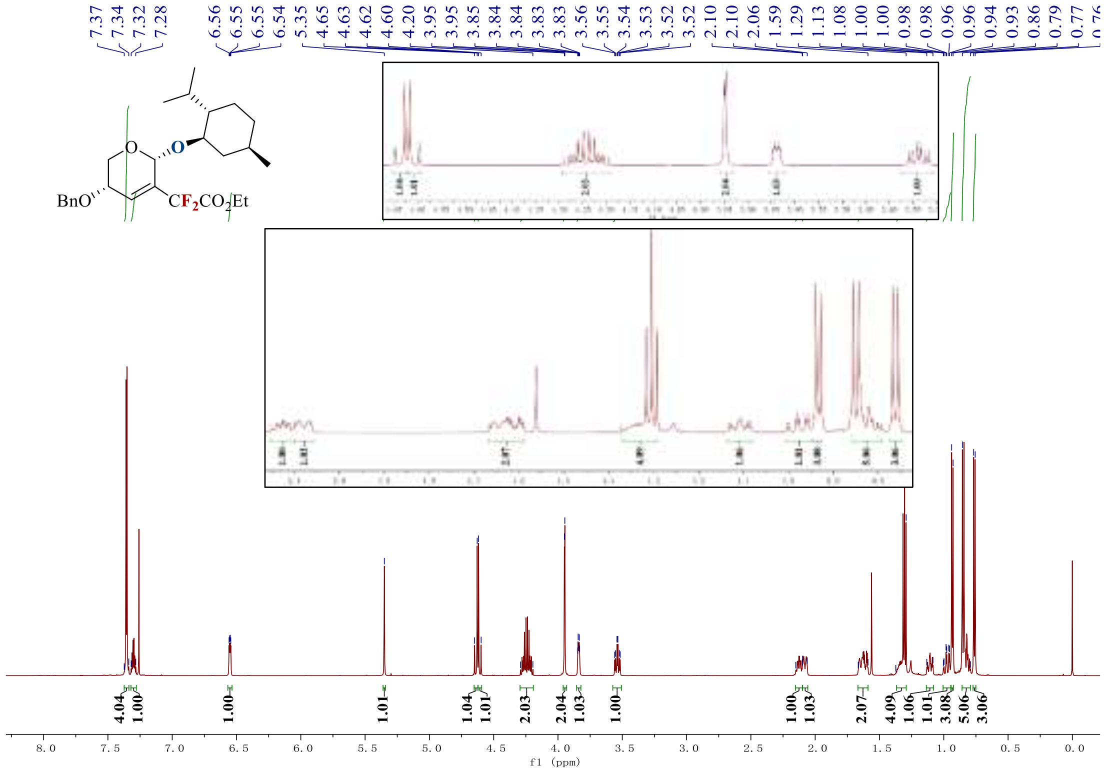

^{1}H NMR spectrum of Compound **4s**

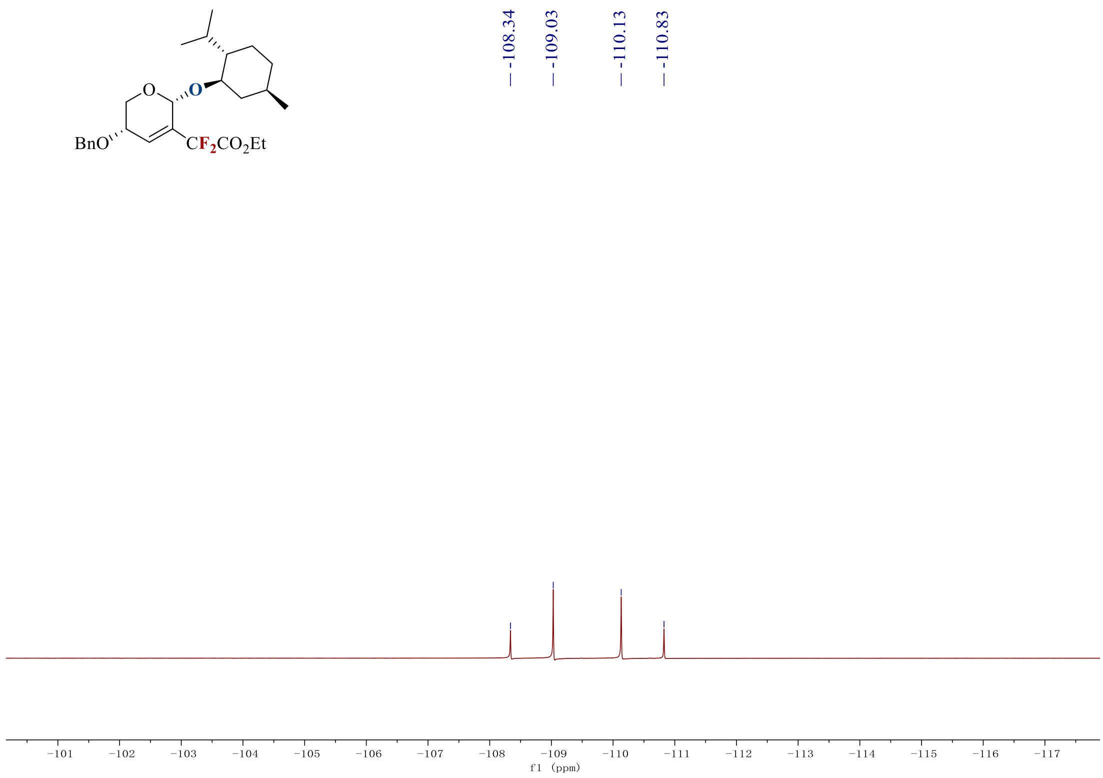

^{19}F NMR spectrum of Compound **4s**

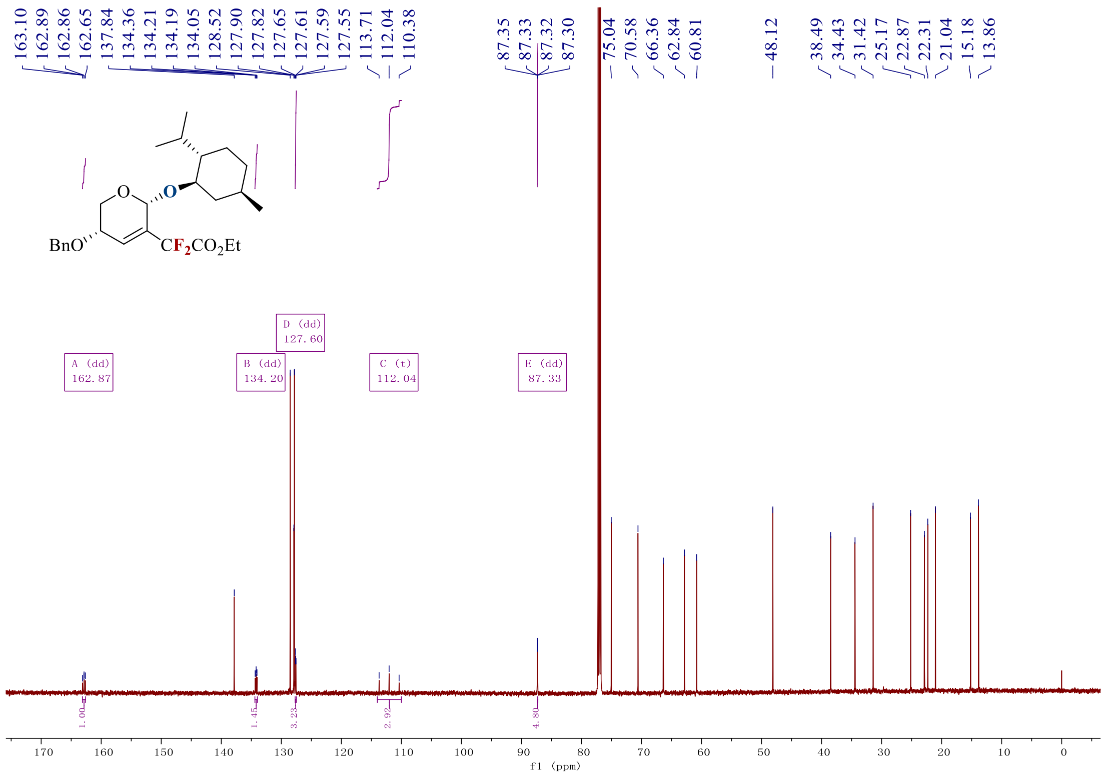

^{13}C NMR spectrum of Compound **4s**

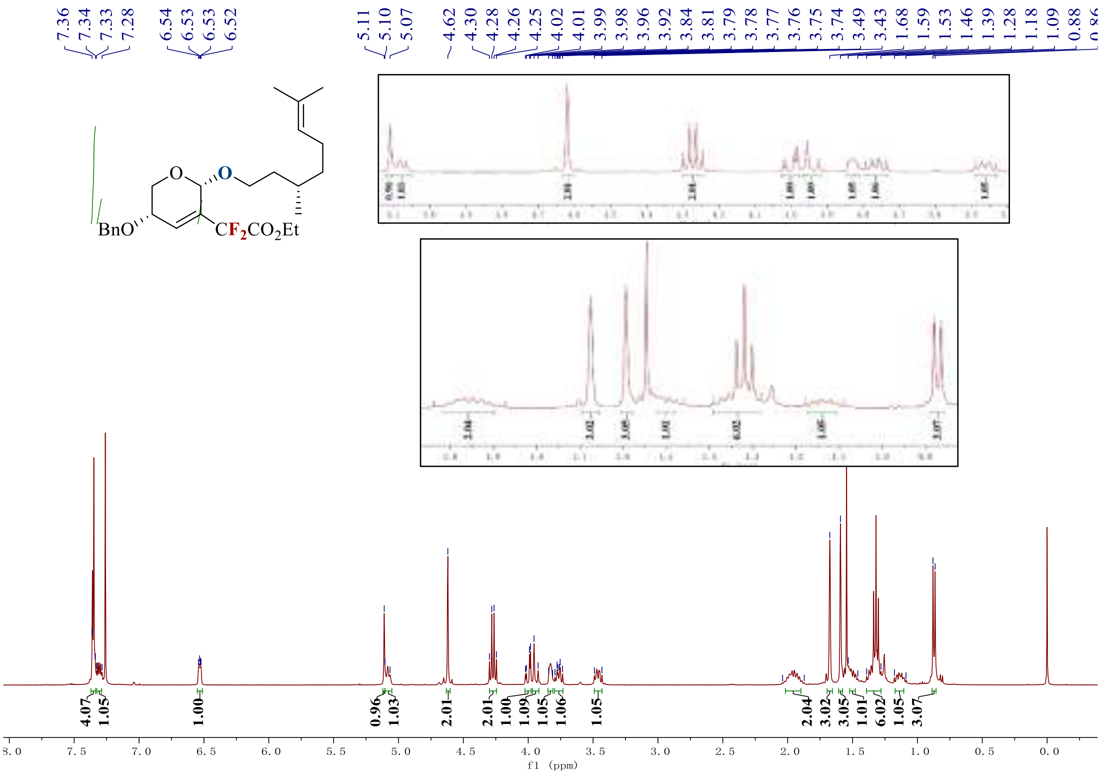

^{1}H NMR spectrum of Compound **4t**

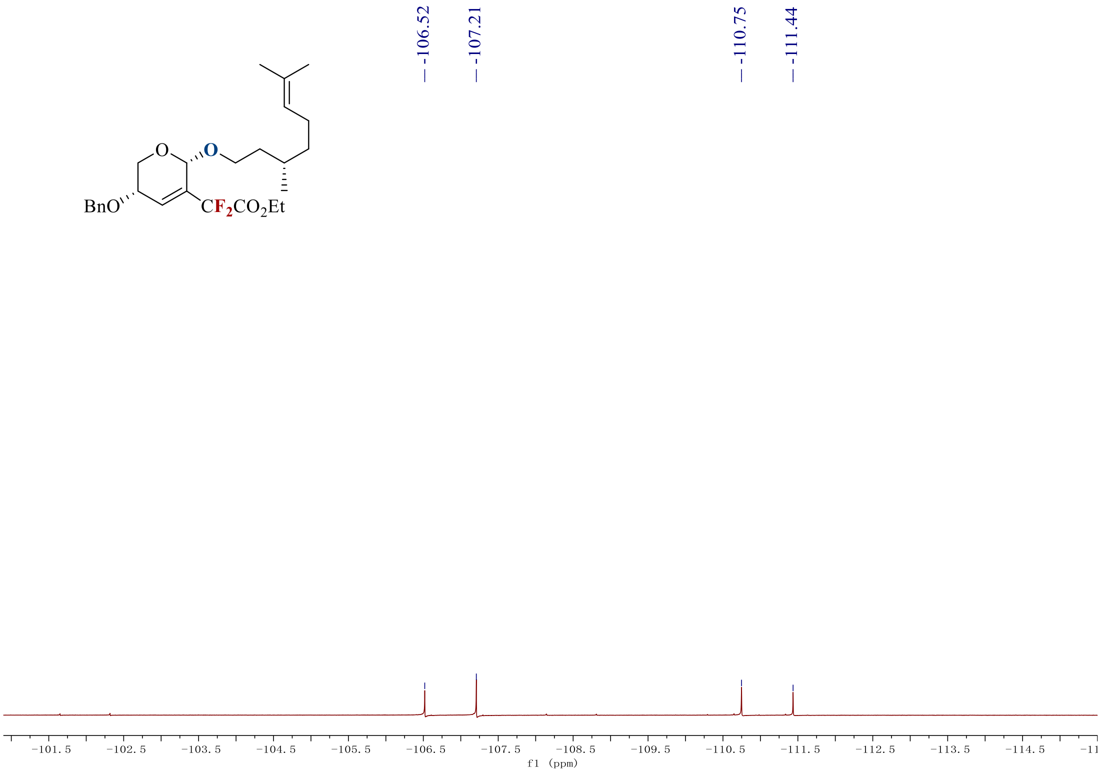

^{19}F NMR spectrum of Compound **4t**

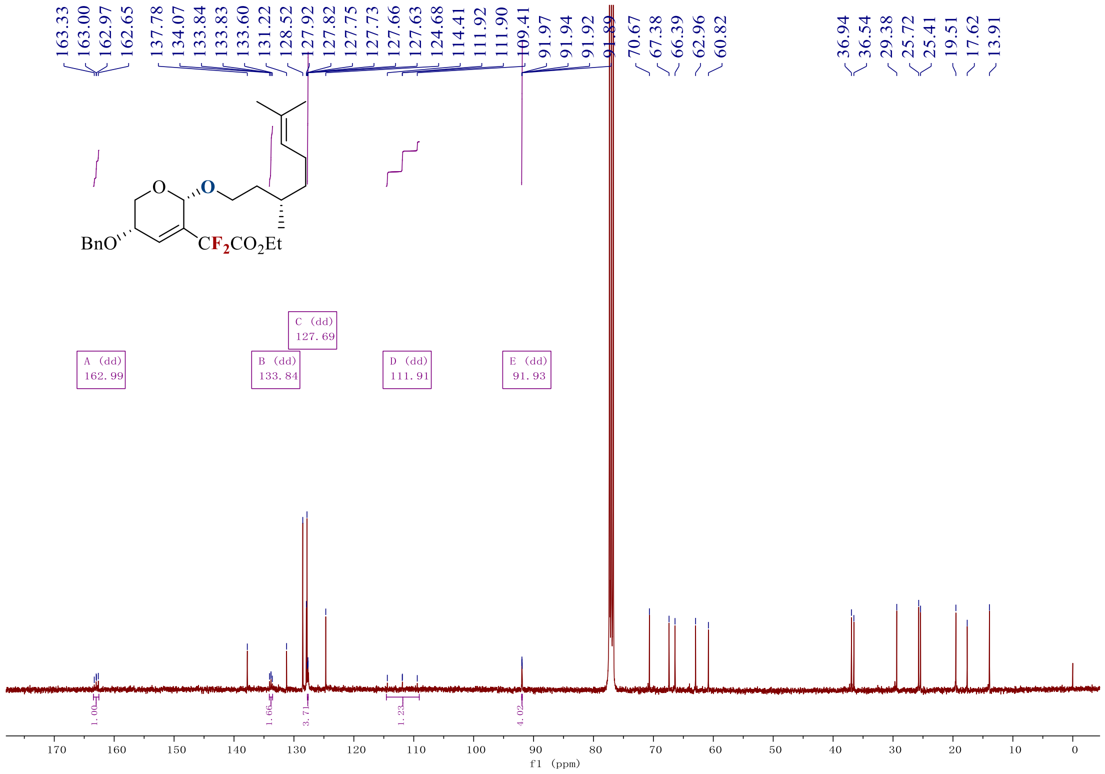

^{13}C NMR spectrum of Compound **4t**

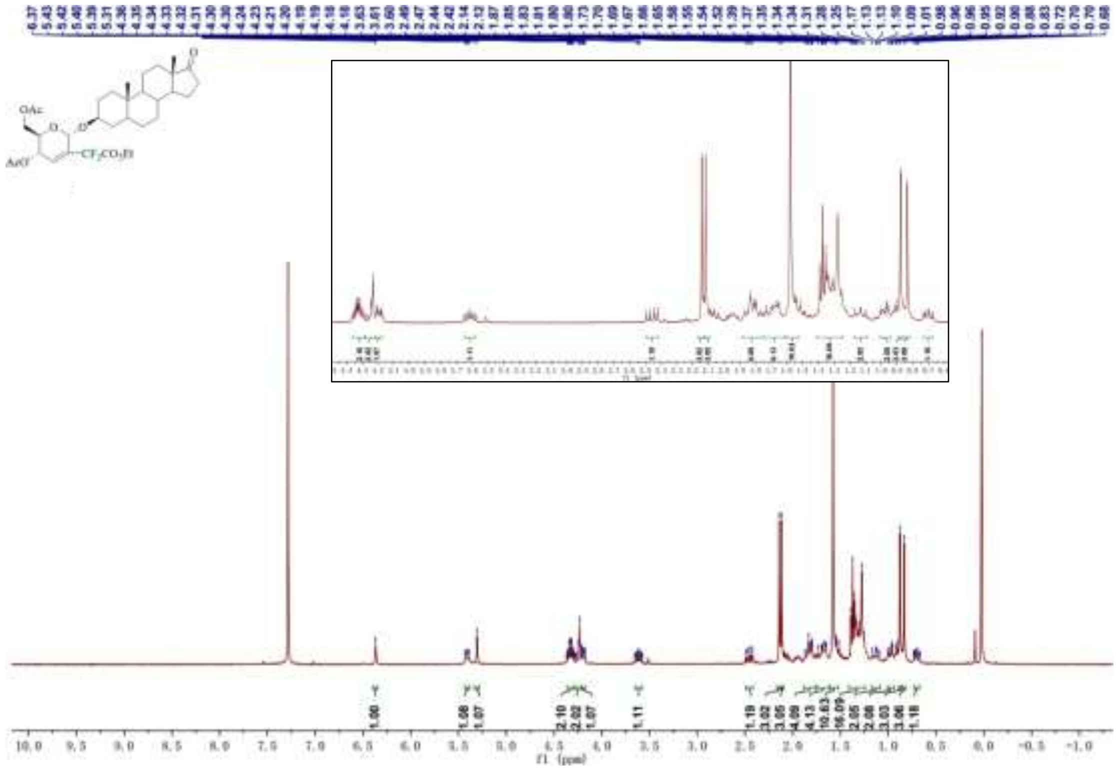

^{1}H NMR spectrum of Compound **4u**

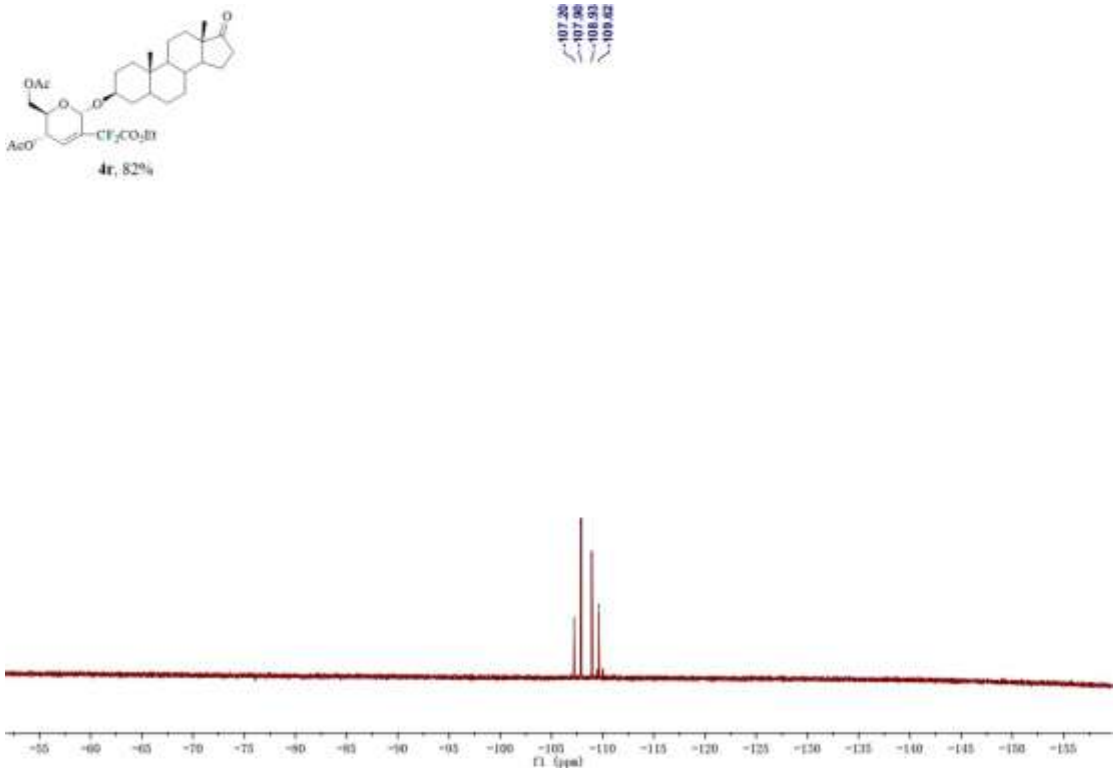

^{19}F NMR spectrum of Compound **4u**

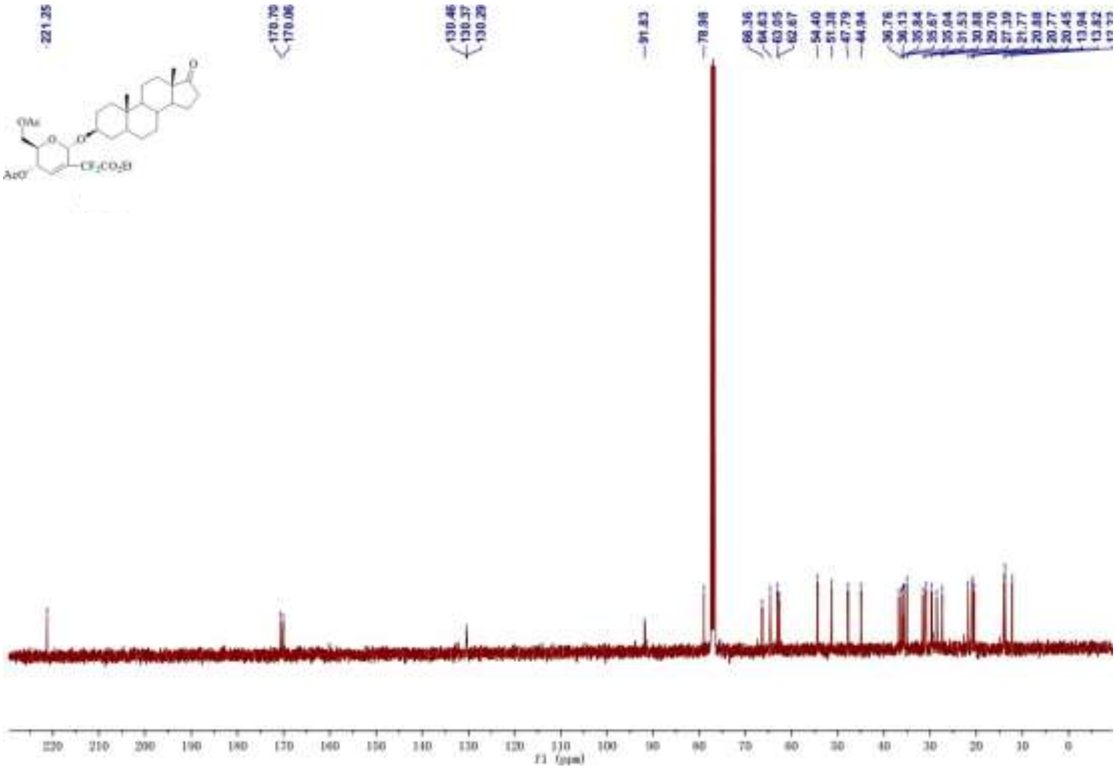

^{13}C NMR spectrum of Compound **4u**

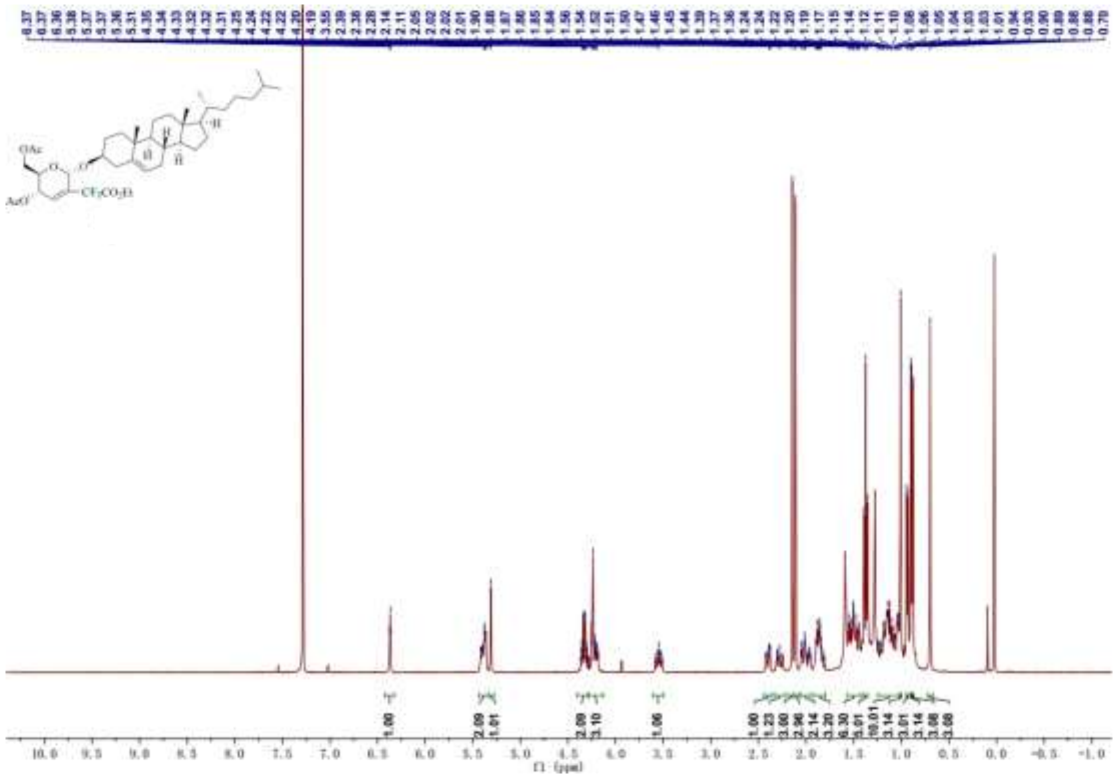

^{1}H NMR spectrum of Compound **4v**

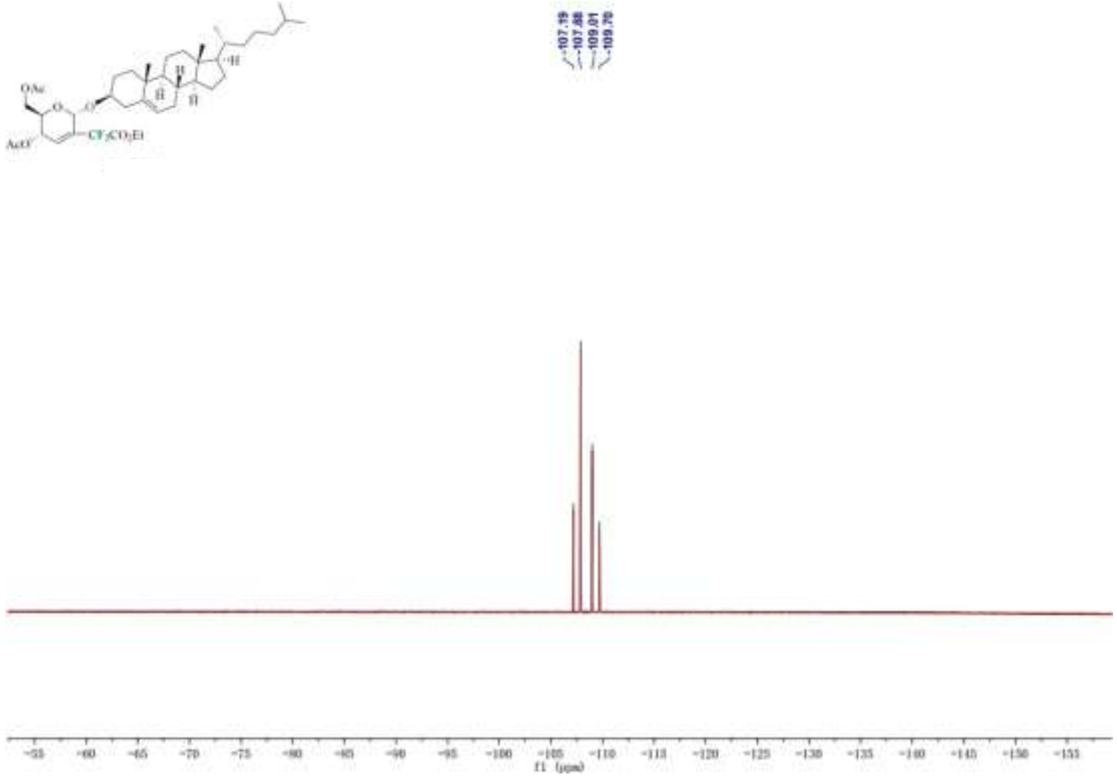

^{19}F NMR spectrum of Compound **4v**

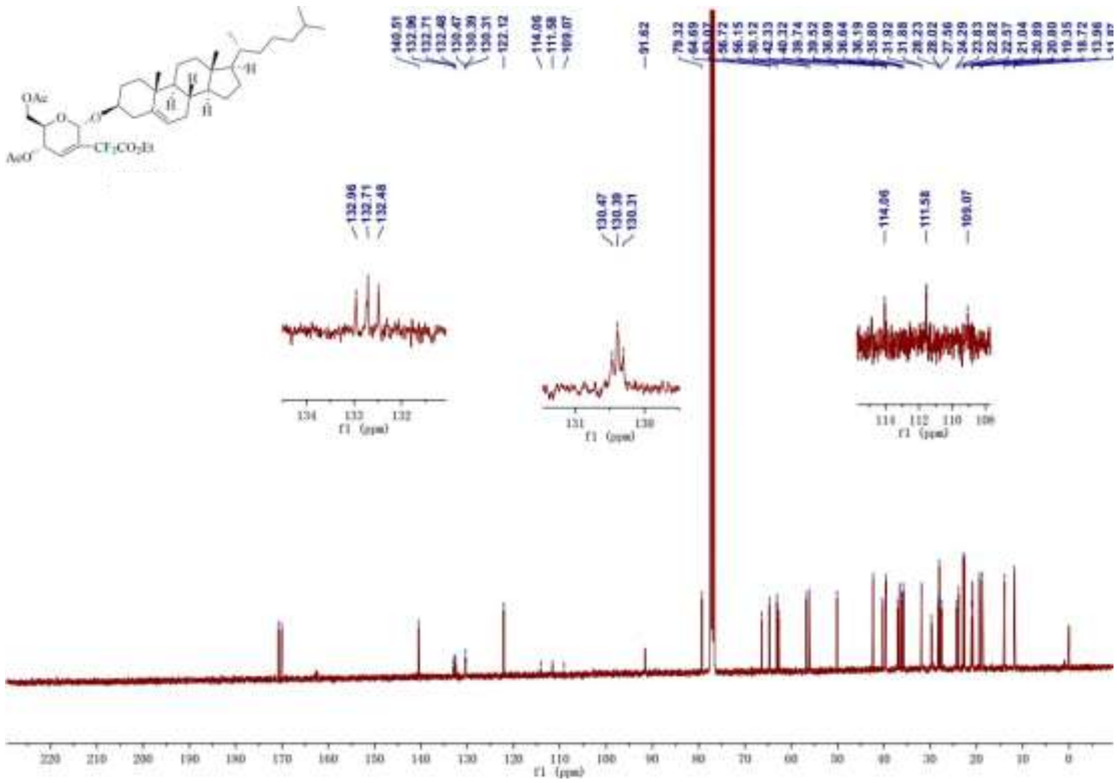

^{13}C NMR spectrum of Compound **4v**

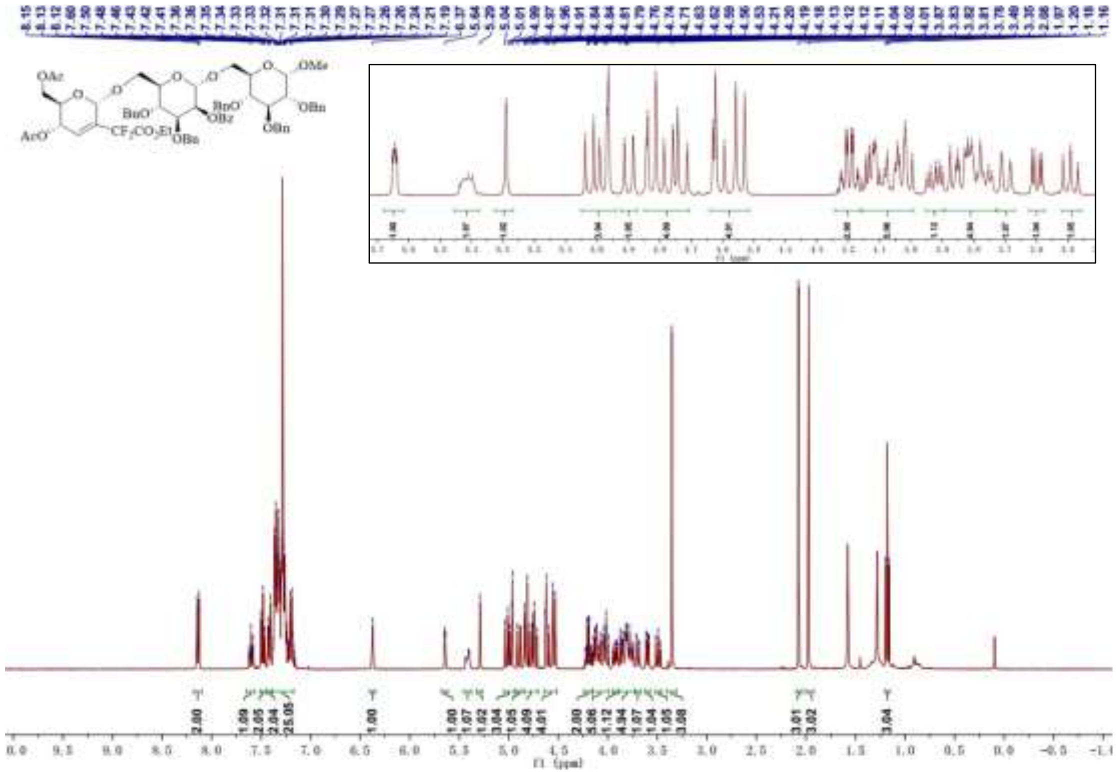

^{1}H NMR spectrum of Compound **5c**

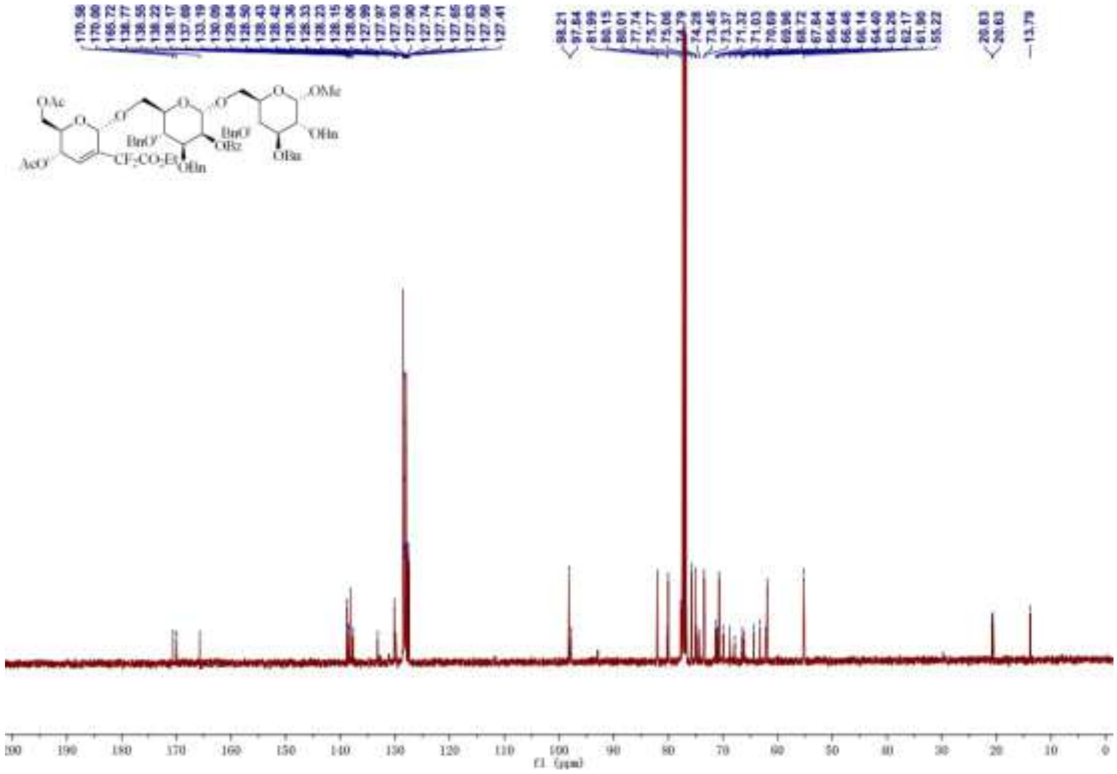

^{13}C NMR spectrum of Compound **5c**
